# Supplementary material for: Diminutive, degraded but dissimilar: Wolbachia genomes from filarial nematodes do not conform to a single paradigm
Source: Microb Genom. 2020 Dec 9;6(12):mgen000487. doi: 10.1099/mgen.0.000487 (PMC8116671; doi:10.1099/mgen.0.000487)
Supplement: Supplementary material 1 [file mgen-6-487-s001.pdf]

Supplementary files 1: Species and Authorities

Nematodes:

Brugia malayi (Brug, 1927)

Brugia pahangi (Buckley & Edeson, 1956)

Cercopithifilaria japonica (Uni, 1983)

Cruorifilaria tuberocauda Eberhard, Morales and Orihel, 1976

Dirofilaria (*Dirofilaria*) *immitis* (Leidy, 1856)

Dipetalonema caudispina (Molin, 1858)

Dipetalonema gracile (Rudolphi, 1809)

Litomosoides brasiliensis Lins de Almeida, 1936

Litomosoides sigmodontis Chandler, 1931

Madathamugadia hiepei Hering-Hagenbeck, Boomker, Petit, Killick-Kendrick and Bain, 2000

Onchocerca ochengi Bwangamoi, 1969

Onchocerca volvulus (Leuckart, 1893)

Pratylenchus penetrans (Cobb, 1917)

Wuchereria bancrofti (Cobbold, 1877)

Arthropods:

Drosophila melanogaster Meigen, 1830

Folsomia candida Willem, 1902

Carposina sasakii Matsumura, 1898

Ctenocephalides felis (Bouché, 1835)

Nomada flava Panzer, 1798

Culex quinquefasciatus Say, 1823

Trichogramma pretiosum (Riley, 1879)

Nilaparvata lugens (Stål, 1854)

Laodelphax striatella (Fallen, 1826)

Armadillidium vulgare (Latreille, 1804)

Cimex lectularius Linnaeus, 1758

Vertebrates:

Ateles paniscus (Linnaeus, 1758)

Canis familiaris Linnaeus, 1758

Carollia perspicillata (Linnaeus, 1758)

Chondrodactylus turneri (Gray, 1864)

Hydrochoerus hydrochaeris (Linnaeus, 1766)

Meriones unguiculatus (Milne-Edwards, 1867)

Supplementary files 2: Validation of the circularization of the *Wolbachia* complete genomes by PCR amplification.

Table. Primers and PCR condition. In bold is indicated the primers which valided the circularization, all the other primers were design to verify previous de novo assembly. The primers which were tested but the PCR product were not sequenced are indicated in grey.

reference	position (bp)	name of oligo	sequence (forward)	sequence (reverse)	size of sequence	annealing T
**wCtub**	626,596-627,580	wCtub_tig1D1	CAGCCAGCATGATAGGGTTA	GGAAGGTTCTATGTTGCTTTGGAC	834 bp	65
	768,172-768,952	wCtub_N1201N5	ATGCAGGAGCGATAGGGATTGAAC	ATGGGCAAGTACCACAATGCGT	781 bp	70
	423,432-424,095	wCtub_N5N1205	TGCATCACCATGACGGGAATCT	ACAAGGAGTGGAAGGCAGGTTAGA	620 bp	70
	418,342-418,921	wCtub_N1205N268	GCTGAAAGCCTTGCATTTGCGT	TGCTCTGCATTCTGGAGTTACC	578 bp	68
	113,621-114,156	wCtub_N40N1206	TGAGGAGACGTTTTGGGAGACA	TTGGGAATGGTCACTACATGCC	493 bp	68
	108,955-109,492	wCtub_N1206N54	AGCTTCAAGACCATGGGATGAAGA	GCGTTAGTACCACCGAAGCCAAAT	500 bp	69
	774,966-775,932	wCtub_N54N1201	CACGGAGATGGCCAAGTATTACTG	TTGTATGAAAGGCGGTCAAGTGCT	856 bp	68
	348,986-349,631	wCtub_N268E	AACCTTTGTCCTCGTCCTGATG	TTCTACCCAATCAGGCAAAAGGC	608 bp	68
	**522,139-522915**	**wCtub_P341224**	**GTATTTACTGCAGTCGCTGGAG**	**GGGAACCTTAACACCTTTAGCC**	**772 bp**	**66**
	167,628-168,831	wCtub_P695900	GCACTGCCGCACTATTTATGCATC	AGGCACTCGAGCAATAGAAAGTGG	1200 bp	70
**wDcau**	**469,087-469,863**	**wDcau_UD**	**AGTGGTCGGTGGAAACGATT**	**TCATCCGCAAAACTTGAGTC**	**776 bp**	**68**
	683,984-684,538	wDcau_N4	AGGAATTATATACAAGACT	CAGGTGTAGCTTCGTACCTT	550 bp	68
**wDimm**	**309,052-308,471**	**wDimm_tig1UAC**	**AGTAAGCATTACACTGCTCT**	**AGCTGCATTTCTGGTTCGGT**	**582 bp**	**68**
	920,005-354	wDimm_tig1_919000	CGGTTATATTGCAGTCGTGA	ACTAGCCTAGAGGTAGGTGA	472 bp	68
	3,093-3761	wDimm_tig1_3000	CTGATGTTTAGCAGAAGCCT	CTGGCCATTCTTGCTGATCT	669 bp	68
**wLsig**	**910,348-910,915**	**wLsig_tig1UAC**	**TGCTGATGACCCTAAAGTGT**	**ACCGAATACTGCCAGGGTT**	**661 bp**	**68**
	166,186-166,892	wLsig_tig1_166000	CCACCTTGCTCTTGTATTCG	TGCTAACCAGCATAATCCGT	707 bp	68
	301778-302366	wLsig_tig1_301000	GGAAAAGTCGTAGAATCAGC	GCCTATCACCATATTCACCT	649 bp	68
	906,793-907,420	wLsig_tig1_906000	GCAAGCTTCAGAAGTAAGGA	ACCCCAACCTTTACTGCATC	628 bp	68
	966,162-966,738	wLsig_tig1_966000	GCAAAGTTATTTGATTGCGA	TAGCAAGTCTACAGCACATC	577 bp	68
	926,196-926,822	wLsig_tig1_926000	ATTATGGATTGGTCTTGACG	AGGGAGCTAAAGTCTCAGT	627 bp	68
	911,336-911,823	wLsig_tig1_912000	GGTCCAGCTTCAGGAGTAA	AACTCTTCCTGTCTACCTTT	488 bp	68

Amplicon sequences:

>wCtubN1201N5

aTAGCCTATGAcaCaGATAGTAGAAGCATTTGGCAAGCCGCATGTAAATGTGGGAACAATAGGGCACGTAGATCACGGGAAAACAACGTTAACGGCAGCGATAACGAAGTATTATGGGCATTTC
ATAGCGTATGATCAAATAGATAAGGCACCTGAAGAGAGAAAGAGGGGAATAACGATAGCAACAGCACATGTTGAGTATGAGACAGATAAGAGGCACTATGCACATGTTGATTGTCCTGGACA
CGCTGACTATGTGAAGAATATGATAGTAGGTGCAGCACAGATGGATGCAGCGATATTAGTAGTATCTGGAGTTGACGGGCCAATGCCACAAACAAGAGAACATATATTACTTGCAAAGCAAGT
TGGTGTTAAGTATATCGTTGTGTACATAAACAAAGCTGATGTAGCTGATCATGATATGATTGGTTTAGTAGAAATGGAAGTTAGAGAATTGCTGAGTAAGTATGAATTTCCCGGTGATGATGTTC
CTGTAGTGATTGGGTCTGCATTAAAAGCATTAGAAGATGAAGGTAATGAGTATGGGAAGAAATCAATAGGAAAGTTGATGGAAAAGTTGGATGAATATGTAGCAGTTCCTCCAAGGCCTGTAG
ATTTGCCGTTTCTAATGCCAATTGAAGATGTATTTTCAATACCTGGgCGTGGaACGGTAGTGACAGGAAGAATAGAGAAgGGAGAAATAAAGACAGGCGAAGAGATCGAGATAGTAGGGTTGA
AAGCaACgcgGAAgacAaTatGTACAGGGgTAGAAATGtTC

>wCtubN1205N268

CTGGTAgatTAgAAcTGCTCTTTTGTGacGTAaTAGCGATTTGTGTGTGAACACTACTAAAGGCTTACGGAAGTTTCGATGGATTTGCCTACGAAGAACATGAAAGTAATTTGCAGGAGTAGAGCA
ATTAACTACTTGCATATTATCCTCTGCACAAAGTTGTAAAAATCTTTCTATACGTGCTGAGCTATGCTCAGGTCCTTGTCCTTCATAACCATGAGGTAAAAGTAAAACTATACCACTAGATCTCAAC
CACTTTGTTTCTGCTGATGCAATGAACTGATCGATTATAATTTGTGCGCCATTTGCAAAATCACCAAACTGCCCTTCCCAAAGCACAAGTGAATATGGAGAATCAAGGCTATACCCATATTCGAAG
CCCATTACAGCATACTCTGATAGGGCACTATCTATAACTTCAAAGTAGGCTTGTTCTTCATTTATATTATTCAGTGGAGTAAACACTTCTTCTGTTACTTGGTCAATAAGCTTTGAATGACGGTGAG
AGAAAGTTCCTCGGCCAGAATCTTGACCTGACAAGCGCACTTCTATTCCTTCTGTAAGTAGTGACGCaatgc

>wCtubP341224

TTCTAtTGtTTGtAGTATTTTCTTCTCTACCaAAAATATTCTTTCTATGACCTTTGAATAGTACTATTACCGCCCATCCTCCTGTCCCTGGATTACCAGAACAGGCTCCATCTGTGTATATTGTTACTTCC
TTTTTGTTCATCTTTTTACTATGTAGATAGATAAAGTATATATTTGCTTCTACGAAAGTTCAATTTTAATGAGATTTATATGCTAAGGCAAACTTAAACTGTATTTTTAAGTTTTACTATAGAAACAA
AAAAAAGCCCAACAACGAGCAACATTAATAGAAAAAGCAAAAAGTAGTTTTTATATTTCTGCTGTTCTTTCATATCTAGAACCAATCGATAGAGCAAAAAATAATACTAGCAAACAAAGAAAATA
GGTAAGAAATTGAATAAGAAAACATTCTTTTTTGTGAATGGTAATCCTTGAACTTAATAATAGATATAGCATACCAAATAAAAACACAACCTTCAAGAATTGCTATACTCAGGTAAAGTAGAACAT
TTTTTAAAAACAGTGATGGAAGTAAGCTAATTAACACTAAAAGTATACTATAAATTAGTATATATTTTCTTGTTCTCTCTGATCCGTAAACAACATTAAACATTGGAATTGATGCTTTTGCATATTCT
TCAGACTTGTTTAAAGACNGAGACCAAAAGTGTGGTGGAGTCCATATAAAAATTATTAAAAATAGAATAAAACTCTCCCaACTAACAGCATTAGTTACGGttGCCCAACCaATCATTGGagaAGAA
AGCACcTGaTGCGC

>wDcauUDF

agGGaagAAgtGCTCATAGTCTTGGTTCGCCGCTAGATCCaAGACTTACTTTTAATAATTTTGTAGTAGGAAAACCGAACGAATTAGCATTTACAGCTGCAAAACGTGTAGCAGATTCTATAGATCC
AATATCAGGGAGTAATCCTCTTTTTTTATATGGTGGAGTGGGGCTTGGTAAAACACACTTAATGCATGCTATAGCTTGGTACATCATTAATTCTCTTCCAATGAAAAGAAAAGTAGTATATTTATC
AGCAGAAAAAATTCATGTACCAATATATTACAGCGTTACGAAGCAAAGATATTATGTTATTTAAGGAACAATTTAGATCAGTAGATGTGTTGATGGTAGATGATGTACAATTTATTAGTGGTAAGG
ATAGTACACAGGAAGAATTTTTTCATACTTTTAATGCGCTAATAGATCAAAATAAGCAGTTGGTTATCTCTGCTGATAGATCTCCTAGCGATCTTGATGGAGTAGAGGAAAGGATAAAGTCTAGG
CTTGGTTGGGGATTGGTAGTGGATATTAATGAAACAACTTTTGAGTTAAGACTTGGTATATTGCAGGCCAAAATGGAACAAATGAACATGTATGTTCCTGATGATGTCCTAAAATTTTTAGCAAG
AAATATAAAGTCTAATATAAGAGAATTGGAgGGGGCCTTAAATAAgGtTGCTCATACTTTATTGATTGGAAGAAGTATGACAGTAGAGTCAGCTAGTGAAACCCTAGCAGATCTTCTCaGATCAA
ACCaTaAGCCAATTACAATAGCA

>wDimm_tig1UAC

ACTAGTCTATATTAAATATAACAGCTACTACTTATCTTTTCTTGCATAATTTTTCCGTATTTTTATATTATATATACAAACTCTTCTGAATCTAGTCATAGTATTGATACTCTTAATATTTCGCACTACT
GAAAACCAGCTTATACATTAGTCCACCAAACGAAAACCCTTGATAATTACACAAAATATTCCTCTACAAGCTGAAGCACACCCTCACCTCCAATTAACAGTACAAAAAAGCATATTCTATTTCATCA
AAACTCAGCTATTTTTTTGTACCTACCTAAATTTATTCATCCACTGCTTTTTTGCAGGCTACAATTACAAGTATTACACAGTAGTTATACTCAATGCTCATATTTTTCTCATGTTATTTAATCGGTTACC
CTACTCTTCTTCTACTACCACATCCAAATAATATTTCCTTTATAACATGAGCAGTACCACAGACAATTGGAGACTATCCTAACCATAAGTTGAATGAACCTGAAGTTTTTTTAATCTTACCTATACAT
TACCTTTTGTTTGGTATCAAGGCTATAAATATTCCATTACTAAATTTATCCACCAAAAAGAACTCAA

>wDimm_tig13000

CTTTATCATAGCGTTTTACTTGACTTGCTGTAGGTTTCATTATCTCTTCCACGCTGTTTAAAAGTTCTCTATGTAATATTGTATTTGTTACCTGGTATGTGTGGCAAATGTCTGCTTAATGTATTTTGT
TGTATAATTTTGTAGCACATTGCCTATTATGCTCATACTCCTTATCTATTTTTGCTTTAGCATTATGTTATTCTGTATGTAAGAAGATTAGTAGTTGGATAGTTATGGAGGTTTGACTTTGTTAAATTT
CAATGAATTGGGAGAATATTTATAAAGATGTTGCTTTTAATGACTAAAACACCTACAATCCTAAATTTTAAACAAGTTTTATGCCACGGAGGAATTTTTTAGTATGGGTGTTGGCATCACTGTTTTA
TGCATACCAATATGTGTTACGTGTGATTCCAAATATAATTGCGCCCGAGTTAATGACGAAGTTTAATGTAAATGTAGTTGATATTGGTCAATTTAGCGGCTTATACTATGTAGGTTATACATTAGC
TCATATACCTGTTGGTCTTTTTCTTGATAGGTCTGGGCTAAAGTTTGTTTTGCCTATATGTACTATTTTGACATTTGTTGGAACGCTGCCACTTATATGTTTTGATAGATGGTATCCTTCAATACTTG
GTAGAATAATCGTTGGAATTGGGTCATCTG

>wDimm_tig919000

AAACTTCAGTAAATCATTAAATATAGTAAGACCTGGCAGATCTTTCATCAGCACTTCAGTTGCACACTTTCTTAATACATAAAACCTTTCAAAAAGAAATAGAAAGACTCTACATGCAATCCTTTCC
CTCAAAGTCCTTTTTTTGCTCAAGTTGCTTCCTATAACAGACAAAATGTTTTCATATTAGATTTATCAAGGAAACTGTTGTCAAATATTTAATAAGCATGTGAATAACACTAAATTAATCTGTGAATT

TCATGTTGATATAAGATATTAACAGCTCCTTAACAACTTTTTCACCAGCATATACATAGTTGTACAGCAGTGTGCAACTGAAAAAAAATTTTCACAAAGAGAGATTACTAACAATCTAAACATTCTA
CTTATGCACATAAATTATAACAGAAAGTCAATAGTATATAAAAGTTATTAACACACTTATTCACAAATTAGGTAAAGAAAATCTTCACCT

>wLsig_tig1UAC

TTTTATCTTACTACACAATCCAATACTAATGACTAGAGCTCCATGATTGGAAATAGTGTGACAGCTCACTAAAAACAATAAAATAAAAAATATTAGCACTTGCATCTCTATTAATAAATCACTTAAA
GCTACAAACATTAACAAATCACCTGTCGTCAAATTTTGTAAAAGTTAAATTGACTGATTTTTGATATCGCTTAATATAATATTTACTTCAGAAAATAATAGGTGTTCTATGTTATTGTTAGAAGCAG
ATCCAATATATAAACCTTTTAATTATCCTTGGGCGTATGATGCCTGGTTACAGCAGCAAAGAATACATTGGATACCAGAAGAAGTTCCTCTTGCTGATGATGTGAAAGACTGGAAAGCTAAACTT
TCGAATGTAGAAAAAAAATTTACTAACTCAGATTTTCAGGTTCTTTACTCAAGCAGACATTGAAGTAAACAACTGCTATATGAGACATTACTCAAATATATTTAAACCAACAGAAATATGCATGATG
CTCGCAAGCTTTTCCAATATGGAAACCATACACATTGCAGCCTATTCTTATCTTTTAGATACAATTGGCATGCCAGAAAGTGAATATCAAGCATTTTTAAAATATGATGCTATGAGAAAGAAATAT
GAATATATGCTAGAATTTGAGGAGTGTAAA

>wLsig_tig1_166000

ATTTGTATTTTTTCTTATATCATCTAAACCTTTAGCTAATTTTGCATTGAAATTCTCTGTGAGTTTTGTAGCTAGTGCTTTTTCATTTTTGTTAAAAGCTAGTATATAGCATTGAAATCAAAATTTTTAA
GTAAGAAAATCATAGAGTCAGCAAAAAAAATGCTTGAAGGAAGTGTAAAATAATGCATATGTTGCAAAAAAACACTAAATGCTGTAATTGTAGCAAAAAAGTACTGTGTAACAGCTGCAGCAAA
GTTTCATTTAAAAGAAGAAAATTGTTTGCTCTTCTTAAGATCGTAGAAAAACTAGATTGAATCAGAGTCGGTTTGAATGAGTTGAAGTATGAATGCAAGAACATTCTGATATTTTCATTAAAGAAA
TGAGATTCAGGAAAAATTTGGCTTGAATATGTGAATCTACAGCCATACCATAATATGTAGAGAATGAAGTTTTCGTATATTGCATCAAGATCAATTCATAATGAACAAGATAAAGGTAGACAGGA
AGAGTTTTAAAAAACTTAATGAGACTATTGGCAAGTATCATGAACAAGAGTTATTCATCTTTGATGAATTATAGTTTAGCACCACACTTGAAAGTTAGACAATGGTAGTTAGAAAAGGACGTTAA
AACACAGGTTAAAGTAAAATTAGGTAGATAAAATTTTTATCTCCACAATGTGGTTAATTCTAGAGATGGAGAGAGCCC

>wLsig_tig1_301000

ACTAAATGCTGTGATTGCAGCAAAAAAAAAGCATAATATAGCAACCTTAAGAAAAATATATTTCTCTTTCAAGAACAGCACTAACTGCGTAGATAAAGCACCTAAAACTAGGAAGAGAAGAAAAA
TTGGTTACTTCCACCCTTCAACGCCATAGAAAAATTAGATTGAAATTAGAGTAACTTCGAATAAGTTGAAGTATGGATACAAGAGAATCCTAATGTTACTGTAAAAAATGAGAATAAGAATCTAG
GAAAAATTTATTTTGAATATCAGTAAATCTACAGTACACCTTAGAATATGCAAAAAATGAAATTTTCGTATATTACGCCAAAACCGGTTTATAATGAGCAAAATAGAGGTAGACAAGAAGAATTA
TAAGAACTTAATAAGAGACTATTTGGTAAGTATTCTAAACAAGAGCTATTATTCTTTAATAAACTGCGGTTTGATATACAATAGAAAATTGGACATCGGTAGTTTAAAAAGGGTGTTGTACCAACT
CTTATTATTAATGAGTCAAATTATTATTAGTGGGTCAAATAAGTAACATTACAGAGCTATTTAATTGCGGAGTGAGAAAGAAGAAAGGTAATAAATTTGCACAAGCACTCTTAAGCAAGGCAATT
GTATTGTAGGCTTTATTATGCAAA

>wLsig_tig1_906000

AAAAATACGAAAGTTGGATCGTATACAACCCAATCACAATTTCCCCGGACAAAACAGTTGCAGAAGCAATTTCATTGATGAAAGAGCATGATTATTCTTGTATTCCTGTAGTTGAGCAATGCAAG
TTAGTCGGAATTTTAACTAACAAAGATATAAGATTTATTGAARATCAGAACATGAGTACAAAAGTCTCTGAGGTAATGACAAAAGAGAAATTAGTTACAGTACGGGAGCAGGGGATAGACAGC
ACTTCAGCAATGAAACTCCTGCATGAAAACAGGATAGAGAAGCTTTTGGTCATAGATGAAAACTTCTGCTGCATAGGTCTGATCACAGCTAAAGACATTAAAGAATACAATAGATACCCCAATTC
ATGCAAAGATAGTAAAGGGCGGCTCAGAGTTGCCGCTGCAGTTGGCACTGGTAAAAAAGATGGTATAGAAAGATGTGAAGCTTTGATCAGAGAAGAGATTGACGTGATTATTGTAGATACTGC
TCACGGTCATTCCGAAAATGTTATTAATACTATTAAAGAAATAAAAACAATGTATCCAAGTACGCAGCTAGTTGCTGGAAATATTACAACAAAAGAAGCTGCTGAAGCRTTGATTAACGCTGGTG
CTGATGC

>wLsig_tig1_912000

AATTGGTGGGTTACAAGCTGCAATGGGGTATACCGGTAATAGAAATATAGAAGAGATGAAGAAAAATTGTAAGTTTACAGTTATGACTGCATCAGGATTAAGAGAAAGCCATGTTCATGACAT
AACTATCACACAGGAATCTCCAAATTATGTTTATCAAGTATCCAACAACTTGTCAAGTGATTCAGACATCTAATTGTTAATTGCCAGCTAATACAGTAAAAGTAAACAGGATAAAGCAGAAATTTT
TGCACTAATACAAAAGTCCCTGCTGTAGTTAAAAATGCATTATTCCTTAATTTGTCAAGTTATAATGCACGTTTGTTGATCTATGTAATATATATTATACCTGTACTTGCCTGTACATCCTACTACAT
CTACCCCGCATCATTATCAATTACCCACCTTCACCATCTTCTGCTCTATCTTCATTATAAGACCAATTTCTACTATATAAAGAACGGATAGACAATAATGAACAAGATAAA

>wLsig_tig1_926000

GATATAAAATTTCATTTTATGCATATAATGATGTATAGACTTGCTGACATTTAAGTTAAATTTTTCCTAAATTCTTATTCTCATTTCTTTAATGGTGATATTAGTTTTTTGTATTCATATTTCAACTTGT
TTAAATTGACTCTGATTCAATCTAATTATTTTCCGATGTTGAGGAGAAGCAAATAAATTTCTTTTCTTTCAAATTTTAGTACTTTATCTGTGTAGTTAGTACTGTTCGCAAAATGAGACATATTTTTAC
TACAGCTGTTATAGTGTATTTTTCTGCAATTACAGCATTTAGTTTTTTTGCAACATATACATTATCTCGCATTTTCTTCAGCATTTCTTGTGCTGATTTCATGACTTTTTCATTTAATAATTTTGATTTCA
ATATCATTTAAACCTCGTTATTTACTTGCTTCAGTTTGGCAAGAATTAGCATAAGATTTTCCCTGAAGAGATCTACATACAAAAGGCCTTTGAACCTAAAAAATTACCAAGATCTCACATATATAAT
GCCCATACTGTTAAAAATGCTTTTGTACTATTTTATCTATTTCTTCTAAATATTTATCACTAGAGTTACTTGTATTACAAGACCTATTTATAAAATATAAAATTCTTAAA

>wLsig_tig1_966000

GAATGAGAAAGAGAGGTAATATGTTTATATAAAGTGCTTCCAAGCAAAGCAATTATGTTATAAACTTTATTATGCAAAATGGTCTGTTTATATAGATAACCAAAGTCTCTCAATAGGATTGAATTT
AGGTAAGTATGTGATGGTAGATATATAGTCTTAATATTTTTAGGCACATTAAAATTTTTCTACTTATACTAACTAGTACAATTCATGACAAGAAATGCTCTTCATGTTTCTAAATACTGCAACATTTG
TTCAAGAAACATATTTATACAATCAGTGTTGACATTCGATGCAAATAAGATAGAATTCTTCCCATTTCTAAAATGGGCTGCATTGTAGAAGTAAAAACTTTATCTACCTAATTTTACTTTAATCTGC
GTTTTAACATCCTTTTTTAATCACCCGTATCCAACTTCTGAATGTGTGCTAAACCACAATTCATTAAAGAAAAGTAACTTTTTTTGAGATACTTACTAATAATCTCGTCAAGTTTTTTTAACTCCTCTT
GTCCACCTTTATCTTGTTCATTGCAAGTTAGTCTTGGTGTAATGTATAACATTTTTTGCATATTAAG

Supplementary files 3: Congruence between filarial nematodes and *Wolbachia* phylogenies. The topologies were inferred using Maximum Likelihood (ML) inference using IQTREE. Nodes are associated with bootstrap values based on 1,000 replicates; only bootstrap values superior to 70 are indicated. Analysis of filarial nematodes based on concatenation of 236 single-copy orthogroups representing a 71,128 amino-acid matrix. The best-fit model calculated using ModelFinder according to the BIC index was JTT+F+R4. Analysis of *Wolbachia* based on concatenation of 483 single-copy orthogroups representing a 143,945 amino-acid matrix. The best-fit model calculated using ModelFinder according to the BIC index was JTT+F+I+G4.

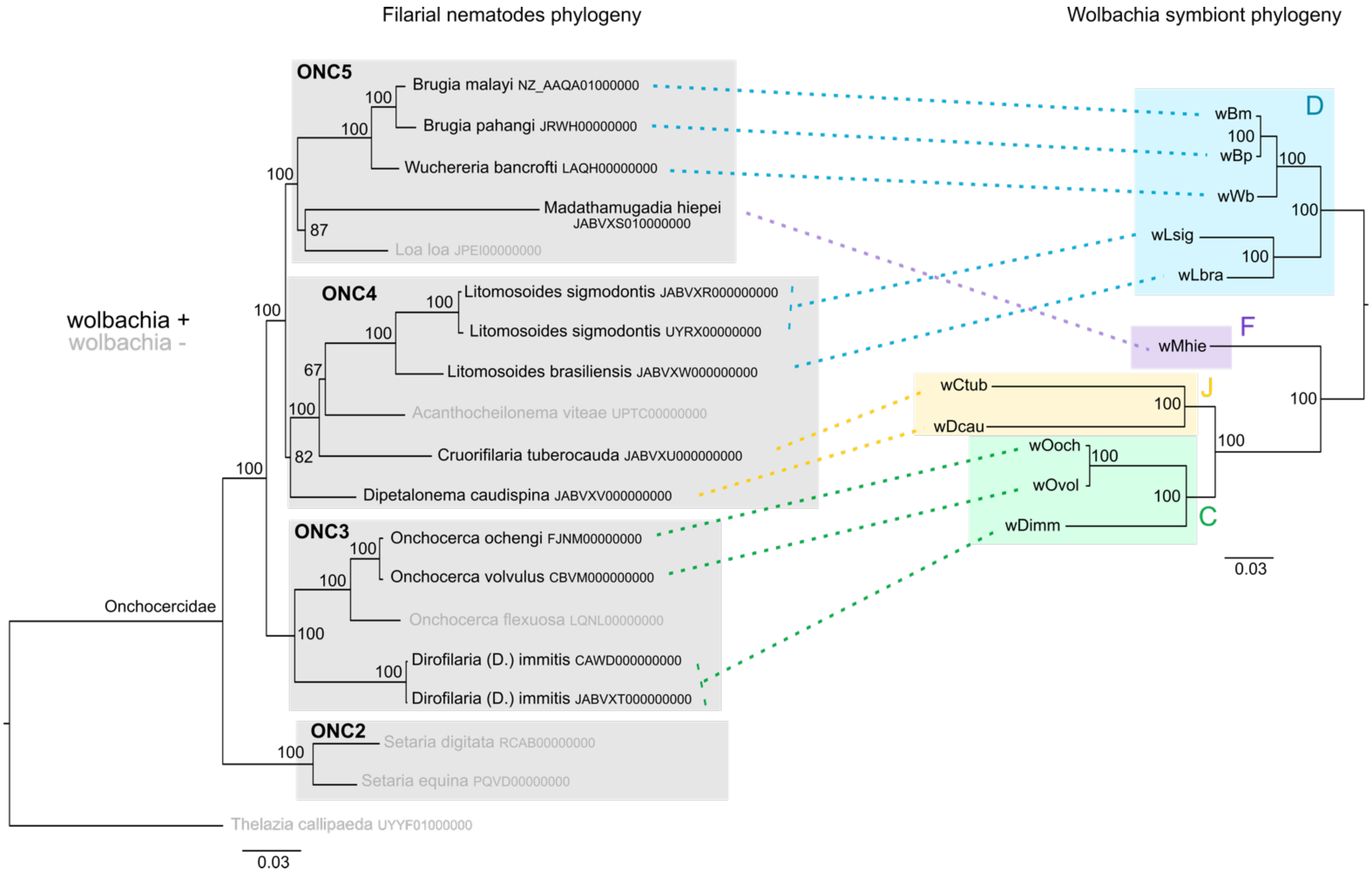

Filarial nematodes phylogeny
Wolbachia symbiont phylogeny
wolbachia +
wolbachia -
ONC5
Brugia malayi NZ_AAQA01000000
Brugia pahangi JRWH00000000
Wuchereria bancrofti LAQH00000000
Madathamugadia hiepei JABVXS010000000
Loa loa JPEI00000000
ONC4
Litomosoides sigmodontis JABVXR000000000
Litomosoides sigmodontis UYRX00000000
Litomosoides brasiliensis JABVXW000000000
Acanthocheilonema viteae UPTC00000000
Cruorifilaria tuberocauda JABVXU000000000
Dipetalonema caudispina JABVXV000000000
ONC3
Onchocerca ochengi FJNM00000000
Onchocerca volvulus CBVM000000000
Onchocerca flexuosa LQNL00000000
Dirofilaria (D.) immitis CAWD000000000
Dirofilaria (D.) immitis JABVXT000000000
ONC2
Setaria digitata RCAB00000000
Setaria equina PQVD00000000
Onchocercidae
Thelazia callipaeda UYYF01000000
0.03
D
wBm
wBp
wWb
wLsig
wLbra
F
wMhie
J
wCtub
wDcau
C
wOoch
wOvol
wDimm
0.03

Table S1. List of Wolbachia genomes used in the study with their strain name, NCBI accession number and detail of the molecular analyses.

The produced genome are indicated in blue. The genomes included in comparative analyses are indicated in bold.

Wolbachia	strain	complete genome	phylogenies 365 locus	160 locus
Wolbachia from *Aedes albopictus*	wAlbB	CP031221	yes	yes
Wolbachia from Atemnus politus	wApol K5	WQMQ00000000	no	yes
Wolbachia from *Drosophila simulans wAu*	wAu	LK055284	yes	yes
Wolbachia from *Plutella australiana wAus*	wAus	MRWX00000000	no	yes
Wolbachia* from *Brugia malayi	**wBm**	**NC_006833**	**yes**	**yes**
Wolbachia* from *Brugia pahangi	**wBp**	**CP050521**	**yes**	**yes**
Wolbachia from *Hypolimnas bolina*	wBol1b	CAOH00000000	no	yes
Wolbachia from *Bemisia tabaci*	wBtab	CP016430	yes	yes
Wolbachia* from *Carposina sasakii	**wCauA**	**CP041215**	**yes**	**yes**
Wolbachia* from *Ctenocephalides felis	**wCfeJ**	**CP051157**	**yes**	**yes**
Wolbachia* from *Ctenocephalides felis	**wCfeT**	**CP051156**	**yes**	**yes**
Wolbachia* from *Cimex lectularius	**wCle**	**AP013028**	**yes**	**yes**
Wolbachia from *Cylisticus convexus*	wcon	QPIP00000000	no	yes
Wolbachia* from *Cruorifilaria tuberocauda	**wCtub**	**CP046579**	**yes**	**yes**
Wolbachia from *Dactylopius coccus*	wDacB	LSYY00000000	no	yes
Wolbachia* from *Dipetalonema caudispina	**wDcau**	**CP046580**	**yes**	**yes**
Wolbachia* from *Dirofilaria (Dirofilaria) immitis	**wDimm**	**CP046578**	**yes**	**yes**
Wolbachia* from *Folsomia candida	**wFol Berlin**	**CP015510**	**yes**	**yes**
Wolbachia from *Glossina morsitans morsitans*	wGmm	AWUH00000000	no	yes
Wolbachia from *Drosophila simulans*	wHa	NC_021089	yes	yes
Wolbachia from *Drosophila incompta*	wIncCu	CP011148	yes	yes
Wolbachia* from *Litomosoides brasiliensis	**wLbra**	**WQMO00000000**	**no**	**yes**

Wolbachia from *Leptopilina clavipes*	wLcla	QJHA00000000	no	yes
Wolbachia* from *Litomosoides sigmondontis	**wLsig**	**CP046577**	**yes**	**yes**
Wolbachia* from *Nilaparvata lugens	**wLug**	**MUIY01000000**	**no**	**yes**
Wolbachia from *Drosophila mauritiana*	wMau	CP034334	yes	yes
Wolbachia from *Chrysomya megacephala*	wMeg	CP021120	yes	yes
Wolbachia* from *Drosophila melanogaster	**wMel**	**NC_002978**	**yes**	**yes**
Wolbachia* from *Madathamugadia hiepei	**wMhie**	**WQMP00000000**	**no**	**yes**
Wolbachia from *Nomada ferruginata*	wNfe	LYUY00000000	no	yes
Wolbachia* from *Nomada flava	**wNfla**	**LYUW00000000**	**no**	**yes**
Wolbachia from *Nomada leucophthalma*	wNleu	LYUV00000000	no	yes
Wolbachia from *Drosophila simulans*	wNo	NC_021084	yes	yes
Wolbachia from *Nomada panzeri*	wNpa	LYUX00000000	no	yes
Wolbachia* from *Onchocerca ochengi	**wOo**	**NC_018267**	**yes**	**yes**
Wolbachia* from *Onchocerca volvulus	**wOv Cameroon**	**HG810405**	**yes**	**yes**
Wolbachia* from *Culex quinquefasciatus	**wPip**	**NC_010981**	**yes**	**yes**
Wolbachia* from *Pratylenchus penetrans	**wPpe**	**MJMG01000000**	**no**	**yes**
Wolbachia from *Drosophila simulans wRi*	wRi	NC_012416	yes	yes
Wolbachia from *Drosphila santomea*	wsan	VCEH00000000	no	yes
Wolbachia* from *Laodelphax striatella	**wstri**	**MUIX00000000**	**no**	**yes**
Wolbachia from *Drosophila teissieri*	wTei	VCEG01000000	no	yes
Wolbachia* from *Trichogramma pretiosum	**wTpre**	**CM003641**	**yes**	**yes**
Wolbachia from *Muscidifurax uniraptor*	wUni	MUJL00000000	no	yes
Wolbachia from *Nasonia vitripennis*	wVitA	MUJM00000000	no	yes
Wolbachia from *Nasonia vitripennis*	wVitB	AERW00000000	no	yes
Wolbachia* from *Armadillidium vulgare	**wVulC**	**ALWU00000000**	**no**	**yes**
Wolbachia* from *Wuchereria bancrofti	**wWb**	**NJBR00000000**	**no**	**yes**
Wolbachia from *Drosophila yakuba*	wYak	VCEF00000000	no	yes

Table S2. Summary of number of genes assigned to 160 different KEGG pathways from Wolbachia genomes using KASS.

Difference of number genes:

<-5	-5	-4	-3	-2	-1	0	1	2	3	4	5

KO	KEGG pathways	Wolbachia																							
		wPpe	wFol	wCfeT	wPip	wLug	wstri	wTpre	wVulC	wMel	wCau	wNfla	wBm	wBp	wWb	wLs	wLbra	wCle	wMhie	wCfeJ	wOv	wOo	wDi	wDcau	wCtub
K00010	Glycolysis / Gluconeogenesis	11	11	11	11	11	11	11	11	11	11	11	11	11	10	11	11	11	10	11	11	11	11	11	11
K00020	Citrate cycle (TCA cycle)	16	17	16	17	15	17	17	17	16	17	16	17	17	16	17	17	17	17	17	17	17	17	17	17
K00030	Pentose phosphate pathway	6	6	6	6	6	6	6	6	6	6	6	6	6	6	6	6	6	4	6	6	6	6	6	6
K00040	Pentose and glucuronate interconversions	1	2	1	1	2	2	1	1	2	2	2	1	1	1	1	1	2	-	1	1	1	1	1	1
K00051	Fructose and mannose metabolism	4	5	5	5	5	5	4	5	4	4	4	4	4	5	4	4	4	4	5	4	4	4	4	4
K00053	Ascorbate and aldarate metabolism	-	1	-	-	1	1	-	-	1	1	1	-	-	-	-	-	1	-	-	-	-	-	-	-
K00520	Amino sugar and nucleotide sugar metabolism	5	7	6	6	7	7	5	6	6	6	6	5	4	6	5	5	6	6	6	3	4	5	5	5
K00620	Pyruvate metabolism	8	8	8	8	8	8	7	8	7	8	8	7	8	7	8	8	7	8	8	7	8	8	8	8
K00630	Glyoxylate and dicarboxylate metabolism	8	9	9	9	9	9	9	9	9	8	9	9	9	9	9	9	9	9	9	9	9	9	9	9
K00640	Propanoate metabolism	5	6	6	6	6	6	6	6	6	6	6	6	6	6	6	6	6	6	6	6	6	6	6	6
K00650	Butanoate metabolism	4	4	3	4	3	4	4	4	3	4	3	4	4	4	4	4	4	4	4	4	4	4	4	4
K00660	C5-Branched dibasic acid metabolism	2	2	2	2	2	2	2	2	2	2	2	2	2	2	2	2	2	2	2	2	2	2	2	2
K00562	Inositol phosphate metabolism	2	2	2	2	2	2	2	2	2	2	2	2	2	2	2	2	2	2	2	2	2	2	2	2
**K00190**	**Oxidative phosphorylation**	**35**	**37**	**35**	**37**	36	36	**37**	36	**38**	**39**	37	**37**	**36**	**36**	**37**	**37**	**36**	**36**	**38**	**36**	**36**	**36**	**36**	**36**
K00195	Photosynthesis	7	8	8	8	8	8	8	8	8	8	8	8	8	8	8	8	8	8	8	8	8	8	8	8
K00710	Carbon fixation in photosynthetic organism	10	10	10	10	10	10	9	10	9	10	10	9	10	10	10	10	9	8	10	9	10	10	10	10
K00720	Carbon fixation pathways in prokaryotes	11	12	11	12	11	12	12	12	11	12	11	12	12	12	12	12	12	12	12	12	12	12	12	12
K00680	Methane metabolism	6	6	6	6	6	6	6	6	6	6	6	6	6	6	6	6	6	5	6	6	6	6	6	6

K00910	Nitrogen metabolism	2	2	2	2	2	2	2	2	2	2	2	2	2	2	2	2	2	2	2	2	2	2	2	2
**K00061**	**Fatty acid biosynthesis**	6	8	8	8	8	8	8	8	8	8	8	8	8	7	8	8	8	8	8	8	8	8	8	8
K00561	Glycerolipid metabolism	3	4	4	4	4	4	3	4	4	4	4	3	3	3	3	3	3	3	3	3	3	3	3	3
**K00564**	**Glycerophospholipid metabolism**	9	11	9	10	10	10	9	11	10	10	10	8	9	9	8	7	9	9	10	8	9	10	9	9
**K00230**	**Purine metabolism**	22	21	21	22	21	21	22	22	22	22	21	22	22	22	21	20	21	20	21	20	21	21	21	21
**K00240**	**Pyrimidine metabolism**	16	17	17	17	17	17	17	17	17	17	17	17	17	17	17	17	17	16	17	17	17	17	17	17
K00250	Alanine, aspartate and glutamate metabolism	11	12	11	12	11	11	12	12	12	12	11	11	11	12	11	11	11	11	11	10	10	10	10	10
K00260	Glycine, serine and threonine metabolism	7	8	7	7	8	8	7	7	8	8	8	7	7	7	7	7	7	7	7	7	7	7	7	7
K00270	Cysteine and methionine metabolism	7	8	8	8	8	8	7	8	8	8	8	8	8	8	8	8	7	7	8	7	8	8	8	8
K00280	Valine, leucine and isoleucine degradation	2	3	3	3	3	3	3	3	3	3	3	3	3	3	3	3	3	3	3	3	3	3	3	3
K00300	Lysine biosynthesis	10	9	10	10	10	10	9	10	10	10	10	10	10	10	10	10	10	10	10	9	10	10	10	10
K00310	Lysine degradation	2	2	2	2	1	2	2	2	2	2	2	2	2	2	2	2	2	2	2	2	2	2	2	2
K00220	Arginine biosynthesis	6	6	6	6	6	6	6	6	6	6	6	6	6	6	6	6	6	6	6	5	6	6	6	6
K00330	Arginine and proline metabolism	2	2	2	2	2	2	2	2	2	2	2	2	2	2	2	2	2	2	2	2	2	1	1	1
K00350	Tyrosine metabolism	1	1	1	1	1	1	1	1	1	1	1	1	1	1	1	1	1	1	1	1	1	1	1	1
K00360	Phenylalanine metabolism	1	1	1	1	1	1	1	1	1	1	1	1	1	1	1	1	1	1	1	1	1	1	1	1
K00380	Tryptophan metabolism	2	2	2	2	1	2	2	2	2	2	2	2	2	2	2	2	2	2	2	2	2	2	1	2
K00400	Phenylalanine, tyrosine and tryptophan biosynthesis	1	1	1	1	1	1	1	1	1	1	1	1	1	1	1	1	1	1	1	1	1	1	1	1
K00410	beta-Alanine metabolism	1	1	2	1	1	1	1	1	1	1	1	1	1	1	1	1	1	1	1	1	1	1	1	1
K00430	Taurine and hypotaurine metabolism	1	1	1	1	1	1	1	1	1	1	1	1	1	1	1	1	1	1	1	1	1	1	1	1
K00450	Selenocompound metabolism	3	3	3	3	3	3	3	3	3	3	3	3	3	3	3	3	3	3	3	3	3	3	3	3
K00460	Cyanoamino acid metabolism	1	1	1	1	1	1	1	1	1	1	1	1	1	1	1	1	1	1	1	1	1	1	1	1
K00471	D-Glutamine and D-glutamate metabolism	3	3	3	3	3	3	3	3	3	3	3	3	3	3	3	3	3	3	3	3	3	3	3	3

K00473	D-Alanine metabolism	1	1	1	1	1	1	1	1	1	1	1	1	1	-	1	1	1	1	1	1	1	1	1	1
K00480	Glutathione metabolism	2	5	5	5	5	5	5	5	5	5	5	5	5	5	5	5	5	5	5	4	5	5	4	4
K00550	Peptidoglycan biosynthesis	10	11	13	11	11	11	11	12	12	12	12	11	12	10	11	12	12	12	13	11	11	11	11	11
**K00730**	**Thiamine metabolism**	2	3	9	4	4	4	2	3	**4**	**4**	**4**	2	2	2	2	2	**4**	2	**4**	2	2	2	2	2
**K00740**	**Riboflavin metabolism**	1	**6**	**6**	**6**	6	6	**6**	6	**6**	**6**	6	**6**	**6**	**6**	**6**	**5**	**6**	**5**	**5**	1	**4**	**5**	**5**	**5**
**K00750**	**Vitamin B6 metabolism**	**2**	**2**	-	**2**	2	2	**2**	2	**2**	**2**	2	-	-	**2**	-	**1**	**2**	**2**	**2**	**1**	**2**	**2**	**2**	**2**
K00760	Nicotinate and nicotinamide metabolism	2	2	2	2	2	2	2	2	2	2	2	2	2	2	2	2	2	2	2	2	2	2	2	2
K00770	Pantothenate and CoA biosynthesis	3	2	5	3	3	3	3	3	3	3	3	3	3	3	3	3	3	3	3	1	3	3	3	3
**K00780**	**Biotin metabolism**	**4**	**5**	9	**5**	10	10	**5**	10	**5**	**5**	10	**5**	**5**	**4**	**5**	**5**	10	**5**	**5**	**5**	**5**	**5**	**5**	**5**
K00785	Lipoic acid metabolism	2	2	2	2	2	2	2	2	2	2	2	2	2	2	2	2	2	2	2	2	2	2	2	2
**K00790**	**Folate biosynthesis**	**2**	**2**	**2**	**4**	5	5	**5**	5	**3**	**5**	5	**1**	**1**	**1**	**1**	-	**4**	**4**	**5**	**5**	**5**	**5**	**4**	**4**
K00670	One carbon pool by folate	7	6	7	7	7	7	7	7	7	7	7	6	7	6	6	5	7	7	5	7	7	7	7	7
**K00860**	**Porphyrin and chlorophyll metabolism**	**11**	**11**	**11**	**11**	11	11	**11**	11	**11**	**11**	11	**10**	**11**	**9**	**11**	**11**	**11**	**11**	**11**	**10**	**10**	**10**	**10**	**10**
K00130	Ubiquinone and other terpenoid-quinone biosynthesis	7	7	7	7	7	7	7	7	7	7	7	7	7	7	7	6	7	7	7	7	7	7	7	7
K00900	Terpenoid backbone biosynthesis	8	8	8	8	8	8	8	8	8	8	8	8	8	8	8	8	8	8	8	4	5	7	8	8
K00908	Zeatin biosynthesis	1	1	1	1	1	1	1	1	1	1	1	1	1	1	1	1	1	1	1	1	1	1	1	1
K01051	Biosynthesis of ansamycins	1	1	1	1	1	1	1	1	1	1	1	1	1	1	1	1	1	1	1	1	1	1	1	1
K00940	Phenylpropanoid biosynthesis	1	1	1	1	1	1	1	1	1	1	1	1	1	1	1	1	1	1	1	1	1	1	1	1
K00950	Isoquinoline alkaloid biosynthesis	1	1	1	1	1	1	1	1	1	1	1	1	1	1	1	1	1	1	1	1	1	1	1	1
K00960	Tropane, piperidine and pyridine alkaloid biosynthesis	1	1	1	1	1	1	1	1	1	1	1	1	1	1	1	1	1	1	1	1	1	1	1	1
K00261	Monobactam biosynthesis	4	4	4	4	4	4	4	4	4	4	4	4	4	4	4	4	4	4	4	4	4	4	4	4
K00521	Streptomycin biosynthesis	1	1	1	1	2	2	1	1	2	2	2	1	1	1	1	1	1	1	1	1	1	1	1	1
K00401	Novobiocin biosynthesis	1	1	1	1	1	1	1	1	1	1	1	1	1	1	1	1	1	1	1	1	1	1	1	1

K00333	Prodigiosin biosynthesis	3	3	3	3	3	3	3	3	3	3	3	3	3	3	3	3	3	3	3	3	3	3	3	3
K00998	Biosynthesis of secondary metabolites - other antibiotics	1	1	1	1	1	1	1	1	1	1	1	1	1	1	1	1	1	1	1	1	1	1	1	1
K00362	Benzoate degradation	1	1	1	-	1	1	-	1	1	-	1	1	1	1	-	-	1	1	1	1	1	1	1	1
K00627	Aminobenzoate degradation	1	1	1	1	1	1	1	1	1	1	1	1	1	1	1	1	1	1	1	1	1	1	1	1
K00624	Polycyclic aromatic hydrocarbon degradation	1	1	1	1	1	1	-	1	1	-	1	1	1	1	-	-	1	1	1	1	1	1	1	1
K00980	Metabolism of xenobiotics by cytochrome P450	1	1	1	1	1	1	1	1	1	1	1	1	1	1	1	1	1	1	1	1	1	1	1	-
K00982	Drug metabolism - cytochrome P450	1	1	1	1	1	1	1	1	1	1	1	1	1	1	1	1	1	1	1	1	1	1	1	-
K00983	Drug metabolism - other enzymes	4	5	5	5	5	5	5	5	5	5	5	5	5	5	5	5	5	5	5	5	5	5	4	4
K03020	RNA polymerase	3	3	4	3	3	3	3	3	3	3	3	3	3	3	3	3	3	3	3	3	3	3	3	3
K03010	Ribosome	51	53	51	53	53	53	52	52	52	53	52	52	53	49	51	52	51	48	51	52	50	52	52	52
K00970	Aminoacyl-tRNA biosynthesis	22	24	24	24	24	24	24	24	24	24	24	24	24	22	24	23	24	24	24	24	24	24	24	24
K03008	Ribosome biogenesis in eukaryotes	-	1	1	1	1	1	1	1	1	1	1	1	1	1	1	1	1	1	1	1	1	1	1	1
K03060	Protein export	15	15	14	15	14	14	15	15	15	15	14	15	15	15	15	15	15	15	14	15	15	15	15	15
K04141	Protein processing in endoplasmic reticulum	2	1	2	2	2	2	2	1	1	2	1	1	1	1	1	1	1	1	2	1	1	1	1	1
K04122	Sulfur relay system	2	2	3	2	2	2	2	2	2	2	2	2	2	2	2	2	2	2	2	2	2	2	2	2
K03018	RNA degradation	8	9	10	8	9	9	9	9	9	9	9	9	9	8	9	9	9	9	10	9	9	9	9	9
K03030	DNA replication	13	13	13	13	13	13	13	13	13	13	13	13	13	12	13	13	13	13	13	13	13	13	13	13
K03410	Base excision repair	8	7	8	8	8	7	8	8	8	8	8	8	8	8	8	8	8	8	8	8	8	8	7	7
K03420	Nucleotide excision repair	3	6	6	6	6	6	6	6	6	6	6	6	6	5	6	6	6	6	6	6	6	6	5	5
K03430	Mismatch repair	12	12	12	12	12	12	12	12	12	12	12	12	12	12	12	12	12	12	12	12	12	12	11	12
**K03440**	**Homologous recombination**	**17**	**17**	**18**	**17**	18	18	**18**	18	**18**	**18**	18	**18**	**18**	**18**	**13**	**11**	**18**	**18**	**18**	**10**	**11**	**11**	**8**	**17**
**K02010**	**ABC transporters**	**20**	**20**	**20**	**20**	20	20	**20**	20	**19**	**19**	19	**20**	**20**	**20**	**17**	**18**	**20**	**20**	**20**	**15**	**15**	**17**	**12**	**12**
**K03070**	**Bacterial secretion system**	**22**	**23**	**22**	**23**	22	22	**23**	23	**23**	**23**	22	**22**	**22**	**22**	**23**	**23**	**23**	**23**	**22**	**22**	**22**	**22**	**22**	**22**

K02020	Two-component system	10	12	14	12	12	12	12	12	14	14	14	12	14	10	12	12	10	12	**16**	10	12	11	11	11
K04013	MAPK signaling pathway - fly	1	1	1	1	1	1	1	1	1	1	1	1	1	1	1	1	1	1	1	1	1	1	1	1
K04016	MAPK signaling pathway - plant	1	1	1	1	1	1	1	1	1	1	1	1	1	1	1	1	1	1	1	1	1	1	1	1
K04066	HIF-1 signaling pathway	5	5	5	5	5	5	5	5	5	5	5	5	5	5	5	5	5	5	5	5	5	5	5	5
K04068	FoxO signaling pathway	1	1	1	1	1	1	1	1	1	1	1	1	1	1	1	1	1	1	1	1	1	1	1	1
K04070	Phosphatidylinositol signaling system	2	3	3	3	3	3	2	3	3	3	3	2	2	2	2	2	2	2	2	2	2	2	2	2
K04151	PI3K-Akt signaling pathway	1	1	1	1	1	1	1	1	1	1	1	1	1	1	1	1	1	1	1	1	1	1	1	1
K04152	AMPK signaling pathway	1	1	1	1	1	1	1	1	1	1	1	1	1	1	1	1	1	1	1	1	1	1	1	1
K04146	Peroxisome	3	3	3	3	3	3	3	3	3	3	3	3	3	3	3	3	3	3	3	3	3	3	3	3
**K04112**	**Cell cycle - Caulobacter**	**11**	**14**	16	**13**	13	13	**13**	13	**14**	**14**	14	**13**	**15**	**13**	**12**	**13**	**12**	**13**	16	**10**	**12**	**11**	**8**	**9**
K04210	Apoptosis	-	1	1	1	1	1	1	1	1	1	1	1	1	1	1	1	1	1	1	1	1	1	1	1
K04214	Apoptosis - fly	2	2	2	2	2	2	2	2	2	2	2	2	2	2	2	2	2	2	2	2	2	2	2	2
K04215	Apoptosis - multiple species	-	1	1	1	1	1	1	1	1	1	1	1	1	1	1	1	1	1	1	1	1	1	1	1
K04217	Necroptosis	2	2	2	2	2	2	2	2	2	2	2	2	2	2	2	2	2	2	2	2	2	2	2	2
K04115	p53 signaling pathway	-	1	1	1	1	1	1	1	1	1	1	1	1	1	1	1	1	1	1	1	1	1	1	1
K02024	Quorum sensing	9	11	11	11	10	10	11	11	11	11	10	11	11	11	11	10	11	10	10	10	10	11	10	10
K02026	Biofilm formation - Escherichia coli	1	1	1	1	1	1	1	1	1	1	1	1	1	1	1	1	-	1	1	1	1	1	1	1
K04621	NOD-like receptor signaling pathway	2	2	2	2	1	1	2	1	2	2	1	2	2	2	2	2	2	2	2	2	2	2	2	2
K04612	Antigen processing and presentation	1	1	1	1	2	2	1	2	1	1	2	1	1	1	1	1	1	1	1	1	1	1	1	1
K04659	Th17 cell differentiation	1	1	1	1	1	1	1	1	1	1	1	1	1	1	1	1	1	1	1	1	1	1	1	1
K04657	IL-17 signaling pathway	1	1	1	1	1	1	1	1	1	1	1	1	1	1	1	1	2	1	1	1	1	1	1	1
K04922	Glucagon signaling pathway	2	2	2	2	1	1	2	1	2	2	1	2	2	2	2	2	2	2	2	2	2	2	2	2
K03320	PPAR signaling pathway	1	1	1	1	2	2	-	2	-	1	2	-	1	1	1	1	-	1	1	-	1	1	1	1

K04915	Estrogen signaling pathway	1	1	1	1	1	1	1	1	1	1	1	1	1	1	1	1	1	1	1	1	1	1	1	1
K04914	Progesterone-mediated oocyte maturation	1	1	1	1	1	1	1	1	1	1	1	1	1	1	1	1	1	1	1	-	1	1	1	1
K04260	Cardiac muscle contraction	3	3	3	3	1	1	3	1	3	3	1	3	3	2	3	3	3	3	3	3	3	3	3	3
K04724	Glutamatergic synapse	1	1	1	1	3	3	1	3	1	1	3	1	1	1	1	1	1	1	1	1	1	1	1	1
K04727	GABAergic synapse	1	1	1	1	1	1	1	1	1	1	1	1	1	1	1	1	1	1	1	1	1	1	1	1
K04211	Longevity regulating pathway	1	1	1	1	1	1	1	1	1	1	1	1	1	1	1	1	1	1	1	1	1	1	1	1
K04212	Longevity regulating pathway - worm	3	5	5	5	1	1	5	1	5	5	1	5	5	5	5	5	5	5	5	5	5	5	4	4
K04213	Longevity regulating pathway - multiple species	2	1	2	2	5	5	2	5	2	2	5	2	2	2	2	2	2	2	2	2	2	2	2	2
K04714	Thermogenesis	6	6	6	6	2	2	6	2	6	6	2	6	6	5	6	6	6	6	6	6	6	6	6	6
K04626	Plant-pathogen interaction	2	2	3	2	6	6	2	6	2	2	6	2	3	2	3	2	2	2	3	2	2	3	2	2
K05200	Pathways in cancer	2	4	4	4	3	3	4	3	4	4	3	4	4	4	4	4	4	4	4	4	4	4	3	3
K05205	Proteoglycans in cancer	-	1	1	1	4	4	1	4	1	1	4	1	1	1	1	1	1	1	1	1	1	1	1	1
K05204	Chemical carcinogenesis	-	1	1	1	1	1	1	1	1	1	1	1	1	1	1	1	1	1	1	1	1	1	-	-
K05230	Central carbon metabolism in cancer	2	2	2	2	1	1	2	1	2	2	1	2	2	2	2	2	2	2	2	2	2	2	2	2
K05231	Choline metabolism in cancer	-	1	1	1	2	2	-	2	1	1	2	-	-	-	-	-	-	-	-	-	-	-	-	-
K05210	Colorectal cancer	-	1	1	1	1	1	1	1	1	1	1	1	1	1	1	1	1	1	1	1	1	1	1	1
K05225	Hepatocellular carcinoma	-	1	1	1	1	1	1	1	1	1	1	1	1	1	1	1	1	1	1	1	1	1	-	-
K05211	Renal cell carcinoma	1	1	1	1	1	1	1	1	1	1	1	1	1	1	1	1	1	1	1	1	1	1	1	1
K05215	Prostate cancer	1	1	1	1	1	1	1	1	1	1	1	1	1	1	1	1	1	1	1	1	1	1	1	1
K05222	Small cell lung cancer	-	1	1	1	1	1	1	1	1	1	1	1	1	1	1	1	1	1	1	1	1	1	1	1
K05010	Alzheimer disease	4	5	5	5	5	5	5	5	5	5	5	5	5	4	5	5	5	5	5	5	5	5	5	5
K05012	Parkinson disease	3	4	4	4	4	4	4	4	4	4	4	4	4	3	4	4	4	4	4	4	4	4	4	4
K05014	Amyotrophic lateral sclerosis (ALS)	-	1	1	1	1	1	1	1	1	1	1	1	1	1	1	1	1	1	1	1	1	1	1	1

K05016	Huntington disease	4	5	5	5	5	5	5	5	5	5	5	5	5	4	5	5	5	5	5	5	5	5	5	5
K05418	Fluid shear stress and atherosclerosis	2	3	3	3	3	3	3	3	3	3	3	3	3	3	3	3	3	3	3	3	3	3	2	2
K05416	Viral myocarditis	-	1	1	1	1	1	1	1	1	1	1	1	1	1	1	1	1	1	1	1	1	1	1	1
K04940	Type I diabetes mellitus	-	1	1	1	1	1	1	1	1	1	1	1	1	1	1	1	1	1	1	1	1	1	1	1
K04932	Non-alcoholic fatty liver disease (NAFLD)	3	4	4	4	4	4	4	4	4	4	4	4	4	3	4	4	4	4	4	4	4	4	4	4
K04931	Insulin resistance	1	1	1	1	1	1	1	1	1	1	1	1	1	1	1	1	1	1	1	-	-	1	1	1
K04934	Cushing syndrome	1	1	1	1	1	1	1	1	1	1	1	1	1	1	1	1	1	1	1	1	1	1	1	1
K05120	Epithelial cell signaling in Helicobacterpylori infection	1	1	1	1	1	1	1	1	1	1	1	1	1	1	1	1	1	1	1	1	1	1	1	1
K05130	Pathogenic Escherichia coli infection	1	2	2	2	2	2	2	2	2	2	2	2	2	2	2	2	2	2	2	2	2	2	2	2
K05133	Pertussis	1	1	1	1	1	1	1	1	1	1	1	1	1	1	1	1	1	1	1	1	1	1	2	1
K05134	Legionellosis	2	4	4	4	4	4	4	4	4	4	4	4	4	4	4	4	4	4	4	4	4	4	4	4
K05152	Tuberculosis	2	4	4	4	4	4	4	4	4	4	4	4	4	4	4	4	4	4	4	4	4	4	4	4
K05170	Human immunodeficiency virus 1 infection	-	1	1	1	1	1	1	1	1	1	1	1	1	1	1	1	1	1	1	1	1	1	1	1
K05162	Measles	-	1	1	1	1	1	1	1	1	1	1	1	1	1	1	1	1	1	1	1	1	1	1	1
K05164	Influenza A	-	1	1	1	1	1	1	1	1	1	1	1	1	1	1	1	1	1	1	1	1	1	1	1
K05161	Hepatitis B	-	1	1	1	1	1	1	1	1	1	1	1	1	1	1	1	1	1	1	1	1	1	1	1
K05160	Hepatitis C	-	1	1	1	1	1	1	1	1	1	1	1	1	1	1	1	1	1	1	1	1	1	1	1
K05168	Herpes simplex virus 1 infection	-	1	1	1	1	1	1	1	1	1	1	1	1	1	1	1	1	1	1	1	1	1	1	1
K05163	Human cytomegalovirus infection	-	1	1	1	1	1	1	1	1	1	1	1	1	1	1	1	1	1	1	1	1	1	1	1
K05167	Kaposi sarcoma-associated herpesvirus infection	-	1	1	1	1	1	1	1	1	1	1	1	1	1	1	1	1	1	1	1	1	1	1	1
K05169	Epstein-Barr virus infection	-	1	1	1	1	1	1	1	1	1	1	1	1	1	1	1	1	1	1	1	1	1	1	1
K05145	Toxoplasmosis	-	1	1	1	1	1	1	1	1	1	1	1	1	1	1	1	1	1	1	1	1	1	1	1
K01501	beta-Lactam resistance	1	3	3	2	2	2	2	3	2	3	3	2	2	2	2	3	3	3	3	2	2	2	2	2

K01502	Vancomycin resistance	4	4	4	4	4	4	4	4	4	4	4	4	4	3	4	4	4	4	4	4	4	4	4	4
K01503	Cationic antimicrobial peptide (CAMP) resistance	2	2	2	2	2	2	2	2	2	2	2	2	2	1	2	2	2	2	2	2	2	2	2	2
K01524	Platinum drug resistance	-	2	2	2	2	2	2	2	2	2	2	2	2	2	2	2	2	2	2	2	2	2	1	1
K01523	Antifolate resistance	2	2	2	3	3	3	3	3	3	3	3	2	2	2	2	1	3	3	3	3	3	3	3	3

Table S3. Summary of number of genes assigned to 165 different KEGG pathways from Wolbachia genomes using KASS.

		Wolbachia											
	**K0**	**wBm**	**wBp**	**wCauA**	**wCfeJ**	**wCfeT**	**wCle**	**wCtub**	**wDcau**	**wDi**	**wFol**	**wLbra**	**wLsig**
**K00730 Thiamine metabolism**													
iscS, NFS1	**K04487**	WP_011256223.1	WP_011256223.1	QDH18146.1	wCfeJ_00074	wCfeT_00368	WP_041045648.1	wCtub_t1_00121	wDcau_t1_00195	wDi2_02130	WP_110409687.1	LOGDKPFN_00194	wLs20_03220
		WP_041571528.1	WP_167896281.1	QDH18693.1	wCfeJ_00245	wCfeT_01262	WP_041045861.1	wCtub_t1_00161	wDcau_t1_00229	wDi2_02520	WP_110410487.1	LOGDKPFN_00887	wLs20_06930
tenA	**K03707**			QDH18495.1	wCfeJ_00019	wCfeT_00958	WP_041045607.1				WP_110409931.1		
					wCfeJ_00020	wCfeT_00959					WP_110409932.1		
adk, AK	**K00939**	WP_011256520.1	WP_167895918.1	QDH18726.1	wCfeJ_01020	wCfeT_00162	WP_041045236.1	wCtub_t1_00434	wDcau_t1_00482	wDi2_05540	WP_110409453.1	LOGDKPFN_00587	wLs20_07400
total		3	3	4	5	5	4	3	3	3	5	3	3
**K00740 Riboflavin metabolism**													
ribA, RIB1	**K01497**	WP_011256477.1	WP_167895986.1	QDH18591.1		wCfeT_00027	WP_041046499.1			wDi2_02950	WP_110409421.1		wLs20_00530
ribD	**K11752**	WP_011256228.1	WP_167895712.1	QDH18687.1	wCfeJ_00068	wCfeT_00693	WP_041045850.1	wCtub_t1_00155	wDcau_t1_00223	wDi2_02470	WP_110409590.1	LOGDKPFN_00881	wLs20_06990
ribB, RIB3	**K02858**	WP_011256511.1	WP_167895926.1	QDH18735.1	wCfeJ_00914	wCfeT_00171	WP_041045526.1	wCtub_t1_00299	wDcau_t1_00418	wDi2_04020	WP_110409444.1	LOGDKPFN_00578	wLs20_07310
ribH, RIB4	**K00794**	WP_011256390.1	WP_011256390.1	QDH18637.1	wCfeJ_00664	wCfeT_00014	WP_041045945.1	wCtub_t1_00207	wDcau_t1_00276	wDi2_03070	WP_110410142.1	LOGDKPFN_00425	wLs20_00650
ribE, RIB5	**K00793**	WP_011256285.1	WP_167896219.1	QDH18442.1	wCfeJ_00607	wCfeT_00072	WP_041046143.1	wCtub_t1_00472	wDcau_t1_00153		WP_110409930.1	LOGDKPFN_00983	wLs20_07900
ribF	**K11753**	WP_011256614.1	WP_011256614.1	QDH18875.1	wCfeJ_00117	wCfeT_00090	WP_041046413.1	wCtub_t1_00641	wDcau_t1_00020	wDi2_07790	WP_110410546.1	LOGDKPFN_01000	wLs20_03330
total		6	6	6	5	6	6	5	5	5	6	5	6
**K00780 Biotin metabolism**													
bioC	**K02169**						WP_041046000.1						
fabF, OXSM, CEM1	**K09458**	WP_011256936.1	WP_011256936.1	QDH18260.1	wCfeJ_00563	wCfeT_00486	WP_041046165.1	wCtub_t1_00663	wDcau_t1_00033	wDi2_07550	WP_110409604.1	LOGDKPFN_00107	wLs20_03840
fabG, OAR1	**K00059**	WP_011256509.1	WP_011256509.1	QDH18738.1	wCfeJ_00911	wCfeT_01005	WP_041045521.1	wCtub_t1_00301	wDcau_t1_00415	wDi2_04050	WP_110409528.1	LOGDKPFN_00575	wLs20_07280
fabZ	**K02372**	WP_011256254.1	WP_167896200.1	QDH19233.1	wCfeJ_00191	wCfeT_00885	WP_041046228.1	wCtub_t1_00569	wDcau_t1_00061	wDi2_00570	WP_110409511.1	LOGDKPFN_00921	wLs20_08750
fabI	**K00208**	WP_011256668.1	WP_167895823.1	QDH18549.1	wCfeJ_00446	wCfeT_01287	WP_041046022.1	wCtub_t1_00491	wDcau_t1_00143	wDi2_01460	WP_110410193.1	LOGDKPFN_00909	wLs20_03390
bioF	**K00652**					wCfeT_01265	WP_052463260.1						
bioA	**K00833**					wCfeT_01269	WP_041045996.1						
bioD	**K01935**					wCfeT_01268	WP_041045998.1						
bioB	**K01012**					wCfeT_01264	WP_041046003.1						
birA	**K03524**	WP_011256818.1	WP_167896086.1	QDH18166.1	wCfeJ_00395	wCfeT_00808	WP_041045174.1	wCtub_t1_00145	wDcau_t1_00214	wDi2_02370	WP_110409976.1	LOGDKPFN_00078	wLs20_04030
total		5	5	5	5	9	10	5	5	5	5	5	5
**K00061 Fatty acid biosynthesis**													

fabD	**K00645**	WP_011256600.1	WP_167895869.1	QDH19181.1	wCfeJ_00103	wCfeT_00563	WP_041046382.1	wCtub_t1_00606	wDcau_t1_00665	wDi2_00220	WP_112477189.1	LOGDKPFN_00857	wLs20_08430
fabH	**K00648**	WP_011256812.1	WP_167896082.1	QDH18153.1	wCfeJ_00525	wCfeT_01176	WP_041045411.1	wCtub_t1_00150	wDcau_t1_00219	wDi2_02420	WP_110409948.1	LOGDKPFN_00071	wLs20_04110
fabF, OXSM, CEM1	**K09458**	WP_011256936.1	WP_011256936.1	QDH18260.1	wCfeJ_00563	wCfeT_00486	WP_041046165.1	wCtub_t1_00663	wDcau_t1_00033	wDi2_07550	WP_110409604.1	LOGDKPFN_00107	wLs20_03840
fabG, OAR1	**K00059**	WP_011256509.1	WP_011256509.1	QDH18738.1	wCfeJ_00911	wCfeT_01005	WP_041045521.1	wCtub_t1_00301	wDcau_t1_00415	wDi2_04050	WP_110409528.1	LOGDKPFN_00575	wLs20_07280
fabZ	**K02372**	WP_011256254.1	WP_167896200.1	QDH19233.1	wCfeJ_00191	wCfeT_00885	WP_041046228.1	wCtub_t1_00569	wDcau_t1_00061	wDi2_00570	WP_110409511.1	LOGDKPFN_00921	wLs20_08750
fabI	**K00208**	WP_011256668.1	WP_167895823.1	QDH18549.1	wCfeJ_00446	wCfeT_01287	WP_041046022.1	wCtub_t1_00491	wDcau_t1_00143	wDi2_01460	WP_110410193.1	LOGDKPFN_00909	wLs20_03390
fabK	**K02371**	WP_011256950.1	WP_167896189.1	QDH18242.1	wCfeJ_00136	wCfeT_00737	WP_041045454.1	wCtub_t1_00517	wDcau_t1_00115	wDi2_01180	WP_110409562.1	LOGDKPFN_00705	wLs20_04850
total		7	7	7	7	7	7	7	7	7	7	7	7
**K00790 Folate biosynthesis**													
ribA, RIB1	**K01497**	WP_011256477.1	WP_167895986.1	QDH18591.1		wCfeT_00027	WP_041046499.1			wDi2_02950	WP_110409421.1		wLs20_00530
folB	**K01633**			QDH19194.1	wCfeJ_00748		WP_041046394.1	wCtub_t1_00386	wDcau_t1_00294	wDi2_04980			
folKP	**K13941**			QDH19193.1	wCfeJ_00747		WP_041046396.1	wCtub_t1_00385	wDcau_t1_00295	wDi2_04970			
folC	**K11754**			QDH19257.1	wCfeJ_00338	wCfeT_01555		wCtub_t1_00180	wDcau_t1_00249	wDi2_02730	WP_110410545.1		
DHFR, folA	**K00287**			QDH19192.1	wCfeJ_00746		WP_041046398.1	wCtub_t1_00384	wDcau_t1_00296	wDi2_04960			
total		1	1	5	4	2	4	4	4	5	2	0	1
**K03070 Bacterial secretion system**													
tolC	**K12340**	WP_011256773.1	WP_167895745.1	QDH18501.1	wCfeJ_00712	wCfeT_01435	WP_041045017.1	wCtub_t1_00050	wDcau_t1_00526	wDi2_05990	WP_110409432.1	LOGDKPFN_00628	wLs20_08250
gspD	**K02453**			QDH19118.1	wCfeJ_00402	wCfeT_00700	WP_041046248.1				WP_110410356.1	LOGDKPFN_00973	wLs20_02520
secD	**K03072**	WP_041571521.1	WP_167896268.1	QDH18548.1	wCfeJ_00752	wCfeT_00908	WP_041044998.1	wCtub_t1_00363	wDcau_t1_00312	wDi2_04740	WP_110409614.1	LOGDKPFN_00464	wLs20_01280
secF	**K03074**	WP_011256387.1	WP_167895973.1	QDH18640.1	wCfeJ_00661	wCfeT_00833	WP_041045951.1	wCtub_t1_00210	wDcau_t1_00278	wDi2_03090	WP_110409898.1	LOGDKPFN_00422	wLs20_00630
secE	**K03073**	WP_041571587.1	WP_041571587.1	QDH18579.1			WP_041046716.1	wCtub_t1_00091	wDcau_t1_00180	wDi2_01950	WP_110409549.1	LOGDKPFN_00234	wLs20_02950
SecG	**K03075**	WP_041571540.1	WP_167895943.1	QDH19088.1	wCfeJ_00877	wCfeT_01419	WP_041045280.1	wCtub_t1_00277	wDcau_t1_00348	wDi2_03790	WP_110410302.1	LOGDKPFN_00164	wLs20_01850
SecY	**K03076**	WP_011256521.1	WP_011256521.1	QDH18725.1	wCfeJ_01021	wCfeT_00161	WP_041046552.1	wCtub_t1_00435	wDcau_t1_00483	wDi2_05550	WP_110409454.1	LOGDKPFN_00588	wLs20_07410
YajC	**K03210**	WP_011256423.1	WP_167896027.1	QDH19026.1	wCfeJ_00399	wCfeT_00921	WP_041045884.1	wCtub_t1_00588	wDcau_t1_00651	wDi2_00390	WP_110409595.1	LOGDKPFN_00101	wLs20_03890
yidC, spoIIIJ, OXA1, ccfA	**K03217**	WP_011256389.1	WP_167895972.1	QDH18638.1	wCfeJ_00663	wCfeT_00015	WP_041045947.1	wCtub_t1_00208	wDcau_t1_00277	wDi2_03080	WP_110410143.1	LOGDKPFN_00424	wLs20_00640
SecA	**K03070**	WP_011256465.1	WP_167895993.1	QDH19019.1	wCfeJ_00621	wCfeT_00190	WP_041046459.1	wCtub_t1_00511	wDcau_t1_00121	wDi2_01300	WP_110409731.1	LOGDKPFN_00616	wLs20_07710
ftsY	**K03110**	WP_041571510.1	WP_041571510.1	QDH19153.1	wCfeJ_00532	wCfeT_00432	WP_041045901.1	wCtub_t1_00528	wDcau_t1_00104	wDi2_01050	WP_110409517.1	LOGDKPFN_00693	wLs20_06050
SecB	**K03071**	WP_011256739.1	WP_011256739.1	QDH18533.1	wCfeJ_00695	wCfeT_01189	WP_041045575.1	wCtub_t1_00244	wDcau_t1_00380	wDi2_03450	WP_110409254.1	LOGDKPFN_00351	wLs20_02070
ffh	**K03106**	WP_011256251.1	WP_167895727.1	QDH19237.1	wCfeJ_00187	wCfeT_01104	WP_041045807.1	wCtub_t1_00566	wDcau_t1_00064	wDi2_00600	WP_110409582.1	LOGDKPFN_00831	wLs20_06840
TatA	**K03116**	WP_006013840.1	WP_006013840.1	QDH18480.1	wCfeJ_00646	wCfeT_00851	WP_006013840.1	wCtub_t1_00026	wDcau_t1_00567	wDi2_06470	WP_110410593.1	LOGDKPFN_00543	wLs20_00320
TatC	**K03118**	WP_011256496.1	WP_011256496.1	QDH19075.1	wCfeJ_00903	wCfeT_00608	WP_041045428.1	wCtub_t1_00309	wDcau_t1_00408	wDi2_04130	WP_110410299.1	LOGDKPFN_00495	wLs20_01370

virB3, lvhB3	**K03198**	WP_011256995.1	WP_011256995.1	QDH19171.1	wCfeJ_00239	wCfeT_01125	WP_041046206.1	wCtub_t1_00729	wDcau_t1_00601	wDi2_06860	WP_110409799.1	LOGDKPFN_00117	wLs20_03710
virB9, lvhB9	**K03204**	WP_011256789.1	WP_041571550.1	QDH18223.1	wCfeJ_00360	wCfeT_00025	WP_041044918.1	wCtub_t1_00198	wDcau_t1_00149	wDi2_01610	WP_110409834.1	LOGDKPFN_00018	wLs20_00550
		WP_041571550.1	WP_167895737.1	QDH18589.1	wCfeJ_00675	wCfeT_00193	WP_041045008.1	wCtub_t1_00477	wDcau_t1_00267	wDi2_02970	WP_110410531.1	LOGDKPFN_00415	wLs20_04540
virB6, lvhB6	**K03201**	WP_011256991.1	WP_167896235.1	QDH19166.1	wCfeJ_00234	wCfeT_01127	WP_041046210.1	wCtub_t1_00723	wDcau_t1_00603	wDi2_06880	WP_110409796.1	LOGDKPFN_00119	wLs20_03660
		WP_041571522.1	WP_167896236.1	QDH19167.1	wCfeJ_00235	wCfeT_01128	WP_041046212.1	wCtub_t1_00725	wDcau_t1_00604	wDi2_06890	WP_110409797.1	LOGDKPFN_00120	wLs20_03670
		WP_050707683.1	WP_167896237.1	QDH19168.1	wCfeJ_00236	wCfeT_01129	WP_041046214.1	wCtub_t1_00726	wDcau_t1_00605	wDi2_06900	WP_110410558.1	LOGDKPFN_00121	wLs20_03680
			WP_167896238.1	QDH19169.1	wCfeJ_00237	wCfeT_01130	WP_041046216.1	wCtub_t1_00727	wDcau_t1_00606	wDi2_06910	WP_112477239.1	LOGDKPFN_00122	wLs20_03690
virB8	**K03203**	WP_011256478.1	WP_167895985.1	QDH18590.1	wCfeJ_00271	wCfeT_00026	WP_041044916.1	wCtub_t1_00197	wDcau_t1_00047	wDi2_00420	WP_110409420.1	LOGDKPFN_00247	wLs20_00540
		WP_011256839.1	WP_167896107.1	QDH19127.1	wCfeJ_00676	wCfeT_00505	WP_041046014.1	wCtub_t1_00585	wDcau_t1_00266	wDi2_02960	WP_110410455.1	LOGDKPFN_00414	wLs20_02830
virB10	**K03195**	WP_011256480.1	WP_167895984.1	QDH18588.1	wCfeJ_00674	wCfeT_00024	WP_041044920.1	wCtub_t1_00199	wDcau_t1_00268	wDi2_02980	WP_110409419.1	LOGDKPFN_00416	wLs20_00560
virB4	**K03199**	WP_011256947.1	WP_167896186.1	QDH18244.1	wCfeJ_00238	wCfeT_01072	WP_041045460.1	wCtub_t1_00519	wDcau_t1_00113	wDi2_01160	WP_110409723.1	LOGDKPFN_00118	wLs20_03700
		WP_011256994.1	WP_167896239.1	QDH19170.1	wCfeJ_00588	wCfeT_01126	WP_041046208.1	wCtub_t1_00728	wDcau_t1_00602	wDi2_06870	WP_110409798.1	LOGDKPFN_00942	wLs20_04810
virB11	**K03196**	WP_011256481.1	WP_167895983.1	QDH18587.1	wCfeJ_00673	wCfeT_00023	WP_041044922.1	wCtub_t1_00200	wDcau_t1_00269	wDi2_02990	WP_110409418.1	LOGDKPFN_00417	wLs20_00570
virD4	**K03205**	WP_011256482.1	WP_167895982.1	QDH18586.1	wCfeJ_00672	wCfeT_00022	WP_041044924.1	wCtub_t1_00201	wDcau_t1_00270	wDi2_03000	WP_110409417.1	LOGDKPFN_00418	wLs20_00580
total		27	28	29	28	28	29	28	28	28	29	29	29
**K00190 Oxidative phosphorylation**													
**NADH Dehydrogenase**													
nuoA	**K00330**	WP_011256440.1	WP_167896015.1	QDH18203.1	wCfeJ_00545	wCfeT_00065	WP_041045630.1	wCtub_t1_00171	wDcau_t1_00238	wDi2_02640	WP_110410184.1	LOGDKPFN_00338	wLs20_06200
nuoB	**K00331**	WP_011256441.1	WP_167896014.1	QDH18204.1	wCfeJ_00544	wCfeT_00066	WP_041045628.1	wCtub_t1_00172	wDcau_t1_00239	wDi2_02650	WP_110410183.1	LOGDKPFN_00337	wLs20_06210
nuoC	**K00332**	WP_011256442.1	WP_167896013.1	QDH18205.1	wCfeJ_00543	wCfeT_00067	WP_041045626.1	wCtub_t1_00173	wDcau_t1_00240	wDi2_02660	WP_110410182.1	LOGDKPFN_00336	wLs20_06220
nuoD	**K00333**	WP_011256326.1	WP_011256326.1	QDH18667.1	wCfeJ_00359	wCfeT_00049	WP_041045597.1	wCtub_t1_00476	wDcau_t1_00148	wDi2_01620	WP_110409234.1	LOGDKPFN_00778	wLs20_03560
nuoE	**K00334**	WP_011256792.1	WP_167895735.1	QDH18894.1	wCfeJ_00047	wCfeT_00479	WP_041046177.1	wCtub_t1_00657	wDcau_t1_00039	wDi2_07620	WP_110410158.1	LOGDKPFN_00021	wLs20_04510
nuoF	**K00335**	WP_041571573.1	WP_167896295.1	QDH18160.1	wCfeJ_00220	wCfeT_01300	WP_041045789.1	wCtub_t1_00494	wDcau_t1_00145	wDi2_01430	WP_110410174.1	LOGDKPFN_00938	wLs20_04750
nuoG	**K00336**	WP_011256574.1	WP_167895884.1	QDH18472.1	wCfeJ_00218	wCfeT_00037	WP_041046491.1	wCtub_t1_00052	wDcau_t1_00541	wDi2_06180	WP_110409810.1	LOGDKPFN_01005	wLs20_05220
nuoH	**K00337**	WP_011256573.1	WP_011256573.1	QDH18473.1	wCfeJ_00217	wCfeT_01298	WP_041046489.1	wCtub_t1_00053	wDcau_t1_00540	wDi2_06170	WP_110409808.1	LOGDKPFN_01006	wLs20_05210
nuoI	**K00338**	WP_011256669.1	WP_167895822.1	QDH18156.1	wCfeJ_00445	wCfeT_01038	WP_041046024.1	wCtub_t1_00490	wDcau_t1_00142	wDi2_01470	WP_110409755.1	LOGDKPFN_00908	wLs20_03380
nuoJ	**K00339**	WP_041571486.1	WP_167896091.1	QDH18171.1	wCfeJ_00390	wCfeT_00803	WP_041045164.1	wCtub_t1_00140	wDcau_t1_00209	wDi2_02320	WP_110409970.1	LOGDKPFN_00083	wLs20_03980
nuoK	**K00340**	WP_011256822.1	WP_167896090.1	QDH18170.1	wCfeJ_00391	wCfeT_00804	WP_041045166.1	wCtub_t1_00141	wDcau_t1_00210	wDi2_02330	WP_110409971.1	LOGDKPFN_00082	wLs20_03990
nuoL	**K00341**	WP_011256821.1	WP_167896089.1	QDH18169.1	wCfeJ_00392	wCfeT_00805	WP_041045168.1	wCtub_t1_00142	wDcau_t1_00211	wDi2_02340	WP_110409972.1	LOGDKPFN_00081	wLs20_04000
nuoM	**K00342**	WP_011256820.1	WP_167896088.1	QDH18168.1	wCfeJ_00393	wCfeT_00806	WP_041045170.1	wCtub_t1_00143	wDcau_t1_00212	wDi2_02350	WP_110409973.1	LOGDKPFN_00080	wLs20_04010
nuoN	**K00343**	WP_011256819.1	WP_167896087.1	QDH18167.1	wCfeJ_00394	wCfeT_00807	WP_041045172.1	wCtub_t1_00144	wDcau_t1_00213	wDi2_02360	WP_110409974.1	LOGDKPFN_00079	wLs20_04020

**succinate dehydrogenase**													
sdhC, frdC	**K00241**	WP_011256684.1	WP_167895807.1	QDH18285.1	wCfeJ_00005	wCfeT_01066	WP_041045380.1	wCtub_t1_00421	wDcau_t1_00469	wDi2_05430	WP_110409772.1	LOGDKPFN_00742	wLs20_00880
sdhD, frdD	**K00242**	WP_011256683.1	WP_167895808.1	QDH18286.1	wCfeJ_00004		WP_041045378.1	wCtub_t1_00420	wDcau_t1_00468	wDi2_05420	WP_110409773.1	LOGDKPFN_00743	wLs20_00890
sdhA, frdA	**K00239**	WP_011256646.1	WP_167895839.1	QDH19062.1	wCfeJ_00305	wCfeT_00717	WP_041045103.1	wCtub_t1_00673	wDcau_t1_00650	wDi2_07410	WP_110409873.1	LOGDKPFN_00041	wLs20_04370
sdhB, frdB	**K00240**	WP_011256798.1	WP_011256798.1	QDH18901.1	wCfeJ_00054	wCfeT_01423	WP_041044980.1	wCtub_t1_00693	wDcau_t1_00635	wDi2_07230	WP_110409860.1	LOGDKPFN_00863	wLs20_08880
**F-type ATPase, prokaryotes and chloroplasts**													
ATPF1A, atpA	**K02111**	WP_011256513.1	WP_167895925.1	QDH18733.1	wCfeJ_00916	wCfeT_00169	WP_041045530.1	wCtub_t1_00297	wDcau_t1_00420	wDi2_04000	WP_110409446.1	LOGDKPFN_00580	wLs20_07330
ATPF1B, atpD	**K02112**	WP_041571591.1	WP_041571591.1	QDH19309.1	wCfeJ_00635	wCfeT_00847	WP_041046666.1	wCtub_t1_00016	wDcau_t1_00576	wDi2_06560	WP_110410534.1	LOGDKPFN_00527	wLs20_00200
ATPF1G, atpG	**K02115**	WP_011256902.1	WP_167896148.1	QDH18293.1	wCfeJ_00313	wCfeT_00777	WP_041045916.1	wCtub_t1_00102	wDcau_t1_00170	wDi2_01840	WP_110410512.1	LOGDKPFN_00404	wLs20_00430
ATPF1D, atpH	**K02113**	WP_011256514.1	WP_167895924.1	QDH18732.1	wCfeJ_00917	wCfeT_00168	WP_041045532.1	wCtub_t1_00296	wDcau_t1_00421	wDi2_03990	WP_110409447.1	LOGDKPFN_00581	wLs20_07340
ATPF1E, atpC	**K02114**	WP_011256885.1	WP_167896136.1	QDH18567.1	wCfeJ_00636	wCfeT_00848	WP_041045689.1	wCtub_t1_00017	wDcau_t1_00575	wDi2_06550	WP_110409480.1	LOGDKPFN_00528	wLs20_00210
ATPF0A, atpB	**K02108**	WP_011256658.1	WP_167895832.1	QDH19052.1	wCfeJ_00335	wCfeT_00110	WP_041045659.1	wCtub_t1_00078	wDcau_t1_00552	wDi2_06310	WP_110410255.1	LOGDKPFN_00056	wLs20_04240
						wCfeT_00121							
ATPF0B, atpF	**K02109**	WP_011256655.1	WP_011256655.1	QDH19054.1	wCfeJ_00332	wCfeT_00112	WP_041045653.1	wCtub_t1_00080	wDcau_t1_00554	wDi2_06330	WP_110410252.1	LOGDKPFN_00053	wLs20_04260
		WP_011256656.1	WP_167895833.1	QDH19055.1	wCfeJ_00333	wCfeT_00113	WP_041045655.1	wCtub_t1_00081	wDcau_t1_00555	wDi2_06340	WP_110410253.1	LOGDKPFN_00054	wLs20_04270
						wCfeT_00123							
						wCfeT_00124							
ATPF0C, atpE	**K02110**	WP_011256657.1	WP_011256657.1	QDH19053.1	wCfeJ_00334	wCfeT_00111	WP_041045657.1	wCtub_t1_00079	wDcau_t1_00553	wDi2_06320	WP_110410254.1	LOGDKPFN_00055	wLs20_04250
						wCfeT_00122							
**cytochrome c reductase**													
UQCRFS1, RIP1, petA	**K00411**	WP_011256604.1	WP_167895876.1	QDH18266.1	wCfeJ_00252	wCfeT_00086	WP_041045911.1	wCtub_t1_00548	wDcau_t1_00081	wDi2_00830	WP_110409374.1	LOGDKPFN_00935	wLs20_05780
CYTB, petB	**K00412**	WP_011256972.1	WP_167896250.1	QDH19241.1	wCfeJ_00742	wCfeT_00818	WP_041045067.1	wCtub_t1_00377	wDcau_t1_00299	wDi2_04920	WP_110409821.1	LOGDKPFN_00379	wLs20_02280
CYC1, CYT1, petC	**K00413**	WP_041571516.1	WP_167896337.1	QDH19373.1	wCfeJ_00743	wCfeT_00819	WP_041046525.1	wCtub_t1_00378	wDcau_t1_00298	wDi2_04930	WP_110410562.1	LOGDKPFN_00380	wLs20_02290
**Cytochrome c oxidase**													
COX10, ctaB, cyoE	**K02257**	WP_011256505.1	WP_167895929.1	QDH18752.1	wCfeJ_00907	wCfeT_01387	WP_041045518.1	wCtub_t1_00306	wDcau_t1_00411	wDi2_04100	WP_110409537.1	LOGDKPFN_00472	wLs20_01330
COX11, ctaG	**K02258**	WP_011256689.1	WP_167895802.1	QDH18229.1	wCfeJ_00011	wCfeT_00954	WP_041045326.1	wCtub_t1_00428	wDcau_t1_00475	wDi2_05490	WP_110409692.1	LOGDKPFN_00735	wLs20_00820
COX15, ctaA	**K02259**	WP_050707669.1	WP_167895834.1	QDH19057.1	wCfeJ_00330	wCfeT_00131	WP_052463207.1	wCtub_t1_00038	wDcau_t1_00556	wDi2_06350	WP_110410251.1	LOGDKPFN_00050	wLs20_04290
coxC, ctaE	**K02276**	WP_011256895.1	WP_167896140.1	QDH18493.1	wCfeJ_00018	wCfeT_01045	WP_041045605.1	wCtub_t1_00269	wDcau_t1_00402	wDi2_03700	WP_110409895.1	LOGDKPFN_00516	wLs20_00090
coxA, ctaD	**K02274**	WP_011256506.1	WP_167895928.1	QDH18751.1	wCfeJ_00908	wCfeT_01388	WP_041045519.1	wCtub_t1_00305	wDcau_t1_00412	wDi2_04090	WP_110409538.1	LOGDKPFN_00471	wLs20_01320
coxB, ctaC	**K02275**	WP_011256507.1	WP_011256507.1	QDH18750.1	wCfeJ_00909	wCfeT_01389	WP_041045520.1	wCtub_t1_00303	wDcau_t1_00413	wDi2_04080	WP_110409539.1	LOGDKPFN_00470	wLs20_01310
**Cytochrome bd ubiquinol oxidase**													

cydA	**K00425**			QDH18890.1	wCfeJ_00801								
cydB	**K00426**			QDH19341.1	wCfeJ_00802								
total		36	36	38	38	39	36	36	36	36	36	36	36
**K04112 Cell cycle - Caulobacter**													
murG	**K02563**	WP_011256755.1	WP_167895754.1	QDH18761.1	wCfeJ_00814	wCfeT_01099	WP_041046749.1	wCtub_t1_00226	wDcau_t1_00365	wDi2_03270	WP_110410532.1	LOGDKPFN_00655	wLs20_08000
ftsW, spoVE	**K03588**			QDH18834.1	wCfeJ_00982	wCfeT_00316	WP_041045840.1				WP_110409641.1	LOGDKPFN_00440	
ftsZ	**K03531**	WP_011256800.1	WP_167896066.1	QDH18905.1	wCfeJ_00057	wCfeT_00103	WP_041044986.1			wDi2_07250	WP_110409902.1	LOGDKPFN_00860	wLs20_08910
ftsQ	**K03589**	WP_011256769.1	WP_167895747.1	QDH18539.1	wCfeJ_00823	wCfeT_00082	WP_041045083.1	wCtub_t1_00084		wDi2_02000	WP_110409427.1	LOGDKPFN_00633	wLs20_08200
ftsA	**K03590**	WP_011256314.1	WP_167896204.1	QDH18466.1	wCfeJ_00034	wCfeT_00369	WP_041045268.1			wDi2_06230	WP_110410210.1	LOGDKPFN_00216	wLs20_03090
PerP	**K06985**	WP_041571541.1	WP_041571541.1	QDH18639.1	wCfeJ_00662	wCfeT_00016	WP_041045949.1				WP_110410144.1		
pleC	**K07716**	WP_011256329.1	WP_167896060.1	QDH18624.1	wCfeJ_00416	wCfeT_01230					WP_110409598.1	LOGDKPFN_00368	wLs20_02180
rseP	**K11749**	WP_011256257.1	WP_011256257.1	QDH19230.1	wCfeJ_00194	wCfeT_00888	WP_041046729.1	wCtub_t1_00572	wDcau_t1_00058	wDi2_00540	WP_110409508.1	LOGDKPFN_00924	wLs20_08720
pleD	**K02488**	WP_011256385.1	WP_167895954.1	QDH18650.1	wCfeJ_00787	wCfeT_00533		wCtub_t1_00224	wDcau_t1_00290	wDi2_03240	WP_110409226.1	LOGDKPFN_00131	wLs20_01590
clpX, CLPX	**K03544**	WP_011256750.1	WP_011256750.1	QDH18765.1	wCfeJ_00821	wCfeT_00639	WP_041046352.1	wCtub_t1_00232	wDcau_t1_00371	wDi2_03330	WP_110409702.1	LOGDKPFN_00661	wLs20_08060
clpP, CLPP	**K01358**	WP_011256751.1	WP_011256751.1	QDH18764.1	wCfeJ_00820	wCfeT_00640	WP_041046354.1	wCtub_t1_00231	wDcau_t1_00370	wDi2_03320	WP_110409703.1	LOGDKPFN_00660	wLs20_08050
dnaA	**K02313**	WP_011256475.1	WP_167895988.1	QDH18593.1	wCfeJ_00679	wCfeT_00995	WP_041044912.1	wCtub_t1_00393	wDcau_t1_00360	wDi2_05070	WP_110409593.1	LOGDKPFN_00411	wLs20_00500
dnaB	**K02314**	WP_011256498.1	WP_167895959.1	QDH18777.1	wCfeJ_00061	wCfeT_01400	WP_041045127.1	wCtub_t1_00325	wDcau_t1_00452	wDi2_04350	WP_110409984.1	LOGDKPFN_00557	wLs20_07150
lon	**K01338**	WP_011256749.1	WP_167895757.1	QDH18766.1	wCfeJ_00822	wCfeT_00638	WP_041046350.1	wCtub_t1_00233	wDcau_t1_00372	wDi2_03340	WP_110409701.1	LOGDKPFN_00662	wLs20_08070
total		13	13	14	14	14	12	9	8	11	14	13	12
**K03440 Homologous recombination**													
recJ	**K07462**	WP_011256325.1	WP_167896336.1	QDH18808.1	wCfeJ_00079	wCfeT_00788	WP_041046660.1	wCtub_t1_00735		wDi2_06790	WP_110409532.1	LOGDKPFN_00777	wLs20_03550
ssb	**K03111**	WP_011256412.1	WP_167896037.1	QDH18863.1	wCfeJ_00460	wCfeT_00697	WP_041046254.1	wCtub_t1_00118	wDcau_t1_00192	wDi2_02100	WP_110409575.1	LOGDKPFN_00029	wLs20_04470
recA	**K03553**	WP_011256625.1	WP_011256625.1	QDH19258.1	wCfeJ_00244	wCfeT_01155	WP_041045712.1	wCtub_t1_00578			WP_110410451.1		wLs20_05870
recF	**K03629**	WP_011256327.1	WP_167896062.1	QDH18623.1	wCfeJ_00418	wCfeT_00070	WP_041046102.1	wCtub_t1_00699			WP_110409675.1		
recO	**K03584**	WP_011256487.1	WP_167895978.1	QDH18651.1	wCfeJ_01046	wCfeT_00917	WP_041045191.1	wCtub_t1_00085			WP_110409777.1		
recR	**K06187**	WP_011256943.1	WP_167896183.1	QDH18250.1	wCfeJ_00597	wCfeT_00292	WP_041046430.1	wCtub_t1_00522			WP_110409781.1		
polA	**K02335**	WP_011256922.1	WP_167896163.1	QDH18141.1	wCfeJ_01064	wCfeT_00005	WP_041045260.1	wCtub_t1_00350	wDcau_t1_00324	wDi2_04600	WP_110410263.1	LOGDKPFN_01016	wLs20_08650
dnaE	**K02337**	WP_011256697.1	WP_167895797.1	QDH19338.1	wCfeJ_00843	wCfeT_01549	WP_041046477.1	wCtub_t1_00255	wDcau_t1_00389	wDi2_03550	WP_110409962.1	LOGDKPFN_00137	wLs20_01600
ruvA	**K03550**	WP_011256450.1	WP_167896006.1	QDH18214.1	wCfeJ_00111	wCfeT_00862	WP_041046324.1	wCtub_t1_00647			WP_110409770.1		
ruvB	**K03551**	WP_011256451.1	WP_167896005.1	QDH18215.1	wCfeJ_00112	wCfeT_00861	WP_041046322.1	wCtub_t1_00646			WP_110409769.1		

ruvC	**K01159**	WP_011256894.1	WP_167896139.1	QDH18492.1	wCfeJ_00017	wCfeT_01046	WP_041045603.1	wCtub_t1_00270			WP_110409896.1		wLs20_00110
recG	**K03655**	WP_011256833.1	WP_167896101.1	QDH19134.1	wCfeJ_00406	wCfeT_00248	WP_041044937.1			wDi2_06820	WP_112477178.1	LOGDKPFN_00255	wLs20_02750
total		12	12	12	12	12	12	11	3	5	12	5	7
**K02010 ABC transporters iron(III) transport system**													
AfuA	**K02012**	WP_011256596.1	WP_167895868.1	QDH19159.1	wCfeJ_00355	wCfeT_00407	WP_041046376.1			wDi2_00250	WP_110409389.1		
AfuB	**K02011**	WP_011256241.1	WP_167895723.1	QDH18193.1	wCfeJ_00165	wCfeT_00820	WP_041046008.1			wDi2_00730	WP_110409663.1	LOGDKPFN_00207	wLs20_03140
												LOGDKPFN_00208	
AfuC	**K02010**	WP_011256875.1	WP_011256875.1	QDH18479.1	wCfeJ_00647	wCfeT_00852	WP_041045636.1			wDi2_06460	WP_110410468.1	LOGDKPFN_00541	wLs20_00310
**phosphate transport system**													
PstS	**K02040**	WP_011256492.1	WP_011256492.1	QDH18822.1	wCfeJ_01014	wCfeT_00705	WP_041045248.1	wCtub_t1_00429	wDcau_t1_00477	wDi2_05500	WP_110410334.1	LOGDKPFN_00570	wLs20_07230
PstC	**K02037**	WP_041571592.1	WP_167896331.1	QDH19308.1	wCfeJ_00634	wCfeT_01134	WP_041046667.1	wCtub_t1_00015	wDcau_t1_00577	wDi2_06570	WP_110409481.1	LOGDKPFN_00526	wLs20_00190
PstA	**K02038**	WP_011256432.1	WP_167896021.1	QDH18125.1	wCfeJ_00369	wCfeT_01086	WP_041045776.1	wCtub_t1_00617	wDcau_t1_00678	wDi2_00090	WP_110409647.1	LOGDKPFN_00682	wLs20_05950
PstB	**K02036**	WP_011256379.1	WP_167895948.1	QDH18815.1	wCfeJ_00991	wCfeT_01147	WP_041045036.1	wCtub_t1_00284	wDcau_t1_00341	wDi2_03860	WP_110409954.1	LOGDKPFN_00490	wLs20_01420
**lipoprotein-releasing system**													
lolC_E	**K09808**	WP_041571469.1	WP_167895810.1	QDH18289.1	wCfeJ_01055	wCfeT_00045	WP_041046260.1	wCtub_t1_00418	wDcau_t1_00465	wDi2_05400	WP_110410353.1	LOGDKPFN_00154	wLs20_01770
LolD	**K09810**	WP_011256225.1	WP_167895711.1	QDH18690.1	wCfeJ_00071	wCfeT_01020	WP_041045856.1	wCtub_t1_00158	wDcau_t1_00226	wDi2_02500	WP_110410541.1	LOGDKPFN_00884	wLs20_06960
**heme exporter**													
CcmC	**K02195**	WP_011256832.1	WP_167896100.1	QDH18789.1	wCfeJ_00199	wCfeT_01309	WP_041046697.1	wCtub_t1_00138	wDcau_t1_00207	wDi2_02300	WP_110409416.1	LOGDKPFN_00259	wLs20_02720
CcmB	**K02194**	WP_011256776.1	WP_167895744.1	QDH19224.1	wCfeJ_00715	wCfeT_01438	WP_041045010.1	wCtub_t1_00068	wDcau_t1_00528	wDi2_06010	WP_110409364.1	LOGDKPFN_00625	wLs20_08290
CcmA	**K02193**	WP_011256216.1	WP_167895704.1	QDH19038.1	wCfeJ_00515	wCfeT_00680	WP_041045869.1	wCtub_t1_00483	wDcau_t1_00136	wDi2_01530	WP_110409788.1	LOGDKPFN_00375	wLs20_02230
**phospholipid transport system**													
MlaC	**K07323**	WP_041571568.1	WP_167895845.1		wCfeJ_00339	wCfeT_01554	WP_041046505.1			wDi2_02740	WP_110409677.1	LOGDKPFN_00945	wLs20_05670
													wLs20_05690
MlaD	**K02067**	WP_011256220.1	WP_167895708.1	QDH18222.1	wCfeJ_00382	wCfeT_01058	WP_041046042.1				WP_110409250.1	LOGDKPFN_00370	
MlaE	**K02066**	WP_011256815.1	WP_167896083.1	QDH18151.1	wCfeJ_00311	wCfeT_01531	WP_041045181.1				WP_110410529.1	LOGDKPFN_00074	wLs20_04080
MlaF	**K02065**	WP_011256816.1	WP_167896084.1	QDH18150.1	wCfeJ_00312	wCfeT_01530	WP_041045178.1				WP_110409375.1		
**zinc transport system**													
ZnuA	**K09815**	WP_041571504.1	WP_041571504.1	QDH19211.1	wCfeJ_00740	wCfeT_01243	WP_041045061.1	wCtub_t1_00359	wDcau_t1_00316	wDi2_04700	WP_110409413.1	LOGDKPFN_00726	wLs20_09030
ZnuB	**K09816**	WP_011256376.1	WP_011256376.1	QDH18770.1	wCfeJ_00996	wCfeT_00221	WP_041045133.1	wCtub_t1_00281	wDcau_t1_00344	wDi2_03830	WP_110409829.1	LOGDKPFN_00487	wLs20_01500
ZnuC	**K09817**	WP_011256912.1	WP_167896156.1	QDH19212.1	wCfeJ_00739	wCfeT_01242	WP_041045063.1	wCtub_t1_00360	wDcau_t1_00315	wDi2_04710	WP_110409412.1	LOGDKPFN_00725	wLs20_09040

**biotin transport system**													
BioY	**K03523**	WP_011256714.1	WP_167895782.1	QDH18270.1	wCfeJ_00689	wCfeT_00521	WP_041045585.1			wDi2_03510	WP_110409280.1	LOGDKPFN_00811	wLs20_05350
total		20	20	19	20	20	20	12	12	16	20	18	17
**K00564 Glycerophospholipid metabolism**													
araM, egsA	**K00096**			QDH18853.1	wCfeJ_00827		WP_041045073.1	wCtub_t1_00039	wDcau_t1_00514	wDi2_05880	WP_110409407.1		
gpsA	**K00057**	WP_011256795.1	WP_011256795.1	QDH18897.1	wCfeJ_00050	wCfeT_00354	WP_041044970.1	wCtub_t1_00697	wDcau_t1_00631	wDi2_07190	WP_112477233.1	LOGDKPFN_00866	wLs20_08850
plsY	**K08591**	WP_011256621.1	WP_167895855.1	QDH19376.1	wCfeJ_00284	wCfeT_00364	WP_041045704.1	wCtub_t1_00649	wDcau_t1_00024	wDi2_07710	WP_110410530.1	LOGDKPFN_00676	wLs20_05930
plsC	**K00655**	WP_041571507.1	WP_167896171.1	QDH18184.1	wCfeJ_00701	wCfeT_00914	WP_041044962.1	wCtub_t1_00237	wDcau_t1_00374	wDi2_03390	WP_110410521.1	LOGDKPFN_00005	wLs20_04660
dgkA, DGK	**K00901**			QDH18178.1		wCfeT_01190					WP_110409833.1		
pld	**K17717**		WP_167896304.1		wCfeJ_00211						WP_110410581.1		
E2.7.7.41, CDS1, CDS2, cdsA	**K00981**	WP_011257000.1	WP_167896277.1	QDH19032.1	wCfeJ_00794	wCfeT_00418	WP_041046114.1	wCtub_t1_00407	wDcau_t1_00439	wDi2_05280	WP_110409887.1	LOGDKPFN_00453	wLs20_01160
CHO1, pssA	**K17103**	WP_011256623.1	WP_011256623.1	QDH19260.1	wCfeJ_00287	wCfeT_00402	WP_041045708.1	wCtub_t1_00651	wDcau_t1_00026	wDi2_07690	WP_110410449.1	LOGDKPFN_00672	wLs20_05890
psd, PISD	**K01613**	WP_041571460.1	WP_167895853.1	QDH19259.1	wCfeJ_00288	wCfeT_00401	WP_041045710.1	wCtub_t1_00652	wDcau_t1_00027	wDi2_07680	WP_110410450.1	LOGDKPFN_00671	wLs20_05880
pgsA, PGS1	**K00995**	WP_011256871.1		QDH19107.1			WP_041045757.1	wCtub_t1_00373	wDcau_t1_00303	wDi2_04870	WP_110409856.1	LOGDKPFN_00994	wLs20_01040
pgpA	**K01095**	WP_041571481.1	WP_167896321.1	QDH18898.1	wCfeJ_00051	wCfeT_00355	WP_041044972.1	wCtub_t1_00696	wDcau_t1_00632	wDi2_07200	WP_110409863.1		
total		8	8	10	9	8	9	9	9	9	11	7	7
**K00860 Porphyrin and chlorophyll metabolism**													
ALAS	**K00643**	WP_011256334.1	WP_011256334.1	QDH18628.1	wCfeJ_00410	wCfeT_00205	WP_041046044.1	wCtub_t1_00705	wDcau_t1_00624	wDi2_07120	WP_110409513.1	LOGDKPFN_00363	wLs20_02130
EARS, gltX	**K01885**	WP_011256643.1	WP_167895837.1	QDH18860.1	wCfeJ_00327	wCfeT_00683	WP_041045649.1	wCtub_t1_00036	wDcau_t1_00558	wDi2_06370	WP_110409937.1	LOGDKPFN_00047	wLs20_04320
		WP_011256651.1	WP_167895841.1	QDH19060.1	wCfeJ_00216	wCfeT_01538	WP_041046680.1	wCtub_t1_00677	wDcau_t1_00647	wDi2_07370	WP_110410484.1	LOGDKPFN_00934	wLs20_05760
hemB, ALAD	**K01698**	WP_041571452.1	WP_167896305.1	QDH18474.1	wCfeJ_00485	wCfeT_01033	WP_041045162.1	wCtub_t1_00120	wDcau_t1_00194	wDi2_02120	WP_110409806.1	LOGDKPFN_00891	wLs20_05180
hemC, HMBS	**K01749**	WP_011256974.1	WP_167896255.1	QDH19022.1	wCfeJ_01100	wCfeT_00420	WP_041045183.1	wCtub_t1_00364	wDcau_t1_00311	wDi2_04750	WP_110409616.1	LOGDKPFN_00158	wLs20_01790
hemD, UROS	**K01719**	WP_011256925.1	WP_011256925.1	QDH18143.1	wCfeJ_00805	wCfeT_00775	WP_041046238.1	wCtub_t1_00345	wDcau_t1_00327	wDi2_04550	WP_110409698.1	LOGDKPFN_00814	wLs20_05320
hemE, UROD	**K01599**	WP_041571524.1	WP_167895693.1	QDH19272.1	wCfeJ_00259	wCfeT_01286	WP_041046675.1	wCtub_t1_00625	wDcau_t1_00002	wDi2_00010	WP_110409669.1	LOGDKPFN_00317	wLs20_06420
CPOX, hemF	**K00228**	WP_011256906.1	WP_167896151.1	QDH18278.1	wCfeJ_00971	wCfeT_01007	WP_041044932.1	wCtub_t1_00318	wDcau_t1_00428	wDi2_04250	WP_110409498.1	LOGDKPFN_00549	wLs20_00370
hemJ	**K08973**	WP_041571401.1	WP_167895936.1	QDH19044.1	wCfeJ_00988	wCfeT_00460	WP_041045043.1	wCtub_t1_00287	wDcau_t1_00338	wDi2_03890	WP_110410277.1	LOGDKPFN_00167	wLs20_01870
hemH, FECH	**K01772**	WP_011256916.1	WP_011256916.1	QDH18253.1	wCfeJ_01070	wCfeT_00350	WP_041045057.1	wCtub_t1_00356	wDcau_t1_00319	wDi2_04660	WP_110409942.1	LOGDKPFN_00729	wLs20_09000
bfr	**K03594**		WP_167896320.1	QDH18305.1	wCfeJ_00209	wCfeT_01200	WP_041046194.1				WP_110409743.1	LOGDKPFN_00820	wLs20_06760
COX10, ctaB, cyoE	**K02257**	WP_011256505.1	WP_167895929.1	QDH18752.1	wCfeJ_00907	wCfeT_01387	WP_041045518.1	wCtub_t1_00306	wDcau_t1_00411	wDi2_04100	WP_110409537.1	LOGDKPFN_00472	wLs20_01330
COX15, ctaA	**K02259**	WP_050707669.1	WP_167895834.1	QDH19057.1	wCfeJ_00330	wCfeT_00131	WP_052463207.1	wCtub_t1_00038	wDcau_t1_00556	wDi2_06350	WP_110410251.1	LOGDKPFN_00050	wLs20_04290

total		12	13	13	13	13	13	12	12	12	13	13	13
**K00230 Purine metabolism**													
PRPS, prsA	**K00948**	WP_011256305.1	WP_011256305.1	QDH18522.1	wCfeJ_00273	wCfeT_01312	WP_041046242.1	wCtub_t1_00592	wDcau_t1_00654	wDi2_00350	WP_110409377.1	LOGDKPFN_00903	wLs20_05100
purF, PPAT	**K00764**	WP_011256454.1	WP_167896003.1	QDH18218.1	wCfeJ_00353	wCfeT_00896	WP_041046402.1	wCtub_t1_00192	wDcau_t1_00260	wDi2_02870	WP_110410305.1	LOGDKPFN_00332	wLs20_06260
purD	**K01945**	WP_011256663.1	WP_167895827.1	QDH18525.1	wCfeJ_00075	wCfeT_00234	WP_041046425.1	wCtub_t1_00633	wDcau_t1_00010	wDi2_07870	WP_110410561.1	LOGDKPFN_00062	wLs20_04190
purN	**K11175**	WP_041571458.1	WP_167896299.1	QDH18871.1	wCfeJ_00123	wCfeT_00937	WP_041046407.1	wCtub_t1_00637	wDcau_t1_00016	wDi2_07830	WP_110409673.1	LOGDKPFN_00876	wLs20_07080
purQ	**K23265**	WP_011256433.1	WP_167896020.1	QDH18126.1	wCfeJ_00368	wCfeT_01085	WP_041045778.1	wCtub_t1_00616	wDcau_t1_00677	wDi2_00100	WP_110409648.1	LOGDKPFN_00681	wLs20_05940
purSL	**K23270**	WP_011256470.1	WP_167895992.1	QDH19199.1	wCfeJ_00152	wCfeT_00684	WP_041045388.1	wCtub_t1_00194	wDcau_t1_00263	wDi2_02900	WP_110410539.1	LOGDKPFN_00503	wLs20_00020
purM	**K01933**	WP_041571547.1	WP_167896025.1	QDH18121.1	wCfeJ_00373	wCfeT_01290	WP_041045768.1	wCtub_t1_00621	wDcau_t1_00682	wDi2_00050	WP_110410191.1	LOGDKPFN_00687	wLs20_06000
purE	**K01588**	WP_011256595.1	WP_011256595.1	QDH18610.1	wCfeJ_00420	wCfeT_00675	WP_041046091.1	wCtub_t1_00104	wDcau_t1_00168	wDi2_01820	WP_110409920.1	LOGDKPFN_00399	wLs20_02430
purC	**K01923**	WP_011256428.1	WP_011256428.1	QDH18122.1	wCfeJ_00372	wCfeT_01293	WP_041045770.1	wCtub_t1_00620	wDcau_t1_00681	wDi2_00060	WP_110409643.1	LOGDKPFN_00686	wLs20_05990
purB, ADSL	**K01756**	WP_011256701.1	WP_167895794.1	QDH18854.1							WP_110409965.1		
purH	**K00602**	WP_041571563.1	WP_167896301.1	QDH19179.1	wCfeJ_00810	wCfeT_01319	WP_041046184.1	wCtub_t1_00732	wDcau_t1_00598	wDi2_06830	WP_110409952.1		wLs20_03290
ndk, NME	**K00940**	WP_011256914.1	WP_167896157.1	QDH18251.1	wCfeJ_01072	wCfeT_00549	WP_041045059.1	wCtub_t1_00358	wDcau_t1_00317	wDi2_04680	WP_110409940.1	LOGDKPFN_00727	wLs20_09020
purA, ADSS	**K01939**	WP_011256472.1	WP_167895990.1	QDH19332.1	wCfeJ_00682	wCfeT_00391	WP_041046767.1	wCtub_t1_00395	wDcau_t1_00357	wDi2_05100	WP_110409230.1	LOGDKPFN_00407	wLs20_00450
gmk	**K00942**	WP_011256778.1	WP_011256778.1	QDH19064.1	wCfeJ_00479	wCfeT_01440	WP_041046741.1	wCtub_t1_00066	wDcau_t1_00530	wDi2_06030	WP_110409362.1	LOGDKPFN_00623	wLs20_08310
dgt	**K01129**	WP_011256227.1	WP_011256227.1	QDH18688.1	wCfeJ_00069	wCfeT_00692	WP_041045852.1	wCtub_t1_00156	wDcau_t1_00224	wDi2_02480	WP_110409589.1	LOGDKPFN_00882	wLs20_06980
surE	**K03787**	WP_011256747.1	WP_011256747.1	QDH19238.1	wCfeJ_00751	wCfeT_00476	WP_041045599.1	wCtub_t1_00388	wDcau_t1_00291	wDi2_05010	WP_110410589.1	LOGDKPFN_01018	wLs20_07940
nrdA, nrdE	**K00525**	WP_011256892.1	WP_011256892.1	QDH18562.1	wCfeJ_00629	wCfeT_01080	WP_041045695.1	wCtub_t1_00008	wDcau_t1_00584	wDi2_06630	WP_110409368.1	LOGDKPFN_00519	wLs20_00130
nrdB, nrdF	**K00526**	WP_011256466.1	WP_011256466.1	QDH18654.1	wCfeJ_00611	wCfeT_00924	WP_041046461.1	wCtub_t1_00510	wDcau_t1_00122	wDi2_01310	WP_110410517.1	LOGDKPFN_00554	wLs20_07130
guaA, GMPS	**K01951**	WP_011256641.1	WP_167895843.1	QDH18561.1	wCfeJ_00127	wCfeT_00467	WP_041046429.1	wCtub_t1_00634	wDcau_t1_00011	wDi2_07860	WP_110409734.1	LOGDKPFN_00930	wLs20_05730
purK	**K01589**	WP_011256243.1	WP_167895724.1	QDH18190.1	wCfeJ_00163	wCfeT_01117	WP_041046004.1	wCtub_t1_00559	wDcau_t1_00091	wDi2_00710		LOGDKPFN_00211	wLs20_03120
IMPDH, guaB	**K00088**	WP_011256725.1	WP_167895777.1	QDH18546.1	wCfeJ_00354	wCfeT_00907	WP_041044994.1	wCtub_t1_00070	wDcau_t1_00543	wDi2_06200	WP_110410241.1	LOGDKPFN_00718	wLs20_04940
total		21	21	21	20	20	20	20	20	20	20	19	20
**K00240 Pyrimidine metabolism**													
carB, CPA2	**K01955**	WP_011256710.1	WP_167895786.1	QDH18619.1	wCfeJ_00918	wCfeT_00084	WP_041045384.1	wCtub_t1_00414	wDcau_t1_00446	wDi2_05350	WP_110409721.1	LOGDKPFN_00753	wLs20_00980
carA, CPA1	**K01956**	WP_011256852.1	WP_167896117.1	QDH18703.1	wCfeJ_00495	wCfeT_00137	WP_041046065.1	wCtub_t1_00459	wDcau_t1_00507	wDi2_05780	WP_110409476.1	LOGDKPFN_00229	wLs20_03000
pyrB, PYR2	**K00609**	WP_011256583.1	WP_011256583.1	QDH19158.1	wCfeJ_00357	wCfeT_01008	WP_041045966.1	wCtub_t1_00601	wDcau_t1_00661	wDi2_00260	WP_110410150.1	LOGDKPFN_00852	wLs20_08480
URA4, pyrC	**K01465**	WP_011256644.1	WP_011256644.1	QDH18643.1	wCfeJ_00215	wCfeT_00308	WP_041045721.1	wCtub_t1_00676	wDcau_t1_00648	wDi2_07390	WP_110410493.1	LOGDKPFN_00039	wLs20_04390
DHODH, pyrD	**K00254**	WP_011256300.1	WP_011256300.1	QDH18298.1	wCfeJ_00131	wCfeT_00570	WP_041045394.1	wCtub_t1_00668	wDcau_t1_00028	wDi2_07500	WP_110409871.1	LOGDKPFN_00894	wLs20_05170
pyrE	**K00762**	WP_011256987.1	WP_011256987.1	QDH18645.1	wCfeJ_00755	wCfeT_01151	WP_041045002.1	wCtub_t1_00362	wDcau_t1_00313	wDi2_04730	WP_110409640.1	LOGDKPFN_00462	wLs20_01290

pyrF	**K01591**	WP_011256984.1	WP_167896267.1	QDH19082.1	wCfeJ_00925	wCfeT_00403	WP_041046286.1	wCtub_t1_00320	wDcau_t1_00329	wDi2_04260	WP_110410318.1	LOGDKPFN_00752	wLs20_00970
surE	**K03787**	WP_011256747.1	WP_011256747.1	QDH19238.1	wCfeJ_00751	wCfeT_00476	WP_041045599.1	wCtub_t1_00388	wDcau_t1_00291	wDi2_05010	WP_110410589.1	LOGDKPFN_01018	wLs20_07940
pyrH	**K09903**	WP_011257003.1	WP_167896279.1	QDH19029.1	wCfeJ_00797	wCfeT_00415	WP_041046121.1	wCtub_t1_00410	wDcau_t1_00442	wDi2_05310	WP_110409892.1	LOGDKPFN_00457	wLs20_01190
ndk, NME	**K00940**	WP_011256914.1	WP_167896157.1	QDH18251.1	wCfeJ_01072	wCfeT_00549	WP_041045059.1	wCtub_t1_00358	wDcau_t1_00317	wDi2_04680	WP_110409940.1	LOGDKPFN_00727	wLs20_09020
dcd	**K01494**	WP_011256491.1	WP_167895976.1	QDH18823.1	wCfeJ_01009	wCfeT_00973	WP_041045442.1	wCtub_t1_00327	wDcau_t1_00332	wDi2_04310	WP_110409218.1	LOGDKPFN_00567	wLs20_07220
pyrG, CTPS	**K01937**	WP_011256370.1	WP_167895942.1	QDH19089.1	wCfeJ_00876	wCfeT_01418	WP_041045278.1	wCtub_t1_00276	wDcau_t1_00349	wDi2_03780	WP_110410303.1	LOGDKPFN_00163	wLs20_01840
ndk, NME	**K00940**	WP_011256914.1	WP_167896157.1	QDH18251.1	wCfeJ_01072	wCfeT_00549	WP_041045059.1	wCtub_t1_00358	wDcau_t1_00317	wDi2_04680	WP_110409940.1	LOGDKPFN_00727	wLs20_09020
E1.17.4.1A, nrdA, nrdE	**K00525**	WP_011256892.1	WP_011256892.1	QDH18562.1	wCfeJ_00629	wCfeT_01080	WP_041045695.1	wCtub_t1_00008	wDcau_t1_00584	wDi2_06630	WP_110409368.1	LOGDKPFN_00519	wLs20_00130
E1.17.4.1B, nrdB, nrdF	**K00526**	WP_011256466.1	WP_011256466.1	QDH18654.1	wCfeJ_00611	wCfeT_00924	WP_041046461.1	wCtub_t1_00510	wDcau_t1_00122	wDi2_01310	WP_110410517.1	LOGDKPFN_00554	wLs20_07130
dcd	**K01494**	WP_011256491.1	WP_167895976.1	QDH18823.1	wCfeJ_01009	wCfeT_00973	WP_041045442.1	wCtub_t1_00327	wDcau_t1_00332	wDi2_04310	WP_110409218.1	LOGDKPFN_00567	wLs20_07220
dut, DUT	**K01520**	WP_041571555.1	WP_167896308.1	QDH18634.1	wCfeJ_00176	wCfeT_00635	WP_041046540.1	wCtub_t1_00688	wDcau_t1_00639	wDi2_07280	WP_110409768.1	LOGDKPFN_00976	wLs20_07860
tmk, DTYMK	**K00943**	WP_011256349.1	WP_167896044.1	QDH19288.1	wCfeJ_00248	wCfeT_01441	WP_041046727.1	wCtub_t1_00552	wDcau_t1_00085	wDi2_00790	WP_110409817.1	LOGDKPFN_00816	wLs20_06720
thyX, thy1	**K03465**	WP_011256938.1	WP_167896177.1	QDH18263.1	wCfeJ_00309	wCfeT_01145	WP_041045735.1	wCtub_t1_00545	wDcau_t1_00078	wDi2_00890	WP_110409558.1	LOGDKPFN_00109	wLs20_03820
total		19	19	19	19	19	19	19	19	19	19	19	19
**K00750 Pyridoxine metabolism**													
pdxH, PNPO	**K00275**			QDH19279.1	wCfeJ_00522		WP_041045403.1	wCtub_t1_00480	wDcau_t1_00132	wDi2_01590	WP_110410165.1	LOGDKPFN_00956	
pdxJ	**K03474**			QDH18504.1	wCfeJ_00708		WP_041045024.1	wCtub_t1_00047	wDcau_t1_00522	wDi2_05950	WP_110409428.1		
total		0	0	2	2	0	2	2	2	2	2	1	0

		Wolbachia											
	K0	wLug	wMel	wMhie	wNfla	wOo	wOv	wPip	wPpe	wStri	wTpre	wVulC	wWb
K00730 Thiamine metabolism													
iscS, NFS1	K04487	wLug_00451	WP_010081988.1	KIHBGAEE_00515	LKKDFFFJ_00530	wOo_06060	WP_025264087.1	WP_007302229.1	WP_070064703.1	AOCBPJOI_00882	WP_068652018.1	KLT22481.1	WP_088414480.1
		wLug_00581	WP_010962973.1	KIHBGAEE_00569	LKKDFFFJ_01116	wOo_06420	WP_025264112.1	WP_007302400.1	WP_070065168.1	AOCBPJOI_00975	WP_068652059.1	KLT22335.1	WP_088415327.1
tenA	K03707	wLug_00292	WP_010962390.1		LKKDFFFJ_01228			WP_007302512.1		AOCBPJOI_01135		KLT22624.1	
		wLug_00293						WP_007302513.1		AOCBPJOI_01136		KLT22625.1	
adk, AK	K00939	wLug_00237	WP_010962746.1	KIHBGAEE_00346	LKKDFFFJ_00183	wOo_07920	WP_025264197.1	WP_007302552.1	WP_070065334.1	AOCBPJOI_01178	WP_068651644.1	KLT23222.1	WP_088414083.1
total		5	4	3	4	3	3	5	3	5	3	5	3
**K00740 Riboflavin metabolism**													
ribA, RIB1	**K01497**	wLug_00148	WP_010082649.1		LKKDFFFJ_00254	wOo_05610	WP_025264063.1	WP_007302608.1		AOCBPJOI_01253	WP_068652574.1	KLT22475.1	WP_088415180.1
ribD	**K11752**	wLug_00575	WP_010962779.1	KIHBGAEE_00521	LKKDFFFJ_01110			WP_012481923.1		AOCBPJOI_00876	WP_068652028.1	KLT22653.1	WP_088415325.1
ribB, RIB3	**K02858**	wLug_00228	WP_010962739.1	KIHBGAEE_00681	LKKDFFFJ_00175	wOo_06530	WP_007302560.1	WP_070065343.1	WP_070065343.1	AOCBPJOI_01187	WP_068651629.1	KLT23213.1	WP_088414101.1
ribH, RIB4	**K00794**	wLug_01192	WP_006279647.1	KIHBGAEE_00541	LKKDFFFJ_00418	wOo_05500		WP_007302797.1		AOCBPJOI_00061	WP_068651225.1	KLT22974.1	WP_088415468.1

ribE, RIB5	**K00793**	wLug_00306	WP_010962384.1	KIHBGAEE_00823	LKKDFFFJ_00376			WP_007302503.1		AOCBPJOI_01124	WP_068651717.1	KLT23169.1	WP_088415045.1
ribF	**K11753**	wLug_00777	WP_010082493.1	KIHBGAEE_00977	LKKDFFFJ_00080	wOo_03530		WP_007301939.1		AOCBPJOI_00457	WP_068651113.1	KLT21729.1	WP_088414322.1
total		6	6	4	6	4	2	6	1	6	6	6	6
**K00780 Biotin metabolism**													
bioC	**K02169**	wLug_01483			LKKDFFFJ_00584					AOCBPJOI_00230		KLT21798.1	
fabF, OXSM, CEM1	**K09458**	wLug_01404	WP_010963089.1	KIHBGAEE_00613	LKKDFFFJ_00346	wOo_03770	WP_025263950.1	WP_007302174.1		AOCBPJOI_00719	WP_068652321.1	KLT22033.1	
fabG, OAR1	**K00059**	wLug_00225	WP_010962736.1	KIHBGAEE_00684	LKKDFFFJ_00172	wOo_06500	WP_025264118.1	WP_007302563.1	WP_070064622.1	AOCBPJOI_01190	WP_068651623.1	KLT23210.1	WP_088414107.1
fabZ	**K02372**	wLug_00404	WP_010082082.1	KIHBGAEE_00100	LKKDFFFJ_00462	wOo_02740	WP_014868871.1	WP_006014455.1	WP_070064951.1	AOCBPJOI_01023	WP_068651904.1	KLT22206.1	WP_088414233.1
fabI	**K00208**	wLug_01351	WP_006280349.1	KIHBGAEE_00857	LKKDFFFJ_01204	wOo_01730	WP_014868810.1	WP_007302843.1	WP_070065243.1	AOCBPJOI_01535	WP_068651336.1	KLT22992.1	WP_088414739.1
bioF	**K00652**	wLug_01485			LKKDFFFJ_00582					AOCBPJOI_00232		KLT21796.1	
bioA	**K00833**	wLug_01481			LKKDFFFJ_00586					AOCBPJOI_00228		KLT21800.1	
bioD	**K01935**	wLug_01482			LKKDFFFJ_00585					AOCBPJOI_00229		KLT21799.1	
bioB	**K01012**	wLug_01486			LKKDFFFJ_00581					AOCBPJOI_00233		KLT21795.1	
birA	**K03524**	wLug_00471	WP_010082374.1	KIHBGAEE_00256	LKKDFFFJ_00556	wOo_06210	WP_025264096.1	WP_007302384.1	WP_070064755.1	AOCBPJOI_00949	WP_068651942.1	KLT22316.1	WP_088415346.1
total		10	5	5	10	5	5	5	4	10	5	10	4
**K00061 Fatty acid biosynthesis**													
fabD	**K00645**	wLug_00738	WP_010962894.1	KIHBGAEE_00246	LKKDFFFJ_00750	wOo_03130	WP_025264388.1	WP_007302089.1	WP_070064750.1	AOCBPJOI_00493	WP_068651076.1	KLT22440.1	WP_088414257.1
fabH	**K00648**	wLug_00413	WP_010962965.1	KIHBGAEE_00784	LKKDFFFJ_00539	wOo_06160	WP_025264093.1	WP_012481973.1	WP_070065180.1	AOCBPJOI_01014	WP_068651888.1	KLT21979.1	WP_088415336.1
fabF, OXSM, CEM1	**K09458**	wLug_01404	WP_010963089.1	KIHBGAEE_00613	LKKDFFFJ_00346	wOo_03770	WP_025263950.1	WP_007302174.1		AOCBPJOI_00719	WP_068652321.1	KLT22033.1	
fabG, OAR1	**K00059**	wLug_00225	WP_010962736.1	KIHBGAEE_00684	LKKDFFFJ_00172	wOo_06500	WP_025264118.1	WP_007302563.1	WP_070064622.1	AOCBPJOI_01190	WP_068651623.1	KLT23210.1	WP_088414107.1
fabZ	**K02372**	wLug_00404	WP_010082082.1	KIHBGAEE_00100	LKKDFFFJ_00462	wOo_02740	WP_014868871.1	WP_006014455.1	WP_070064951.1	AOCBPJOI_01023	WP_068651904.1	KLT22206.1	WP_088414233.1
fabI	**K00208**	wLug_01351	WP_006280349.1	KIHBGAEE_00857	LKKDFFFJ_01204	wOo_01730	WP_014868810.1	WP_007302843.1	WP_070065243.1	AOCBPJOI_01535	WP_068651336.1	KLT22992.1	WP_088414739.1
fabK	**K02371**	wLug_01276	WP_010082023.1	KIHBGAEE_00948	LKKDFFFJ_00325	wOo_02040	WP_025264322.1	WP_007302056.1		AOCBPJOI_00601	WP_068652375.1	KLT22569.1	WP_088414883.1
total		7	7	7	7	7	7	7	5	7	7	7	6
**K00790 Folate biosynthesis**													
ribA, RIB1	**K01497**	wLug_00148	WP_010082649.1		LKKDFFFJ_00254	wOo_05610	WP_025264063.1	WP_007302608.1		AOCBPJOI_01253	WP_068652574.1	KLT22653.1	WP_088415180.1
folB	**K01633**	wLug_01317		KIHBGAEE_00239	LKKDFFFJ_00086	wOo_05290	WP_025264044.1	WP_007302114.1		AOCBPJOI_00655	WP_068652070.1	KLT22021.1	
folKP	**K13941**	wLug_00511		KIHBGAEE_00238	LKKDFFFJ_00087	wOo_05280	WP_025264043.1			AOCBPJOI_00654	WP_068652067.1	KLT22020.1	
folC	**K11754**	wLug_01316	WP_010963010.1	KIHBGAEE_00169	LKKDFFFJ_01246	wOo_05830	WP_025264075.1	WP_012481955.1	WP_070064581.1	AOCBPJOI_00816	WP_068652177.1	KLT21699.1	
DHFR, folA	**K00287**	wLug_01315	WP_010962904.1	KIHBGAEE_00237	LKKDFFFJ_00959	wOo_05270	WP_025264042.1	WP_007302111.1		AOCBPJOI_00653	WP_068652065.1	KLT22019.1	
total		5	3	4	5	5	5	4	1	5	5	5	1
**K03070 Bacterial secretion system**													
tolC	**K12340**	wLug_01251	WP_010082597.1	KIHBGAEE_00027	LKKDFFFJ_01165	wOo_00090	WP_025264220.1	WP_012481865.1	WP_070065063.1	AOCBPJOI_00576	WP_068652676.1	KLT22114.1	WP_088414005.1

gspD	**K02453**	wLug_00880	WP_022626270.1	KIHBGAEE_00080	LKKDFFFJ_01072			WP_044475426.1	WP_070064591.1	AOCBPJOI_00388	WP_068651130.1	KLT22978.1	
secD	**K03072**	wLug_01337	WP_010962357.1	KIHBGAEE_00723	LKKDFFFJ_01205	wOo_05050	WP_025264030.1	WP_012481842.1	WP_081326513.1	AOCBPJOI_00675	WP_068651145.1	KLT22990.1	WP_088414312.1
secF	**K03074**	wLug_01195	WP_010082185.1	KIHBGAEE_00996	LKKDFFFJ_00421	wOo_05480	WP_014869084.1	WP_007302794.1	WP_070064926.1	AOCBPJOI_00065	WP_068651216.1	KLT22970.1	WP_088415464.1
secE	**K03073**		WP_006279543.1	KIHBGAEE_00215		wOo_01230	WP_025264279.1	WP_012481857.1	WP_081326526.1		WP_068652482.1	KLT23377.1	WP_088415412.1
SecG	**K03075**	wLug_00169	WP_010962615.1	KIHBGAEE_00563	LKKDFFFJ_00681	wOo_06770	WP_025264135.1	WP_007302593.1	WP_070064867.1	AOCBPJOI_01226	WP_068652570.1	KLT22895.1	WP_088415305.1
SecY	**K03076**	wLug_00238	WP_010962747.1	KIHBGAEE_00345	LKKDFFFJ_00184	wOo_07940	WP_025264198.1	WP_007302551.1	WP_070065333.1	AOCBPJOI_01177	WP_068651646.1	KLT23223.1	WP_088414081.1
YajC	**K03210**	wLug_00163	WP_010081840.1	KIHBGAEE_00145	LKKDFFFJ_00398	wOo_02930	WP_014868887.1	WP_006012489.1	WP_070064804.1	AOCBPJOI_01234	WP_068651320.1	KLT21938.1	WP_088415517.1
yidC, spoIIIJ, OXA1, ccfA	**K03217**	wLug_01193	WP_010962455.1	KIHBGAEE_00540	LKKDFFFJ_00419	wOo_05490	WP_025264056.1	WP_007302796.1	WP_070065122.1	AOCBPJOI_00062	WP_068651220.1	KLT22973.1	WP_088415466.1
SecA	**K03070**	wLug_00864	WP_022626296.1	KIHBGAEE_00827	LKKDFFFJ_01073	wOo_01950	WP_025264315.1	WP_012481813.1	WP_070065308.1	AOCBPJOI_00367	WP_068651578.1	KLT22169.1	WP_088414298.1
ftsY	**K03110**	wLug_00135	WP_010962907.1	KIHBGAEE_00816	LKKDFFFJ_00813	wOo_02240	WP_014868835.1	WP_007302619.1	WP_070064642.1	AOCBPJOI_01264	WP_068651517.1	KLT23183.1	WP_088414879.1
SecB	**K03071**	wLug_00346	WP_006279761.1	KIHBGAEE_00558	LKKDFFFJ_00825	wOo_07150	WP_025264149.1	WP_007302467.1	WP_081326532.1	AOCBPJOI_01085	WP_068651781.1	KLT22281.1	WP_088414841.1
ffh	**K03106**	wLug_00408	WP_010963030.1	KIHBGAEE_00671	LKKDFFFJ_00465	wOo_02710	WP_025264366.1	WP_007302411.1	WP_070064671.1	AOCBPJOI_01019	WP_068651896.1	KLT22202.1	WP_088414476.1
TatA	**K03116**	wLug_00946	WP_006013840.1	KIHBGAEE_00689	LKKDFFFJ_00273	wOo_00560	WP_014868718.1	WP_006013840.1	WP_070065314.1	AOCBPJOI_01527	WP_006013840.1	KLT23260.1	WP_006013840.1
TatC	**K03118**	wLug_01040	WP_010962605.1	KIHBGAEE_00513	LKKDFFFJ_00613	wOo_04430	WP_014869000.1	WP_007302699.1	WP_070064595.1	AOCBPJOI_01442	WP_068651390.1	KLT22877.1	WP_088414135.1
virB3, lvhB3	**K03198**	wLug_01246	WP_010962887.1	KIHBGAEE_00002	LKKDFFFJ_00740	wOo_01000	WP_014868750.1	WP_006015054.1	WP_070064713.1	AOCBPJOI_00571	WP_068652414.1	KLT22118.1	WP_011256995.1
virB9, lvhB9	**K03204**	wLug_00146	WP_010082330.1	KIHBGAEE_00032	LKKDFFFJ_00252	wOo_01580	WP_014868799.1	WP_007302273.1	WP_070064991.1	AOCBPJOI_00983	WP_068652081.1	KLT22651.1	WP_088414033.1
		wLug_00440	WP_010962300.1	KIHBGAEE_00108	LKKDFFFJ_00668	wOo_05590	WP_052307037.1	WP_007302610.1	WP_070065046.1	AOCBPJOI_01255	WP_068652572.1	KLT22340.1	WP_088415194.1
virB6, lvhB6	**K03201**	wLug_01241	WP_010962882.1	KIHBGAEE_00004	LKKDFFFJ_00735	wOo_01020	WP_025264268.1	WP_007302028.1	WP_070064715.1	AOCBPJOI_00566	WP_068652418.1	KLT22120.1	WP_088414779.1
		wLug_01242	WP_010962883.1	KIHBGAEE_00005	LKKDFFFJ_00736	wOo_01030	WP_025264269.1	WP_007302029.1	WP_070064717.1	AOCBPJOI_00567	WP_068652421.1	KLT22121.1	WP_088414781.1
		wLug_01243	WP_010962884.1	KIHBGAEE_00006	LKKDFFFJ_00737	wOo_01040	WP_025264271.1	WP_012481863.1	WP_081326504.1	AOCBPJOI_00568	WP_068652423.1	KLT22122.1	WP_088414783.1
		wLug_01244	WP_010962885.1	KIHBGAEE_00007	LKKDFFFJ_00738	wOo_01050		WP_012481864.1	WP_081326505.1	AOCBPJOI_00569	WP_068652678.1	KLT22123.1	
virB8	**K03203**	wLug_00147	WP_010962299.1	KIHBGAEE_00107	LKKDFFFJ_00109	wOo_02890	WP_025264062.1	WP_007302609.1	WP_070064992.1	AOCBPJOI_01254	WP_068651308.1	KLT22652.1	WP_088415178.1
		wLug_01015	WP_010962854.1	KIHBGAEE_00740	LKKDFFFJ_00253	wOo_05600	WP_025264375.1	WP_007302720.1	WP_070065082.1	AOCBPJOI_01467	WP_068651427.1	KLT22664.1	WP_088415388.1
virB10	**K03195**	wLug_00145	WP_010962301.1	KIHBGAEE_00109	LKKDFFFJ_00251	wOo_05580	WP_025264061.1	WP_007302611.1		AOCBPJOI_01256	WP_068651310.1	KLT22650.1	WP_088415176.1
virB4	**K03199**	wLug_01245	WP_010962886.1	KIHBGAEE_00003	LKKDFFFJ_00328	wOo_01010	WP_025264267.1	WP_007302030.1	WP_070064714.1	AOCBPJOI_00570	WP_068652368.1	KLT22566.1	WP_088414785.1
		wLug_01279	WP_022626146.1	KIHBGAEE_00223	LKKDFFFJ_00739	wOo_02070	WP_025264323.1	WP_007302059.1		AOCBPJOI_00604	WP_068652416.1	KLT22119.1	WP_088414887.1
virB11	**K03196**	wLug_00144	WP_010962302.1	KIHBGAEE_00110	LKKDFFFJ_00250	wOo_05570	WP_025264060.1	WP_007302612.1	WP_070064990.1	AOCBPJOI_01257	WP_068651312.1	KLT22649.1	WP_088415174.1
virD4	**K03205**	wLug_00143	WP_010962303.1	KIHBGAEE_00111	LKKDFFFJ_00249	wOo_05560	WP_025264059.1	WP_007302613.1	WP_070064791.1	AOCBPJOI_01258	WP_068651314.1	KLT22648.1	WP_088415172.1
total		28	29	29	28	28	27	29	27	28	29	29	27

K00190 Oxidative phosphorylation

NADH Dehydrogenase

nuoA	**K00330**	wLug_00419	WP_006279576.1	KIHBGAEE_00311	LKKDFFFJ_00783	wOo_05960	WP_014869121.1	WP_012481929.1	WP_070064575.1	AOCBPJOI_01005	WP_068651876.1	KLT21973.1	WP_088414392.1

nuoB	**K00331**	wLug_00420	WP_006279574.1	KIHBGAEE_00310	LKKDFFFJ_00784	wOo_05950	WP_014869120.1	WP_007302257.1	WP_070064574.1	AOCBPJOI_01004	WP_068651874.1	KLT21972.1	WP_088414394.1
nuoC	**K00332**	wLug_00421	WP_006279573.1	KIHBGAEE_00309	LKKDFFFJ_00785	wOo_05940	WP_014869119.1	WP_007302258.1	WP_070064573.1	AOCBPJOI_01003	WP_068651872.1	KLT21971.1	WP_088414396.1
nuoD	**K00333**	wLug_01274	WP_010962676.1	KIHBGAEE_00204	LKKDFFFJ_01067	wOo_01570	WP_025264298.1	WP_007302054.1	WP_070065280.1	AOCBPJOI_00599	WP_068652379.1	KLT22571.1	WP_088414805.1
nuoE	**K00334**	wLug_00717	WP_010962799.1		LKKDFFFJ_01179	wOo_03710	WP_025263945.1	WP_007302017.1	WP_070065307.1	AOCBPJOI_00514	WP_068652438.1	KLT22734.1	WP_088414037.1
nuoF	**K00335**	wLug_00463	WP_010962959.1	KIHBGAEE_00506	LKKDFFFJ_00548	wOo_01750	WP_025264306.1	WP_007302390.1	WP_070065164.1	AOCBPJOI_00963	WP_068651928.1	KLT22322.1	WP_088414617.1
nuoG	**K00336**	wLug_00340	WP_010962403.1	KIHBGAEE_00467	LKKDFFFJ_00264	wOo_00260	WP_025264230.1	WP_007302472.1	WP_070064587.1	AOCBPJOI_01091	WP_068652608.1	KLT22015.1	WP_088414211.1
nuoH	**K00337**	wLug_00339	WP_010962402.1	KIHBGAEE_00466	LKKDFFFJ_00265	wOo_00250	WP_025264229.1	WP_007302473.1	WP_070064586.1	AOCBPJOI_01093	WP_068651773.1	KLT22014.1	WP_088414209.1
nuoI	**K00338**	wLug_00460	WP_022626393.1	KIHBGAEE_00858	LKKDFFFJ_00543	wOo_01720	WP_014868809.1	WP_012481971.1	WP_070065244.1	AOCBPJOI_00966	WP_068652629.1	KLT22326.1	WP_088414741.1
nuoJ	**K00339**	wLug_00477	WP_010962952.1	KIHBGAEE_00261	LKKDFFFJ_00561	wOo_06260	WP_025264101.1	WP_012481965.1	WP_070065103.1	AOCBPJOI_00943	WP_068651951.1	KLT22311.1	WP_088415356.1
nuoK	**K00340**	wLug_00476	WP_010962953.1	KIHBGAEE_00260	LKKDFFFJ_00560	wOo_06250	WP_025264100.1	WP_006012916.1	WP_070065104.1	AOCBPJOI_00944	WP_068651949.1	KLT22312.1	WP_088415354.1
nuoL	**K00341**	wLug_00475	WP_022626390.1	KIHBGAEE_00259	LKKDFFFJ_00559	wOo_06240	WP_025264099.1	WP_012481966.1	WP_070065105.1	AOCBPJOI_00945	WP_068651947.1	KLT22313.1	WP_088415352.1
nuoM	**K00342**	wLug_00474	WP_010962955.1	KIHBGAEE_00258	LKKDFFFJ_00558	wOo_06230	WP_025264098.1	WP_012481967.1	WP_070065106.1	AOCBPJOI_00946	WP_068651945.1	KLT22314.1	WP_088415350.1
nuoN	**K00343**	wLug_00473	WP_010962956.1	KIHBGAEE_00257	LKKDFFFJ_00557	wOo_06220	WP_025264097.1	WP_012481968.1	WP_070065107.1	AOCBPJOI_00947	WP_068651944.1	KLT22315.1	WP_088415348.1
**succinate dehydrogenase**													
sdhC, frdC	**K00241**	wLug_00370	WP_010963112.1	KIHBGAEE_00355	LKKDFFFJ_01176	wOo_07800	WP_025264187.1	WP_012481985.1	WP_070065130.1	AOCBPJOI_01063	WP_068651812.1	KLT22260.1	WP_088415000.1
sdhD, frdD	**K00242**			KIHBGAEE_00356		wOo_07790	WP_025264186.1	WP_012481986.1	WP_070065129.1	AOCBPJOI_01064	WP_068651810.1	KLT22261.1	WP_088414998.1
sdhA, frdA	**K00239**	wLug_00728	WP_010962592.1	KIHBGAEE_00712	LKKDFFFJ_00430	wOo_03910	WP_025263958.1	WP_012481881.1	WP_070064874.1	AOCBPJOI_00503	WP_068652546.1	KLT22451.1	WP_088415258.1
sdhB, frdB	**K00240**	wLug_00705	WP_010082472.1	KIHBGAEE_00474	LKKDFFFJ_01187	wOo_04120	WP_025263973.1	WP_007302009.1	WP_070065230.1	AOCBPJOI_00522	WP_068652446.1	KLT23194.1	WP_088415154.1
**F-type ATPase, prokaryotes and chloroplasts**													
ATPF1A, atpA	**K02111**	wLug_00230	WP_010962741.1	KIHBGAEE_00679	LKKDFFFJ_00177	wOo_06550	WP_025264120.1	WP_007302558.1		AOCBPJOI_01185	WP_068651633.1	KLT23215.1	WP_088414097.1
ATPF1B, atpD	**K02112**	wLug_00269/00278	WP_010962432.1	KIHBGAEE_00118	LKKDFFFJ_00690	wOo_00670	WP_029784320.1	WP_019078549.1	WP_081326572.1	AOCBPJOI_01147	WP_068651694.1	KLT22582.1	WP_088414577.1
ATPF1G, atpG	**K02115**	wLug_00362	WP_010081973.1	KIHBGAEE_00691	LKKDFFFJ_00776	wOo_01350	WP_025264286.1	WP_007302454.1	WP_070064900.1	AOCBPJOI_01071	WP_068651800.1	KLT22267.1	WP_088414563.1
ATPF1D, atpH	**K02113**	wLug_00231	WP_010962742.1	KIHBGAEE_00929	LKKDFFFJ_00178	wOo_06560	WP_025264121.1	WP_007302557.1	WP_070065341.1	AOCBPJOI_01184	WP_068651635.1	KLT23216.1	WP_088414095.1
ATPF1E, atpC	**K02114**	wLug_00268/00277	WP_006279902.1	KIHBGAEE_00117	LKKDFFFJ_00689	wOo_00660	WP_014868725.1	WP_012482002.1	WP_070065353.1	AOCBPJOI_01148	WP_068651692.1	KLT22581.1	WP_088414531.1
ATPF0A, atpB	**K02108**	wLug_01265	WP_010962583.1	KIHBGAEE_00263	LKKDFFFJ_00514	wOo_00400	WP_014868703.1	WP_007302047.1	WP_070064846.1	AOCBPJOI_00590	WP_068652394.1	KLT21774.1	WP_088415450.1
ATPF0B, atpF	**K02109**	wLug_01262	WP_010962584.1	KIHBGAEE_00265	LKKDFFFJ_00516	wOo_00420	WP_014868706.1	WP_007302045.1	WP_070064667.1	AOCBPJOI_00587	WP_068652397.1	KLT21771.1	WP_088415444.1
		wLug_01263	WP_010962585.1	KIHBGAEE_00266	LKKDFFFJ_00517	wOo_00430	WP_025264238.1	WP_007302046.1	WP_081326500.1	AOCBPJOI_00588	WP_068652398.1	KLT21772.1	WP_088415446.1
ATPF0C, atpE	**K02110**	wLug_01264	WP_006014985.1	KIHBGAEE_00264	LKKDFFFJ_00515	wOo_00410	WP_014868704.1	WP_006014985.1	WP_070064666.1	AOCBPJOI_00589	WP_068652396.1	KLT21773.1	WP_088415448.1
**cytochrome c reductase**													

UQCRFS1, RIP1, petA	**K00411**	wLug_00604	WP_010963095.1	KIHBGAEE_00998	LKKDFFFJ_00351	wOo_02510	WP_025264353.1	WP_006014178.1	WP_070064727.1	AOCBPJOI_00901	WP_068652187.1	KLT21674.1	WP_088415224.1
CYTB, petB	**K00412**	wLug_01429	WP_006280242.1	KIHBGAEE_00233	LKKDFFFJ_01264	wOo_05230	WP_025264038.1	WP_007302150.1	WP_070065368.1	AOCBPJOI_00744	WP_068652285.1	KLT22059.1	
CYC1, CYT1, petC	**K00413**	wLug_01428	WP_010963022.1	KIHBGAEE_00234	LKKDFFFJ_01263	wOo_05240	WP_025264039.1	WP_007302151.1	WP_081326574.1	AOCBPJOI_00743	WP_068652287.1	KLT22058.1	WP_088414970.1
**Cytochrome c oxidase**													
COX10, ctaB, cyoE	**K02257**	wLug_00939	WP_010962500.1	KIHBGAEE_00424	LKKDFFFJ_00915	wOo_04400	WP_014868997.1	WP_007302763.1	WP_070065172.1	AOCBPJOI_01519	WP_068651283.1	KLT23266.1	WP_088414113.1
COX11, ctaG	**K02258**	wLug_00583	WP_010082343.1	KIHBGAEE_00053	LKKDFFFJ_00577	wOo_07860	WP_025264192.1	WP_010407017.1	WP_081326502.1	AOCBPJOI_00884	WP_068652016.1	KLT22483.1	WP_088415011.1
COX15, ctaA	**K02259**	wLug_01260	WP_022626285.1	KIHBGAEE_00268	LKKDFFFJ_00519	wOo_00440	WP_044104096.1	WP_012481867.1	WP_070064579.1	AOCBPJOI_00585	WP_068652399.1	KLT21769.1	WP_088415442.1
coxC, ctaE	**K02276**	wLug_00291	WP_006279354.1	KIHBGAEE_01014	LKKDFFFJ_01230	wOo_06840	WP_025264140.1	WP_007302514.1	WP_070065161.1	AOCBPJOI_01137	WP_068652606.1	KLT22623.1	WP_088414542.1
coxA, ctaD	**K02274**	wLug_00938	WP_010962499.1	KIHBGAEE_00423	LKKDFFFJ_00916	wOo_04390	WP_025263989.1	WP_012481731.1	WP_070065171.1	AOCBPJOI_01518	WP_068651281.1	KLT23267.1	WP_088414111.1
coxB, ctaC	**K02275**	wLug_00937	WP_010962498.1	KIHBGAEE_00422	LKKDFFFJ_00917	wOo_04380	WP_014868995.1	WP_007302761.1	WP_070065210.1	AOCBPJOI_01517	WP_068651279.1	KLT23268.1	WP_088414109.1
**Cytochrome bd ubiquinol oxidase**													
cydA	**K00425**		WP_010962801.1		LKKDFFFJ_00721								
cydB	**K00426**		WP_010962802.1		LKKDFFFJ_00720								
total		35	37	35	37	36	36	36	35	36	36	36	35
**K04112 Cell cycle - Caulobacter**													
murG	**K02563**	wLug_00535	WP_007549072.1	KIHBGAEE_00154	LKKDFFFJ_00488	wOo_07340	WP_025264157.1	WP_007302295.1	WP_070064664.1	AOCBPJOI_00839	WP_068652109.1	KLT22560.1	WP_088413973.1
ftsW, spoVE	**K03588**		WP_010962558.1	KIHBGAEE_00981	LKKDFFFJ_01035								
ftsZ	**K03531**	wLug_00702	WP_010962790.1		LKKDFFFJ_01191	wOo_04100	WP_025263971.1	WP_012481860.1		AOCBPJOI_00525	WP_068652440.1	KLT23198.1	WP_088415152.1
ftsQ	**K03589**	wLug_00805	WP_010962363.1	KIHBGAEE_00092	LKKDFFFJ_00972	wOo_01160	WP_025264276.1	WP_007302888.1	WP_081326543.1	AOCBPJOI_00413	WP_068651163.1	KLT23388.1	WP_088413995.1
ftsA	**K03590**	wLug_01470	WP_010962406.1	KIHBGAEE_00778	LKKDFFFJ_01293	wOo_00320	WP_025264234.1	WP_007302316.1	WP_070065058.1	AOCBPJOI_00788	WP_068652140.1	KLT23319.1	WP_088415434.1
PerP	**K06985**	wLug_01194	WP_006279648.1	KIHBGAEE_00995	LKKDFFFJ_00420			WP_007302795.1	WP_081326545.1	AOCBPJOI_00063	WP_068651218.1	KLT22972.1	WP_088415478.1
pleC	**K07716**	wLug_01380	WP_010963156.1	KIHBGAEE_00156	LKKDFFFJ_00877	wOo_04190<br>wOo_04200		WP_010403276.1	WP_070064863.1	AOCBPJOI_00699	WP_068652238.1	KLT23426.1	WP_088414629.1
rseP	**K11749**	wLug_00401	WP_010963034.1	KIHBGAEE_00097	LKKDFFFJ_00459	wOo_02770	WP_025264370.1	WP_007302416.1	WP_070064948.1	AOCBPJOI_01026	WP_068651909.1	KLT22209.1	WP_088414238.1
pleD	**K02488**	wLug_00824	WP_010962441.1	KIHBGAEE_00208	LKKDFFFJ_00231	wOo_05330		WP_007302914.1	WP_070065194.1	AOCBPJOI_00435	WP_068651584.1	KLT22180.1	WP_088415303.1
clpX, CLPX	**K03544**	wLug_01206	WP_010962512.1	KIHBGAEE_00148	LKKDFFFJ_00482	wOo_07280	WP_014869236.1	WP_007302000.1	WP_070065016.1	AOCBPJOI_00531	WP_068652468.1	KLT23204.1	WP_088413963.1
clpP, CLPP	**K01358**	wLug_01207	WP_010962513.1	KIHBGAEE_00149	LKKDFFFJ_00483	wOo_07290	WP_014869237.1	WP_007301999.1	WP_070065015.1	AOCBPJOI_00532	WP_068652682.1	KLT23205.1	WP_088413965.1
dnaA	**K02313**	wLug_01196	WP_010962298.1	KIHBGAEE_00104	LKKDFFFJ_00257	wOo_07410	WP_025264162.1	WP_012481713.1		AOCBPJOI_00066	WP_068651214.1	KLT22969.1	WP_088415184.1
dnaB	**K02314**	wLug_00809	WP_010962532.1	KIHBGAEE_00062	LKKDFFFJ_00605	wOo_04630	WP_025264003.1	WP_007302722.1	WP_070065072.1	AOCBPJOI_00453	WP_068651429.1	KLT21624.1	WP_088414131.1
lon	**K01338**	wLug_01205	WP_010962511.1	KIHBGAEE_00147	LKKDFFFJ_00481	wOo_07270	WP_025264155.1	WP_007302001.1	WP_081326530.1	AOCBPJOI_00530	WP_068652466.1	KLT23203.1	WP_088413961.1
total		13	14	13	14	13	10	13	11	13	13	13	13
**K03440 Homologous recombination**													
recJ	**K07462**	wLug_01272	WP_010962506.1	KIHBGAEE_00733	LKKDFFFJ_01218	wOo_00900	WP_025264262.1	WP_007302053.1	WP_070065272.1	AOCBPJOI_00597	WP_068652380.1	KLT21655.1	WP_088414803.1

ssb	**K03111**	wLug_00590	WP_010082448.1	KIHBGAEE_00076	LKKDFFFJ_00064	wOo_06450	WP_025264114.1	WP_006014313.1	WP_070065313.1	AOCBPJOI_00891	WP_068652009.1	KLT22487.1	WP_088415491.1
recA	**K03553**	wLug_00496	WP_010963008.1	KIHBGAEE_00401	LKKDFFFJ_01243			WP_007302323.1	WP_070065226.1	AOCBPJOI_00802	WP_068652151.1	KLT21684.1	WP_088415100.1
recF	**K03629**	wLug_00532	WP_010963158.1	KIHBGAEE_00158	LKKDFFFJ_00879			WP_007302298.1	WP_070065277.1	AOCBPJOI_00836	WP_068652113.1	KLT23021.1	WP_088414633.1
recO	**K03584**	wLug_00825	WP_010962439.1	KIHBGAEE_01000	LKKDFFFJ_00230			WP_007302913.1	WP_070065047.1	AOCBPJOI_00434	WP_068651582.1	KLT22179.1	WP_088414753.1
recR	**K06187**	wLug_01391	WP_010963080.1	KIHBGAEE_00814	LKKDFFFJ_00335			WP_012481907.1	WP_070065239.1	AOCBPJOI_00710	WP_068651981.1	KLT21947.1	WP_088414482.1
polA	**K02335**	wLug_00314	WP_010962979.1	KIHBGAEE_00532	LKKDFFFJ_00523	wOo_04910	WP_025264018.1	WP_007302475.1	WP_070065099.1	AOCBPJOI_01116	WP_068651731.1	KLT22073.1	WP_088414189.1
dnaE	**K02337**	wLug_01301	WP_010962830.1	KIHBGAEE_00639	LKKDFFFJ_00988	wOo_07050	WP_025264145.1	WP_012481876.1	WP_070065008.1	AOCBPJOI_00632	WP_068652338.1	KLT21703.1	WP_088414496.1
ruvA	**K03550**	wLug_00483	WP_010082382.1	KIHBGAEE_00609	LKKDFFFJ_00660	wOo_03590		WP_007302375.1	WP_070064943.1	AOCBPJOI_00938	WP_068651959.1	KLT22299.1	WP_088414412.1
ruvB	**K03551**	wLug_00484	WP_010963048.1	KIHBGAEE_00608	LKKDFFFJ_00661			WP_007302374.1		AOCBPJOI_00937	WP_068651961.1	KLT22298.1	WP_088414414.1
ruvC	**K01159**	wLug_00290	WP_010962392.1	KIHBGAEE_01013	LKKDFFFJ_01231			WP_012481999.1	WP_070065162.1	AOCBPJOI_01138	WP_068651707.1	KLT22622.1	WP_088414540.1
recG	**K03655**	wLug_00871	WP_010962860.1	KIHBGAEE_00828	LKKDFFFJ_00117				WP_070065345.1	AOCBPJOI_00380	WP_068651133.1	KLT22656.1	WP_088415374.1
total		12	12	12	12	5	4	11	11	12	12	12	12
**K02010 ABC transporters**													
**iron(III) transport system**													
AfuA	**K02012**	wLug_00734	WP_010082338.1	KIHBGAEE_00973	LKKDFFFJ_00806	wOo_03090	WP_025264384.1	WP_007302093.1	WP_070064782.1	AOCBPJOI_00497	WP_068651071.1	KLT22444.1	WP_088414251.1
AfuB	**K02011**	wLug_01445	WP_010963057.1	KIHBGAEE_01024	LKKDFFFJ_00310	wOo_02620	WP_025264361.1	WP_007302135.1	WP_070064682.1	AOCBPJOI_00763	WP_068652267.1	KLT23129.1	WP_088414456.1
AfuC	**K02010**	wLug_00947	WP_006280146.1	KIHBGAEE_00690	LKKDFFFJ_00272	wOo_00550	WP_014868717.1	WP_007302769.1	WP_081326558.1	AOCBPJOI_01528	WP_068651293.1	KLT23259.1	WP_088414699.1
**phosphate transport system**													
PstS	**K02040**	wLug_00157	WP_010962546.1	KIHBGAEE_00716	LKKDFFFJ_01127	wOo_07870	WP_025264193.1	WP_012482020.1	WP_070064753.1	AOCBPJOI_01240	WP_068651328.1	KLT23413.1	WP_088414900.1
PstC	**K02037**	wLug_00270/00279	WP_038228098.1	KIHBGAEE_00119	LKKDFFFJ_00691	wOo_00680	WP_014868727.1	WP_012482001.1	WP_070065351.1	AOCBPJOI_01146	WP_068651696.1	KLT22583.1	WP_088414579.1
PstA	**K02038**	wLug_00551	WP_010962988.1	KIHBGAEE_00523	LKKDFFFJ_00644	wOo_03250	WP_025263916.1	WP_007302279.1	WP_070064618.1	AOCBPJOI_00854	WP_068652095.1	KLT21636.1	WP_088414376.1
PstB	**K02036**	wLug_00970	WP_010962543.1	KIHBGAEE_00017	LKKDFFFJ_00592	wOo_06690	WP_025264129.1	WP_012481738.1	WP_070064616.1	AOCBPJOI_00046	WP_068651249.1	KLT21956.1	WP_088415291.1
**lipoprotein-releasing system**													
lolC_E	**K09808**	wLug_00365	WP_010963118.1	KIHBGAEE_00069	LKKDFFFJ_01213	wOo_07770	WP_025264184.1	WP_007302450.1	WP_070065102.1	AOCBPJOI_01067	WP_068651806.1	KLT22264.1	WP_088414992.1
LolD	**K09810**	wLug_00578	WP_010962776.1	KIHBGAEE_00518	LKKDFFFJ_01113	wOo_06090	WP_025264089.1	WP_007302231.1	WP_070065030.1	AOCBPJOI_00879	WP_068652022.1	KLT22478.1	WP_088415319.1
**heme exporter**													
CcmC	**K02195**	wLug_00882	WP_010962525.1	KIHBGAEE_00478	LKKDFFFJ_01289	wOo_06290	WP_025264103.1	WP_007301928.1	WP_070064705.1	AOCBPJOI_00390	WP_068652562.1	KLT22804.1	WP_088415372.1
CcmB	**K02194**	wLug_01067	WP_010963039.1	KIHBGAEE_00030	LKKDFFFJ_00453	wOo_00110	WP_025264222.1	WP_007302672.1	WP_081326557.1	AOCBPJOI_01411	WP_068651380.1	KLT23307.1	WP_088414007.1
CcmA	**K02193**	wLug_00028	WP_007548816.1	KIHBGAEE_00657	LKKDFFFJ_00449	wOo_01660	WP_014868803.1	WP_007302587.1	WP_070065371.1	AOCBPJOI_01221	WP_068651124.1	KLT22185.1	WP_088414358.1
**phospholipid transport system**													
MlaC	**K07323**	wLug_00510		KIHBGAEE_00170				WP_019236836.1	WP_070064582.1	AOCBPJOI_00815	WP_068652648.1	KLT21698.1	WP_088414604.1

MlaD	**K02067**	wLug_00447	WP_010082331.1	KIHBGAEE_00159	LKKDFFFJ_00667			WP_007302404.1	WP_070064774.1	AOCBPJOI_00979	WP_068652051.1	KLT22339.1	WP_088415315.1
MlaE	**K02066**	wLug_00410	WP_010962968.1	KIHBGAEE_00252	LKKDFFFJ_00536			WP_012481974.1	WP_070064751.1	AOCBPJOI_01017	WP_068651892.1	KLT21982.1	WP_088415340.1
MlaF	**K02065**	wLug_00409	WP_006279859.1	KIHBGAEE_00253	LKKDFFFJ_00535			WP_012481975.1	WP_070064752.1	AOCBPJOI_01018	WP_068651894.1	KLT21983.1	WP_088415342.1
**zinc transport system**													
ZnuA	**K09815**	wLug_01030	WP_010962934.1	KIHBGAEE_00230	LKKDFFFJ_00803	wOo_05010	WP_025264026.1	WP_007302707.1	WP_070064643.1	AOCBPJOI_01453	WP_068651408.1	KLT23347.1	WP_088414171.1
ZnuB	**K09816**	wLug_01052	WP_010962536.1	KIHBGAEE_00067	LKKDFFFJ_00597	wOo_06730	WP_025264131.1	WP_007302687.1	WP_070064597.1	AOCBPJOI_01428	WP_068651358.1	KLT23066.1	WP_088415284.1
ZnuC	**K09817**	wLug_01031	WP_022626380.1	KIHBGAEE_00231	LKKDFFFJ_00804	wOo_05020	WP_025264027.1	WP_007302706.1	WP_070064644.1	AOCBPJOI_01452	WP_068651406.1	KLT23348.1	WP_088414169.1
**biotin transport system**													
BioY	**K03523**	wLug_00609	WP_010963099.1	KIHBGAEE_00792	LKKDFFFJ_00356			WP_007302344.1	WP_070065036.1	AOCBPJOI_00906	WP_068652195.1	KLT21678.1	WP_088414687.1
total		20	19	20	19	15	15	20	20	20	20	20	20
**K00564 Glycerophospholipid metabolism**													
araM, egsA	**K00096**	wLug_01306	WP_010962834.1	KIHBGAEE_00799	LKKDFFFJ_00993	wOo_08290		WP_007302109.1	WP_070064937.1	AOCBPJOI_00640	WP_068652330.1	KLT21708.1	WP_088413989.1
gpsA	**K00057**	wLug_00709	WP_010962796.1	KIHBGAEE_00470	LKKDFFFJ_01183	wOo_04160	WP_025263977.1	WP_007302013.1	WP_070064625.1	AOCBPJOI_00518	WP_068652454.1	KLT23190.1	WP_088415162.1
plsY	**K08591**	wLug_00324	WP_010963004.1	KIHBGAEE_00406	LKKDFFFJ_00923	wOo_03620	WP_014868937.1	WP_007302484.1	WP_070064740.1	AOCBPJOI_01107	WP_068651749.1	KLT22083.1	WP_088415108.1
plsC	**K00655**	wLug_00631	WP_010963063.1	KIHBGAEE_00907	LKKDFFFJ_00301	wOo_07210	WP_025264153.1	WP_012481964.1	WP_070064552.1	AOCBPJOI_00931	WP_068652226.1	KLT22290.1	WP_088414715.1
dgkA, DGK	**K00901**	wLug_00522	WP_007549043.1		LKKDFFFJ_00321			WP_007302306.1		AOCBPJOI_00826		KLT21600.1	
pld	**K17717**											KLT22761.1	
E2.7.7.41, CDS1, CDS2, cdsA	**K00981**	wLug_00201	WP_010082212.1	KIHBGAEE_00369	LKKDFFFJ_00390	wOo_07610	WP_025264174.1	WP_007302837.1	WP_070064932.1	AOCBPJOI_00325	WP_068651348.1	KLT22999.1	WP_088414512.1
CHO1, pssA	**K17103**	wLug_00320	WP_010963007.1	KIHBGAEE_00403	LKKDFFFJ_00926	wOo_03640	WP_014868939.1	WP_007302481.1	WP_070064851.1	AOCBPJOI_01110	WP_068651741.1	KLT22080.1	WP_088415104.1
psd, PISD	**K01613**	wLug_00319	WP_007548750.1	KIHBGAEE_00402	LKKDFFFJ_00927	wOo_03650	WP_025263942.1	WP_007302480.1	WP_070064737.1	AOCBPJOI_01111	WP_068651739.1	KLT22079.1	WP_088415102.1
pgsA, PGS1	**K00995**	wLug_00216	WP_010962629.1	KIHBGAEE_00497	LKKDFFFJ_01054	wOo_05190	WP_014869058.1	WP_006012406.1	WP_070064728.1	AOCBPJOI_01202	WP_068651615.1	KLT22903.1	WP_088414703.1
pgpA	**K01095**	wLug_00708	WP_010962795.1	KIHBGAEE_00471	LKKDFFFJ_01184	wOo_04150	WP_025263976.1	WP_012481861.1	WP_081326496.1	AOCBPJOI_00519	WP_068652452.1	KLT23191.1	WP_088415160.1
total		10	10	9	10	9	8	10	9	10	9	11	9
**K00860 Porphyrin and chlorophyll metabolism**													
ALAS	**K00643**	wLug_01386	WP_010963153.1	KIHBGAEE_00810	LKKDFFFJ_00872	wOo_04240	WP_025263980.1	WP_019078653.1	WP_070065211.1	AOCBPJOI_00706	WP_068651971.1	KLT21952.1	WP_088414619.1
EARS, gltX	**K01885**	wLug_01049	WP_010962590.1	KIHBGAEE_00271	LKKDFFFJ_00061	wOo_00460	WP_025263961.1	WP_007302040.1	WP_070064545.1	AOCBPJOI_00582	WP_068651437.1	KLT23069.1	WP_088415252.1
		wLug_01257	WP_010962828.1	KIHBGAEE_00499	LKKDFFFJ_00522	wOo_03950	WP_025264241.1	WP_007302690.1	WP_070065196.1	AOCBPJOI_01432	WP_068652401.1	KLT21766.1	
hemB, ALAD	**K01698**	wLug_00338	WP_010962401.1	KIHBGAEE_00934	LKKDFFFJ_00266	wOo_06430	WP_014869164.1	WP_007302474.1	WP_070064982.1	AOCBPJOI_01094	WP_068651772.1	KLT22013.1	WP_088414205.1
hemC, HMBS	**K01749**	wLug_01340	WP_010962666.1	KIHBGAEE_00701	LKKDFFFJ_00402	wOo_05060	WP_014869049.1	WP_007301916.1	WP_070064660.1	AOCBPJOI_00677	WP_068651147.1	KLT22988.1	
hemD, UROS	**K01719**	wLug_00311	WP_010962976.1	KIHBGAEE_00921	LKKDFFFJ_00526	wOo_04870	WP_025264015.1	WP_007302498.1	WP_070065369.1	AOCBPJOI_01119	WP_068651725.1	KLT22591.1	WP_088414725.1
hemE, UROD	**K01599**	wLug_00559	WP_010082013.1	KIHBGAEE_00457	LKKDFFFJ_00653	wOo_03330	WP_025263922.1	WP_007302249.1	WP_081326497.1	AOCBPJOI_00862	WP_068652047.1	KLT21781.1	WP_088414912.1
CPOX, hemF	**K00228**	wLug_00621	WP_010963106.1	KIHBGAEE_00277	LKKDFFFJ_01015	wOo_04530	WP_025263996.1	WP_007302354.1	WP_070064763.1	AOCBPJOI_00917	WP_068652209.1	KLT23331.1	WP_088414568.1

hemJ	**K08973**	wLug_00935	WP_010962574.1	KIHBGAEE_00014	LKKDFFFJ_00443	wOo_06670	WP_014869184.1	WP_012481732.1	WP_070064778.1	AOCBPJOI_01515	WP_068651275.1	KLT23270.1	WP_088414199.1
hemH, FECH	**K01772**	wLug_01412	WP_010082099.1	KIHBGAEE_00227	LKKDFFFJ_00338	wOo_04970	WP_025264022.1	WP_007302167.1	WP_070065309.1	AOCBPJOI_00727	WP_068652308.1	KLT22042.1	WP_088414177.1
bfr	**K03594**	wLug_01083	WP_010081932.1	KIHBGAEE_00410	LKKDFFFJ_01106			WP_007302660.1	WP_070065124.1	AOCBPJOI_01394	WP_068651546.1	KLT23053.1	
COX10, ctaB, cyoE	**K02257**	wLug_00939	WP_010962500.1	KIHBGAEE_00424	LKKDFFFJ_00915	wOo_04400	WP_014868997.1	WP_007302763.1	WP_070065172.1	AOCBPJOI_01519	WP_068651283.1	KLT23266.1	WP_088414113.1
COX15, ctaA	**K02259**	wLug_01260	WP_022626285.1	KIHBGAEE_00268	LKKDFFFJ_00519	wOo_00440	WP_044104096.1	WP_012481867.1	WP_070064579.1	AOCBPJOI_00585	WP_068652399.1	KLT21769.1	WP_088415442.1
total		13	13	13	13	12	12	13	13	13	13	13	10
**K00230 Purine metabolism**													
PRPS, prsA	**K00948**	wLug_01099	WP_010962322.1	KIHBGAEE_00084	LKKDFFFJ_00861	wOo_02980	WP_025264378.1	WP_007302646.1	WP_070065204.1	AOCBPJOI_01742	WP_068651566.1	KLT22855.1	WP_088414771.1
purF, PPAT	**K00764**	wLug_00639	WP_010082377.1	KIHBGAEE_00890	LKKDFFFJ_00663	wOo_05680	WP_025264066.1	WP_007302311.1	WP_070064738.1	AOCBPJOI_00796	WP_068652134.1	KLT22724.1	WP_088414418.1
purD	**K01945**	wLug_01228	WP_010962317.1	KIHBGAEE_00879	LKKDFFFJ_00467	wOo_03440	WP_025263931.1	WP_007301981.1	WP_070064735.1	AOCBPJOI_00553	WP_068652494.1	KLT23367.1	WP_088415460.1
purN	**K11175**	wLug_00772	WP_010082490.1	KIHBGAEE_00759	LKKDFFFJ_00075	wOo_03490	WP_014868928.1	WP_007301943.1	WP_070064652.1	AOCBPJOI_00462	WP_068651109.1	KLT21725.1	WP_088415116.1
purQ	**K23265**	wLug_00550	WP_006279487.1	KIHBGAEE_00522	LKKDFFFJ_00643	wOo_03240	WP_025263915.1	WP_007302280.1	WP_070064617.1	AOCBPJOI_00853	WP_068652097.1	KLT21637.1	WP_088414378.1
purSL	**K23270**	wLug_01091	WP_010962921.1	KIHBGAEE_00537	LKKDFFFJ_00818	wOo_05650	WP_025264064.1	WP_007302652.1	WP_070065056.1	AOCBPJOI_01749	WP_068651555.1	KLT22861.1	WP_088414952.1
purM	**K01933**	wLug_00555	WP_010962991.1	KIHBGAEE_00527	LKKDFFFJ_00649	wOo_03290	WP_025263919.1	WP_007302275.1	WP_070065241.1	AOCBPJOI_00858	WP_068652087.1	KLT21632.1	WP_088414368.1
purE	**K01588**	wLug_00383	WP_010963167.1	KIHBGAEE_00644	LKKDFFFJ_00895	wOo_01370	WP_025264288.1	WP_007302433.1	WP_070064541.1	AOCBPJOI_01047	WP_068651834.1	KLT22223.1	WP_088415206.1
purC	**K01923**	wLug_00554	WP_010081959.1	KIHBGAEE_00526	LKKDFFFJ_00648	wOo_03280		WP_007302276.1	WP_070064620.1	AOCBPJOI_00857	WP_068652089.1	KLT21633.1	WP_088414370.1
purB, ADSL	**K01756**		WP_010962833.1					WP_007302075.1	WP_070065011.1		WP_068652332.1	KLT21707.1	WP_088414504.1
purH	**K00602**	wLug_00750	WP_010962893.1	KIHBGAEE_00860	LKKDFFFJ_00747	wOo_00960	WP_025264266.1	WP_007301976.1	WP_070064683.1	AOCBPJOI_00480	WP_068651086.1	KLT23091.1	WP_088414360.1
ndk, NME	**K00940**	wLug_01414	WP_010082097.1	KIHBGAEE_00229	LKKDFFFJ_00336	wOo_05000	WP_014869043.1	WP_007302165.1	WP_070065312.1	AOCBPJOI_00729	WP_068652306.1	KLT22044.1	WP_088414173.1
purA, ADSS	**K01939**	wLug_00999	WP_010962523.1	KIHBGAEE_00894	LKKDFFFJ_01284	wOo_07430	WP_025264164.1	WP_007302822.1	WP_070064736.1	AOCBPJOI_01549	WP_068651480.1	KLT21964.1	WP_088415190.1
gmk	**K00942**	wLug_00730	WP_022626283.1	KIHBGAEE_00383	LKKDFFFJ_00428	wOo_00130	WP_025264223.1	WP_007302097.1	WP_070064629.1	AOCBPJOI_00501	WP_068652550.1	KLT22449.1	WP_088414011.1
dgt	**K01129**	wLug_00576	WP_010962778.1	KIHBGAEE_00520	LKKDFFFJ_01111	wOo_06110	WP_014869135.1	WP_007302233.1	WP_070065029.1	AOCBPJOI_00877	WP_068652026.1	KLT22476.1	WP_088415323.1
surE	**K03787**	wLug_01437	WP_010963027.1	KIHBGAEE_00202	LKKDFFFJ_01269	wOo_05320	WP_014869070.1	WP_007302143.1	WP_070065354.1	AOCBPJOI_00754	WP_068652278.1	KLT23120.1	WP_088413957.1
nrdA, nrdE	**K00525**	wLug_00288	WP_006280151.1	KIHBGAEE_00124	LKKDFFFJ_00697	wOo_00740	WP_025264253.1	WP_007302519.1	WP_070065300.1	AOCBPJOI_01140	WP_068651703.1	KLT22587.1	WP_088414537.1
nrdB, nrdF	**K00526**	wLug_00828	WP_006280119.1	KIHBGAEE_00132	LKKDFFFJ_00837	wOo_01930	WP_014868819.1	WP_007302810.1	WP_070064921.1	AOCBPJOI_00431	WP_068651462.1	KLT22176.1	WP_011256466.1
guaA, GMPS	**K01951**	wLug_00879	WP_010962427.1	KIHBGAEE_01016	LKKDFFFJ_01206	wOo_03460	WP_029784303.1	WP_007301932.1	WP_070065298.1	AOCBPJOI_00387	WP_068651129.1	KLT23036.1	WP_088415248.1
purK	**K01589**	wLug_01443	WP_010963059.1		LKKDFFFJ_00308	wOo_02640	WP_014868861.1	WP_029237555.1	WP_070064543.1	AOCBPJOI_00761	WP_068652650.1	KLT23127.1	WP_088414460.1
IMPDH, guaB	**K00088**	wLug_00798	WP_022626203.1	KIHBGAEE_00938	LKKDFFFJ_00979	wOo_00290	WP_025264231.1	WP_012481851.1	WP_070064799.1	AOCBPJOI_00404	WP_068652508.1	KLT23398.1	WP_088414675.1
total		20	21	19	20	20	19	21	21	20	21	21	21
**K00240 Pyrimidine metabolism**													
carB, CPA2	**K01955**	wLug_00493	WP_010963164.1	KIHBGAEE_00352	LKKDFFFJ_00883	wOo_07690	WP_025264179.1	WP_007302307.1	WP_070065304.1	AOCBPJOI_00801	WP_068652125.1	KLT23284.1	WP_088414947.1
carA, CPA1	**K01956**	wLug_00262	WP_010962760.1	KIHBGAEE_00189	LKKDFFFJ_00130	wOo_08180	WP_025264206.1	WP_007302530.1		AOCBPJOI_01153	WP_068651684.1	KLT23245.1	WP_088415242.1

pyrB, PYR2	**K00609**	wLug_00733	WP_006280416.1	KIHBGAEE_00640	LKKDFFFJ_00807	wOo_03080	WP_025264383.1	WP_007302094.1	WP_070064538.1	AOCBPJOI_00498	WP_068651070.1	KLT22445.1	WP_088414342.1
URA4, pyrC	**K01465**	wLug_00813	WP_022626228.1	KIHBGAEE_00659	LKKDFFFJ_00240	wOo_03930	WP_025263960.1	WP_007302727.1	WP_070065159.1	AOCBPJOI_00447	WP_068651447.1	KLT21621.1	WP_088415254.1
DHODH, pyrD	**K00254**	wLug_00351	WP_010082053.1	KIHBGAEE_00832	LKKDFFFJ_00768	wOo_03820	WP_025263953.1	WP_007302462.1	WP_070064854.1	AOCBPJOI_01080	WP_068651786.1	KLT22275.1	WP_088414763.1
pyrE	**K00762**	wLug_01343	WP_010962448.1	KIHBGAEE_00725	LKKDFFFJ_00238	wOo_05040	WP_025264029.1	WP_007301912.1	WP_070065080.1	AOCBPJOI_00680	WP_068651153.1	KLT22985.1	WP_088414306.1
pyrF	**K01591**	wLug_00975	WP_010082622.1		LKKDFFFJ_00606	wOo_04540	WP_025263997.1	WP_012481735.1	WP_081326559.1	AOCBPJOI_01499	WP_068651246.1	KLT22923.1	WP_088415120.1
surE	**K03787**	wLug_01437	WP_010963027.1	KIHBGAEE_00202	LKKDFFFJ_01269	wOo_05320	WP_014869070.1	WP_007302143.1	WP_070065354.1	AOCBPJOI_00754	WP_068652278.1	KLT23120.1	WP_088413957.1
pyrH	**K09903**	wLug_00159	WP_010962659.1	KIHBGAEE_00366	LKKDFFFJ_00394	wOo_07650	WP_025264176.1	WP_010407219.1	WP_070064929.1	AOCBPJOI_01238	WP_068651324.1	KLT21941.1	WP_088414518.1
ndk, NME	**K00940**	wLug_01414	WP_010082097.1	KIHBGAEE_00229	LKKDFFFJ_00336	wOo_05000	WP_014869043.1	WP_007302165.1	WP_070065312.1	AOCBPJOI_00729	WP_068652306.1	KLT22044.1	WP_088414173.1
dcd	**K01494**	wLug_00154	WP_006280485.1	KIHBGAEE_01018	LKKDFFFJ_01124	wOo_04600	WP_014869015.1	WP_007302605.1	WP_070065153.1	AOCBPJOI_01243	WP_068651304.1	KLT22825.1	WP_088414749.1
pyrG, CTPS	**K01937**	wLug_00170	WP_010962616.1	KIHBGAEE_00564	LKKDFFFJ_00680	wOo_06780	WP_014869194.1	WP_007302592.1	WP_070064887.1	AOCBPJOI_01225	WP_068651299.1	KLT22894.1	WP_088415278.1
ndk, NME	**K00940**	wLug_01414	WP_010082097.1	KIHBGAEE_00229	LKKDFFFJ_00336	wOo_05000	WP_014869043.1	WP_007302165.1	WP_070065312.1	AOCBPJOI_00729	WP_068652306.1	KLT22044.1	WP_088414173.1
E1.17.4.1A, nrdA, nrdE	**K00525**	wLug_00288	WP_006280151.1	KIHBGAEE_00124	LKKDFFFJ_00697	wOo_00740	WP_025264253.1	WP_007302519.1	WP_070065300.1	AOCBPJOI_01140	WP_068651703.1	KLT22587.1	WP_088414537.1
E1.17.4.1B, nrdB, nrdF	**K00526**	wLug_00828	WP_006280119.1	KIHBGAEE_00132	LKKDFFFJ_00837	wOo_01930	WP_014868819.1	WP_007302810.1	WP_070064921.1	AOCBPJOI_00431	WP_068651462.1	KLT22176.1	WP_011256466.1
dcd	**K01494**	wLug_00154	WP_006280485.1	KIHBGAEE_01018	LKKDFFFJ_01124	wOo_04600	WP_014869015.1	WP_007302605.1	WP_070065153.1	AOCBPJOI_01243	WP_068651304.1	KLT22825.1	WP_088414749.1
dut, DUT	**K01520**	wLug_01189	WP_006279458.1	KIHBGAEE_00976	LKKDFFFJ_00415	wOo_04050	WP_044104110.1	WP_007302799.1	WP_070065206.1	AOCBPJOI_00058	WP_068651231.1	KLT22100.1	WP_088414591.1
tmk, DTYMK	**K00943**	wLug_00528	WP_038227975.1	KIHBGAEE_00414	LKKDFFFJ_01101	wOo_02550	WP_025264356.1	WP_007302302.1	WP_070064942.1	AOCBPJOI_00832	WP_068652121.1	KLT23017.1	WP_088414817.1
thyX, thy1	**K03465**	wLug_01402	WP_010082033.1	KIHBGAEE_00664	LKKDFFFJ_00348	wOo_02430	WP_025264349.1	WP_007302176.1	WP_070064593.1	AOCBPJOI_00717	WP_068652325.1	KLT22031.1	WP_088414799.1
total		19	19	18	19	19	19	19	18	19	19	19	19
**K00750 Pyridoxine metabolism**													
pdxH, PNPO	**K00275**	wLug_00547	WP_022626142.1	KIHBGAEE_00288	LKKDFFFJ_01149	wOo_01610	WP_014868800.1	WP_006014087.1	WP_070065260.1	AOCBPJOI_00851	WP_068652098.1	KLT21606.1	WP_088414027.1
pdxJ	**K03474**	wLug_01255	WP_006279877.1	KIHBGAEE_00023	LKKDFFFJ_01169	wOo_00050		WP_007302038.1	WP_070065135.1	AOCBPJOI_00580	WP_068652403.1	KLT22110.1	WP_088413997.1
total		2	2	2	2	2	1	2	2	2	2	2	2

Table S4. Summary of BUSCOs analyses of Wolbachia and filarial nematodes complete genomes and draft genomes. Dataset used for the analysis was a proteobacteria dataset containing 1520 species representing 221 BUSCOs for *Wolbachia* and a nematodes dataset containing 8 species representing 982 BUSCOs for the nematodes. Abbreviation: "C": Complete BUSCOs; "S": Complete and single-copy BUSCOs; "D": Complete and duplicated BUSCOs (D); "F": Fragmented BUSCOs; "M": Missing BUSCOs.

			C		S		D		F		M		Total
**organism**	**species/strain**	**NCBI accession**	**n**	**%**	**n**	**%**	**n**	**%**	**n**	**%**	**n**	**%**	
	wLsig	**CP046577**	168	76.0	167	75.6	1	0.5	8	3.6	45	20	221
	wDimm	**CP046578**	168	76.0	168	76.0	0	0.0	8	3.6	45	20.4	221
	wCtub	**CP046579**	171	77.4	171	77.4	0	0.0	6	2.7	44	19.9	221
	wDcau	**CP046580**	162	73.4	161	72.9	1	0.5	6	2.7	53	23.9	221
	wLbra	**WQMO00000000**	169	76.5	168	76.0	1	0.5	5	2.3	47	21.2	221
	wMhie	**WQMP00000000**	167	75.6	166	75.1	1	0.5	5	2.3	49	22.1	221
	wBm	NC_006833	175	79.2	175	79.2	0	0.0	4	1.8	41	19.0	221
	wBp	CP050521	178	81.3	178	81.3	0	0.0	7	3.2	34	15.5	219
	wCau	CP041215	180	81.4	180	81.4	0	0.0	3	1.4	38	17.2	221
Wolbachia	wCfeJ	CP051157	185	84.5	184	84.0	1	0.5	3	1.4	31	14.1	219
	wCfeT	CP051156	184	84.1	183	83.6	1	0.5	4	1.8	31	14.1	219
	wCle	AP013028	177	80.1	177	80.1	0	0.0	9	4.1	35	15.8	221
	wFol	CP015510	179	81.0	179	81.0	0	0.0	4	1.8	38	17.2	221
	wMel	NC_002978	180	81.4	180	81.4	0	0.0	2	0.9	39	17.7	221
	wOo	NC_018267	165	74.7	165	74.7	0	0.0	6	2.7	50	22.6	221
	wOv	HG810405	166	75.1	166	75.1	0	0.0	4	1.8	51	23.1	221
	wPel	NC_010981	178	80.5	178	80.5	0	0.0	5	2.3	38	17.2	221
	wPpe	MJMG01000000	161	72.9	160	72.4	1	0.5	12	5.4	48	21.7	221
	wTpre	CM003641	180	81.4	180	81.4	0	0.0	4	1.8	37	16.8	221

	wNfla	LYUW00000000	179	81.0	177	80.1	2	0.9	4	1.8	38	17.2	221
	wLug	MUIY01000000	179	81.0	177	80.1	2	0.9	4	1.8	38	17.2	221
	wstri	MUIX00000000	179	81.0	179	81.0	0	0.0	4	1.8	38	17.2	221
	wVulC	ALWU00000000	180	81.5	179	81.0	1	0.5	4	1.8	37	16.7	221
Nematode	B. malayi	AAQA00000000	860	87.5	842	86	18	1.8	57	5.8	65	6.7	982
	B. pahangi	JRWH00000000	827	84.2	817	83	10	1.0	76	7.7	79	8.0	982
	C. tuberocauda	**JABVXU000000000**	939	95.6	935	95	4	0.4	31	3.2	12	1.2	982
	D. immitis	**JABVXT000000000**	932	94.9	926	94	6	0.6	30	3.1	20	2.0	982
	D. caudispina	**JABVXV000000000**	959	97.7	956	97.4	3	0.3	18	1.8	5	0.5	982
	L. brasiliensis	**JABVXR000000000**	917	93.4	911	92.8	6	0.6	46	4.7	19	1.9	982
	L. sigmodontis	**JABVXW000000000**	924	94	919	94	5	1	40	4	18	2	982
	M. hiepei	**JABVXS000000000**	849	86	789	80	60	6	73	7	60	6	982
	O. ochengi	CAWC010000000	841	85.6	836	85	5	0.5	93	9.5	48	4.9	982
	O. volvulus	CBVM000000000	958	97.6	955	97	3	0.3	17	1.7	7	0.7	982
	W. bancrofti	LAQH01000000	917	93.4	852	87	65	6.6	38	3.9	27	2.7	982

Table S5. nblast similarity of sequences annotated as belonging to *Wolbachia* from *Dipetalonema gracile* previously sequenced.

gene	accession nb	description	nblast				reference
			% identity with wDcau	with NCBI database			
				description	accession	% identity	
16S	AJ548802	Wolbachia pipientis partial 16S rRNA gene, specific host Dipetalonema gracile	99.71%	achia endosymbiont of Dipetalonema gracile partial 16S rRNA gene (MIB:ZPL:01175)	FR827938	99.78%	Casiraghi et al. 2004
	FR827938	chia endosymbiont of Dipetalonema gracile partial 16S rRNA gene (MIB:ZPL:01175)	99.71%	Wolbachia pipientis partial 16S rRNA gene, specific host Dipetalonema gracile	AJ548802	99.78%	Ferri and al. 2011
	KU255234	Wolbachia endosymbiont of Dipetalonema gracile isolate 63YT MNHN 16S	99.89%	Volbachia endosymbiont of Dipetalonema gracile isolate 215YU ind4 MNHN 16S	KU255233	99.89%	Lefoulon and al. 2016
	KU255233	/olbachia endosymbiont of Dipetalonema gracile isolate 215YU ind4 MNHN 16S	99.18%	achia endosymbiont of Dipetalonema gracile partial 16S rRNA gene (MIB:ZPL:01175)	FR827938	99.08%	Lefoulon and al. 2016
	KU255232	Wolbachia endosymbiont of Dipetalonema gracile isolate 124CV MNHN 16S	99.47%	achia endosymbiont of Dipetalonema gracile partial 16S rRNA gene (MIB:ZPL:01175)	FR827938	99.79%	Lefoulon and al. 2016
gltA	AJ609648	lbachia pipientis (from Dipetalonema gracile) partial gltA gene for citrate synthase	99.06%	Wolbachia endosymbiont of Dipetalonema gracile citrate synthase (gltA) gene	FJ390335	100.00%	Casiraghi et al. 2005
	FJ390335	Wolbachia endosymbiont of Dipetalonema gracile citrate synthase (gltA) gene	98.95%	olbachia pipientis (from Dipetalonema gracile) partial gltA gene for citrate synthase	AJ609648	100.00%	Bordenstein and al. 2009
groEL	AJ609658	Wolbachia pipientis (from Dipetalonema gracile) partial gene for groEL protein	97.81%	Wolbachia pipientis (from Dipetalonema gracile) partial gene for groEL protein	FJ390369	100.00%	Casiraghi et al. 2005
	FJ390369	olbachia endosymbiont of Dipetalonema gracile chaperonin GroEL (groEL) gene	97.81%	Wolbachia pipientis (from Dipetalonema gracile) partial gene for groEL protein	AJ609658	100.00%	Bordenstein and al. 2009
	FR827918	Wolbachia endosymbiont of Dipetalonema gracile partial groEL gene	97.81%	Wolbachia endosymbiont of Dipetalonema gracile chaperonin GroEL gene	FJ390369	100.00%	Ferri and al. 2011
	KU255204	olbachia endosymbiont of Dipetalonema gracile isolate 215YU ind4 MNHN groEL	98.60%	Wolbachia endosymbiont of Dipetalonema gracile chaperonin GroEL gene	FJ390369	99.11%	Lefoulon and al. 2016
	KU255203	Wolbachia endosymbiont of Dipetalonema gracile isolate 124CV MNHN groEL	98.34%	Wolbachia endosymbiont of Dipetalonema gracile chaperonin GroEL gene	FJ390369	99.62%	Lefoulon and al. 2016
ftsZ	FR827924	achia endosymbiont of Dipetalonema gracile partial ftsZ gene for cell division protein	72.37%*	achia endosymbiont of Onchocerca skrjabini partial ftsZ gene for cell division protein	FR827925	96.53%	Ferri and al. 2011
dnaA	FJ390365	Wolbachia endosymbiont of Dipetalonema gracile dnaA gene	83.42%	Wolbachia pipientis strain wMel_ZH26 chromosome, complete genome	CP042445	100.00%	Bordenstein and al. 2009
	KU255295	olbachia endosymbiont of Dipetalonema gracile isolate 124CV MNHN dnaA gene	96.60%	bachia endosymbiont of Dipetalonema gracile isolate 215YU ind4 MNHN dnaA gene	KU255296	98.98%	Lefoulon and al. 2016
	KU255296	achia endosymbiont of Dipetalonema gracile isolate 215YU ind4 MNHN dnaA gene	95.36%	/olbachia endosymbiont of Dipetalonema gracile isolate 124CV MNHN dnaA gene	KU255295	98.98%	Lefoulon and al. 2016
	KU255297	/olbachia endosymbiont of Dipetalonema gracile isolate 63YT MNHN dnaA gene	98.98%	lbachia endosymbiont of Dipetalonema robini isolate 217YU ind7 MNHN dnaA gene	KU255301	97.28%	Lefoulon and al. 2016
coxA	FJ390250	ia endosymbiont of Dipetalonema gracile cytochrome c oxidase subunit I (coxA) gene	98.18%	/olbachia endosymbiont of Dipetalonema gracile isolate 124CV MNHN coxA gene	KU255263	99.22%	Bordenstein and al. 2009
	KU255265	/olbachia endosymbiont of Dipetalonema gracile isolate 63YT MNHN coxA gene	99.98%	bachia endosymbiont of Dipetalonema gracile isolate 215YU ind4 MNHN coxA gene	KU255264	99.47%	Lefoulon and al. 2016
	KU255264	achia endosymbiont of Dipetalonema gracile isolate 215YU ind4 MNHN coxA gene	98.39%	/olbachia endosymbiont of Dipetalonema gracile isolate 124CV MNHN coxA gene	KU255263	99.28%	Lefoulon and al. 2016
	KU255263	olbachia endosymbiont of Dipetalonema gracile isolate 124CV MNHN coxA gene	98.32%	bachia endosymbiont of Dipetalonema gracile isolate 215YU ind4 MNHN coxA gene	KU255264	98.36%	Lefoulon and al. 2016
gatB	KU255375	Volbachia endosymbiont of Dipetalonema gracile isolate 215YU ind4 gatB gene	98.39%	Wolbachia endosymbiont of Dipetalonema gracile isolate 124CV MNHN gatB	KU255374	98.56%	Lefoulon and al. 2016
	KU255374	Wolbachia endosymbiont of Dipetalonema gracile isolate 124CV MNHN gatB	98.02%	Wolbachia endosymbiont of Dipetalonema gracile isolate 215YU ind4 gatB gene	KU255375	98.56%	Lefoulon and al. 2016
fbpA	KU255325	achia endosymbiont of Dipetalonema gracile isolate 215YU ind4 MNHN fbpA gene	96.88%	Volbachia endosymbiont of Dipetalonema gracile isolate 124CV MNHN fbpA gene	KU255324	99.48%	Lefoulon and al. 2016
	KU255324	achia endosymbiont of Dipetalonema gracile isolate 124CV MNHN gatB fbpA gene	97.40%	bachia endosymbiont of Dipetalonema gracile isolate 215YU ind4 MNHN fbpA gene	KU255325	99.48%	Lefoulon and al. 2016
nuoD	FJ390289	Wolbachia endosymbiont of Dipetalonema gracile nuoD gene	97.88%	ia endosymbiont of Onchocerca volvulus str. Cameroon W_O_volvulus_Cameroon_v3	HG810405	88.67	Bordenstein and al. 2009
atpD	FJ390360	hia endosymbiont of Dipetalonema gracile ATP synthase F1 beta subunit (atpD) gene	89.91%	Wolbachia pipientis strain wMel_ZH26 chromosome, complete genome	CP042445	99.83%	Bordenstein and al. 2009
aspC	FJ390330	chia endosymbiont of Dipetalonema gracile aspartate aminotransferase (aspC) gene	81.37%	Wolbachia pipientis strain wAlbB-HN2016 chromosome, complete genome	CP041924	85.92%	Bordenstein and al. 2009
NADH	FJ390209	chia endosymbiont of Dipetalonema gracile NADH dehydrogenase I subunit F gene	98.92%	Wolbachia endosymbiont of Drosophila simulans wHa, complete genome	CP003884	85.18%	Bordenstein and al. 2009
pdhB	FJ390153	Wolbachia endosymbiont of Dipetalonema gracile pdhB gene	85.71%	Volbachia pipientis strain wTei pyruvate dehydrogenase beta subunit (pdhB) gene	DQ235372	99.70%	Bordenstein and al. 2009
hyp	FJ390302	Wolbachia endosymbiont of Dipetalonema gracile hypothetical protein gene	98.28%	Volbachia endosymbiont of Cordylochernes scorpioides hypothetical protein gene	FJ390303	83.88%	Bordenstein and al. 2009
hyp	FJ390237	Wolbachia endosymbiont of Dipetalonema gracile hypothetical protein gene	82.96%	Wolbachia pipientis strain wMel_ZH26 chromosome, complete genome	CP042445	99.81%	Bordenstein and al. 2009
hyp	FJ390176	Wolbachia endosymbiont of Dipetalonema gracile hypothetical protein gene	98.85%	Wolbachia endosymbiont of Onchocerca ochengi complete genome, strain wOo	HE660029	84.78%	Bordenstein and al. 2009
		* 54% coverage only					

Table S6. List of potential insertion sequences element (ISs) identify using ISSAGA.

	IS family	ISL3	IS3			IS4				IS5			IS6	IS200_IS605	IS256	IS481	IS66	IS982	IS630	IS110		ISNCY	total
	sous-group		IS3	IS51	IS150	IS4	IS50	IS231	IS427	IS1031	ISL2	IS903		IS200			ISBst12				IS1111		
wBm	complete	0	0	0	0	0	0	0	0	0	0	0	0	0	0	0	0	0	0	0	0	0	0
	partial	0	0	0	0	0	0	0	0	1	0	0	0	0	0	0	0	0	2	0	0	0	3
	unknown	0	0	0	0	0	0	0	0	0	0	0	0	0	0	0	0	0	0	0	0	0	0
	total	0	0	0	0	0	0	0	0	1	0	0	0	0	0	0	0	0	2	0	0	0	3
wBp	complete	0	0	0	0	0	0	0	0	0	0	0	0	0	0	0	0	0	0	0	0	0	0
	partial	0	0	0	0	0	0	0	0	1	0	0	0	0	0	0	0	0	0	0	0	0	1
	unknown	0	0	0	0	0	0	0	0	0	0	0	0	0	0	0	0	0	0	0	0	0	0
	total	0	0	0	0	0	0	0	0	1	0	0	0	0	0	0	0	0	0	0	0	0	1
wCau	complete	1	2	0	0	0	0	3	0	54	0	0	0	0	0	1	0	0	3	2	2	0	68
	partial	0	0	0	2	6	5	1	0	58	0	0	0	0	0	2	5	1	11	9	1	0	101
	unknown	4	10	0	0	0	0	0	0	0	0	16	0	0	0	0	0	0	1	4	1	0	36
	total	5	12	0	2	6	5	4	0	112	0	16	0	0	0	3	5	1	15	15	4	0	205
wCfeJ	complete	0	0	0	0	0	0	0	0	1	0	0	0	0	0	0	0	2	0	2	0	0	5
	partial	0	0	0	0	1	0	0	1	3	0	0	0	0	0	9	0	1	0	0	0	0	15
	unknown	0	0	0	0	0	0	0	0	0	0	0	0	0	0	0	0	0	0	0	0	0	0
	total	0	0	0	0	1	0	0	1	4	0	0	0	0	0	9	0	3	0	2	0	0	20
wCfeT	complete	0	0	0	0	0	0	0	1	0	0	0	0	0	0	0	104	1	0	0	8	0	114
	partial	0	0	0	0	0	0	0	0	32	0	1	0	0	0	0	87	0	0	0	0	0	120
	unknown	0	0	0	0	0	0	0	0	0	0	55	0	0	0	0	0	0	0	0	0	0	55
	total	0	0	0	0	0	0	0	1	32	0	56	0	0	0	0	191	1	0	0	8	0	289
wCle	complete	0	0	0	0	0	0	0	0	39	0	0	0	0	0	0	0	0	0	0	0	0	39
	partial	2	0	0	0	0	1	0	0	181	0	0	0	0	0	0	0	0	1	4	0	0	189
	unknown	0	0	0	0	1	1	0	0	1	0	0	0	0	0	0	0	0	0	0	0	0	3
	total	2	0	0	0	1	2	0	0	221	0	0	0	0	0	0	0	0	1	4	0	0	231
wCtub	complete	0	0	0	0	0	0	0	0	0	0	0	0	0	0	0	0	0	0	0	0	0	0
	partial	0	0	0	0	0	0	0	0	0	0	0	0	0	0	0	0	0	0	0	0	0	0
	unknown	0	0	0	0	0	0	0	0	0	0	0	0	0	0	0	0	0	0	0	0	0	0
	total	0	0	0	0	0	0	0	0	0	0	0	0	0	0	0	0	0	0	0	0	0	0
wDcau	complete	0	0	0	0	0	0	0	0	0	0	0	0	0	0	0	0	0	0	0	0	0	0
	partial	0	0	0	0	0	0	0	0	0	0	0	0	0	0	0	0	0	0	0	0	0	0
	unknown	0	0	0	0	0	0	0	0	0	0	0	0	0	0	0	0	0	0	0	0	0	0
	total	0	0	0	0	0	0	0	0	0	0	0	0	0	0	0	0	0	0	0	0	0	0

wDimm	complete	0	0	0	0	0	0	0	0	0	0	0	0	0	0	0	0	0	0	0	0	0	0
	partial	0	0	0	0	0	0	0	0	0	0	0	0	0	0	0	0	0	0	0	0	0	0
	unknown	0	0	0	0	0	0	0	0	0	0	0	0	0	0	0	0	0	0	0	0	0	0
	total	0	0	0	0	0	0	0	0	0	0	0	0	0	0	0	0	0	0	0	0	0	0
wFol	complete	0	0	0	0	0	1	0	6	0	0	0	0	0	2	0	0	0	3	15	0	0	27
	partial	0	1	0	0	0	0	0	0	0	0	0	0	0	0	0	0	3	0	12	0	0	16
	unknown	1	0	0	0	16	20	0	0	0	0	13	0	0	0	20	0	0	0	5	0	0	75
	total	1	1	0	0	16	21	0	6	0	0	13	0	0	2	20	0	3	3	32	0	0	118
wLsig	complete	0	0	0	0	0	0	0	0	0	0	0	0	0	0	0	0	0	0	0	0	0	0
	partial	0	0	0	0	0	0	0	0	0	0	0	0	0	0	0	0	0	15	0	0	0	15
	unknown	0	0	0	0	0	0	0	0	0	0	0	0	0	0	0	0	0	2	0	0	0	2
	total	0	0	0	0	0	0	0	0	0	0	0	0	0	0	0	0	0	17	0	0	0	17
wLbra	complete	0	0	0	0	0	0	0	0	0	0	0	0	0	0	0	0	0	1	0	0	0	1
	partial	0	0	0	0	0	0	0	0	0	0	0	0	0	0	0	0	0	32	0	0	0	32
	unknown	0	0	0	0	0	0	0	0	0	0	0	0	0	0	0	0	0	6	0	0	0	6
	total	0	0	0	0	0	0	0	0	0	0	0	0	0	0	0	0	0	39	0	0	0	39
wLug	complete	1	3	0	0	0	0	4	0	4	0	0	3	1	0	0	2	0	1	3	0	0	22
	partial	0	20	0	0	0	2	18	1	14	1	0	0	4	1	8	0	5	7	4	0	0	85
	unknown	1	0	0	0	13	0	0	0	0	0	1	0	0	0	0	0	0	1	0	0	0	16
	total	2	23	0	0	13	2	22	1	18	1	1	3	5	1	8	2	5	9	7	0	0	123
wMel	complete	0	3	0	0	0	0	3	0	13	0	0	0	0	1	1	0	0	3	2	0	0	26
	partial	0	0	1	0	8	5	0	0	16	0	0	0	0	1	0	3	0	2	8	1	0	45
	unknown	1	6	0	0	0	0	0	0	0	0	16	0	0	0	0	0	0	1	3	0	0	27
	total	1	9	1	0	8	5	3	0	29	0	16	0	0	2	1	3	0	6	13	1	0	98
wMhie	complete	1	0	0	0	0	0	0	0	0	0	0	0	0	0	0	0	0	0	0	0	0	1
	partial	0	0	0	0	0	0	0	1	1	0	0	0	0	0	2	1	8	0	0	0	0	13
	unknown	0	0	0	0	0	0	0	1	0	0	0	0	0	0	0	0	0	0	0	0	0	1
	total	1	0	0	0	0	0	0	2	1	0	0	0	0	0	2	1	8	0	0	0	0	15
wNfla	complete	0	2	0	0	0	0	0	0	0	2	0	0	0	0	1	0	0	1	2	0	0	8
	partial	0	0	1	0	4	4	3	0	0	23	0	0	0	3	2	3	2	5	6	1	0	57
	unknown	1	1	0	0	0	0	0	0	0	0	3	0	0	0	0	0	0	0	2	1	0	8
	total	1	3	1	0	4	4	3	0	0	25	3	0	0	3	3	3	2	6	10	2	0	73
wOo	complete	0	0	0	0	0	0	0	0	0	0	0	0	0	0	0	0	0	0	0	0	0	0
	partial	0	0	0	0	0	0	1	0	0	0	0	0	0	0	0	0	0	0	0	0	0	1
	unknown	0	0	0	0	0	0	0	0	0	0	0	0	0	0	0	0	0	0	0	0	0	0
	total	0	0	0	0	0	0	1	0	0	0	0	0	0	0	0	0	0	0	0	0	0	1
wOv	complete	0	0	0	0	0	0	0	0	0	0	0	0	0	0	0	0	0	0	0	0	0	0

	partial	0	0	0	0	0	0	0	0	0	0	0	0	0	0	0	0	0	0	0	0	0	0
	unknown	0	0	0	0	0	0	0	0	0	0	0	0	0	0	0	0	0	0	0	0	0	0
	total	0	0	0	0	0	0	0	0	0	0	0	0	0	0	0	0	0	0	0	0	0	0
wPip	complete	0	0	0	0	0	0	0	0	1	0	0	0	0	4	0	1	44	10	3	0	0	63
	partial	1	0	0	0	0	0	0	0	3	0	1	9	3	36	0	0	8	4	0	0	0	65
	unknown	2	0	0	0	0	0	0	0	0	0	26	0	0	0	0	0	0	0	0	0	1	29
	total	3	0	0	0	0	1	0	0	4	0	27	9	3	40	0	0	52	14	3	0	1	157
wPpe	complete	0	0	0	0	0	0	0	0	0	0	0	0	0	0	0	0	0	0	0	0	0	0
	partial	0	0	0	0	0	0	0	0	0	6	0	0	0	0	0	0	0	0	0	0	0	6
	unknown	0	0	0	0	0	0	0	0	0	1	0	0	0	0	0	0	0	0	0	0	0	1
	total	0	0	0	0	0	0	0	0	0	7	0	0	0	0	0	0	0	0	0	0	0	7
wstri	complete	2	21	0	0	0	0	0	0	5	0	0	1	11	0	0	0	6	3	14	0	0	63
	partial	0	0	0	0	0	0	1	0	4	1	0	0	1	3	0	0	0	1	6	0	0	17
	unknown	1	0	0	0	0	0	0	0	0	0	2	0	0	0	0	0	0	0	0	0	0	3
	total	3	21	0	0	0	0	1	0	9	1	2	1	12	3	0	0	6	4	20	0	0	83
wTpre	complete	0	0	0	0	0	0	0	0	0	0	0	0	0	0	0	0	0	0	0	0	0	0
	partial	0	0	0	0	0	0	19	0	2	1	0	0	0	0	2	0	0	1	1	0	0	26
	unknown	0	0	0	0	0	0	0	0	0	0	0	0	0	0	0	0	0	0	0	0	0	0
	total	0	0	0	0	0	0	19	0	2	1	0	0	0	0	2	0	0	1	1	0	0	26
wVul C	complete	0	0	0	17	9	0	0	0	0	0	0	0	0	0	0	2	0	5	9	0	0	42
	partial	2	0	0	8	6	29	3	2	2	7	0	0	0	31	11	4	0	10	46	6	0	167
	unknown	1	3	0	0	1	0	0	0	0	0	5	0	0	0	0	0	0	0	0	0	0	10
	total	3	3	0	25	16	29	3	2	2	7	5	0	0	31	11	6	0	15	55	6	0	219
wWb	complete	0	0	0	0	0	0	0	0	0	0	0	0	0	0	0	0	0	0	0	0	0	0
	partial	1	0	0	0	0	0	0	0	1	0	0	0	0	2	0	0	0	0	0	0	0	4
	unknown	0	0	0	0	0	0	0	0	0	0	0	0	0	0	0	0	0	0	0	0	0	0
	total	1	0	0	0	0	0	0	0	1	0	0	0	0	2	0	0	0	0	0	0	0	4

Table S7. List of Group II intron-associated genes detected by RAST pipeline.

genome	Feature ID	Type	Contig	Start	Stop	Strand	Length (bp)	Function	Subsystem
wBm	-	-	-	-	-	-	-	-	-
wBp	-	-	-	-	-	-	-	-	-
wCauA	fig|2591635.5.peg.438	CDS	CP041215.1	409378	409653	+	276	Retron-type RNA-directed DNA polymerase (EC 2.7.7.49)	Group II intron-associated genes
	fig|2591635.5.peg.1150	CDS	CP041215.1	1085440	1086753	+	1314	Retron-type RNA-directed DNA polymerase (EC 2.7.7.49)	Group II intron-associated genes
wCtub	-	-	-	-	-	-	-	-	-
wCfeJ	fig|212123.3.peg.24	CDS	CP051157.1	20515	19613	-	903	Retron-type RNA-directed DNA polymerase (EC 2.7.7.49)	Group II intron-associated genes
	fig|212123.3.peg.25	CDS	CP051157.1	21483	20473	-	1011	Retron-type RNA-directed DNA polymerase (EC 2.7.7.49)	Group II intron-associated genes
	fig|212123.3.peg.915	CDS	CP051157.1	943435	945081	+	1647	Retron-type RNA-directed DNA polymerase (EC 2.7.7.49)	Group II intron-associated genes
wCfeT	fig|212123.4.peg.109	CDS	CP051156.1	104955	106331	+	1377	Retron-type RNA-directed DNA polymerase (EC 2.7.7.49)	Group II intron-associated genes
	fig|212123.4.peg.120	CDS	CP051156.1	112631	114007	+	1377	Retron-type RNA-directed DNA polymerase (EC 2.7.7.49)	Group II intron-associated genes
	fig|212123.4.peg.131	CDS	CP051156.1	120307	121683	+	1377	Retron-type RNA-directed DNA polymerase (EC 2.7.7.49)	Group II intron-associated genes
	fig|212123.4.peg.904	CDS	CP051156.1	913030	911393	-	1638	Retron-type RNA-directed DNA polymerase (EC 2.7.7.49)	Group II intron-associated genes
	fig|212123.4.peg.1096	CDS	CP051156.1	1111462	1112943	+	1482	Retron-type RNA-directed DNA polymerase (EC 2.7.7.49)	Group II intron-associated genes
	fig|212123.4.peg.1236	CDS	CP051156.1	1238861	1236990	-	1872	Retron-type RNA-directed DNA polymerase (EC 2.7.7.49)	Group II intron-associated genes
	fig|212123.4.peg.1413	CDS	CP051156.1	1396973	1395642	-	1332	Retron-type RNA-directed DNA polymerase (EC 2.7.7.49)	Group II intron-associated genes
	fig|212123.4.peg.1457	CDS	CP051156.1	1436914	1438551	+	1638	Retron-type RNA-directed DNA polymerase (EC 2.7.7.49)	Group II intron-associated genes
wCle	-	-	-	-	-	-	-	-	-
wDimm	-	-	-	-	-	-	-	-	-
wDcau	-	-	-	-	-	-	-	-	-
wFol	fig|169402.10.peg.411	CDS	NZ_CP015510.2	425791	426789	+	999	Retron-type RNA-directed DNA polymerase (EC 2.7.7.49)	Group II intron-associated genes
	fig|169402.10.peg.1022	CDS	NZ_CP015510.2	1083651	1082887	-	765	Retron-type RNA-directed DNA polymerase (EC 2.7.7.49)	Group II intron-associated genes
	fig|169402.10.peg.1146	CDS	NZ_CP015510.2	1223669	1223989	+	321	Retron-type RNA-directed DNA polymerase (EC 2.7.7.49)	Group II intron-associated genes
	fig|169402.10.peg.1149	CDS	NZ_CP015510.2	1226526	1227566	+	1041	Retron-type RNA-directed DNA polymerase (EC 2.7.7.49)	Group II intron-associated genes
	fig|169402.10.peg.1169	CDS	NZ_CP015510.2	1246407	1246850	+	444	Retron-type RNA-directed DNA polymerase (EC 2.7.7.49)	Group II intron-associated genes
wMel	fig|163164.1.peg.621	CDS	NC_002978	669520	671067	+	1548	Retron-type RNA-directed DNA polymerase (EC 2.7.7.49)	Group II intron-associated genes
	fig|163164.1.peg.887	CDS	NC_002978	951605	953152	+	1548	Retron-type RNA-directed DNA polymerase (EC 2.7.7.49)	Group II intron-associated genes
	fig|163164.1.peg.1020	CDS	NC_002978	1090833	1089286	-	1548	Retron-type RNA-directed DNA polymerase (EC 2.7.7.49)	Group II intron-associated genes
	fig|163164.1.peg.1198	CDS	NC_002978	48840	48064	-	777	Retron-type RNA-directed DNA polymerase (EC 2.7.7.49)	Group II intron-associated genes
	fig|163164.1.peg.1218	CDS	NC_002978	245008	243932	-	1077	Retron-type RNA-directed DNA polymerase (EC 2.7.7.49)	Group II intron-associated genes
	fig|163164.1.peg.1241	CDS	NC_002978	378869	377574	-	1296	Retron-type RNA-directed DNA polymerase (EC 2.7.7.49)	Group II intron-associated genes
	fig|163164.1.peg.1246	CDS	NC_002978	487718	486642	-	1077	Retron-type RNA-directed DNA polymerase (EC 2.7.7.49)	Group II intron-associated genes
	fig|163164.1.peg.1248	CDS	NC_002978	509575	508823	-	753	Retron-type RNA-directed DNA polymerase (EC 2.7.7.49)	Group II intron-associated genes
	fig|163164.1.peg.1251	CDS	NC_002978	523124	524419	+	1296	Retron-type RNA-directed DNA polymerase (EC 2.7.7.49)	Group II intron-associated genes
	fig|163164.1.peg.1260	CDS	NC_002978	583296	584372	+	1077	Retron-type RNA-directed DNA polymerase (EC 2.7.7.49)	Group II intron-associated genes
wMhie	-	-	-	-	-	-	-	-	-
wLbra	-	-	-	-	-	-	-	-	-
wLsig	-	-	-	-	-	-	-	-	-

wLug	fig|1335053.6.peg.47	CDS	MUIY01000001.1	45754	47004	+		1251	Retron-type RNA-directed DNA polymerase (EC 2.7.7.49)	Group II intron-associated genes
	fig|1335053.6.peg.55	CDS	MUIY01000001.1	52785	54035	+		1251	Retron-type RNA-directed DNA polymerase (EC 2.7.7.49)	Group II intron-associated genes
	fig|1335053.6.peg.94	CDS	MUIY01000001.1	85363	84092	-		1272	Retron-type RNA-directed DNA polymerase (EC 2.7.7.49)	Group II intron-associated genes
	fig|1335053.6.peg.598	CDS	MUIY01000001.1	559490	560740	+		1251	Retron-type RNA-directed DNA polymerase (EC 2.7.7.49)	Group II intron-associated genes
	fig|1335053.6.peg.651	CDS	MUIY01000001.1	604803	606050	+		1248	Retron-type RNA-directed DNA polymerase (EC 2.7.7.49)	Group II intron-associated genes
	fig|1335053.6.peg.909	CDS	MUIY01000001.1	842776	843441	+		666	Retron-type RNA-directed DNA polymerase (EC 2.7.7.49)	Group II intron-associated genes
	fig|1335053.6.peg.914	CDS	MUIY01000001.1	849817	848855	-		963	Retron-type RNA-directed DNA polymerase (EC 2.7.7.49)	Group II intron-associated genes
	fig|1335053.6.peg.994	CDS	MUIY01000001.1	932421	931174	-		1248	Retron-type RNA-directed DNA polymerase (EC 2.7.7.49)	Group II intron-associated genes
	fig|1335053.6.peg.1161	CDS	MUIY01000001.1	1091876	1093126	+		1251	Retron-type RNA-directed DNA polymerase (EC 2.7.7.49)	Group II intron-associated genes
	fig|1335053.6.peg.1219	CDS	MUIY01000001.1	1144340	1143090	-		1251	Retron-type RNA-directed DNA polymerase (EC 2.7.7.49)	Group II intron-associated genes
	fig|1335053.6.peg.1258	CDS	MUIY01000002.1	18649	17402	-		1248	Retron-type RNA-directed DNA polymerase (EC 2.7.7.49)	Group II intron-associated genes
	fig|1335053.6.peg.1442	CDS	MUIY01000002.1	214193	215443	+		1251	Retron-type RNA-directed DNA polymerase (EC 2.7.7.49)	Group II intron-associated genes
	fig|1335053.6.peg.1451	CDS	MUIY01000002.1	224755	223667	-		1089	Retron-type RNA-directed DNA polymerase (EC 2.7.7.49)	Group II intron-associated genes
	fig|1335053.6.peg.1532	CDS	MUIY01000002.1	306864	305617	-		1248	Retron-type RNA-directed DNA polymerase (EC 2.7.7.49)	Group II intron-associated genes
wNfla	fig|1854759.6.peg.501	CDS	NZ_LYUW01000015.1	14040	13885	-		156	Retron-type RNA-directed DNA polymerase (EC 2.7.7.49)	Group II intron-associated genes
	fig|1854759.6.peg.1363	CDS	NZ_LYUW01000091.1	327	37	-		291	Retron-type RNA-directed DNA polymerase (EC 2.7.7.49)	Group II intron-associated genes
	fig|1854759.6.peg.1364	CDS	NZ_LYUW01000091.1	1204	308	-		897	Retron-type RNA-directed DNA polymerase (EC 2.7.7.49)	Group II intron-associated genes
	fig|1854759.6.peg.1441	CDS	NZ_LYUW01000110.1	535	1785	+		1251	Retron-type RNA-directed DNA polymerase (EC 2.7.7.49)	Group II intron-associated genes
	fig|1854759.6.peg.1507	CDS	NZ_LYUW01000147.1	243	1	-		243	Retron-type RNA-directed DNA polymerase (EC 2.7.7.49)	Group II intron-associated genes
	fig|1854759.6.peg.1509	CDS	NZ_LYUW01000151.1	369	1	-		369	Retron-type RNA-directed DNA polymerase (EC 2.7.7.49)	Group II intron-associated genes
wOv	-	-	-	-	-	-	-		-	-
wOo	-	-	-	-	-	-	-		-	-
wPip	fig|955.1.peg.354	CDS	culex88a09.p1k	1374	2717	+		1344	Retron-type RNA-directed DNA polymerase (EC 2.7.7.49)	Group II intron-associated genes
	fig|955.1.peg.1056	CDS	culex35f07.p1k	1768	1013	-		756	Retron-type RNA-directed DNA polymerase (EC 2.7.7.49)	Group II intron-associated genes
	fig|955.1.peg.1057	CDS	culex35f07.p1k	3045	1801	-		1245	Retron-type RNA-directed DNA polymerase (EC 2.7.7.49)	Group II intron-associated genes
	fig|955.1.peg.1059	CDS	culex35f07.p1k	4408	3557	-		852	Retron-type RNA-directed DNA polymerase (EC 2.7.7.49)	Group II intron-associated genes
	fig|955.1.peg.1062	CDS	culex35f07.p1k	7184	5841	-		1344	Retron-type RNA-directed DNA polymerase (EC 2.7.7.49)	Group II intron-associated genes
	fig|955.1.peg.1375	CDS	culex143c03.p1k	18716	17415	-		1302	Retron-type RNA-directed DNA polymerase (EC 2.7.7.49)	Group II intron-associated genes
wPpe	-	-	-	-	-	-	-		-	-
wstri	fig|368602.8.peg.17	CDS	MUIX01000001.1	14980	13754	-		1227	Retron-type RNA-directed DNA polymerase (EC 2.7.7.49)	Group II intron-associated genes
	fig|368602.8.peg.28	CDS	MUIX01000001.1	22475	23326	+		852	Retron-type RNA-directed DNA polymerase (EC 2.7.7.49)	Group II intron-associated genes
	fig|368602.8.peg.30	CDS	MUIX01000001.1	23840	25084	+		1245	Retron-type RNA-directed DNA polymerase (EC 2.7.7.49)	Group II intron-associated genes
	fig|368602.8.peg.31	CDS	MUIX01000001.1	25138	25872	+		735	Retron-type RNA-directed DNA polymerase (EC 2.7.7.49)	Group II intron-associated genes
	fig|368602.8.peg.37	CDS	MUIX01000001.1	28602	29453	+		852	Retron-type RNA-directed DNA polymerase (EC 2.7.7.49)	Group II intron-associated genes
	fig|368602.8.peg.39	CDS	MUIX01000001.1	29967	31211	+		1245	Retron-type RNA-directed DNA polymerase (EC 2.7.7.49)	Group II intron-associated genes
	fig|368602.8.peg.40	CDS	MUIX01000001.1	31265	32230	+		966	Retron-type RNA-directed DNA polymerase (EC 2.7.7.49)	Group II intron-associated genes
	fig|368602.8.peg.43	CDS	MUIX01000001.1	38813	40057	+		1245	Retron-type RNA-directed DNA polymerase (EC 2.7.7.49)	Group II intron-associated genes
	fig|368602.8.peg.44	CDS	MUIX01000001.1	40111	40845	+		735	Retron-type RNA-directed DNA polymerase (EC 2.7.7.49)	Group II intron-associated genes
	fig|368602.8.peg.81	CDS	MUIX01000001.1	75109	73883	-		1227	Retron-type RNA-directed DNA polymerase (EC 2.7.7.49)	Group II intron-associated genes
	fig|368602.8.peg.108	CDS	MUIX01000001.1	98449	97136	-		1314	Retron-type RNA-directed DNA polymerase (EC 2.7.7.49)	Group II intron-associated genes
	fig|368602.8.peg.132	CDS	MUIX01000001.1	118288	119757	+		1470	Retron-type RNA-directed DNA polymerase (EC 2.7.7.49)	Group II intron-associated genes

fig|368602.8.peg.190	CDS	MUIX01000001.1	177581	176355	-	1227	Retron-type RNA-directed DNA polymerase (EC 2.7.7.49)	Group II intron-associated genes
fig|368602.8.peg.208	CDS	MUIX01000001.1	190567	191880	+	1314	Retron-type RNA-directed DNA polymerase (EC 2.7.7.49)	Group II intron-associated genes
fig|368602.8.peg.219	CDS	MUIX01000001.1	210525	210046	-	480	Retron-type RNA-directed DNA polymerase (EC 2.7.7.49)	Group II intron-associated genes
fig|368602.8.peg.279	CDS	MUIX01000001.1	258901	257630	-	1272	Retron-type RNA-directed DNA polymerase (EC 2.7.7.49)	Group II intron-associated genes
fig|368602.8.peg.342	CDS	MUIX01000001.1	318580	317309	-	1272	Retron-type RNA-directed DNA polymerase (EC 2.7.7.49)	Group II intron-associated genes
fig|368602.8.peg.353	CDS	MUIX01000001.1	329350	330594	+	1245	Retron-type RNA-directed DNA polymerase (EC 2.7.7.49)	Group II intron-associated genes
fig|368602.8.peg.354	CDS	MUIX01000001.1	330648	331382	+	735	Retron-type RNA-directed DNA polymerase (EC 2.7.7.49)	Group II intron-associated genes
fig|368602.8.peg.394	CDS	MUIX01000001.1	373387	372161	-	1227	Retron-type RNA-directed DNA polymerase (EC 2.7.7.49)	Group II intron-associated genes
fig|368602.8.peg.644	CDS	MUIX01000001.1	642493	643974	+	1482	Retron-type RNA-directed DNA polymerase (EC 2.7.7.49)	Group II intron-associated genes
fig|368602.8.peg.656	CDS	MUIX01000001.1	657736	656510	-	1227	Retron-type RNA-directed DNA polymerase (EC 2.7.7.49)	Group II intron-associated genes
fig|368602.8.peg.660	CDS	MUIX01000001.1	662316	661003	-	1314	Retron-type RNA-directed DNA polymerase (EC 2.7.7.49)	Group II intron-associated genes
fig|368602.8.peg.701	CDS	MUIX01000001.1	696321	695050	-	1272	Retron-type RNA-directed DNA polymerase (EC 2.7.7.49)	Group II intron-associated genes
fig|368602.8.peg.767	CDS	MUIX01000001.1	781667	780198	-	1470	Retron-type RNA-directed DNA polymerase (EC 2.7.7.49)	Group II intron-associated genes
fig|368602.8.peg.977	CDS	MUIX01000001.1	970601	969375	-	1227	Retron-type RNA-directed DNA polymerase (EC 2.7.7.49)	Group II intron-associated genes
fig|368602.8.peg.1080	CDS	MUIX01000001.1	1060427	1059156	-	1272	Retron-type RNA-directed DNA polymerase (EC 2.7.7.49)	Group II intron-associated genes
fig|368602.8.peg.1261	CDS	MUIX01000001.1	1239295	1238561	-	735	Retron-type RNA-directed DNA polymerase (EC 2.7.7.49)	Group II intron-associated genes
fig|368602.8.peg.1262	CDS	MUIX01000001.1	1240593	1239349	-	1245	Retron-type RNA-directed DNA polymerase (EC 2.7.7.49)	Group II intron-associated genes
fig|368602.8.peg.1264	CDS	MUIX01000001.1	1241958	1241107	-	852	Retron-type RNA-directed DNA polymerase (EC 2.7.7.49)	Group II intron-associated genes
fig|368602.8.peg.1312	CDS	MUIX01000001.1	1287747	1288988	+	1242	Retron-type RNA-directed DNA polymerase (EC 2.7.7.49)	Group II intron-associated genes
fig|368602.8.peg.1343	CDS	MUIX01000001.1	1314385	1315854	+	1470	Retron-type RNA-directed DNA polymerase (EC 2.7.7.49)	Group II intron-associated genes
fig|368602.8.peg.1376	CDS	MUIX01000001.1	1340599	1342146	+	1548	Retron-type RNA-directed DNA polymerase (EC 2.7.7.49)	Group II intron-associated genes
fig|368602.8.peg.1443	CDS	MUIX01000001.1	1413840	1415321	+	1482	Retron-type RNA-directed DNA polymerase (EC 2.7.7.49)	Group II intron-associated genes
fig|368602.8.peg.1506	CDS	MUIX01000001.1	1474450	1473209	-	1242	Retron-type RNA-directed DNA polymerase (EC 2.7.7.49)	Group II intron-associated genes
fig|368602.8.peg.1514	CDS	MUIX01000001.1	1481600	1480119	-	1482	Retron-type RNA-directed DNA polymerase (EC 2.7.7.49)	Group II intron-associated genes
fig|368602.8.peg.1524	CDS	MUIX01000001.1	1488474	1489700	+	1227	Retron-type RNA-directed DNA polymerase (EC 2.7.7.49)	Group II intron-associated genes
fig|368602.8.peg.1580	CDS	MUIX01000001.1	1549498	1550769	+	1272	Retron-type RNA-directed DNA polymerase (EC 2.7.7.49)	Group II intron-associated genes
fig|368602.8.peg.1587	CDS	MUIX01000001.1	1561281	1562594	+	1314	Retron-type RNA-directed DNA polymerase (EC 2.7.7.49)	Group II intron-associated genes
fig|368602.8.peg.1591	CDS	MUIX01000001.1	1568851	1567370	-	1482	Retron-type RNA-directed DNA polymerase (EC 2.7.7.49)	Group II intron-associated genes
fig|368602.8.peg.1599	CDS	MUIX01000001.1	1576075	1577346	+	1272	Retron-type RNA-directed DNA polymerase (EC 2.7.7.49)	Group II intron-associated genes
fig|368602.8.peg.1604	CDS	MUIX01000001.1	1582692	1581451	-	1242	Retron-type RNA-directed DNA polymerase (EC 2.7.7.49)	Group II intron-associated genes
fig|368602.8.peg.1610	CDS	MUIX01000001.1	1586192	1584879	-	1314	Retron-type RNA-directed DNA polymerase (EC 2.7.7.49)	Group II intron-associated genes
fig|368602.8.peg.1618	CDS	MUIX01000001.1	1592503	1591262	-	1242	Retron-type RNA-directed DNA polymerase (EC 2.7.7.49)	Group II intron-associated genes
fig|368602.8.peg.1624	CDS	MUIX01000001.1	1596003	1594690	-	1314	Retron-type RNA-directed DNA polymerase (EC 2.7.7.49)	Group II intron-associated genes
fig|368602.8.peg.1654	CDS	MUIX01000002.1	16889	15576	-	1314	Retron-type RNA-directed DNA polymerase (EC 2.7.7.49)	Group II intron-associated genes
fig|368602.8.peg.1657	CDS	MUIX01000002.1	19278	20759	+	1482	Retron-type RNA-directed DNA polymerase (EC 2.7.7.49)	Group II intron-associated genes
fig|368602.8.peg.1659	CDS	MUIX01000002.1	21319	21798	+	480	Retron-type RNA-directed DNA polymerase (EC 2.7.7.49)	Group II intron-associated genes
fig|368602.8.peg.1708	CDS	MUIX01000002.1	63777	62464	-	1314	Retron-type RNA-directed DNA polymerase (EC 2.7.7.49)	Group II intron-associated genes
fig|368602.8.peg.1715	CDS	MUIX01000002.1	70074	71345	+	1272	Retron-type RNA-directed DNA polymerase (EC 2.7.7.49)	Group II intron-associated genes
fig|368602.8.peg.1762	CDS	MUIX01000002.1	109901	108630	-	1272	Retron-type RNA-directed DNA polymerase (EC 2.7.7.49)	Group II intron-associated genes
fig|368602.8.peg.1798	CDS	MUIX01000002.1	152978	154162	+	1185	Retron-type RNA-directed DNA polymerase (EC 2.7.7.49)	Group II intron-associated genes
fig|368602.8.peg.1799	CDS	MUIX01000002.1	154216	154950	+	735	Retron-type RNA-directed DNA polymerase (EC 2.7.7.49)	Group II intron-associated genes

	fig|368602.8.peg.1805	CDS	MUIX01000002.1	160079	161305	+		1227	Retron-type RNA-directed DNA polymerase (EC 2.7.7.49)	Group II intron-associated genes
wTpre	fig|125593.4.peg.526	CDS	NZ_CM003641.1	421271	420969	-		303	Retron-type RNA-directed DNA polymerase (EC 2.7.7.49)	Group II intron-associated genes
	fig|125593.4.peg.527	CDS	NZ_CM003641.1	421627	421268	-		360	Retron-type RNA-directed DNA polymerase (EC 2.7.7.49)	Group II intron-associated genes
	fig|125593.4.peg.528	CDS	NZ_CM003641.1	421909	421640	-		270	Retron-type RNA-directed DNA polymerase (EC 2.7.7.49)	Group II intron-associated genes
wVulC	fig|1220511.6.peg.141	CDS	ALWU01000001.1	131094	130579	-		516	Retron-type RNA-directed DNA polymerase (EC 2.7.7.49)	Group II intron-associated genes
	fig|1220511.6.peg.214	CDS	ALWU01000001.1	194427	193747	-		681	Retron-type RNA-directed DNA polymerase (EC 2.7.7.49)	Group II intron-associated genes
	fig|1220511.6.peg.216	CDS	ALWU01000001.1	194982	195479	+		498	Retron-type RNA-directed DNA polymerase (EC 2.7.7.49)	Group II intron-associated genes
	fig|1220511.6.peg.344	CDS	ALWU01000001.1	300284	299736	-		549	Retron-type RNA-directed DNA polymerase (EC 2.7.7.49)	Group II intron-associated genes
	fig|1220511.6.peg.345	CDS	ALWU01000001.1	301515	300343	-		1173	Retron-type RNA-directed DNA polymerase (EC 2.7.7.49)	Group II intron-associated genes
	fig|1220511.6.peg.347	CDS	ALWU01000001.1	302964	302620	-		345	Retron-type RNA-directed DNA polymerase (EC 2.7.7.49)	Group II intron-associated genes
	fig|1220511.6.peg.352	CDS	ALWU01000001.1	304451	304873	+		423	Retron-type RNA-directed DNA polymerase (EC 2.7.7.49)	Group II intron-associated genes
	fig|1220511.6.peg.363	CDS	ALWU01000001.1	311311	311826	+		516	Retron-type RNA-directed DNA polymerase (EC 2.7.7.49)	Group II intron-associated genes
	fig|1220511.6.peg.379	CDS	ALWU01000001.1	323123	322341	-		783	Retron-type RNA-directed DNA polymerase (EC 2.7.7.49)	Group II intron-associated genes
	fig|1220511.6.peg.699	CDS	ALWU01000002.1	14691	13576	-		1116	Retron-type RNA-directed DNA polymerase (EC 2.7.7.49)	Group II intron-associated genes
	fig|1220511.6.peg.700	CDS	ALWU01000002.1	15415	14744	-		672	Retron-type RNA-directed DNA polymerase (EC 2.7.7.49)	Group II intron-associated genes
	fig|1220511.6.peg.847	CDS	ALWU01000005.1	30121	30366	+		246	Retron-type RNA-directed DNA polymerase (EC 2.7.7.49)	Group II intron-associated genes
	fig|1220511.6.peg.857	CDS	ALWU01000005.1	36848	37093	+		246	Retron-type RNA-directed DNA polymerase (EC 2.7.7.49)	Group II intron-associated genes
	fig|1220511.6.peg.946	CDS	ALWU01000005.1	113463	112963	-		501	Retron-type RNA-directed DNA polymerase (EC 2.7.7.49)	Group II intron-associated genes
	fig|1220511.6.peg.1040	CDS	ALWU01000005.1	190520	192301	+		1782	Retron-type RNA-directed DNA polymerase (EC 2.7.7.49)	Group II intron-associated genes
	fig|1220511.6.peg.1079	CDS	ALWU01000006.1	3901	5682	+		1782	Retron-type RNA-directed DNA polymerase (EC 2.7.7.49)	Group II intron-associated genes
	fig|1220511.6.peg.1108	CDS	ALWU01000006.1	28951	29604	+		654	Retron-type RNA-directed DNA polymerase (EC 2.7.7.49)	Group II intron-associated genes
	fig|1220511.6.peg.1113	CDS	ALWU01000006.1	32799	33422	+		624	Retron-type RNA-directed DNA polymerase (EC 2.7.7.49)	Group II intron-associated genes
	fig|1220511.6.peg.1327	CDS	ALWU01000008.1	175500	174985	-		516	Retron-type RNA-directed DNA polymerase (EC 2.7.7.49)	Group II intron-associated genes
	fig|1220511.6.peg.1530	CDS	ALWU01000010.1	148506	148321	-		186	Retron-type RNA-directed DNA polymerase (EC 2.7.7.49)	Group II intron-associated genes
	fig|1220511.6.peg.1602	CDS	ALWU01000010.1	208558	208139	-		420	Retron-type RNA-directed DNA polymerase (EC 2.7.7.49)	Group II intron-associated genes
	fig|1220511.6.peg.1603	CDS	ALWU01000010.1	208853	208548	-		306	Retron-type RNA-directed DNA polymerase (EC 2.7.7.49)	Group II intron-associated genes
	fig|1220511.6.peg.1610	CDS	ALWU01000010.1	212470	212775	+		306	Retron-type RNA-directed DNA polymerase (EC 2.7.7.49)	Group II intron-associated genes
	fig|1220511.6.peg.1611	CDS	ALWU01000010.1	212765	213184	+		420	Retron-type RNA-directed DNA polymerase (EC 2.7.7.49)	Group II intron-associated genes
	fig|1220511.6.peg.1690	CDS	ALWU01000010.1	276574	277398	+		825	Retron-type RNA-directed DNA polymerase (EC 2.7.7.49)	Group II intron-associated genes
	fig|1220511.6.peg.1691	CDS	ALWU01000010.1	277373	277630	+		258	Retron-type RNA-directed DNA polymerase (EC 2.7.7.49)	Group II intron-associated genes
	fig|1220511.6.peg.1692	CDS	ALWU01000010.1	277620	278039	+		420	Retron-type RNA-directed DNA polymerase (EC 2.7.7.49)	Group II intron-associated genes
	fig|1220511.6.peg.1799	CDS	ALWU01000010.1	377187	376435	-		753	Retron-type RNA-directed DNA polymerase (EC 2.7.7.49)	Group II intron-associated genes
	fig|1220511.6.peg.1801	CDS	ALWU01000010.1	377742	378239	+		498	Retron-type RNA-directed DNA polymerase (EC 2.7.7.49)	Group II intron-associated genes
	fig|1220511.6.peg.1941	CDS	ALWU01000010.1	507287	507802	+		516	Retron-type RNA-directed DNA polymerase (EC 2.7.7.49)	Group II intron-associated genes
wWb	-	-	-	-	-	-	-		-	-

Table S8. List of mobile element genes detected using RAST pipeline.

genome	Feature ID	Type	Contig	Start	Stop	Strand	Length (bp)	Function	Subsystem
wBm	fig|292805.13.peg.74	CDS	wBm_reseq_reversed	45903	45631	-	273	Mobile element protein	- none -
	fig|292805.13.peg.515	CDS	wBm_reseq_reversed	356167	355979	-	189	Mobile element protein	- none -
	fig|292805.13.peg.760	CDS	wBm_reseq_reversed	522826	522984	+	159	Mobile element protein	- none -
wBp	fig|96495.5.peg.194	CDS	NZ_CP050521.1	152947	153219	+	273	Mobile element protein	- none -
	fig|96495.5.peg.254	CDS	NZ_CP050521.1	200393	200121	-	273	Mobile element protein	- none -
	fig|96495.5.peg.1182	CDS	NZ_CP050521.1	969737	969579	-	159	Mobile element protein	- none -
	fig|96495.5.peg.1215	CDS	NZ_CP050521.1	991912	992184	+	273	Mobile element protein	- none -
	fig|96495.5.peg.1244	CDS	NZ_CP050521.1	1028753	1028592	-	162	Mobile element protein	- none -
	fig|96495.5.peg.1285	CDS	NZ_CP050521.1	1054315	1054587	+	273	Mobile element protein	- none -
	fig|96495.5.peg.1302	CDS	NZ_CP050521.1	1068612	1068884	+	273	Mobile element protein	- none -
wCtub	-	-	-	-	-	-	-	-	-
wCauA	fig|2591635.5.peg.23	CDS	CP041215.1	24095	24379	+	285	Mobile element protein	- none -
	fig|2591635.5.peg.25	CDS	CP041215.1	25017	25145	+	129	Mobile element protein	- none -
	fig|2591635.5.peg.34	CDS	CP041215.1	35460	35825	+	366	Mobile element protein	- none -
	fig|2591635.5.peg.53	CDS	CP041215.1	51796	52161	+	366	Mobile element protein	- none -
	fig|2591635.5.peg.58	CDS	CP041215.1	54789	55154	+	366	Mobile element protein	- none -
	fig|2591635.5.peg.70	CDS	CP041215.1	65690	65502	-	189	Mobile element protein	- none -
	fig|2591635.5.peg.74	CDS	CP041215.1	66495	66292	-	204	Mobile element protein	- none -
	fig|2591635.5.peg.76	CDS	CP041215.1	67349	66984	-	366	Mobile element protein	- none -
	fig|2591635.5.peg.139	CDS	CP041215.1	122153	122482	+	330	Mobile element protein	- none -
	fig|2591635.5.peg.140	CDS	CP041215.1	122466	123101	+	636	Mobile element protein	- none -

fig|2591635.5.peg.150	CDS	CP041215.1	131020	131289	+	270	Mobile element protein	- none -
fig|2591635.5.peg.156	CDS	CP041215.1	136492	136857	+	366	Mobile element protein	- none -
fig|2591635.5.peg.206	CDS	CP041215.1	190832	190197	-	636	Mobile element protein	- none -
fig|2591635.5.peg.207	CDS	CP041215.1	191145	190816	-	330	Mobile element protein	- none -
fig|2591635.5.peg.218	CDS	CP041215.1	201739	201374	-	366	Mobile element protein	- none -
fig|2591635.5.peg.232	CDS	CP041215.1	216153	215788	-	366	Mobile element protein	- none -
fig|2591635.5.peg.259	CDS	CP041215.1	241662	241297	-	366	Mobile element protein	- none -
fig|2591635.5.peg.262	CDS	CP041215.1	243248	242916	-	333	Mobile element protein	- none -
fig|2591635.5.peg.276	CDS	CP041215.1	254122	253757	-	366	Mobile element protein	- none -
fig|2591635.5.peg.299	CDS	CP041215.1	270346	270651	+	306	Mobile element protein	- none -
fig|2591635.5.peg.311	CDS	CP041215.1	287679	287314	-	366	Mobile element protein	- none -
fig|2591635.5.peg.331	CDS	CP041215.1	305256	305621	+	366	Mobile element protein	- none -
fig|2591635.5.peg.369	CDS	CP041215.1	339797	340111	+	315	Mobile element protein	- none -
fig|2591635.5.peg.370	CDS	CP041215.1	340181	341134	+	954	Mobile element protein	- none -
fig|2591635.5.peg.393	CDS	CP041215.1	374395	374769	+	375	Mobile element protein	- none -
fig|2591635.5.peg.394	CDS	CP041215.1	374821	375489	+	669	Mobile element protein	- none -
fig|2591635.5.peg.395	CDS	CP041215.1	375537	375665	+	129	Mobile element protein	- none -
fig|2591635.5.peg.397	CDS	CP041215.1	376428	376063	-	366	Mobile element protein	- none -
fig|2591635.5.peg.410	CDS	CP041215.1	385857	385492	-	366	Mobile element protein	- none -
fig|2591635.5.peg.411	CDS	CP041215.1	386001	385900	-	102	Mobile element protein	- none -
fig|2591635.5.peg.412	CDS	CP041215.1	386293	386081	-	213	Mobile element protein	- none -
fig|2591635.5.peg.413	CDS	CP041215.1	387359	386691	-	669	Mobile element protein	- none -
fig|2591635.5.peg.431	CDS	CP041215.1	406509	406144	-	366	Mobile element protein	- none -

fig|2591635.5.peg.434	CDS	CP041215.1	408304	408140	-	165	Mobile element protein	- none -
fig|2591635.5.peg.437	CDS	CP041215.1	409183	408881	-	303	Mobile element protein	- none -
fig|2591635.5.peg.443	CDS	CP041215.1	412418	412053	-	366	Mobile element protein	- none -
fig|2591635.5.peg.446	CDS	CP041215.1	413999	413634	-	366	Mobile element protein	- none -
fig|2591635.5.peg.448	CDS	CP041215.1	415537	415902	+	366	Mobile element protein	- none -
fig|2591635.5.peg.479	CDS	CP041215.1	443377	443742	+	366	Mobile element protein	- none -
fig|2591635.5.peg.483	CDS	CP041215.1	445690	445325	-	366	Mobile element protein	- none -
fig|2591635.5.peg.504	CDS	CP041215.1	460412	460714	+	303	Mobile element protein	- none -
fig|2591635.5.peg.507	CDS	CP041215.1	461291	461455	+	165	Mobile element protein	- none -
fig|2591635.5.peg.516	CDS	CP041215.1	470726	471154	+	429	Mobile element protein	- none -
fig|2591635.5.peg.517	CDS	CP041215.1	471637	471449	-	189	Mobile element protein	- none -
fig|2591635.5.peg.518	CDS	CP041215.1	471943	472890	+	948	Mobile element protein	- none -
fig|2591635.5.peg.528	CDS	CP041215.1	482617	482982	+	366	Mobile element protein	- none -
fig|2591635.5.peg.535	CDS	CP041215.1	488566	488931	+	366	Mobile element protein	- none -
fig|2591635.5.peg.537	CDS	CP041215.1	489465	489749	+	285	Mobile element protein	- none -
fig|2591635.5.peg.570	CDS	CP041215.1	519654	519289	-	366	Mobile element protein	- none -
fig|2591635.5.peg.573	CDS	CP041215.1	521550	521843	+	294	Mobile element protein	- none -
fig|2591635.5.peg.576	CDS	CP041215.1	522420	522584	+	165	Mobile element protein	- none -
fig|2591635.5.peg.606	CDS	CP041215.1	549419	549784	+	366	Mobile element protein	- none -
fig|2591635.5.peg.608	CDS	CP041215.1	550413	550192	-	222	Mobile element protein	- none -
fig|2591635.5.peg.646	CDS	CP041215.1	598063	597698	-	366	Mobile element protein	- none -
fig|2591635.5.peg.661	CDS	CP041215.1	612161	611901	-	261	Mobile element protein	- none -
fig|2591635.5.peg.681	CDS	CP041215.1	637982	638347	+	366	Mobile element protein	- none -

fig|2591635.5.peg.704	CDS	CP041215.1	661183	660818	-	366	Mobile element protein	- none -
fig|2591635.5.peg.720	CDS	CP041215.1	679773	679408	-	366	Mobile element protein	- none -
fig|2591635.5.peg.726	CDS	CP041215.1	684487	683333	-	1155	Mobile element protein	- none -
fig|2591635.5.peg.731	CDS	CP041215.1	688898	688614	-	285	Mobile element protein	- none -
fig|2591635.5.peg.742	CDS	CP041215.1	699152	699517	+	366	Mobile element protein	- none -
fig|2591635.5.peg.758	CDS	CP041215.1	723892	724257	+	366	Mobile element protein	- none -
fig|2591635.5.peg.760	CDS	CP041215.1	724793	724665	-	129	Mobile element protein	- none -
fig|2591635.5.peg.773	CDS	CP041215.1	733962	734327	+	366	Mobile element protein	- none -
fig|2591635.5.peg.775	CDS	CP041215.1	735459	734944	-	516	Mobile element protein	- none -
fig|2591635.5.peg.776	CDS	CP041215.1	735700	735449	-	252	Mobile element protein	- none -
fig|2591635.5.peg.824	CDS	CP041215.1	772655	772939	+	285	Mobile element protein	- none -
fig|2591635.5.peg.826	CDS	CP041215.1	773590	773955	+	366	Mobile element protein	- none -
fig|2591635.5.peg.833	CDS	CP041215.1	781559	781711	+	153	Mobile element protein	- none -
fig|2591635.5.peg.839	CDS	CP041215.1	785191	785475	+	285	Mobile element protein	- none -
fig|2591635.5.peg.847	CDS	CP041215.1	795122	794715	-	408	Mobile element protein	- none -
fig|2591635.5.peg.848	CDS	CP041215.1	795342	795142	-	201	Mobile element protein	- none -
fig|2591635.5.peg.849	CDS	CP041215.1	795655	795326	-	330	Mobile element protein	- none -
fig|2591635.5.peg.852	CDS	CP041215.1	798078	798443	+	366	Mobile element protein	- none -
fig|2591635.5.peg.871	CDS	CP041215.1	815731	815366	-	366	Mobile element protein	- none -
fig|2591635.5.peg.879	CDS	CP041215.1	823006	822641	-	366	Mobile element protein	- none -
fig|2591635.5.peg.892	CDS	CP041215.1	837236	836952	-	285	Mobile element protein	- none -
fig|2591635.5.peg.900	CDS	CP041215.1	843638	843934	+	297	Mobile element protein	- none -
fig|2591635.5.peg.901	CDS	CP041215.1	844127	844492	+	366	Mobile element protein	- none -

fig|2591635.5.peg.908	CDS	CP041215.1	849586	849221	-	366	Mobile element protein	- none -
fig|2591635.5.peg.910	CDS	CP041215.1	851497	851862	+	366	Mobile element protein	- none -
fig|2591635.5.peg.914	CDS	CP041215.1	855002	855271	+	270	Mobile element protein	- none -
fig|2591635.5.peg.921	CDS	CP041215.1	863125	863490	+	366	Mobile element protein	- none -
fig|2591635.5.peg.923	CDS	CP041215.1	863979	864344	+	366	Mobile element protein	- none -
fig|2591635.5.peg.931	CDS	CP041215.1	869247	869549	+	303	Mobile element protein	- none -
fig|2591635.5.peg.934	CDS	CP041215.1	870126	870290	+	165	Mobile element protein	- none -
fig|2591635.5.peg.962	CDS	CP041215.1	900311	900676	+	366	Mobile element protein	- none -
fig|2591635.5.peg.964	CDS	CP041215.1	902658	901315	-	1344	Mobile element protein	- none -
fig|2591635.5.peg.978	CDS	CP041215.1	914510	914875	+	366	Mobile element protein	- none -
fig|2591635.5.peg.1019	CDS	CP041215.1	958304	958597	+	294	Mobile element protein	- none -
fig|2591635.5.peg.1021	CDS	CP041215.1	958979	959230	+	252	Mobile element protein	- none -
fig|2591635.5.peg.1088	CDS	CP041215.1	1023095	1022730	-	366	Mobile element protein	- none -
fig|2591635.5.peg.1104	CDS	CP041215.1	1048366	1048124	-	243	Mobile element protein	- none -
fig|2591635.5.peg.1106	CDS	CP041215.1	1048684	1049049	+	366	Mobile element protein	- none -
fig|2591635.5.peg.1110	CDS	CP041215.1	1051302	1050937	-	366	Mobile element protein	- none -
fig|2591635.5.peg.1127	CDS	CP041215.1	1064148	1063915	-	234	Mobile element protein	- none -
fig|2591635.5.peg.1128	CDS	CP041215.1	1064248	1064613	+	366	Mobile element protein	- none -
fig|2591635.5.peg.1167	CDS	CP041215.1	1098415	1098720	+	306	Mobile element protein	- none -
fig|2591635.5.peg.1181	CDS	CP041215.1	1117546	1117418	-	129	Mobile element protein	- none -
fig|2591635.5.peg.1183	CDS	CP041215.1	1118468	1118184	-	285	Mobile element protein	- none -
fig|2591635.5.peg.1211	CDS	CP041215.1	1142602	1142474	-	129	Mobile element protein	- none -
fig|2591635.5.peg.1213	CDS	CP041215.1	1143524	1143240	-	285	Mobile element protein	- none -

fig|2591635.5.peg.1250	CDS	CP041215.1	1191920	1191555	-	366	Mobile element protein	- none -
fig|2591635.5.peg.1279	CDS	CP041215.1	1216896	1217261	+	366	Mobile element protein	- none -
fig|2591635.5.peg.1285	CDS	CP041215.1	1224736	1224101	-	636	Mobile element protein	- none -
fig|2591635.5.peg.1286	CDS	CP041215.1	1225049	1224720	-	330	Mobile element protein	- none -
fig|2591635.5.peg.1303	CDS	CP041215.1	1237452	1237087	-	366	Mobile element protein	- none -
fig|2591635.5.peg.1315	CDS	CP041215.1	1250757	1251041	+	285	Mobile element protein	- none -
fig|2591635.5.peg.1318	CDS	CP041215.1	1252076	1253230	+	1155	Mobile element protein	- none -
fig|2591635.5.peg.1335	CDS	CP041215.1	1267744	1267109	-	636	Mobile element protein	- none -
fig|2591635.5.peg.1336	CDS	CP041215.1	1268027	1267728	-	300	Mobile element protein	- none -
fig|2591635.5.peg.1351	CDS	CP041215.1	1287126	1286830	-	297	Mobile element protein	- none -
fig|2591635.5.peg.1352	CDS	CP041215.1	1287236	1287409	+	174	Mobile element protein	- none -
fig|2591635.5.peg.1354	CDS	CP041215.1	1287746	1288057	+	312	Mobile element protein	- none -
fig|2591635.5.peg.1356	CDS	CP041215.1	1288532	1288714	+	183	Mobile element protein	- none -
fig|2591635.5.peg.1357	CDS	CP041215.1	1288806	1289000	+	195	Mobile element protein	- none -
fig|2591635.5.peg.1359	CDS	CP041215.1	1289435	1289800	+	366	Mobile element protein	- none -
fig|2591635.5.peg.1372	CDS	CP041215.1	1301081	1301509	+	429	Mobile element protein	- none -
fig|2591635.5.peg.1373	CDS	CP041215.1	1301992	1301804	-	189	Mobile element protein	- none -
fig|2591635.5.peg.1374	CDS	CP041215.1	1302351	1302716	+	366	Mobile element protein	- none -
fig|2591635.5.peg.1379	CDS	CP041215.1	1304667	1304260	-	408	Mobile element protein	- none -
fig|2591635.5.peg.1380	CDS	CP041215.1	1304887	1304687	-	201	Mobile element protein	- none -
fig|2591635.5.peg.1381	CDS	CP041215.1	1305200	1304871	-	330	Mobile element protein	- none -
fig|2591635.5.peg.1408	CDS	CP041215.1	1338817	1338452	-	366	Mobile element protein	- none -
fig|2591635.5.peg.1412	CDS	CP041215.1	1340776	1340348	-	429	Mobile element protein	- none -

	fig|2591635.5.peg.1420	CDS	CP041215.1	1346824	1346630	-	195	Mobile element protein	- none -
	fig|2591635.5.peg.1421	CDS	CP041215.1	1347098	1346916	-	183	Mobile element protein	- none -
	fig|2591635.5.peg.1423	CDS	CP041215.1	1347851	1348216	+	366	Mobile element protein	- none -
	fig|2591635.5.peg.1433	CDS	CP041215.1	1353969	1354175	+	207	Mobile element protein	- none -
	fig|2591635.5.peg.1434	CDS	CP041215.1	1354293	1354676	+	384	Mobile element protein	- none -
	fig|2591635.5.peg.1453	CDS	CP041215.1	1378271	1377924	-	348	Mobile element protein	- none -
	fig|2591635.5.peg.1455	CDS	CP041215.1	1380538	1380350	-	189	Mobile element protein	- none -
	fig|2591635.5.peg.1458	CDS	CP041215.1	1381879	1382178	+	300	Mobile element protein	- none -
	fig|2591635.5.peg.1459	CDS	CP041215.1	1382162	1382797	+	636	Mobile element protein	- none -
	fig|2591635.5.peg.1479	CDS	CP041215.1	1400752	1400387	-	366	Mobile element protein	- none -
	fig|2591635.5.peg.1496	CDS	CP041215.1	1418544	1417909	-	636	Mobile element protein	- none -
	fig|2591635.5.peg.1497	CDS	CP041215.1	1418857	1418528	-	330	Mobile element protein	- none -
	fig|2591635.5.peg.1519	CDS	CP041215.1	1440382	1440017	-	366	Mobile element protein	- none -
wCfeJ	fig|212123.3.peg.15	CDS	CP051157.1	13901	13032	-	870	Mobile element protein	- none -
	fig|212123.3.peg.93	CDS	CP051157.1	89982	90935	+	954	Mobile element protein	- none -
	fig|212123.3.peg.269	CDS	CP051157.1	275016	275261	+	246	Mobile element protein	- none -
	fig|212123.3.peg.530	CDS	CP051157.1	539929	540117	+	189	Mobile element protein	- none -
	fig|212123.3.peg.547	CDS	CP051157.1	562196	562561	+	366	Mobile element protein	- none -
	fig|212123.3.peg.701	CDS	CP051157.1	716149	716298	+	150	Mobile element protein	- none -
	fig|212123.3.peg.905	CDS	CP051157.1	934347	934646	+	300	Mobile element protein	- none -
	fig|212123.3.peg.906	CDS	CP051157.1	934840	935007	+	168	Mobile element protein	- none -
	fig|212123.3.peg.909	CDS	CP051157.1	936536	937183	+	648	Mobile element protein	- none -
	fig|212123.3.peg.910	CDS	CP051157.1	937180	937440	+	261	Mobile element protein	- none -

	fig|212123.3.peg.1152	CDS	CP051157.1	1173814	1174230	+	417	Mobile element protein	- none -
wCfeT	fig|212123.4.peg.1	CDS	CP051156.1	531	139	-	393	Mobile element protein	- none -
	fig|212123.4.peg.3	CDS	CP051156.1	4905	3544	-	1362	Mobile element protein	- none -
	fig|212123.4.peg.8	CDS	CP051156.1	10203	9376	-	828	Mobile element protein	- none -
	fig|212123.4.peg.9	CDS	CP051156.1	10557	10345	-	213	Mobile element protein	- none -
	fig|212123.4.peg.30	CDS	CP051156.1	29990	29034	-	957	Mobile element protein	- none -
	fig|212123.4.peg.43	CDS	CP051156.1	41140	41370	+	231	Mobile element protein	- none -
	fig|212123.4.peg.48	CDS	CP051156.1	47068	45728	-	1341	Mobile element protein	- none -
	fig|212123.4.peg.75	CDS	CP051156.1	72746	71406	-	1341	Mobile element protein	- none -
	fig|212123.4.peg.79	CDS	CP051156.1	75727	76098	+	372	Mobile element protein	- none -
	fig|212123.4.peg.80	CDS	CP051156.1	76240	77067	+	828	Mobile element protein	- none -
	fig|212123.4.peg.93	CDS	CP051156.1	91154	90327	-	828	Mobile element protein	- none -
	fig|212123.4.peg.94	CDS	CP051156.1	91667	91296	-	372	Mobile element protein	- none -
	fig|212123.4.peg.108	CDS	CP051156.1	104468	103599	-	870	Mobile element protein	- none -
	fig|212123.4.peg.119	CDS	CP051156.1	112144	111275	-	870	Mobile element protein	- none -
	fig|212123.4.peg.130	CDS	CP051156.1	119820	118951	-	870	Mobile element protein	- none -
	fig|212123.4.peg.134	CDS	CP051156.1	123480	124091	+	612	Mobile element protein	- none -
	fig|212123.4.peg.135	CDS	CP051156.1	125053	124097	-	957	Mobile element protein	- none -
	fig|212123.4.peg.136	CDS	CP051156.1	125690	125319	-	372	Mobile element protein	- none -
	fig|212123.4.peg.178	CDS	CP051156.1	154323	155684	+	1362	Mobile element protein	- none -
	fig|212123.4.peg.183	CDS	CP051156.1	159234	160037	+	804	Mobile element protein	- none -
	fig|212123.4.peg.186	CDS	CP051156.1	160878	161048	+	171	Mobile element protein	- none -
	fig|212123.4.peg.207	CDS	CP051156.1	187656	188996	+	1341	Mobile element protein	- none -

fig|212123.4.peg.209	CDS	CP051156.1	191115	190159	-	957	Mobile element protein	- none -
fig|212123.4.peg.213	CDS	CP051156.1	194651	195991	+	1341	Mobile element protein	- none -
fig|212123.4.peg.215	CDS	CP051156.1	198936	197596	-	1341	Mobile element protein	- none -
fig|212123.4.peg.224	CDS	CP051156.1	211293	209953	-	1341	Mobile element protein	- none -
fig|212123.4.peg.225	CDS	CP051156.1	212788	211427	-	1362	Mobile element protein	- none -
fig|212123.4.peg.226	CDS	CP051156.1	214417	213056	-	1362	Mobile element protein	- none -
fig|212123.4.peg.235	CDS	CP051156.1	227384	226428	-	957	Mobile element protein	- none -
fig|212123.4.peg.237	CDS	CP051156.1	230591	231931	+	1341	Mobile element protein	- none -
fig|212123.4.peg.239	CDS	CP051156.1	233235	232309	-	927	Mobile element protein	- none -
fig|212123.4.peg.243	CDS	CP051156.1	238649	237693	-	957	Mobile element protein	- none -
fig|212123.4.peg.245	CDS	CP051156.1	242441	241101	-	1341	Mobile element protein	- none -
fig|212123.4.peg.264	CDS	CP051156.1	263363	264319	+	957	Mobile element protein	- none -
fig|212123.4.peg.289	CDS	CP051156.1	286594	285638	-	957	Mobile element protein	- none -
fig|212123.4.peg.291	CDS	CP051156.1	287718	288089	+	372	Mobile element protein	- none -
fig|212123.4.peg.292	CDS	CP051156.1	288231	289058	+	828	Mobile element protein	- none -
fig|212123.4.peg.296	CDS	CP051156.1	291867	293207	+	1341	Mobile element protein	- none -
fig|212123.4.peg.299	CDS	CP051156.1	295758	297098	+	1341	Mobile element protein	- none -
fig|212123.4.peg.321	CDS	CP051156.1	321273	320317	-	957	Mobile element protein	- none -
fig|212123.4.peg.324	CDS	CP051156.1	324435	323479	-	957	Mobile element protein	- none -
fig|212123.4.peg.327	CDS	CP051156.1	327731	326391	-	1341	Mobile element protein	- none -
fig|212123.4.peg.337	CDS	CP051156.1	337975	337817	-	159	Mobile element protein	- none -
fig|212123.4.peg.338	CDS	CP051156.1	338067	338438	+	372	Mobile element protein	- none -
fig|212123.4.peg.341	CDS	CP051156.1	339510	340265	+	756	Mobile element protein	- none -

fig|212123.4.peg.373	CDS	CP051156.1	370736	369375	-	1362	Mobile element protein	- none -
fig|212123.4.peg.377	CDS	CP051156.1	375660	376616	+	957	Mobile element protein	- none -
fig|212123.4.peg.379	CDS	CP051156.1	377056	378396	+	1341	Mobile element protein	- none -
fig|212123.4.peg.381	CDS	CP051156.1	378909	379865	+	957	Mobile element protein	- none -
fig|212123.4.peg.383	CDS	CP051156.1	382478	381423	-	1056	Mobile element protein	- none -
fig|212123.4.peg.384	CDS	CP051156.1	383869	382529	-	1341	Mobile element protein	- none -
fig|212123.4.peg.400	CDS	CP051156.1	400810	400007	-	804	Mobile element protein	- none -
fig|212123.4.peg.401	CDS	CP051156.1	401323	400952	-	372	Mobile element protein	- none -
fig|212123.4.peg.420	CDS	CP051156.1	417498	418838	+	1341	Mobile element protein	- none -
fig|212123.4.peg.423	CDS	CP051156.1	420909	420709	-	201	Mobile element protein	- none -
fig|212123.4.peg.430	CDS	CP051156.1	430156	428795	-	1362	Mobile element protein	- none -
fig|212123.4.peg.435	CDS	CP051156.1	435523	434609	-	915	Mobile element protein	- none -
fig|212123.4.peg.438	CDS	CP051156.1	436589	437929	+	1341	Mobile element protein	- none -
fig|212123.4.peg.442	CDS	CP051156.1	441077	439737	-	1341	Mobile element protein	- none -
fig|212123.4.peg.443	CDS	CP051156.1	441285	442241	+	957	Mobile element protein	- none -
fig|212123.4.peg.456	CDS	CP051156.1	455453	456409	+	957	Mobile element protein	- none -
fig|212123.4.peg.461	CDS	CP051156.1	463678	462338	-	1341	Mobile element protein	- none -
fig|212123.4.peg.464	CDS	CP051156.1	467059	465743	-	1317	Mobile element protein	- none -
fig|212123.4.peg.479	CDS	CP051156.1	480399	479572	-	828	Mobile element protein	- none -
fig|212123.4.peg.480	CDS	CP051156.1	480912	480541	-	372	Mobile element protein	- none -
fig|212123.4.peg.483	CDS	CP051156.1	485154	484198	-	957	Mobile element protein	- none -
fig|212123.4.peg.487	CDS	CP051156.1	490628	489672	-	957	Mobile element protein	- none -
fig|212123.4.peg.495	CDS	CP051156.1	494934	496274	+	1341	Mobile element protein	- none -

fig|212123.4.peg.506	CDS	CP051156.1	505358	504684	-	675	Mobile element protein	- none -
fig|212123.4.peg.507	CDS	CP051156.1	505871	505500	-	372	Mobile element protein	- none -
fig|212123.4.peg.530	CDS	CP051156.1	533698	532742	-	957	Mobile element protein	- none -
fig|212123.4.peg.537	CDS	CP051156.1	539018	538191	-	828	Mobile element protein	- none -
fig|212123.4.peg.538	CDS	CP051156.1	539531	539160	-	372	Mobile element protein	- none -
fig|212123.4.peg.543	CDS	CP051156.1	542537	543877	+	1341	Mobile element protein	- none -
fig|212123.4.peg.548	CDS	CP051156.1	548794	547607	-	1188	Mobile element protein	- none -
fig|212123.4.peg.551	CDS	CP051156.1	550679	549723	-	957	Mobile element protein	- none -
fig|212123.4.peg.556	CDS	CP051156.1	556041	556997	+	957	Mobile element protein	- none -
fig|212123.4.peg.557	CDS	CP051156.1	558424	557084	-	1341	Mobile element protein	- none -
fig|212123.4.peg.571	CDS	CP051156.1	572896	572165	-	732	Mobile element protein	- none -
fig|212123.4.peg.572	CDS	CP051156.1	573409	573038	-	372	Mobile element protein	- none -
fig|212123.4.peg.580	CDS	CP051156.1	581602	582942	+	1341	Mobile element protein	- none -
fig|212123.4.peg.592	CDS	CP051156.1	595051	595422	+	372	Mobile element protein	- none -
fig|212123.4.peg.593	CDS	CP051156.1	595564	596391	+	828	Mobile element protein	- none -
fig|212123.4.peg.594	CDS	CP051156.1	596551	596378	-	174	Mobile element protein	- none -
fig|212123.4.peg.599	CDS	CP051156.1	599055	599426	+	372	Mobile element protein	- none -
fig|212123.4.peg.600	CDS	CP051156.1	599568	600395	+	828	Mobile element protein	- none -
fig|212123.4.peg.604	CDS	CP051156.1	603636	602680	-	957	Mobile element protein	- none -
fig|212123.4.peg.630	CDS	CP051156.1	628914	630254	+	1341	Mobile element protein	- none -
fig|212123.4.peg.631	CDS	CP051156.1	631507	630251	-	1257	Mobile element protein	- none -
fig|212123.4.peg.637	CDS	CP051156.1	639236	638052	-	1185	Mobile element protein	- none -
fig|212123.4.peg.644	CDS	CP051156.1	646539	645583	-	957	Mobile element protein	- none -

fig|212123.4.peg.647	CDS	CP051156.1	647873	648460	+	588	Mobile element protein	- none -
fig|212123.4.peg.687	CDS	CP051156.1	688045	687089	-	957	Mobile element protein	- none -
fig|212123.4.peg.690	CDS	CP051156.1	692253	690913	-	1341	Mobile element protein	- none -
fig|212123.4.peg.697	CDS	CP051156.1	700240	698879	-	1362	Mobile element protein	- none -
fig|212123.4.peg.698	CDS	CP051156.1	701545	700589	-	957	Mobile element protein	- none -
fig|212123.4.peg.700	CDS	CP051156.1	702474	703814	+	1341	Mobile element protein	- none -
fig|212123.4.peg.706	CDS	CP051156.1	708547	709503	+	957	Mobile element protein	- none -
fig|212123.4.peg.709	CDS	CP051156.1	711654	712610	+	957	Mobile element protein	- none -
fig|212123.4.peg.714	CDS	CP051156.1	716733	715393	-	1341	Mobile element protein	- none -
fig|212123.4.peg.737	CDS	CP051156.1	743664	744023	+	360	Mobile element protein	- none -
fig|212123.4.peg.738	CDS	CP051156.1	744165	744992	+	828	Mobile element protein	- none -
fig|212123.4.peg.751	CDS	CP051156.1	762080	762451	+	372	Mobile element protein	- none -
fig|212123.4.peg.752	CDS	CP051156.1	762593	763420	+	828	Mobile element protein	- none -
fig|212123.4.peg.758	CDS	CP051156.1	767626	768966	+	1341	Mobile element protein	- none -
fig|212123.4.peg.762	CDS	CP051156.1	773298	773576	+	279	Mobile element protein	- none -
fig|212123.4.peg.763	CDS	CP051156.1	774538	773582	-	957	Mobile element protein	- none -
fig|212123.4.peg.787	CDS	CP051156.1	795485	796123	+	639	Mobile element protein	- none -
fig|212123.4.peg.793	CDS	CP051156.1	801938	800598	-	1341	Mobile element protein	- none -
fig|212123.4.peg.799	CDS	CP051156.1	809694	808738	-	957	Mobile element protein	- none -
fig|212123.4.peg.800	CDS	CP051156.1	809735	810556	+	822	Mobile element protein	- none -
fig|212123.4.peg.804	CDS	CP051156.1	813372	813743	+	372	Mobile element protein	- none -
fig|212123.4.peg.806	CDS	CP051156.1	814126	814713	+	588	Mobile element protein	- none -
fig|212123.4.peg.825	CDS	CP051156.1	831373	832329	+	957	Mobile element protein	- none -

fig|212123.4.peg.827	CDS	CP051156.1	832769	834109	+	1341	Mobile element protein	- none -
fig|212123.4.peg.835	CDS	CP051156.1	840507	841463	+	957	Mobile element protein	- none -
fig|212123.4.peg.837	CDS	CP051156.1	841717	842304	+	588	Mobile element protein	- none -
fig|212123.4.peg.838	CDS	CP051156.1	843225	842284	-	942	Mobile element protein	- none -
fig|212123.4.peg.839	CDS	CP051156.1	843402	843632	+	231	Mobile element protein	- none -
fig|212123.4.peg.842	CDS	CP051156.1	847093	845753	-	1341	Mobile element protein	- none -
fig|212123.4.peg.848	CDS	CP051156.1	853107	851767	-	1341	Mobile element protein	- none -
fig|212123.4.peg.856	CDS	CP051156.1	860154	859198	-	957	Mobile element protein	- none -
fig|212123.4.peg.861	CDS	CP051156.1	866387	867343	+	957	Mobile element protein	- none -
fig|212123.4.peg.870	CDS	CP051156.1	879404	878091	-	1314	Mobile element protein	- none -
fig|212123.4.peg.879	CDS	CP051156.1	887615	886659	-	957	Mobile element protein	- none -
fig|212123.4.peg.880	CDS	CP051156.1	888082	889422	+	1341	Mobile element protein	- none -
fig|212123.4.peg.890	CDS	CP051156.1	898806	899177	+	372	Mobile element protein	- none -
fig|212123.4.peg.898	CDS	CP051156.1	906081	905125	-	957	Mobile element protein	- none -
fig|212123.4.peg.906	CDS	CP051156.1	915870	914530	-	1341	Mobile element protein	- none -
fig|212123.4.peg.908	CDS	CP051156.1	917266	916310	-	957	Mobile element protein	- none -
fig|212123.4.peg.922	CDS	CP051156.1	930706	929366	-	1341	Mobile element protein	- none -
fig|212123.4.peg.925	CDS	CP051156.1	932288	933628	+	1341	Mobile element protein	- none -
fig|212123.4.peg.940	CDS	CP051156.1	945097	944510	-	588	Mobile element protein	- none -
fig|212123.4.peg.947	CDS	CP051156.1	950143	949187	-	957	Mobile element protein	- none -
fig|212123.4.peg.964	CDS	CP051156.1	967780	968736	+	957	Mobile element protein	- none -
fig|212123.4.peg.968	CDS	CP051156.1	970958	972298	+	1341	Mobile element protein	- none -
fig|212123.4.peg.990	CDS	CP051156.1	993516	992560	-	957	Mobile element protein	- none -

fig|212123.4.peg.997	CDS	CP051156.1	999100	1000440	+	1341	Mobile element protein	- none -
fig|212123.4.peg.998	CDS	CP051156.1	1000539	1001879	+	1341	Mobile element protein	- none -
fig|212123.4.peg.1004	CDS	CP051156.1	1007115	1006159	-	957	Mobile element protein	- none -
fig|212123.4.peg.1052	CDS	CP051156.1	1059547	1058591	-	957	Mobile element protein	- none -
fig|212123.4.peg.1056	CDS	CP051156.1	1062311	1062682	+	372	Mobile element protein	- none -
fig|212123.4.peg.1057	CDS	CP051156.1	1062824	1063651	+	828	Mobile element protein	- none -
fig|212123.4.peg.1077	CDS	CP051156.1	1083495	1082539	-	957	Mobile element protein	- none -
fig|212123.4.peg.1080	CDS	CP051156.1	1087812	1088042	+	231	Mobile element protein	- none -
fig|212123.4.peg.1082	CDS	CP051156.1	1089376	1088420	-	957	Mobile element protein	- none -
fig|212123.4.peg.1102	CDS	CP051156.1	1119077	1117737	-	1341	Mobile element protein	- none -
fig|212123.4.peg.1126	CDS	CP051156.1	1141921	1142658	+	738	Mobile element protein	- none -
fig|212123.4.peg.1127	CDS	CP051156.1	1142625	1142876	+	252	Mobile element protein	- none -
fig|212123.4.peg.1135	CDS	CP051156.1	1151598	1150642	-	957	Mobile element protein	- none -
fig|212123.4.peg.1140	CDS	CP051156.1	1155674	1157014	+	1341	Mobile element protein	- none -
fig|212123.4.peg.1146	CDS	CP051156.1	1160219	1161256	+	1038	Mobile element protein	- none -
fig|212123.4.peg.1147	CDS	CP051156.1	1161329	1161559	+	231	Mobile element protein	- none -
fig|212123.4.peg.1166	CDS	CP051156.1	1180793	1180563	-	231	Mobile element protein	- none -
fig|212123.4.peg.1192	CDS	CP051156.1	1201427	1201657	+	231	Mobile element protein	- none -
fig|212123.4.peg.1233	CDS	CP051156.1	1234458	1233118	-	1341	Mobile element protein	- none -
fig|212123.4.peg.1237	CDS	CP051156.1	1239445	1240785	+	1341	Mobile element protein	- none -
fig|212123.4.peg.1248	CDS	CP051156.1	1249535	1250875	+	1341	Mobile element protein	- none -
fig|212123.4.peg.1254	CDS	CP051156.1	1257621	1256281	-	1341	Mobile element protein	- none -
fig|212123.4.peg.1271	CDS	CP051156.1	1276151	1275195	-	957	Mobile element protein	- none -

fig|212123.4.peg.1272	CDS	CP051156.1	1276172	1276477	+	306	Mobile element protein	- none -
fig|212123.4.peg.1274	CDS	CP051156.1	1276808	1277446	+	639	Mobile element protein	- none -
fig|212123.4.peg.1280	CDS	CP051156.1	1284273	1285613	+	1341	Mobile element protein	- none -
fig|212123.4.peg.1284	CDS	CP051156.1	1289018	1288146	-	873	Mobile element protein	- none -
fig|212123.4.peg.1289	CDS	CP051156.1	1295154	1294198	-	957	Mobile element protein	- none -
fig|212123.4.peg.1293	CDS	CP051156.1	1296985	1296347	-	639	Mobile element protein	- none -
fig|212123.4.peg.1294	CDS	CP051156.1	1297687	1296989	-	699	Mobile element protein	- none -
fig|212123.4.peg.1302	CDS	CP051156.1	1304851	1304213	-	639	Mobile element protein	- none -
fig|212123.4.peg.1307	CDS	CP051156.1	1309594	1308767	-	828	Mobile element protein	- none -
fig|212123.4.peg.1308	CDS	CP051156.1	1310041	1309736	-	306	Mobile element protein	- none -
fig|212123.4.peg.1309	CDS	CP051156.1	1310062	1311018	+	957	Mobile element protein	- none -
fig|212123.4.peg.1315	CDS	CP051156.1	1315810	1316181	+	372	Mobile element protein	- none -
fig|212123.4.peg.1316	CDS	CP051156.1	1316323	1317150	+	828	Mobile element protein	- none -
fig|212123.4.peg.1327	CDS	CP051156.1	1326222	1325395	-	828	Mobile element protein	- none -
fig|212123.4.peg.1328	CDS	CP051156.1	1326576	1326364	-	213	Mobile element protein	- none -
fig|212123.4.peg.1334	CDS	CP051156.1	1331433	1330795	-	639	Mobile element protein	- none -
fig|212123.4.peg.1336	CDS	CP051156.1	1332102	1331764	-	339	Mobile element protein	- none -
fig|212123.4.peg.1344	CDS	CP051156.1	1341193	1340366	-	828	Mobile element protein	- none -
fig|212123.4.peg.1345	CDS	CP051156.1	1341706	1341335	-	372	Mobile element protein	- none -
fig|212123.4.peg.1353	CDS	CP051156.1	1347441	1346614	-	828	Mobile element protein	- none -
fig|212123.4.peg.1354	CDS	CP051156.1	1347934	1347590	-	345	Mobile element protein	- none -
fig|212123.4.peg.1357	CDS	CP051156.1	1348703	1350043	+	1341	Mobile element protein	- none -
fig|212123.4.peg.1366	CDS	CP051156.1	1358646	1357819	-	828	Mobile element protein	- none -

	fig|212123.4.peg.1367	CDS	CP051156.1	1359159	1358788	-	372	Mobile element protein	- none -
	fig|212123.4.peg.1397	CDS	CP051156.1	1383513	1382686	-	828	Mobile element protein	- none -
	fig|212123.4.peg.1398	CDS	CP051156.1	1384026	1383655	-	372	Mobile element protein	- none -
	fig|212123.4.peg.1404	CDS	CP051156.1	1389956	1389207	-	750	Mobile element protein	- none -
	fig|212123.4.peg.1461	CDS	CP051156.1	1441907	1443268	+	1362	Mobile element protein	- none -
	fig|212123.4.peg.1462	CDS	CP051156.1	1443346	1444692	+	1347	Mobile element protein	- none -
	fig|212123.4.peg.1469	CDS	CP051156.1	1449907	1450863	+	957	Mobile element protein	- none -
	fig|212123.4.peg.1474	CDS	CP051156.1	1455126	1455497	+	372	Mobile element protein	- none -
	fig|212123.4.peg.1475	CDS	CP051156.1	1455639	1456466	+	828	Mobile element protein	- none -
	fig|212123.4.peg.1489	CDS	CP051156.1	1473809	1474765	+	957	Mobile element protein	- none -
	fig|212123.4.peg.1495	CDS	CP051156.1	1481119	1479779	-	1341	Mobile element protein	- none -
	fig|212123.4.peg.1502	CDS	CP051156.1	1489851	1488895	-	957	Mobile element protein	- none -
	fig|212123.4.peg.1508	CDS	CP051156.1	1494125	1494721	+	597	Mobile element protein	- none -
	fig|212123.4.peg.1509	CDS	CP051156.1	1495538	1494708	-	831	Mobile element protein	- none -
wCle	fig|246273.9.peg.13	CDS	wCle_NZ_AP013028	13005	13319	+	315	Mobile element protein	- none -
	fig|246273.9.peg.14	CDS	wCle_NZ_AP013028	13438	13794	+	357	Mobile element protein	- none -
	fig|246273.9.peg.21	CDS	wCle_NZ_AP013028	26655	26299	-	357	Mobile element protein	- none -
	fig|246273.9.peg.22	CDS	wCle_NZ_AP013028	27088	26774	-	315	Mobile element protein	- none -
	fig|246273.9.peg.50	CDS	wCle_NZ_AP013028	51176	50754	-	423	Mobile element protein	- none -
	fig|246273.9.peg.51	CDS	wCle_NZ_AP013028	51307	51621	+	315	Mobile element protein	- none -
	fig|246273.9.peg.52	CDS	wCle_NZ_AP013028	51633	52097	+	465	Mobile element protein	- none -
	fig|246273.9.peg.53	CDS	wCle_NZ_AP013028	52366	52094	-	273	Mobile element protein	- none -
	fig|246273.9.peg.76	CDS	wCle_NZ_AP013028	78119	77655	-	465	Mobile element protein	- none -

fig|246273.9.peg.77	CDS	wCle_NZ_AP013028	78445	78131	-	315	Mobile element protein	- none -
fig|246273.9.peg.109	CDS	wCle_NZ_AP013028	105865	106329	+	465	Mobile element protein	- none -
fig|246273.9.peg.131	CDS	wCle_NZ_AP013028	127772	128086	+	315	Mobile element protein	- none -
fig|246273.9.peg.132	CDS	wCle_NZ_AP013028	128205	128612	+	408	Mobile element protein	- none -
fig|246273.9.peg.145	CDS	wCle_NZ_AP013028	137718	138074	+	357	Mobile element protein	- none -
fig|246273.9.peg.147	CDS	wCle_NZ_AP013028	139024	139338	+	315	Mobile element protein	- none -
fig|246273.9.peg.148	CDS	wCle_NZ_AP013028	139457	139813	+	357	Mobile element protein	- none -
fig|246273.9.peg.156	CDS	wCle_NZ_AP013028	149781	150095	+	315	Mobile element protein	- none -
fig|246273.9.peg.157	CDS	wCle_NZ_AP013028	150214	150570	+	357	Mobile element protein	- none -
fig|246273.9.peg.166	CDS	wCle_NZ_AP013028	157008	157364	+	357	Mobile element protein	- none -
fig|246273.9.peg.185	CDS	wCle_NZ_AP013028	178293	177829	-	465	Mobile element protein	- none -
fig|246273.9.peg.186	CDS	wCle_NZ_AP013028	178619	178305	-	315	Mobile element protein	- none -
fig|246273.9.peg.194	CDS	wCle_NZ_AP013028	186666	186980	+	315	Mobile element protein	- none -
fig|246273.9.peg.195	CDS	wCle_NZ_AP013028	186992	187456	+	465	Mobile element protein	- none -
fig|246273.9.peg.199	CDS	wCle_NZ_AP013028	189861	189397	-	465	Mobile element protein	- none -
fig|246273.9.peg.200	CDS	wCle_NZ_AP013028	190187	189873	-	315	Mobile element protein	- none -
fig|246273.9.peg.204	CDS	wCle_NZ_AP013028	192383	192697	+	315	Mobile element protein	- none -
fig|246273.9.peg.205	CDS	wCle_NZ_AP013028	192816	193172	+	357	Mobile element protein	- none -
fig|246273.9.peg.219	CDS	wCle_NZ_AP013028	205679	205218	-	462	Mobile element protein	- none -
fig|246273.9.peg.220	CDS	wCle_NZ_AP013028	206005	205691	-	315	Mobile element protein	- none -
fig|246273.9.peg.262	CDS	wCle_NZ_AP013028	233485	233799	+	315	Mobile element protein	- none -
fig|246273.9.peg.263	CDS	wCle_NZ_AP013028	233811	234173	+	363	Mobile element protein	- none -
fig|246273.9.peg.267	CDS	wCle_NZ_AP013028	236273	235917	-	357	Mobile element protein	- none -

fig|246273.9.peg.268	CDS	wCle_NZ_AP013028	236706	236392	-	315	Mobile element protein	- none -
fig|246273.9.peg.272	CDS	wCle_NZ_AP013028	237804	238118	+	315	Mobile element protein	- none -
fig|246273.9.peg.273	CDS	wCle_NZ_AP013028	238237	238593	+	357	Mobile element protein	- none -
fig|246273.9.peg.274	CDS	wCle_NZ_AP013028	239251	238895	-	357	Mobile element protein	- none -
fig|246273.9.peg.275	CDS	wCle_NZ_AP013028	239684	239370	-	315	Mobile element protein	- none -
fig|246273.9.peg.293	CDS	wCle_NZ_AP013028	258275	257919	-	357	Mobile element protein	- none -
fig|246273.9.peg.294	CDS	wCle_NZ_AP013028	258708	258394	-	315	Mobile element protein	- none -
fig|246273.9.peg.301	CDS	wCle_NZ_AP013028	265341	264985	-	357	Mobile element protein	- none -
fig|246273.9.peg.302	CDS	wCle_NZ_AP013028	265774	265460	-	315	Mobile element protein	- none -
fig|246273.9.peg.319	CDS	wCle_NZ_AP013028	282111	281755	-	357	Mobile element protein	- none -
fig|246273.9.peg.320	CDS	wCle_NZ_AP013028	282544	282335	-	210	Mobile element protein	- none -
fig|246273.9.peg.323	CDS	wCle_NZ_AP013028	285184	284828	-	357	Mobile element protein	- none -
fig|246273.9.peg.324	CDS	wCle_NZ_AP013028	285619	285305	-	315	Mobile element protein	- none -
fig|246273.9.peg.331	CDS	wCle_NZ_AP013028	292811	292347	-	465	Mobile element protein	- none -
fig|246273.9.peg.332	CDS	wCle_NZ_AP013028	292942	293256	+	315	Mobile element protein	- none -
fig|246273.9.peg.333	CDS	wCle_NZ_AP013028	293375	293731	+	357	Mobile element protein	- none -
fig|246273.9.peg.334	CDS	wCle_NZ_AP013028	294000	293728	-	273	Mobile element protein	- none -
fig|246273.9.peg.343	CDS	wCle_NZ_AP013028	300242	299886	-	357	Mobile element protein	- none -
fig|246273.9.peg.344	CDS	wCle_NZ_AP013028	300675	300361	-	315	Mobile element protein	- none -
fig|246273.9.peg.349	CDS	wCle_NZ_AP013028	306943	307152	+	210	Mobile element protein	- none -
fig|246273.9.peg.350	CDS	wCle_NZ_AP013028	307269	307733	+	465	Mobile element protein	- none -
fig|246273.9.peg.378	CDS	wCle_NZ_AP013028	338132	337776	-	357	Mobile element protein	- none -
fig|246273.9.peg.379	CDS	wCle_NZ_AP013028	338565	338251	-	315	Mobile element protein	- none -

fig|246273.9.peg.389	CDS	wCle_NZ_AP013028	350581	350895	+	315	Mobile element protein	- none -
fig|246273.9.peg.390	CDS	wCle_NZ_AP013028	351016	351372	+	357	Mobile element protein	- none -
fig|246273.9.peg.394	CDS	wCle_NZ_AP013028	355568	355212	-	357	Mobile element protein	- none -
fig|246273.9.peg.395	CDS	wCle_NZ_AP013028	356001	355687	-	315	Mobile element protein	- none -
fig|246273.9.peg.397	CDS	wCle_NZ_AP013028	356563	356877	+	315	Mobile element protein	- none -
fig|246273.9.peg.398	CDS	wCle_NZ_AP013028	356996	357352	+	357	Mobile element protein	- none -
fig|246273.9.peg.400	CDS	wCle_NZ_AP013028	360282	359926	-	357	Mobile element protein	- none -
fig|246273.9.peg.401	CDS	wCle_NZ_AP013028	360715	360401	-	315	Mobile element protein	- none -
fig|246273.9.peg.409	CDS	wCle_NZ_AP013028	370459	369995	-	465	Mobile element protein	- none -
fig|246273.9.peg.410	CDS	wCle_NZ_AP013028	370785	370576	-	210	Mobile element protein	- none -
fig|246273.9.peg.430	CDS	wCle_NZ_AP013028	395841	396113	+	273	Mobile element protein	- none -
fig|246273.9.peg.431	CDS	wCle_NZ_AP013028	396253	396110	-	144	Mobile element protein	- none -
fig|246273.9.peg.432	CDS	wCle_NZ_AP013028	396573	396211	-	363	Mobile element protein	- none -
fig|246273.9.peg.433	CDS	wCle_NZ_AP013028	396899	396585	-	315	Mobile element protein	- none -
fig|246273.9.peg.434	CDS	wCle_NZ_AP013028	397137	397493	+	357	Mobile element protein	- none -
fig|246273.9.peg.435	CDS	wCle_NZ_AP013028	397933	398304	+	372	Mobile element protein	- none -
fig|246273.9.peg.436	CDS	wCle_NZ_AP013028	398320	398802	+	483	Mobile element protein	- none -
fig|246273.9.peg.444	CDS	wCle_NZ_AP013028	406327	405845	-	483	Mobile element protein	- none -
fig|246273.9.peg.445	CDS	wCle_NZ_AP013028	406714	406343	-	372	Mobile element protein	- none -
fig|246273.9.peg.448	CDS	wCle_NZ_AP013028	407857	408171	+	315	Mobile element protein	- none -
fig|246273.9.peg.449	CDS	wCle_NZ_AP013028	408290	408646	+	357	Mobile element protein	- none -
fig|246273.9.peg.459	CDS	wCle_NZ_AP013028	416874	417188	+	315	Mobile element protein	- none -
fig|246273.9.peg.460	CDS	wCle_NZ_AP013028	417307	417663	+	357	Mobile element protein	- none -

fig|246273.9.peg.471	CDS	wCle_NZ_AP013028	430526	430882	+	357	Mobile element protein	- none -
fig|246273.9.peg.480	CDS	wCle_NZ_AP013028	439007	439183	+	177	Mobile element protein	- none -
fig|246273.9.peg.482	CDS	wCle_NZ_AP013028	440419	439955	-	465	Mobile element protein	- none -
fig|246273.9.peg.483	CDS	wCle_NZ_AP013028	440745	440431	-	315	Mobile element protein	- none -
fig|246273.9.peg.486	CDS	wCle_NZ_AP013028	443059	443373	+	315	Mobile element protein	- none -
fig|246273.9.peg.487	CDS	wCle_NZ_AP013028	443385	443849	+	465	Mobile element protein	- none -
fig|246273.9.peg.490	CDS	wCle_NZ_AP013028	445312	445626	+	315	Mobile element protein	- none -
fig|246273.9.peg.491	CDS	wCle_NZ_AP013028	445638	446012	+	375	Mobile element protein	- none -
fig|246273.9.peg.492	CDS	wCle_NZ_AP013028	446473	446009	-	465	Mobile element protein	- none -
fig|246273.9.peg.493	CDS	wCle_NZ_AP013028	446799	446485	-	315	Mobile element protein	- none -
fig|246273.9.peg.505	CDS	wCle_NZ_AP013028	452208	452522	+	315	Mobile element protein	- none -
fig|246273.9.peg.506	CDS	wCle_NZ_AP013028	452643	452999	+	357	Mobile element protein	- none -
fig|246273.9.peg.543	CDS	wCle_NZ_AP013028	488634	488170	-	465	Mobile element protein	- none -
fig|246273.9.peg.544	CDS	wCle_NZ_AP013028	488960	488646	-	315	Mobile element protein	- none -
fig|246273.9.peg.546	CDS	wCle_NZ_AP013028	489839	489696	-	144	Mobile element protein	- none -
fig|246273.9.peg.547	CDS	wCle_NZ_AP013028	490159	489797	-	363	Mobile element protein	- none -
fig|246273.9.peg.548	CDS	wCle_NZ_AP013028	490485	490171	-	315	Mobile element protein	- none -
fig|246273.9.peg.553	CDS	wCle_NZ_AP013028	494223	493570	-	654	Mobile element protein	- none -
fig|246273.9.peg.554	CDS	wCle_NZ_AP013028	494584	494255	-	330	Mobile element protein	- none -
fig|246273.9.peg.555	CDS	wCle_NZ_AP013028	494822	495178	+	357	Mobile element protein	- none -
fig|246273.9.peg.576	CDS	wCle_NZ_AP013028	510286	510600	+	315	Mobile element protein	- none -
fig|246273.9.peg.577	CDS	wCle_NZ_AP013028	510719	511075	+	357	Mobile element protein	- none -
fig|246273.9.peg.592	CDS	wCle_NZ_AP013028	526798	527112	+	315	Mobile element protein	- none -

fig|246273.9.peg.593	CDS	wCle_NZ_AP013028	527231	527587	+	357	Mobile element protein	- none -
fig|246273.9.peg.615	CDS	wCle_NZ_AP013028	545423	545067	-	357	Mobile element protein	- none -
fig|246273.9.peg.616	CDS	wCle_NZ_AP013028	545856	545542	-	315	Mobile element protein	- none -
fig|246273.9.peg.625	CDS	wCle_NZ_AP013028	554222	553866	-	357	Mobile element protein	- none -
fig|246273.9.peg.626	CDS	wCle_NZ_AP013028	554655	554341	-	315	Mobile element protein	- none -
fig|246273.9.peg.635	CDS	wCle_NZ_AP013028	561106	561420	+	315	Mobile element protein	- none -
fig|246273.9.peg.636	CDS	wCle_NZ_AP013028	561432	561896	+	465	Mobile element protein	- none -
fig|246273.9.peg.650	CDS	wCle_NZ_AP013028	574601	574915	+	315	Mobile element protein	- none -
fig|246273.9.peg.651	CDS	wCle_NZ_AP013028	575034	575390	+	357	Mobile element protein	- none -
fig|246273.9.peg.652	CDS	wCle_NZ_AP013028	575659	575387	-	273	Mobile element protein	- none -
fig|246273.9.peg.661	CDS	wCle_NZ_AP013028	583494	583030	-	465	Mobile element protein	- none -
fig|246273.9.peg.662	CDS	wCle_NZ_AP013028	583820	583506	-	315	Mobile element protein	- none -
fig|246273.9.peg.664	CDS	wCle_NZ_AP013028	584419	584733	+	315	Mobile element protein	- none -
fig|246273.9.peg.665	CDS	wCle_NZ_AP013028	584852	585208	+	357	Mobile element protein	- none -
fig|246273.9.peg.699	CDS	wCle_NZ_AP013028	622093	622407	+	315	Mobile element protein	- none -
fig|246273.9.peg.700	CDS	wCle_NZ_AP013028	622419	622883	+	465	Mobile element protein	- none -
fig|246273.9.peg.707	CDS	wCle_NZ_AP013028	627489	627133	-	357	Mobile element protein	- none -
fig|246273.9.peg.708	CDS	wCle_NZ_AP013028	627922	627608	-	315	Mobile element protein	- none -
fig|246273.9.peg.717	CDS	wCle_NZ_AP013028	637381	637695	+	315	Mobile element protein	- none -
fig|246273.9.peg.718	CDS	wCle_NZ_AP013028	637814	638170	+	357	Mobile element protein	- none -
fig|246273.9.peg.746	CDS	wCle_NZ_AP013028	662977	662513	-	465	Mobile element protein	- none -
fig|246273.9.peg.747	CDS	wCle_NZ_AP013028	663303	662989	-	315	Mobile element protein	- none -
fig|246273.9.peg.765	CDS	wCle_NZ_AP013028	680675	680319	-	357	Mobile element protein	- none -

fig|246273.9.peg.766	CDS	wCle_NZ_AP013028	681110	680796	-	315	Mobile element protein	- none -
fig|246273.9.peg.774	CDS	wCle_NZ_AP013028	684882	685196	+	315	Mobile element protein	- none -
fig|246273.9.peg.775	CDS	wCle_NZ_AP013028	685208	685672	+	465	Mobile element protein	- none -
fig|246273.9.peg.777	CDS	wCle_NZ_AP013028	687491	687135	-	357	Mobile element protein	- none -
fig|246273.9.peg.778	CDS	wCle_NZ_AP013028	687926	687612	-	315	Mobile element protein	- none -
fig|246273.9.peg.783	CDS	wCle_NZ_AP013028	692034	692348	+	315	Mobile element protein	- none -
fig|246273.9.peg.784	CDS	wCle_NZ_AP013028	692467	692823	+	357	Mobile element protein	- none -
fig|246273.9.peg.791	CDS	wCle_NZ_AP013028	695944	696258	+	315	Mobile element protein	- none -
fig|246273.9.peg.792	CDS	wCle_NZ_AP013028	696270	696734	+	465	Mobile element protein	- none -
fig|246273.9.peg.815	CDS	wCle_NZ_AP013028	718850	718386	-	465	Mobile element protein	- none -
fig|246273.9.peg.816	CDS	wCle_NZ_AP013028	719176	718862	-	315	Mobile element protein	- none -
fig|246273.9.peg.833	CDS	wCle_NZ_AP013028	738398	738670	+	273	Mobile element protein	- none -
fig|246273.9.peg.834	CDS	wCle_NZ_AP013028	739023	738667	-	357	Mobile element protein	- none -
fig|246273.9.peg.835	CDS	wCle_NZ_AP013028	739456	739142	-	315	Mobile element protein	- none -
fig|246273.9.peg.859	CDS	wCle_NZ_AP013028	768349	768098	-	252	Mobile element protein	- none -
fig|246273.9.peg.860	CDS	wCle_NZ_AP013028	768782	768468	-	315	Mobile element protein	- none -
fig|246273.9.peg.868	CDS	wCle_NZ_AP013028	775257	774793	-	465	Mobile element protein	- none -
fig|246273.9.peg.869	CDS	wCle_NZ_AP013028	775583	775269	-	315	Mobile element protein	- none -
fig|246273.9.peg.872	CDS	wCle_NZ_AP013028	779276	778920	-	357	Mobile element protein	- none -
fig|246273.9.peg.873	CDS	wCle_NZ_AP013028	779709	779395	-	315	Mobile element protein	- none -
fig|246273.9.peg.890	CDS	wCle_NZ_AP013028	794064	794378	+	315	Mobile element protein	- none -
fig|246273.9.peg.891	CDS	wCle_NZ_AP013028	794390	794752	+	363	Mobile element protein	- none -
fig|246273.9.peg.892	CDS	wCle_NZ_AP013028	794710	794853	+	144	Mobile element protein	- none -

fig|246273.9.peg.895	CDS	wCle_NZ_AP013028	797258	796794	-	465	Mobile element protein	- none -
fig|246273.9.peg.896	CDS	wCle_NZ_AP013028	797584	797270	-	315	Mobile element protein	- none -
fig|246273.9.peg.900	CDS	wCle_NZ_AP013028	801633	801169	-	465	Mobile element protein	- none -
fig|246273.9.peg.901	CDS	wCle_NZ_AP013028	801959	801645	-	315	Mobile element protein	- none -
fig|246273.9.peg.906	CDS	wCle_NZ_AP013028	804943	805257	+	315	Mobile element protein	- none -
fig|246273.9.peg.907	CDS	wCle_NZ_AP013028	805269	805733	+	465	Mobile element protein	- none -
fig|246273.9.peg.913	CDS	wCle_NZ_AP013028	810737	810273	-	465	Mobile element protein	- none -
fig|246273.9.peg.914	CDS	wCle_NZ_AP013028	811063	810749	-	315	Mobile element protein	- none -
fig|246273.9.peg.925	CDS	wCle_NZ_AP013028	818691	819005	+	315	Mobile element protein	- none -
fig|246273.9.peg.926	CDS	wCle_NZ_AP013028	819017	819481	+	465	Mobile element protein	- none -
fig|246273.9.peg.929	CDS	wCle_NZ_AP013028	822089	822403	+	315	Mobile element protein	- none -
fig|246273.9.peg.930	CDS	wCle_NZ_AP013028	822522	822878	+	357	Mobile element protein	- none -
fig|246273.9.peg.934	CDS	wCle_NZ_AP013028	825930	825616	-	315	Mobile element protein	- none -
fig|246273.9.peg.935	CDS	wCle_NZ_AP013028	826363	826049	-	315	Mobile element protein	- none -
fig|246273.9.peg.939	CDS	wCle_NZ_AP013028	831099	831368	+	270	Mobile element protein	- none -
fig|246273.9.peg.946	CDS	wCle_NZ_AP013028	835948	835592	-	357	Mobile element protein	- none -
fig|246273.9.peg.947	CDS	wCle_NZ_AP013028	836381	836067	-	315	Mobile element protein	- none -
fig|246273.9.peg.961	CDS	wCle_NZ_AP013028	848608	848252	-	357	Mobile element protein	- none -
fig|246273.9.peg.962	CDS	wCle_NZ_AP013028	849041	848727	-	315	Mobile element protein	- none -
fig|246273.9.peg.966	CDS	wCle_NZ_AP013028	853742	853386	-	357	Mobile element protein	- none -
fig|246273.9.peg.967	CDS	wCle_NZ_AP013028	854175	853861	-	315	Mobile element protein	- none -
fig|246273.9.peg.975	CDS	wCle_NZ_AP013028	857947	858261	+	315	Mobile element protein	- none -
fig|246273.9.peg.976	CDS	wCle_NZ_AP013028	858380	858736	+	357	Mobile element protein	- none -

fig|246273.9.peg.981	CDS	wCle_NZ_AP013028	862932	863246	+	315	Mobile element protein	- none -
fig|246273.9.peg.982	CDS	wCle_NZ_AP013028	863258	863620	+	363	Mobile element protein	- none -
fig|246273.9.peg.983	CDS	wCle_NZ_AP013028	863578	863721	+	144	Mobile element protein	- none -
fig|246273.9.peg.1012	CDS	wCle_NZ_AP013028	893235	893549	+	315	Mobile element protein	- none -
fig|246273.9.peg.1013	CDS	wCle_NZ_AP013028	893561	893923	+	363	Mobile element protein	- none -
fig|246273.9.peg.1014	CDS	wCle_NZ_AP013028	893881	894024	+	144	Mobile element protein	- none -
fig|246273.9.peg.1019	CDS	wCle_NZ_AP013028	895780	896094	+	315	Mobile element protein	- none -
fig|246273.9.peg.1020	CDS	wCle_NZ_AP013028	896215	896571	+	357	Mobile element protein	- none -
fig|246273.9.peg.1043	CDS	wCle_NZ_AP013028	920622	920266	-	357	Mobile element protein	- none -
fig|246273.9.peg.1044	CDS	wCle_NZ_AP013028	921055	920741	-	315	Mobile element protein	- none -
fig|246273.9.peg.1051	CDS	wCle_NZ_AP013028	925594	925908	+	315	Mobile element protein	- none -
fig|246273.9.peg.1052	CDS	wCle_NZ_AP013028	926027	926341	+	315	Mobile element protein	- none -
fig|246273.9.peg.1053	CDS	wCle_NZ_AP013028	926651	926379	-	273	Mobile element protein	- none -
fig|246273.9.peg.1075	CDS	wCle_NZ_AP013028	943312	943626	+	315	Mobile element protein	- none -
fig|246273.9.peg.1076	CDS	wCle_NZ_AP013028	943745	944101	+	357	Mobile element protein	- none -
fig|246273.9.peg.1084	CDS	wCle_NZ_AP013028	950402	950716	+	315	Mobile element protein	- none -
fig|246273.9.peg.1085	CDS	wCle_NZ_AP013028	950728	951192	+	465	Mobile element protein	- none -
fig|246273.9.peg.1090	CDS	wCle_NZ_AP013028	954264	953800	-	465	Mobile element protein	- none -
fig|246273.9.peg.1091	CDS	wCle_NZ_AP013028	954590	954276	-	315	Mobile element protein	- none -
fig|246273.9.peg.1110	CDS	wCle_NZ_AP013028	970103	969639	-	465	Mobile element protein	- none -
fig|246273.9.peg.1111	CDS	wCle_NZ_AP013028	970429	970115	-	315	Mobile element protein	- none -
fig|246273.9.peg.1112	CDS	wCle_NZ_AP013028	971044	970580	-	465	Mobile element protein	- none -
fig|246273.9.peg.1113	CDS	wCle_NZ_AP013028	971370	971056	-	315	Mobile element protein	- none -

fig|246273.9.peg.1119	CDS	wCle_NZ_AP013028	974882	975196	+	315	Mobile element protein	- none -
fig|246273.9.peg.1120	CDS	wCle_NZ_AP013028	975208	975570	+	363	Mobile element protein	- none -
fig|246273.9.peg.1164	CDS	wCle_NZ_AP013028	1027754	1028068	+	315	Mobile element protein	- none -
fig|246273.9.peg.1165	CDS	wCle_NZ_AP013028	1028080	1028544	+	465	Mobile element protein	- none -
fig|246273.9.peg.1174	CDS	wCle_NZ_AP013028	1036077	1036391	+	315	Mobile element protein	- none -
fig|246273.9.peg.1175	CDS	wCle_NZ_AP013028	1036510	1036890	+	381	Mobile element protein	- none -
fig|246273.9.peg.1182	CDS	wCle_NZ_AP013028	1042475	1042119	-	357	Mobile element protein	- none -
fig|246273.9.peg.1183	CDS	wCle_NZ_AP013028	1042908	1042594	-	315	Mobile element protein	- none -
fig|246273.9.peg.1185	CDS	wCle_NZ_AP013028	1043508	1043822	+	315	Mobile element protein	- none -
fig|246273.9.peg.1186	CDS	wCle_NZ_AP013028	1043941	1044297	+	357	Mobile element protein	- none -
fig|246273.9.peg.1215	CDS	wCle_NZ_AP013028	1074844	1075158	+	315	Mobile element protein	- none -
fig|246273.9.peg.1216	CDS	wCle_NZ_AP013028	1075277	1075633	+	357	Mobile element protein	- none -
fig|246273.9.peg.1219	CDS	wCle_NZ_AP013028	1078868	1079182	+	315	Mobile element protein	- none -
fig|246273.9.peg.1220	CDS	wCle_NZ_AP013028	1079194	1079658	+	465	Mobile element protein	- none -
fig|246273.9.peg.1237	CDS	wCle_NZ_AP013028	1094020	1093664	-	357	Mobile element protein	- none -
fig|246273.9.peg.1238	CDS	wCle_NZ_AP013028	1094453	1094139	-	315	Mobile element protein	- none -
fig|246273.9.peg.1243	CDS	wCle_NZ_AP013028	1098512	1098156	-	357	Mobile element protein	- none -
fig|246273.9.peg.1244	CDS	wCle_NZ_AP013028	1098945	1098631	-	315	Mobile element protein	- none -
fig|246273.9.peg.1248	CDS	wCle_NZ_AP013028	1101807	1102121	+	315	Mobile element protein	- none -
fig|246273.9.peg.1249	CDS	wCle_NZ_AP013028	1102133	1102597	+	465	Mobile element protein	- none -
fig|246273.9.peg.1287	CDS	wCle_NZ_AP013028	1138396	1138668	+	273	Mobile element protein	- none -
fig|246273.9.peg.1288	CDS	wCle_NZ_AP013028	1139129	1138665	-	465	Mobile element protein	- none -
fig|246273.9.peg.1289	CDS	wCle_NZ_AP013028	1139455	1139141	-	315	Mobile element protein	- none -

	fig|246273.9.peg.1290	CDS	wCle_NZ_AP013028	1139693	1140049	+		357	Mobile element protein	- none -
	fig|246273.9.peg.1299	CDS	wCle_NZ_AP013028	1148985	1148521	-		465	Mobile element protein	- none -
	fig|246273.9.peg.1300	CDS	wCle_NZ_AP013028	1149311	1148997	-		315	Mobile element protein	- none -
	fig|246273.9.peg.1308	CDS	wCle_NZ_AP013028	1155935	1155579	-		357	Mobile element protein	- none -
	fig|246273.9.peg.1309	CDS	wCle_NZ_AP013028	1156368	1156054	-		315	Mobile element protein	- none -
	fig|246273.9.peg.1326	CDS	wCle_NZ_AP013028	1169551	1169159	-		393	Mobile element protein	- none -
	fig|246273.9.peg.1327	CDS	wCle_NZ_AP013028	1169984	1169670	-		315	Mobile element protein	- none -
	fig|246273.9.peg.1343	CDS	wCle_NZ_AP013028	1187338	1187652	+		315	Mobile element protein	- none -
	fig|246273.9.peg.1344	CDS	wCle_NZ_AP013028	1187664	1188128	+		465	Mobile element protein	- none -
	fig|246273.9.peg.1364	CDS	wCle_NZ_AP013028	1207471	1207007	-		465	Mobile element protein	- none -
	fig|246273.9.peg.1365	CDS	wCle_NZ_AP013028	1207797	1207483	-		315	Mobile element protein	- none -
	fig|246273.9.peg.1367	CDS	wCle_NZ_AP013028	1208957	1209271	+		315	Mobile element protein	- none -
	fig|246273.9.peg.1368	CDS	wCle_NZ_AP013028	1209390	1209746	+		357	Mobile element protein	- none -
	fig|246273.9.peg.1381	CDS	wCle_NZ_AP013028	1223391	1223705	+		315	Mobile element protein	- none -
	fig|246273.9.peg.1382	CDS	wCle_NZ_AP013028	1223824	1224276	+		453	Mobile element protein	- none -
wDimm	-	-	-	-	-	-	-		-	-
wDcau	-	-	-	-	-	-	-		-	-
wFol	fig|169402.10.peg.67	CDS	wFol_NZ_CP015510	85045	85617	+		573	Mobile element protein	- none -
	fig|169402.10.peg.83	CDS	wFol_NZ_CP015510	102814	102065	-		750	Mobile element protein	- none -
	fig|169402.10.peg.123	CDS	wFol_NZ_CP015510	151236	150664	-		573	Mobile element protein	- none -
	fig|169402.10.peg.189	CDS	wFol_NZ_CP015510	199332	198874	-		459	Mobile element protein	- none -
	fig|169402.10.peg.203	CDS	wFol_NZ_CP015510	208998	209969	+		972	Mobile element protein	- none -
	fig|169402.10.peg.205	CDS	wFol_NZ_CP015510	211839	211531	-		309	Mobile element protein	- none -

fig|169402.10.peg.207	CDS	wFol_NZ_CP015510	213865	213344	-	522	Mobile element protein	- none -
fig|169402.10.peg.221	CDS	wFol_NZ_CP015510	231074	230103	-	972	Mobile element protein	- none -
fig|169402.10.peg.225	CDS	wFol_NZ_CP015510	234594	235565	+	972	Mobile element protein	- none -
fig|169402.10.peg.227	CDS	wFol_NZ_CP015510	237341	238291	+	951	Mobile element protein	- none -
fig|169402.10.peg.246	CDS	wFol_NZ_CP015510	260870	261841	+	972	Mobile element protein	- none -
fig|169402.10.peg.247	CDS	wFol_NZ_CP015510	261958	262917	+	960	Mobile element protein	- none -
fig|169402.10.peg.258	CDS	wFol_NZ_CP015510	276312	275353	-	960	Mobile element protein	- none -
fig|169402.10.peg.287	CDS	wFol_NZ_CP015510	306562	307311	+	750	Mobile element protein	- none -
fig|169402.10.peg.318	CDS	wFol_NZ_CP015510	340321	340629	+	309	Mobile element protein	- none -
fig|169402.10.peg.320	CDS	wFol_NZ_CP015510	343163	342192	-	972	Mobile element protein	- none -
fig|169402.10.peg.338	CDS	wFol_NZ_CP015510	363464	363333	-	132	Mobile element protein	- none -
fig|169402.10.peg.339	CDS	wFol_NZ_CP015510	365540	363516	-	2025	Mobile element protein	- none -
fig|169402.10.peg.341	CDS	wFol_NZ_CP015510	366410	366063	-	348	Mobile element protein	- none -
fig|169402.10.peg.345	CDS	wFol_NZ_CP015510	369085	369939	+	855	Mobile element protein	- none -
fig|169402.10.peg.347	CDS	wFol_NZ_CP015510	370478	372502	+	2025	Mobile element protein	- none -
fig|169402.10.peg.348	CDS	wFol_NZ_CP015510	372554	372685	+	132	Mobile element protein	- none -
fig|169402.10.peg.380	CDS	wFol_NZ_CP015510	398067	396043	-	2025	Mobile element protein	- none -
fig|169402.10.peg.382	CDS	wFol_NZ_CP015510	399460	398606	-	855	Mobile element protein	- none -
fig|169402.10.peg.408	CDS	wFol_NZ_CP015510	422342	423196	+	855	Mobile element protein	- none -
fig|169402.10.peg.410	CDS	wFol_NZ_CP015510	423735	425759	+	2025	Mobile element protein	- none -
fig|169402.10.peg.463	CDS	wFol_NZ_CP015510	513040	513333	+	294	Mobile element protein	- none -
fig|169402.10.peg.469	CDS	wFol_NZ_CP015510	517685	518656	+	972	Mobile element protein	- none -
fig|169402.10.peg.470	CDS	wFol_NZ_CP015510	519689	518730	-	960	Mobile element protein	- none -

fig|169402.10.peg.508	CDS	wFol_NZ_CP015510	562897	563868	+	972	Mobile element protein	- none -
fig|169402.10.peg.578	CDS	wFol_NZ_CP015510	638367	638236	-	132	Mobile element protein	- none -
fig|169402.10.peg.579	CDS	wFol_NZ_CP015510	640443	638419	-	2025	Mobile element protein	- none -
fig|169402.10.peg.581	CDS	wFol_NZ_CP015510	641313	640966	-	348	Mobile element protein	- none -
fig|169402.10.peg.587	CDS	wFol_NZ_CP015510	644907	645761	+	855	Mobile element protein	- none -
fig|169402.10.peg.589	CDS	wFol_NZ_CP015510	646300	648324	+	2025	Mobile element protein	- none -
fig|169402.10.peg.590	CDS	wFol_NZ_CP015510	648376	648507	+	132	Mobile element protein	- none -
fig|169402.10.peg.652	CDS	wFol_NZ_CP015510	697686	697555	-	132	Mobile element protein	- none -
fig|169402.10.peg.653	CDS	wFol_NZ_CP015510	699762	697738	-	2025	Mobile element protein	- none -
fig|169402.10.peg.693	CDS	wFol_NZ_CP015510	735037	736257	+	1221	Mobile element protein	- none -
fig|169402.10.peg.701	CDS	wFol_NZ_CP015510	756671	757525	+	855	Mobile element protein	- none -
fig|169402.10.peg.703	CDS	wFol_NZ_CP015510	758064	760088	+	2025	Mobile element protein	- none -
fig|169402.10.peg.704	CDS	wFol_NZ_CP015510	760140	760271	+	132	Mobile element protein	- none -
fig|169402.10.peg.735	CDS	wFol_NZ_CP015510	790591	791163	+	573	Mobile element protein	- none -
fig|169402.10.peg.753	CDS	wFol_NZ_CP015510	805472	805897	+	426	Mobile element protein	- none -
fig|169402.10.peg.754	CDS	wFol_NZ_CP015510	805930	806430	+	501	Mobile element protein	- none -
fig|169402.10.peg.755	CDS	wFol_NZ_CP015510	807266	806475	-	792	Mobile element protein	- none -
fig|169402.10.peg.773	CDS	wFol_NZ_CP015510	825224	825973	+	750	Mobile element protein	- none -
fig|169402.10.peg.789	CDS	wFol_NZ_CP015510	844113	843142	-	972	Mobile element protein	- none -
fig|169402.10.peg.792	CDS	wFol_NZ_CP015510	844561	844857	+	297	Mobile element protein	- none -
fig|169402.10.peg.840	CDS	wFol_NZ_CP015510	894075	893782	-	294	Mobile element protein	- none -
fig|169402.10.peg.842	CDS	wFol_NZ_CP015510	894761	895564	+	804	Mobile element protein	- none -
fig|169402.10.peg.877	CDS	wFol_NZ_CP015510	939075	938104	-	972	Mobile element protein	- none -

fig|169402.10.peg.897	CDS	wFol_NZ_CP015510	965463	964504	-	960	Mobile element protein	- none -
fig|169402.10.peg.924	CDS	wFol_NZ_CP015510	990543	989572	-	972	Mobile element protein	- none -
fig|169402.10.peg.927	CDS	wFol_NZ_CP015510	992683	992390	-	294	Mobile element protein	- none -
fig|169402.10.peg.976	CDS	wFol_NZ_CP015510	1039236	1040423	+	1188	Mobile element protein	- none -
fig|169402.10.peg.1021	CDS	wFol_NZ_CP015510	1081816	1082934	+	1119	Mobile element protein	- none -
fig|169402.10.peg.1023	CDS	wFol_NZ_CP015510	1085665	1083641	-	2025	Mobile element protein	- none -
fig|169402.10.peg.1025	CDS	wFol_NZ_CP015510	1087058	1086204	-	855	Mobile element protein	- none -
fig|169402.10.peg.1028	CDS	wFol_NZ_CP015510	1089357	1089136	-	222	Mobile element protein	- none -
fig|169402.10.peg.1043	CDS	wFol_NZ_CP015510	1116273	1117289	+	1017	Mobile element protein	- none -
fig|169402.10.peg.1072	CDS	wFol_NZ_CP015510	1142833	1142084	-	750	Mobile element protein	- none -
fig|169402.10.peg.1106	CDS	wFol_NZ_CP015510	1171445	1170429	-	1017	Mobile element protein	- none -
fig|169402.10.peg.1138	CDS	wFol_NZ_CP015510	1206584	1207093	+	510	Mobile element protein	- none -
fig|169402.10.peg.1148	CDS	wFol_NZ_CP015510	1224512	1226536	+	2025	Mobile element protein	- none -
fig|169402.10.peg.1168	CDS	wFol_NZ_CP015510	1246264	1245755	-	510	Mobile element protein	- none -
fig|169402.10.peg.1183	CDS	wFol_NZ_CP015510	1258327	1259547	+	1221	Mobile element protein	- none -
fig|169402.10.peg.1186	CDS	wFol_NZ_CP015510	1262251	1263000	+	750	Mobile element protein	- none -
fig|169402.10.peg.1227	CDS	wFol_NZ_CP015510	1322890	1321919	-	972	Mobile element protein	- none -
fig|169402.10.peg.1261	CDS	wFol_NZ_CP015510	1353973	1354944	+	972	Mobile element protein	- none -
fig|169402.10.peg.1289	CDS	wFol_NZ_CP015510	1380980	1380231	-	750	Mobile element protein	- none -
fig|169402.10.peg.1342	CDS	wFol_NZ_CP015510	1428324	1429283	+	960	Mobile element protein	- none -
fig|169402.10.peg.1429	CDS	wFol_NZ_CP015510	1535699	1536007	+	309	Mobile element protein	- none -
fig|169402.10.peg.1431	CDS	wFol_NZ_CP015510	1538541	1537570	-	972	Mobile element protein	- none -
fig|169402.10.peg.1457	CDS	wFol_NZ_CP015510	1569518	1568547	-	972	Mobile element protein	- none -

	fig|169402.10.peg.1512	CDS	wFol_NZ_CP015510	1630319	1631290	+	972	Mobile element protein	- none -
	fig|169402.10.peg.1517	CDS	wFol_NZ_CP015510	1640441	1639470	-	972	Mobile element protein	- none -
	fig|169402.10.peg.1539	CDS	wFol_NZ_CP015510	1668023	1667565	-	459	Mobile element protein	- none -
	fig|169402.10.peg.1594	CDS	wFol_NZ_CP015510	1725463	1724492	-	972	Mobile element protein	- none -
	fig|169402.10.peg.1595	CDS	wFol_NZ_CP015510	1725754	1726326	+	573	Mobile element protein	- none -
	fig|169402.10.peg.1604	CDS	wFol_NZ_CP015510	1735518	1736477	+	960	Mobile element protein	- none -
	fig|169402.10.peg.1634	CDS	wFol_NZ_CP015510	1763802	1764773	+	972	Mobile element protein	- none -
	fig|169402.10.peg.1651	CDS	wFol_NZ_CP015510	1782463	1781492	-	972	Mobile element protein	- none -
	fig|169402.10.peg.1652	CDS	wFol_NZ_CP015510	1782754	1783326	+	573	Mobile element protein	- none -
	fig|169402.10.peg.1669	CDS	wFol_NZ_CP015510	1797182	1798153	+	972	Mobile element protein	- none -
wLbra	fig|1812117.6.peg.13	CDS	N762_781_tig19	7146	6934	-	213	Mobile element protein	- none -
	fig|1812117.6.peg.40	CDS	N762_781_tig19	16351	16229	-	123	Mobile element protein	- none -
	fig|1812117.6.peg.61	CDS	N851_N842_tig26	6679	6876	+	198	Mobile element protein	- none -
	fig|1812117.6.peg.107	CDS	NODE_153_length_129046_cov_595.533057_wb	6539	6718	+	180	Mobile element protein	- none -
	fig|1812117.6.peg.124	CDS	NODE_153_length_129046_cov_595.533057_wb	17139	16960	-	180	Mobile element protein	- none -
	fig|1812117.6.peg.360	CDS	NODE_231_length_86603_cov_648.545756_wb	68282	68533	+	252	Mobile element protein	- none -
	fig|1812117.6.peg.406	CDS	NODE_243_length_83068_cov_648.680295_wb	8405	8283	-	123	Mobile element protein	- none -
	fig|1812117.6.peg.474	CDS	NODE_243_length_83068_cov_648.680295_wb	51712	51852	+	141	Mobile element protein	- none -
	fig|1812117.6.peg.521	CDS	NODE_243_length_83068_cov_648.680295_wb	74785	74895	+	111	Mobile element protein	- none -
	fig|1812117.6.peg.531	CDS	NODE_243_length_83068_cov_648.680295_wb	79556	79762	+	207	Mobile element protein	- none -
	fig|1812117.6.peg.564	CDS	NODE_316_length_62772_cov_522.837373_wb	15888	16136	+	249	Mobile element protein	- none -
	fig|1812117.6.peg.619	CDS	NODE_316_length_62772_cov_522.837373_wb	51369	51491	+	123	Mobile element protein	- none -
	fig|1812117.6.peg.662	CDS	NODE_339_length_57503_cov_566.844413_wb	18017	17781	-	237	Mobile element protein	- none -

	fig|1812117.6.peg.701	CDS	NODE_339_length_57503_cov_566.844413_wb	46665	46432	-	234	Mobile element protein	- none -
	fig|1812117.6.peg.720	CDS	NODE_360_length_53405_cov_576.764539_wb	236	3	-	234	Mobile element protein	- none -
	fig|1812117.6.peg.796	CDS	NODE_363_length_52864_cov_761.858878_wb	2794	2510	-	285	Mobile element protein	- none -
	fig|1812117.6.peg.840	CDS	NODE_363_length_52864_cov_761.858878_wb	30258	30103	-	156	Mobile element protein	- none -
	fig|1812117.6.peg.951	CDS	NODE_368_length_52152_cov_864.133273_wb	51526	51738	+	213	Mobile element protein	- none -
	fig|1812117.6.peg.1082	CDS	NODE_444_length_40856_cov_557.980812_wb	24157	23897	-	261	Mobile element protein	- none -
	fig|1812117.6.peg.1324	CDS	NODE_602_length_23040_cov_541.960155_wb	211	2	-	210	Mobile element protein	- none -
	fig|1812117.6.peg.1342	CDS	NODE_602_length_23040_cov_541.960155_wb	8601	8416	-	186	Mobile element protein	- none -
	fig|1812117.6.peg.1430	CDS	NODE_686_length_15990_cov_877.273460_wb	13983	13867	-	117	Mobile element protein	- none -
	fig|1812117.6.peg.1442	CDS	NODE_735_length_12473_cov_275.707808_wb	3278	3099	-	180	Mobile element protein	- none -
	fig|1812117.6.peg.1569	CDS	NODE_814_length_8512_cov_493.240518_wb	8355	8510	+	156	Mobile element protein	- none -
	fig|1812117.6.peg.1614	CDS	NODE_939_length_3662_cov_553.698645_wb	3496	3660	+	165	Mobile element protein	- none -
wLsig	fig|80850.8.peg.147	CDS	canuwLsigCt1p4_wLsig_tig1_corr_circularized_ori	93557	93673	+	117	Mobile element protein	- none -
	fig|80850.8.peg.415	CDS	canuwLsigCt1p4_wLsig_tig1_corr_circularized_ori	257572	257733	+	162	Mobile element protein	- none -
	fig|80850.8.peg.535	CDS	canuwLsigCt1p4_wLsig_tig1_corr_circularized_ori	337457	337570	+	114	Mobile element protein	- none -
	fig|80850.8.peg.587	CDS	canuwLsigCt1p4_wLsig_tig1_corr_circularized_ori	379343	379552	+	210	Mobile element protein	- none -
	fig|80850.8.peg.876	CDS	canuwLsigCt1p4_wLsig_tig1_corr_circularized_ori	615885	616058	+	174	Mobile element protein	- none -
	fig|80850.8.peg.881	CDS	canuwLsigCt1p4_wLsig_tig1_corr_circularized_ori	617049	616882	-	168	Mobile element protein	- none -
	fig|80850.8.peg.1246	CDS	canuwLsigCt1p4_wLsig_tig1_corr_circularized_ori	890552	890719	+	168	Mobile element protein	- none -
	fig|80850.8.peg.1299	CDS	canuwLsigCt1p4_wLsig_tig1_corr_circularized_ori	926592	926401	-	192	Mobile element protein	- none -
	fig|80850.8.peg.1303	CDS	canuwLsigCt1p4_wLsig_tig1_corr_circularized_ori	927234	927425	+	192	Mobile element protein	- none -
	fig|80850.8.peg.1410	CDS	canuwLsigCt1p4_wLsig_tig1_corr_circularized_ori	997075	997251	+	177	Mobile element protein	- none -
wLug	fig|1335053.6.peg.34	CDS	MUIY01000001.1	35358	35507	+	150	Mobile element protein	- none -

fig|1335053.6.peg.36	CDS	MUIY01000001.1	35987	36202	+	216	Mobile element protein	- none -
fig|1335053.6.peg.37	CDS	MUIY01000001.1	36171	36362	+	192	Mobile element protein	- none -
fig|1335053.6.peg.60	CDS	MUIY01000001.1	57573	56227	-	1347	Mobile element protein	- none -
fig|1335053.6.peg.78	CDS	MUIY01000001.1	72180	71839	-	342	Mobile element protein	- none -
fig|1335053.6.peg.79	CDS	MUIY01000001.1	72671	72291	-	381	Mobile element protein	- none -
fig|1335053.6.peg.180	CDS	MUIY01000001.1	179694	180512	+	819	Mobile element protein	- none -
fig|1335053.6.peg.181	CDS	MUIY01000001.1	180630	180974	+	345	Mobile element protein	- none -
fig|1335053.6.peg.184	CDS	MUIY01000001.1	182588	183406	+	819	Mobile element protein	- none -
fig|1335053.6.peg.185	CDS	MUIY01000001.1	183524	183868	+	345	Mobile element protein	- none -
fig|1335053.6.peg.188	CDS	MUIY01000001.1	184722	186068	+	1347	Mobile element protein	- none -
fig|1335053.6.peg.204	CDS	MUIY01000001.1	202204	202049	-	156	Mobile element protein	- none -
fig|1335053.6.peg.207	CDS	MUIY01000001.1	203006	202893	-	114	Mobile element protein	- none -
fig|1335053.6.peg.214	CDS	MUIY01000001.1	210047	209754	-	294	Mobile element protein	- none -
fig|1335053.6.peg.215	CDS	MUIY01000001.1	210181	210062	-	120	Mobile element protein	- none -
fig|1335053.6.peg.216	CDS	MUIY01000001.1	210571	210308	-	264	Mobile element protein	- none -
fig|1335053.6.peg.379	CDS	MUIY01000001.1	364741	365178	+	438	Mobile element protein	- none -
fig|1335053.6.peg.440	CDS	MUIY01000001.1	422985	423170	+	186	Mobile element protein	- none -
fig|1335053.6.peg.441	CDS	MUIY01000001.1	423541	423852	+	312	Mobile element protein	- none -
fig|1335053.6.peg.449	CDS	MUIY01000001.1	428972	429235	+	264	Mobile element protein	- none -
fig|1335053.6.peg.462	CDS	MUIY01000001.1	439800	440018	+	219	Mobile element protein	- none -
fig|1335053.6.peg.463	CDS	MUIY01000001.1	440165	440350	+	186	Mobile element protein	- none -
fig|1335053.6.peg.501	CDS	MUIY01000001.1	476720	476595	-	126	Mobile element protein	- none -
fig|1335053.6.peg.502	CDS	MUIY01000001.1	477223	476999	-	225	Mobile element protein	- none -

fig|1335053.6.peg.503	CDS	MUIY01000001.1	477437	477267	-	171	Mobile element protein	- none -
fig|1335053.6.peg.524	CDS	MUIY01000001.1	495640	495467	-	174	Mobile element protein	- none -
fig|1335053.6.peg.525	CDS	MUIY01000001.1	496032	496145	+	114	Mobile element protein	- none -
fig|1335053.6.peg.580	CDS	MUIY01000001.1	544729	544181	-	549	Mobile element protein	- none -
fig|1335053.6.peg.609	CDS	MUIY01000001.1	569720	569388	-	333	Mobile element protein	- none -
fig|1335053.6.peg.611	CDS	MUIY01000001.1	570811	570527	-	285	Mobile element protein	- none -
fig|1335053.6.peg.642	CDS	MUIY01000001.1	597920	597624	-	297	Mobile element protein	- none -
fig|1335053.6.peg.643	CDS	MUIY01000001.1	598416	598084	-	333	Mobile element protein	- none -
fig|1335053.6.peg.645	CDS	MUIY01000001.1	599082	599378	+	297	Mobile element protein	- none -
fig|1335053.6.peg.657	CDS	MUIY01000001.1	610183	609458	-	726	Mobile element protein	- none -
fig|1335053.6.peg.658	CDS	MUIY01000001.1	610780	610574	-	207	Mobile element protein	- none -
fig|1335053.6.peg.659	CDS	MUIY01000001.1	611016	610825	-	192	Mobile element protein	- none -
fig|1335053.6.peg.660	CDS	MUIY01000001.1	611200	610985	-	216	Mobile element protein	- none -
fig|1335053.6.peg.662	CDS	MUIY01000001.1	611829	611680	-	150	Mobile element protein	- none -
fig|1335053.6.peg.665	CDS	MUIY01000001.1	612568	612759	+	192	Mobile element protein	- none -
fig|1335053.6.peg.666	CDS	MUIY01000001.1	613140	612796	-	345	Mobile element protein	- none -
fig|1335053.6.peg.667	CDS	MUIY01000001.1	614076	613258	-	819	Mobile element protein	- none -
fig|1335053.6.peg.668	CDS	MUIY01000001.1	614637	614467	-	171	Mobile element protein	- none -
fig|1335053.6.peg.669	CDS	MUIY01000001.1	614909	614718	-	192	Mobile element protein	- none -
fig|1335053.6.peg.670	CDS	MUIY01000001.1	615093	614878	-	216	Mobile element protein	- none -
fig|1335053.6.peg.675	CDS	MUIY01000001.1	624453	624157	-	297	Mobile element protein	- none -
fig|1335053.6.peg.681	CDS	MUIY01000001.1	629063	629185	+	123	Mobile element protein	- none -
fig|1335053.6.peg.682	CDS	MUIY01000001.1	629205	629378	+	174	Mobile element protein	- none -

fig|1335053.6.peg.683	CDS	MUIY01000001.1	629344	629529	+	186	Mobile element protein	- none -
fig|1335053.6.peg.685	CDS	MUIY01000001.1	629905	630528	+	624	Mobile element protein	- none -
fig|1335053.6.peg.690	CDS	MUIY01000001.1	636330	636866	+	537	Mobile element protein	- none -
fig|1335053.6.peg.695	CDS	MUIY01000001.1	640321	639785	-	537	Mobile element protein	- none -
fig|1335053.6.peg.724	CDS	MUIY01000001.1	666965	668257	+	1293	Mobile element protein	- none -
fig|1335053.6.peg.726	CDS	MUIY01000001.1	670348	670611	+	264	Mobile element protein	- none -
fig|1335053.6.peg.727	CDS	MUIY01000001.1	670627	670752	+	126	Mobile element protein	- none -
fig|1335053.6.peg.728	CDS	MUIY01000001.1	670842	671138	+	297	Mobile element protein	- none -
fig|1335053.6.peg.795	CDS	MUIY01000001.1	734441	734611	+	171	Mobile element protein	- none -
fig|1335053.6.peg.796	CDS	MUIY01000001.1	734655	734768	+	114	Mobile element protein	- none -
fig|1335053.6.peg.797	CDS	MUIY01000001.1	734965	735138	+	174	Mobile element protein	- none -
fig|1335053.6.peg.798	CDS	MUIY01000001.1	735150	735275	+	126	Mobile element protein	- none -
fig|1335053.6.peg.799	CDS	MUIY01000001.1	735952	735608	-	345	Mobile element protein	- none -
fig|1335053.6.peg.800	CDS	MUIY01000001.1	736888	736070	-	819	Mobile element protein	- none -
fig|1335053.6.peg.809	CDS	MUIY01000001.1	744727	744431	-	297	Mobile element protein	- none -
fig|1335053.6.peg.810	CDS	MUIY01000001.1	745221	744817	-	405	Mobile element protein	- none -
fig|1335053.6.peg.821	CDS	MUIY01000001.1	757302	757177	-	126	Mobile element protein	- none -
fig|1335053.6.peg.822	CDS	MUIY01000001.1	757512	757384	-	129	Mobile element protein	- none -
fig|1335053.6.peg.823	CDS	MUIY01000001.1	757663	757514	-	150	Mobile element protein	- none -
fig|1335053.6.peg.835	CDS	MUIY01000001.1	770803	771183	+	381	Mobile element protein	- none -
fig|1335053.6.peg.836	CDS	MUIY01000001.1	771279	771635	+	357	Mobile element protein	- none -
fig|1335053.6.peg.865	CDS	MUIY01000001.1	799879	800028	+	150	Mobile element protein	- none -
fig|1335053.6.peg.867	CDS	MUIY01000001.1	800508	800723	+	216	Mobile element protein	- none -

fig|1335053.6.peg.868	CDS	MUIY01000001.1	800692	800883	+	192	Mobile element protein	- none -
fig|1335053.6.peg.903	CDS	MUIY01000001.1	835989	835645	-	345	Mobile element protein	- none -
fig|1335053.6.peg.904	CDS	MUIY01000001.1	836925	836107	-	819	Mobile element protein	- none -
fig|1335053.6.peg.913	CDS	MUIY01000001.1	848819	848571	-	249	Mobile element protein	- none -
fig|1335053.6.peg.917	CDS	MUIY01000001.1	850193	850342	+	150	Mobile element protein	- none -
fig|1335053.6.peg.919	CDS	MUIY01000001.1	850822	851037	+	216	Mobile element protein	- none -
fig|1335053.6.peg.920	CDS	MUIY01000001.1	851006	851197	+	192	Mobile element protein	- none -
fig|1335053.6.peg.931	CDS	MUIY01000001.1	864132	863947	-	186	Mobile element protein	- none -
fig|1335053.6.peg.932	CDS	MUIY01000001.1	864497	864279	-	219	Mobile element protein	- none -
fig|1335053.6.peg.935	CDS	MUIY01000001.1	866408	866064	-	345	Mobile element protein	- none -
fig|1335053.6.peg.936	CDS	MUIY01000001.1	867344	866526	-	819	Mobile element protein	- none -
fig|1335053.6.peg.942	CDS	MUIY01000001.1	871548	871766	+	219	Mobile element protein	- none -
fig|1335053.6.peg.955	CDS	MUIY01000001.1	897293	898585	+	1293	Mobile element protein	- none -
fig|1335053.6.peg.971	CDS	MUIY01000001.1	915253	915411	+	159	Mobile element protein	- none -
fig|1335053.6.peg.972	CDS	MUIY01000001.1	915414	915572	+	159	Mobile element protein	- none -
fig|1335053.6.peg.978	CDS	MUIY01000001.1	922717	922526	-	192	Mobile element protein	- none -
fig|1335053.6.peg.979	CDS	MUIY01000001.1	922901	922686	-	216	Mobile element protein	- none -
fig|1335053.6.peg.981	CDS	MUIY01000001.1	923530	923381	-	150	Mobile element protein	- none -
fig|1335053.6.peg.986	CDS	MUIY01000001.1	926800	926985	+	186	Mobile element protein	- none -
fig|1335053.6.peg.987	CDS	MUIY01000001.1	927356	927667	+	312	Mobile element protein	- none -
fig|1335053.6.peg.988	CDS	MUIY01000001.1	927960	927745	-	216	Mobile element protein	- none -
fig|1335053.6.peg.989	CDS	MUIY01000001.1	928085	927972	-	114	Mobile element protein	- none -
fig|1335053.6.peg.990	CDS	MUIY01000001.1	928370	928116	-	255	Mobile element protein	- none -

fig|1335053.6.peg.1012	CDS	MUIY01000001.1	954244	952952	-	1293	Mobile element protein	- none -
fig|1335053.6.peg.1015	CDS	MUIY01000001.1	959719	959549	-	171	Mobile element protein	- none -
fig|1335053.6.peg.1016	CDS	MUIY01000001.1	959991	959800	-	192	Mobile element protein	- none -
fig|1335053.6.peg.1017	CDS	MUIY01000001.1	960175	959960	-	216	Mobile element protein	- none -
fig|1335053.6.peg.1019	CDS	MUIY01000001.1	960804	960655	-	150	Mobile element protein	- none -
fig|1335053.6.peg.1043	CDS	MUIY01000001.1	982363	982250	-	114	Mobile element protein	- none -
fig|1335053.6.peg.1179	CDS	MUIY01000001.1	1108978	1108847	-	132	Mobile element protein	- none -
fig|1335053.6.peg.1180	CDS	MUIY01000001.1	1110037	1109369	-	669	Mobile element protein	- none -
fig|1335053.6.peg.1190	CDS	MUIY01000001.1	1117346	1117552	+	207	Mobile element protein	- none -
fig|1335053.6.peg.1191	CDS	MUIY01000001.1	1117659	1118207	+	549	Mobile element protein	- none -
fig|1335053.6.peg.1198	CDS	MUIY01000001.1	1125869	1125696	-	174	Mobile element protein	- none -
fig|1335053.6.peg.1203	CDS	MUIY01000001.1	1130274	1130606	+	333	Mobile element protein	- none -
fig|1335053.6.peg.1204	CDS	MUIY01000001.1	1130769	1131065	+	297	Mobile element protein	- none -
fig|1335053.6.peg.1213	CDS	MUIY01000001.1	1139222	1139554	+	333	Mobile element protein	- none -
fig|1335053.6.peg.1214	CDS	MUIY01000001.1	1139717	1140013	+	297	Mobile element protein	- none -
fig|1335053.6.peg.1216	CDS	MUIY01000001.1	1141833	1141522	-	312	Mobile element protein	- none -
fig|1335053.6.peg.1217	CDS	MUIY01000001.1	1142389	1142204	-	186	Mobile element protein	- none -
fig|1335053.6.peg.1387	CDS	MUIY01000002.1	162663	162352	-	312	Mobile element protein	- none -
fig|1335053.6.peg.1388	CDS	MUIY01000002.1	163219	163034	-	186	Mobile element protein	- none -
fig|1335053.6.peg.1397	CDS	MUIY01000002.1	170549	170734	+	186	Mobile element protein	- none -
fig|1335053.6.peg.1398	CDS	MUIY01000002.1	171105	171416	+	312	Mobile element protein	- none -
fig|1335053.6.peg.1402	CDS	MUIY01000002.1	174522	174211	-	312	Mobile element protein	- none -
fig|1335053.6.peg.1403	CDS	MUIY01000002.1	175078	174893	-	186	Mobile element protein	- none -

	fig|1335053.6.peg.1408	CDS	MUIY01000002.1	177309	176965	-	345	Mobile element protein	- none -
	fig|1335053.6.peg.1409	CDS	MUIY01000002.1	178245	177427	-	819	Mobile element protein	- none -
	fig|1335053.6.peg.1411	CDS	MUIY01000002.1	179940	181232	+	1293	Mobile element protein	- none -
	fig|1335053.6.peg.1420	CDS	MUIY01000002.1	193344	193192	-	153	Mobile element protein	- none -
	fig|1335053.6.peg.1421	CDS	MUIY01000002.1	193940	193515	-	426	Mobile element protein	- none -
	fig|1335053.6.peg.1423	CDS	MUIY01000002.1	194175	194324	+	150	Mobile element protein	- none -
	fig|1335053.6.peg.1425	CDS	MUIY01000002.1	194804	195019	+	216	Mobile element protein	- none -
	fig|1335053.6.peg.1426	CDS	MUIY01000002.1	194988	195179	+	192	Mobile element protein	- none -
	fig|1335053.6.peg.1427	CDS	MUIY01000002.1	196606	195260	-	1347	Mobile element protein	- none -
	fig|1335053.6.peg.1449	CDS	MUIY01000002.1	222511	221585	-	927	Mobile element protein	- none -
	fig|1335053.6.peg.1453	CDS	MUIY01000002.1	225676	225275	-	402	Mobile element protein	- none -
	fig|1335053.6.peg.1508	CDS	MUIY01000002.1	282380	281856	-	525	Mobile element protein	- none -
	fig|1335053.6.peg.1509	CDS	MUIY01000002.1	282867	282736	-	132	Mobile element protein	- none -
	fig|1335053.6.peg.1534	CDS	MUIY01000002.1	307626	307384	-	243	Mobile element protein	- none -
	fig|1335053.6.peg.1539	CDS	MUIY01000002.1	309540	310076	+	537	Mobile element protein	- none -
	fig|1335053.6.peg.1614	CDS	MUIY01000002.1	371439	371029	-	411	Mobile element protein	- none -
	fig|1335053.6.peg.1619	CDS	MUIY01000002.1	381193	380975	-	219	Mobile element protein	- none -
wMel	fig|163164.1.peg.40	CDS	wMel_NC_002978	46860	46174	-	687	Mobile element protein	- none -
	fig|163164.1.peg.41	CDS	wMel_NC_002978	47198	46857	-	342	Mobile element protein	- none -
	fig|163164.1.peg.42	CDS	wMel_NC_002978	47689	47309	-	381	Mobile element protein	- none -
	fig|163164.1.peg.100	CDS	wMel_NC_002978	107885	109213	+	1329	Mobile element protein	- none -
	fig|163164.1.peg.122	CDS	wMel_NC_002978	126290	126670	+	381	Mobile element protein	- none -
	fig|163164.1.peg.123	CDS	wMel_NC_002978	126781	127122	+	342	Mobile element protein	- none -

fig|163164.1.peg.161	CDS	wMel_NC_002978	165751	165245	-	507	Mobile element protein	- none -
fig|163164.1.peg.191	CDS	wMel_NC_002978	197767	197426	-	342	Mobile element protein	- none -
fig|163164.1.peg.192	CDS	wMel_NC_002978	198258	197878	-	381	Mobile element protein	- none -
fig|163164.1.peg.225	CDS	wMel_NC_002978	239076	237748	-	1329	Mobile element protein	- none -
fig|163164.1.peg.226	CDS	wMel_NC_002978	240427	239195	-	1233	Mobile element protein	- none -
fig|163164.1.peg.291	CDS	wMel_NC_002978	309070	308729	-	342	Mobile element protein	- none -
fig|163164.1.peg.292	CDS	wMel_NC_002978	309561	309181	-	381	Mobile element protein	- none -
fig|163164.1.peg.407	CDS	wMel_NC_002978	437813	438193	+	381	Mobile element protein	- none -
fig|163164.1.peg.408	CDS	wMel_NC_002978	438304	438645	+	342	Mobile element protein	- none -
fig|163164.1.peg.460	CDS	wMel_NC_002978	507960	507580	-	381	Mobile element protein	- none -
fig|163164.1.peg.461	CDS	wMel_NC_002978	508294	507953	-	342	Mobile element protein	- none -
fig|163164.1.peg.462	CDS	wMel_NC_002978	508785	508405	-	381	Mobile element protein	- none -
fig|163164.1.peg.485	CDS	wMel_NC_002978	532315	532695	+	381	Mobile element protein	- none -
fig|163164.1.peg.486	CDS	wMel_NC_002978	532806	533147	+	342	Mobile element protein	- none -
fig|163164.1.peg.499	CDS	wMel_NC_002978	547952	547731	-	222	Mobile element protein	- none -
fig|163164.1.peg.500	CDS	wMel_NC_002978	548107	549435	+	1329	Mobile element protein	- none -
fig|163164.1.peg.523	CDS	wMel_NC_002978	568583	568242	-	342	Mobile element protein	- none -
fig|163164.1.peg.524	CDS	wMel_NC_002978	569074	568694	-	381	Mobile element protein	- none -
fig|163164.1.peg.575	CDS	wMel_NC_002978	634537	634058	-	480	Mobile element protein	- none -
fig|163164.1.peg.576	CDS	wMel_NC_002978	634725	635105	+	381	Mobile element protein	- none -
fig|163164.1.peg.577	CDS	wMel_NC_002978	635216	635557	+	342	Mobile element protein	- none -
fig|163164.1.peg.788	CDS	wMel_NC_002978	848079	847783	-	297	Mobile element protein	- none -
fig|163164.1.peg.814	CDS	wMel_NC_002978	872362	872748	+	387	Mobile element protein	- none -

fig|163164.1.peg.815	CDS	wMel_NC_002978	874056	873715	-	342	Mobile element protein	- none -
fig|163164.1.peg.816	CDS	wMel_NC_002978	874547	874167	-	381	Mobile element protein	- none -
fig|163164.1.peg.824	CDS	wMel_NC_002978	886563	886222	-	342	Mobile element protein	- none -
fig|163164.1.peg.825	CDS	wMel_NC_002978	887054	886674	-	381	Mobile element protein	- none -
fig|163164.1.peg.834	CDS	wMel_NC_002978	896358	896606	+	249	Mobile element protein	- none -
fig|163164.1.peg.835	CDS	wMel_NC_002978	896627	897007	+	381	Mobile element protein	- none -
fig|163164.1.peg.836	CDS	wMel_NC_002978	897118	897459	+	342	Mobile element protein	- none -
fig|163164.1.peg.843	CDS	wMel_NC_002978	908139	908546	+	408	Mobile element protein	- none -
fig|163164.1.peg.1102	CDS	wMel_NC_002978	1172316	1172696	+	381	Mobile element protein	- none -
fig|163164.1.peg.1103	CDS	wMel_NC_002978	1172807	1173148	+	342	Mobile element protein	- none -
fig|163164.1.peg.1214	CDS	wMel_NC_002978	226892	227221	+	330	Mobile element protein	- none -
fig|163164.1.peg.1215	CDS	wMel_NC_002978	227205	227405	+	201	Mobile element protein	- none -
fig|163164.1.peg.1216	CDS	wMel_NC_002978	227425	227817	+	393	Mobile element protein	- none -
fig|163164.1.peg.1229	CDS	wMel_NC_002978	310033	309626	-	408	Mobile element protein	- none -
fig|163164.1.peg.1230	CDS	wMel_NC_002978	310253	310053	-	201	Mobile element protein	- none -
fig|163164.1.peg.1231	CDS	wMel_NC_002978	310566	310237	-	330	Mobile element protein	- none -
fig|163164.1.peg.1270	CDS	wMel_NC_002978	723000	722692	-	309	Mobile element protein	- none -
fig|163164.1.peg.1271	CDS	wMel_NC_002978	723618	723325	-	294	Mobile element protein	- none -
fig|163164.1.peg.1284	CDS	wMel_NC_002978	868830	869237	+	408	Mobile element protein	- none -
fig|163164.1.peg.1285	CDS	wMel_NC_002978	873202	872774	-	429	Mobile element protein	- none -
fig|163164.1.peg.1287	CDS	wMel_NC_002978	874805	875197	+	393	Mobile element protein	- none -
fig|163164.1.peg.1288	CDS	wMel_NC_002978	897773	898165	+	393	Mobile element protein	- none -
fig|163164.1.peg.1301	CDS	wMel_NC_002978	1068928	1068521	-	408	Mobile element protein	- none -

	fig|163164.1.peg.1302	CDS	wMel_NC_002978	1069148	1068948	-	201	Mobile element protein	- none -
	fig|163164.1.peg.1303	CDS	wMel_NC_002978	1069461	1069132	-	330	Mobile element protein	- none -
wMhie	NODE_13561_length_1237_cov_105.168412_wb_F	CDS		49	213	+	165	Mobile element protein	- none -
	NODE_1527_length_14466_cov_128.152000	CDS		98	262	+	165	Mobile element protein	- none -
	NODE_1788_length_12468_cov_131.988689	CDS		12467	12207	-	261	Mobile element protein	- none -
	NODE_2037_length_10977_cov_113.065864	CDS		7126	6770	-	357	Mobile element protein	- none -
	NODE_2047_length_10911_cov_144.540665	CDS		1	318	+	318	Mobile element protein	- none -
	NODE_2794_length_7599_cov_92.124401_wb_F	CDS		624	22	-	603	Mobile element protein	- none -
	NODE_3238_length_6354_cov_98.911384_wb_F	CDS		439	17	-	423	Mobile element protein	- none -
	NODE_3292_length_6258_cov_98.149343_wb_F	CDS		6256	6005	-	252	Mobile element protein	- none -
	NODE_3460_length_5886_cov_91.401208_wb_F	CDS		5884	5648	-	237	Mobile element protein	- none -
	NODE_3707_length_5372_cov_129.935618	CDS		254	3	-	252	Mobile element protein	- none -
	NODE_4051_length_4797_cov_82.495325_wb_F	CDS		4691	4795	+	105	Mobile element protein	- none -
	NODE_4620_length_3999_cov_93.416325_wb_F	CDS		1	162	+	162	Mobile element protein	- none -
	NODE_4627_length_3995_cov_118.168545	CDS		3820	3993	+	174	Mobile element protein	- none -
	NODE_4649_length_3964_cov_82.086238_wb_F	CDS		3	110	+	108	Mobile element protein	- none -
	NODE_5236_length_3419_cov_86.496394	CDS		251	60	-	192	Mobile element protein	- none -
	NODE_5396_length_3316_cov_195.765271	CDS		2	304	+	303	Mobile element protein	- none -
	NODE_6358_length_2708_cov_112.667176	CDS		2706	2566	-	141	Mobile element protein	- none -
	NODE_8063_length_2068_cov_73.752656_wb_F	CDS		224	565	+	342	Mobile element protein	- none -
wNfla	fig|1854759.6.peg.2	CDS	NZ_LYUW01000001.1	720	989	+	270	Mobile element protein	- none -
	fig|1854759.6.peg.64	CDS	NZ_LYUW01000001.1	56688	56948	+	261	Mobile element protein	- none -
	fig|1854759.6.peg.65	CDS	NZ_LYUW01000001.1	56938	57060	+	123	Mobile element protein	- none -

fig|1854759.6.peg.113	CDS	NZ_LYUW01000002.1	52251	52469	+	219	Mobile element protein	- none -
fig|1854759.6.peg.155	CDS	NZ_LYUW01000003.1	38862	38674	-	189	Mobile element protein	- none -
fig|1854759.6.peg.158	CDS	NZ_LYUW01000003.1	39669	39466	-	204	Mobile element protein	- none -
fig|1854759.6.peg.205	CDS	NZ_LYUW01000005.1	303	1	-	303	Mobile element protein	- none -
fig|1854759.6.peg.239	CDS	NZ_LYUW01000006.1	65	229	+	165	Mobile element protein	- none -
fig|1854759.6.peg.412	CDS	NZ_LYUW01000012.1	221	3	-	219	Mobile element protein	- none -
fig|1854759.6.peg.414	CDS	NZ_LYUW01000012.1	1214	1354	+	141	Mobile element protein	- none -
fig|1854759.6.peg.415	CDS	NZ_LYUW01000012.1	1869	2138	+	270	Mobile element protein	- none -
fig|1854759.6.peg.581	CDS	NZ_LYUW01000020.1	1	231	+	231	Mobile element protein	- none -
fig|1854759.6.peg.582	CDS	NZ_LYUW01000020.1	1107	646	-	462	Mobile element protein	- none -
fig|1854759.6.peg.583	CDS	NZ_LYUW01000020.1	1324	1142	-	183	Mobile element protein	- none -
fig|1854759.6.peg.584	CDS	NZ_LYUW01000020.1	1665	1321	-	345	Mobile element protein	- none -
fig|1854759.6.peg.603	CDS	NZ_LYUW01000020.1	17926	18144	+	219	Mobile element protein	- none -
fig|1854759.6.peg.627	CDS	NZ_LYUW01000022.1	435	301	-	135	Mobile element protein	- none -
fig|1854759.6.peg.708	CDS	NZ_LYUW01000027.1	159	1	-	159	Mobile element protein	- none -
fig|1854759.6.peg.740	CDS	NZ_LYUW01000028.1	14906	15169	+	264	Mobile element protein	- none -
fig|1854759.6.peg.765	CDS	NZ_LYUW01000030.1	13803	14135	+	333	Mobile element protein	- none -
fig|1854759.6.peg.801	CDS	NZ_LYUW01000033.1	221	3	-	219	Mobile element protein	- none -
fig|1854759.6.peg.812	CDS	NZ_LYUW01000033.1	8812	7787	-	1026	Mobile element protein	- none -
fig|1854759.6.peg.871	CDS	NZ_LYUW01000037.1	221	3	-	219	Mobile element protein	- none -
fig|1854759.6.peg.900	CDS	NZ_LYUW01000038.1	11442	11140	-	303	Mobile element protein	- none -
fig|1854759.6.peg.901	CDS	NZ_LYUW01000038.1	11671	11414	-	258	Mobile element protein	- none -
fig|1854759.6.peg.902	CDS	NZ_LYUW01000038.1	11927	11658	-	270	Mobile element protein	- none -

fig|1854759.6.peg.903	CDS	NZ_LYUW01000038.1	12201	11932	-	270	Mobile element protein	- none -
fig|1854759.6.peg.916	CDS	NZ_LYUW01000040.1	194	3	-	192	Mobile element protein	- none -
fig|1854759.6.peg.960	CDS	NZ_LYUW01000043.1	234	112	-	123	Mobile element protein	- none -
fig|1854759.6.peg.961	CDS	NZ_LYUW01000043.1	484	224	-	261	Mobile element protein	- none -
fig|1854759.6.peg.1006	CDS	NZ_LYUW01000046.1	10873	11127	+	255	Mobile element protein	- none -
fig|1854759.6.peg.1007	CDS	NZ_LYUW01000046.1	11151	11234	+	84	Mobile element protein	- none -
fig|1854759.6.peg.1060	CDS	NZ_LYUW01000051.1	221	3	-	219	Mobile element protein	- none -
fig|1854759.6.peg.1091	CDS	NZ_LYUW01000054.1	1030	779	-	252	Mobile element protein	- none -
fig|1854759.6.peg.1092	CDS	NZ_LYUW01000054.1	1179	1000	-	180	Mobile element protein	- none -
fig|1854759.6.peg.1093	CDS	NZ_LYUW01000054.1	1705	1412	-	294	Mobile element protein	- none -
fig|1854759.6.peg.1114	CDS	NZ_LYUW01000056.1	234	112	-	123	Mobile element protein	- none -
fig|1854759.6.peg.1115	CDS	NZ_LYUW01000056.1	484	224	-	261	Mobile element protein	- none -
fig|1854759.6.peg.1227	CDS	NZ_LYUW01000068.1	6489	6572	+	84	Mobile element protein	- none -
fig|1854759.6.peg.1248	CDS	NZ_LYUW01000071.1	6113	6331	+	219	Mobile element protein	- none -
fig|1854759.6.peg.1266	CDS	NZ_LYUW01000074.1	221	3	-	219	Mobile element protein	- none -
fig|1854759.6.peg.1283	CDS	NZ_LYUW01000076.1	369	1	-	369	Mobile element protein	- none -
fig|1854759.6.peg.1297	CDS	NZ_LYUW01000077.1	5182	5352	+	171	Mobile element protein	- none -
fig|1854759.6.peg.1333	CDS	NZ_LYUW01000085.1	433	95	-	339	Mobile element protein	- none -
fig|1854759.6.peg.1335	CDS	NZ_LYUW01000085.1	1061	1357	+	297	Mobile element protein	- none -
fig|1854759.6.peg.1349	CDS	NZ_LYUW01000087.1	2677	2273	-	405	Mobile element protein	- none -
fig|1854759.6.peg.1378	CDS	NZ_LYUW01000093.1	3157	3375	+	219	Mobile element protein	- none -
fig|1854759.6.peg.1400	CDS	NZ_LYUW01000098.1	2815	2495	-	321	Mobile element protein	- none -
fig|1854759.6.peg.1411	CDS	NZ_LYUW01000101.1	2649	2867	+	219	Mobile element protein	- none -

fig|1854759.6.peg.1428	CDS	NZ_LYUW01000106.1	221	3	-	219	Mobile element protein	- none -
fig|1854759.6.peg.1448	CDS	NZ_LYUW01000115.1	277	954	+	678	Mobile element protein	- none -
fig|1854759.6.peg.1449	CDS	NZ_LYUW01000115.1	1138	1614	+	477	Mobile element protein	- none -
fig|1854759.6.peg.1463	CDS	NZ_LYUW01000120.1	248	1045	+	798	Mobile element protein	- none -
fig|1854759.6.peg.1468	CDS	NZ_LYUW01000122.1	1134	1352	+	219	Mobile element protein	- none -
fig|1854759.6.peg.1476	CDS	NZ_LYUW01000128.1	221	3	-	219	Mobile element protein	- none -
fig|1854759.6.peg.1479	CDS	NZ_LYUW01000129.1	81	461	+	381	Mobile element protein	- none -
fig|1854759.6.peg.1483	CDS	NZ_LYUW01000131.1	234	112	-	123	Mobile element protein	- none -
fig|1854759.6.peg.1484	CDS	NZ_LYUW01000131.1	484	224	-	261	Mobile element protein	- none -
fig|1854759.6.peg.1486	CDS	NZ_LYUW01000132.1	173	466	+	294	Mobile element protein	- none -
fig|1854759.6.peg.1487	CDS	NZ_LYUW01000132.1	758	522	-	237	Mobile element protein	- none -
fig|1854759.6.peg.1488	CDS	NZ_LYUW01000133.1	74	358	+	285	Mobile element protein	- none -
fig|1854759.6.peg.1494	CDS	NZ_LYUW01000137.1	551	24	-	528	Mobile element protein	- none -
fig|1854759.6.peg.1496	CDS	NZ_LYUW01000138.1	400	621	+	222	Mobile element protein	- none -
fig|1854759.6.peg.1498	CDS	NZ_LYUW01000140.1	230	78	-	153	Mobile element protein	- none -
fig|1854759.6.peg.1499	CDS	NZ_LYUW01000141.1	221	3	-	219	Mobile element protein	- none -
fig|1854759.6.peg.1500	CDS	NZ_LYUW01000141.1	511	329	-	183	Mobile element protein	- none -
fig|1854759.6.peg.1505	CDS	NZ_LYUW01000145.1	81	422	+	342	Mobile element protein	- none -
fig|1854759.6.peg.1506	CDS	NZ_LYUW01000146.1	435	208	-	228	Mobile element protein	- none -
fig|1854759.6.peg.1512	CDS	NZ_LYUW01000154.1	3	185	+	183	Mobile element protein	- none -
fig|1854759.6.peg.1515	CDS	NZ_LYUW01000157.1	296	3	-	294	Mobile element protein	- none -
fig|1854759.6.peg.1516	CDS	NZ_LYUW01000159.1	1	249	+	249	Mobile element protein	- none -
fig|1854759.6.peg.1517	CDS	NZ_LYUW01000160.1	32	235	+	204	Mobile element protein	- none -

	fig|1854759.6.peg.1520	CDS	NZ_LYUW01000167.1	202	2	-	201	Mobile element protein	- none -
wOo	-	-	-	-	-	-	-	-	-
wOv	-	-	-	-	-	-	-	-	-
wPpe	fig|6666666.425803.peg.109	CDS	wPpe_NZ_MJMG01000001	98290	98141	-	150	Mobile element protein	- none -
	fig|6666666.425803.peg.431	CDS	NZ_MJMG01000003.1	22176	21970	-	207	Mobile element protein	- none -
	fig|6666666.425803.peg.938	CDS	NZ_MJMG01000010.1	53277	53366	+	90	Mobile element protein	- none -
	fig|6666666.425803.peg.959	CDS	NZ_MJMG01000011.1	13531	13698	+	168	Mobile element protein	- none -
	fig|6666666.425803.peg.961	CDS	NZ_MJMG01000011.1	13850	14083	+	234	Mobile element protein	- none -
wPip	fig|955.1.peg.1	CDS	wPip_culex15b06	967	494	-	474	Mobile element protein	- none -
	fig|955.1.peg.4	CDS	wPip_culex15b06	2316	2738	+	423	Mobile element protein	- none -
	fig|955.1.peg.57	CDS	culex182c05.q1k	733	128	-	606	Mobile element protein	- none -
	fig|955.1.peg.58	CDS	culex182c05.q1k	1290	883	-	408	Mobile element protein	- none -
	fig|955.1.peg.59	CDS	culex182c05.q1k	1478	1290	-	189	Mobile element protein	- none -
	fig|955.1.peg.62	CDS	culex169h11.p1k	269	649	+	381	Mobile element protein	- none -
	fig|955.1.peg.75	CDS	culex57g05.p1k	1944	1072	-	873	Mobile element protein	- none -
	fig|955.1.peg.87	CDS	culex57g05.p1k	12448	12630	+	183	Mobile element protein	- none -
	fig|955.1.peg.113	CDS	culex8d01.q1ka	23914	24786	+	873	Mobile element protein	- none -
	fig|955.1.peg.186	CDS	culex8d01.q1ka	112015	112683	+	669	Mobile element protein	- none -
	fig|955.1.peg.187	CDS	culex8d01.q1ka	112655	113245	+	591	Mobile element protein	- none -
	fig|955.1.peg.230	CDS	culex136g12.p1k	19996	19124	-	873	Mobile element protein	- none -
	fig|955.1.peg.240	CDS	culex136g12.p1k	26263	26745	+	483	Mobile element protein	- none -
	fig|955.1.peg.252	CDS	culex164g03.p1k	80	538	+	459	Mobile element protein	- none -
	fig|955.1.peg.255	CDS	culex164g03.p1k	2734	1862	-	873	Mobile element protein	- none -

fig|955.1.peg.261	CDS	culex164g03.p1k	8856	9074	+	219	Mobile element protein	- none -
fig|955.1.peg.266	CDS	culex56c01.p1k	3284	3607	+	324	Mobile element protein	- none -
fig|955.1.peg.276	CDS	culex56c01.p1k	13242	12370	-	873	Mobile element protein	- none -
fig|955.1.peg.278	CDS	culex56c01.p1k	13726	14202	+	477	Mobile element protein	- none -
fig|955.1.peg.279	CDS	culex56c01.p1k	14186	14350	+	165	Mobile element protein	- none -
fig|955.1.peg.280	CDS	culex56c01.p1k	14310	14666	+	357	Mobile element protein	- none -
fig|955.1.peg.284	CDS	culex56c01.p1k	16776	16420	-	357	Mobile element protein	- none -
fig|955.1.peg.285	CDS	culex56c01.p1k	16900	16736	-	165	Mobile element protein	- none -
fig|955.1.peg.286	CDS	culex56c01.p1k	17360	16884	-	477	Mobile element protein	- none -
fig|955.1.peg.288	CDS	culex56c01.p1k	19617	19874	+	258	Mobile element protein	- none -
fig|955.1.peg.289	CDS	culex56c01.p1k	20056	20196	+	141	Mobile element protein	- none -
fig|955.1.peg.290	CDS	culex56c01.p1k	20263	20502	+	240	Mobile element protein	- none -
fig|955.1.peg.294	CDS	culex56c01.p1k	25446	24574	-	873	Mobile element protein	- none -
fig|955.1.peg.331	CDS	culex61a11.q1k	9530	9123	-	408	Mobile element protein	- none -
fig|955.1.peg.332	CDS	culex61a11.q1k	10499	9627	-	873	Mobile element protein	- none -
fig|955.1.peg.345	CDS	culex61a11.q1k	26704	26348	-	357	Mobile element protein	- none -
fig|955.1.peg.346	CDS	culex61a11.q1k	26828	26664	-	165	Mobile element protein	- none -
fig|955.1.peg.347	CDS	culex61a11.q1k	27288	26812	-	477	Mobile element protein	- none -
fig|955.1.peg.360	CDS	culex12a01.q1k	3952	3080	-	873	Mobile element protein	- none -
fig|955.1.peg.391	CDS	culex6c12.q1k	127	723	+	597	Mobile element protein	- none -
fig|955.1.peg.402	CDS	culex6c12.q1k	11993	11586	-	408	Mobile element protein	- none -
fig|955.1.peg.405	CDS	culex35f11.p1k	96	968	+	873	Mobile element protein	- none -
fig|955.1.peg.409	CDS	culex35f11.p1k	3601	3272	-	330	Mobile element protein	- none -

fig|955.1.peg.412	CDS	culex35f11.p1k	5690	5100	-	591	Mobile element protein	- none -
fig|955.1.peg.413	CDS	culex35f11.p1k	6330	5662	-	669	Mobile element protein	- none -
fig|955.1.peg.426	CDS	culex35f11.p1k	21351	20113	-	1239	Mobile element protein	- none -
fig|955.1.peg.429	CDS	culex35f11.p1k	22690	21818	-	873	Mobile element protein	- none -
fig|955.1.peg.469	CDS	culex171h04.q1k	6	716	+	711	Mobile element protein	- none -
fig|955.1.peg.500	CDS	culex98d12.p1k	19840	20712	+	873	Mobile element protein	- none -
fig|955.1.peg.501	CDS	culex98d12.p1k	21014	20790	-	225	Mobile element protein	- none -
fig|955.1.peg.503	CDS	culex98d12.p1k	21935	21720	-	216	Mobile element protein	- none -
fig|955.1.peg.505	CDS	culex98d12.p1k	22606	22400	-	207	Mobile element protein	- none -
fig|955.1.peg.566	CDS	culex102f01.p1k	49864	48992	-	873	Mobile element protein	- none -
fig|955.1.peg.576	CDS	culex107d08.p1k	11768	10788	-	981	Mobile element protein	- none -
fig|955.1.peg.577	CDS	culex107d08.p1k	12026	11778	-	249	Mobile element protein	- none -
fig|955.1.peg.597	CDS	culex107d08.p1k	29347	30219	+	873	Mobile element protein	- none -
fig|955.1.peg.638	CDS	culex184h04.q1k	37738	37938	+	201	Mobile element protein	- none -
fig|955.1.peg.641	CDS	culex22a03.p1k	217	807	+	591	Mobile element protein	- none -
fig|955.1.peg.642	CDS	culex22a03.p1k	1186	857	-	330	Mobile element protein	- none -
fig|955.1.peg.644	CDS	culex48c09.p1k	458	198	-	261	Mobile element protein	- none -
fig|955.1.peg.645	CDS	culex48c09.p1k	1302	520	-	783	Mobile element protein	- none -
fig|955.1.peg.652	CDS	culex95h05.q1k	13131	12259	-	873	Mobile element protein	- none -
fig|955.1.peg.656	CDS	culex95h05.q1k	16191	15319	-	873	Mobile element protein	- none -
fig|955.1.peg.659	CDS	culex95h05.q1k	18585	18833	+	249	Mobile element protein	- none -
fig|955.1.peg.660	CDS	culex95h05.q1k	18843	19253	+	411	Mobile element protein	- none -
fig|955.1.peg.661	CDS	culex95h05.q1k	19225	19815	+	591	Mobile element protein	- none -

fig|955.1.peg.662	CDS	culex95h05.q1k	20164	19835	-	330	Mobile element protein	- none -
fig|955.1.peg.674	CDS	culex46e07.p1k	3743	4843	+	1101	Mobile element protein	- none -
fig|955.1.peg.675	CDS	culex46e07.p1k	4849	4980	+	132	Mobile element protein	- none -
fig|955.1.peg.685	CDS	culex46e07.p1k	16478	16678	+	201	Mobile element protein	- none -
fig|955.1.peg.686	CDS	culex46e07.p1k	16755	17627	+	873	Mobile element protein	- none -
fig|955.1.peg.723	CDS	culex111d03.p1k	738	37	-	702	Mobile element protein	- none -
fig|955.1.peg.728	CDS	culex111d03.p1k	4889	5761	+	873	Mobile element protein	- none -
fig|955.1.peg.731	CDS	culex111d03.p1k	9470	9048	-	423	Mobile element protein	- none -
fig|955.1.peg.734	CDS	culex111d03.p1k	12195	12971	+	777	Mobile element protein	- none -
fig|955.1.peg.735	CDS	culex111d03.p1k	12995	13243	+	249	Mobile element protein	- none -
fig|955.1.peg.736	CDS	culex111d03.p1k	13253	13663	+	411	Mobile element protein	- none -
fig|955.1.peg.737	CDS	culex111d03.p1k	13635	14045	+	411	Mobile element protein	- none -
fig|955.1.peg.738	CDS	culex111d03.p1k	14094	14225	+	132	Mobile element protein	- none -
fig|955.1.peg.752	CDS	culex111d03.p1k	34110	34532	+	423	Mobile element protein	- none -
fig|955.1.peg.756	CDS	culex111d03.p1k	36581	35709	-	873	Mobile element protein	- none -
fig|955.1.peg.759	CDS	culex111d03.p1k	38450	39322	+	873	Mobile element protein	- none -
fig|955.1.peg.774	CDS	culex111d03.p1k	55089	55565	+	477	Mobile element protein	- none -
fig|955.1.peg.775	CDS	culex111d03.p1k	55549	55713	+	165	Mobile element protein	- none -
fig|955.1.peg.776	CDS	culex111d03.p1k	55673	56029	+	357	Mobile element protein	- none -
fig|955.1.peg.786	CDS	culex111d03.p1k	65158	64802	-	357	Mobile element protein	- none -
fig|955.1.peg.791	CDS	culex60b10.p1k	312	1	-	312	Mobile element protein	- none -
fig|955.1.peg.798	CDS	culex60b10.p1k	2259	2450	+	192	Mobile element protein	- none -
fig|955.1.peg.806	CDS	culex60b10.p1k	11112	10540	-	573	Mobile element protein	- none -

fig|955.1.peg.809	CDS	culex161e12.p1k	2192	3064	+	873	Mobile element protein	- none -
fig|955.1.peg.846	CDS	culex40h12.p1k	321	73	-	249	Mobile element protein	- none -
fig|955.1.peg.851	CDS	culex40h12.p1k	3347	3820	+	474	Mobile element protein	- none -
fig|955.1.peg.852	CDS	culex40h12.p1k	4609	3737	-	873	Mobile element protein	- none -
fig|955.1.peg.857	CDS	culex40h12.p1k	7390	6800	-	591	Mobile element protein	- none -
fig|955.1.peg.858	CDS	culex40h12.p1k	8030	7362	-	669	Mobile element protein	- none -
fig|955.1.peg.862	CDS	culex40h12.p1k	9215	10147	+	933	Mobile element protein	- none -
fig|955.1.peg.884	CDS	culex74f02.p1k	18546	17674	-	873	Mobile element protein	- none -
fig|955.1.peg.916	CDS	culex154c10.q1k	672	1307	+	636	Mobile element protein	- none -
fig|955.1.peg.917	CDS	culex154c10.q1k	1273	1908	+	636	Mobile element protein	- none -
fig|955.1.peg.922	CDS	culex154c10.q1k	4895	4566	-	330	Mobile element protein	- none -
fig|955.1.peg.933	CDS	culex154c10.q1k	11862	12482	+	621	Mobile element protein	- none -
fig|955.1.peg.951	CDS	culex6a11.q1k	19335	19583	+	249	Mobile element protein	- none -
fig|955.1.peg.952	CDS	culex6a11.q1k	19593	19904	+	312	Mobile element protein	- none -
fig|955.1.peg.953	CDS	culex183b05.p1k	647	291	-	357	Mobile element protein	- none -
fig|955.1.peg.954	CDS	culex183b05.p1k	771	607	-	165	Mobile element protein	- none -
fig|955.1.peg.955	CDS	culex183b05.p1k	1231	755	-	477	Mobile element protein	- none -
fig|955.1.peg.977	CDS	culex181f03.p1k	6851	7723	+	873	Mobile element protein	- none -
fig|955.1.peg.979	CDS	culex181f03.p1k	8734	9210	+	477	Mobile element protein	- none -
fig|955.1.peg.980	CDS	culex181f03.p1k	9194	9358	+	165	Mobile element protein	- none -
fig|955.1.peg.981	CDS	culex181f03.p1k	9318	9674	+	357	Mobile element protein	- none -
fig|955.1.peg.998	CDS	culex147c02.q1k	3641	4513	+	873	Mobile element protein	- none -
fig|955.1.peg.999	CDS	culex147c02.q1k	4610	5017	+	408	Mobile element protein	- none -

fig|955.1.peg.1002	CDS	culex180a01.p1k	140	3	-	138	Mobile element protein	- none -
fig|955.1.peg.1003	CDS	culex180a01.p1k	780	112	-	669	Mobile element protein	- none -
fig|955.1.peg.1005	CDS	culex180a01.p1k	1849	2721	+	873	Mobile element protein	- none -
fig|955.1.peg.1032	CDS	culex180a01.p1k	27157	27633	+	477	Mobile element protein	- none -
fig|955.1.peg.1033	CDS	culex180a01.p1k	27617	27745	+	129	Mobile element protein	- none -
fig|955.1.peg.1036	CDS	culex150d03.p1k	4866	4444	-	423	Mobile element protein	- none -
fig|955.1.peg.1043	CDS	culex150d03.p1k	11328	11678	+	351	Mobile element protein	- none -
fig|955.1.peg.1045	CDS	culex150d03.p1k	11967	12557	+	591	Mobile element protein	- none -
fig|955.1.peg.1051	CDS	culex150d03.p1k	20112	20588	+	477	Mobile element protein	- none -
fig|955.1.peg.1052	CDS	culex150d03.p1k	20572	20736	+	165	Mobile element protein	- none -
fig|955.1.peg.1053	CDS	culex150d03.p1k	20696	21052	+	357	Mobile element protein	- none -
fig|955.1.peg.1084	CDS	culex35f07.p1k	28920	29792	+	873	Mobile element protein	- none -
fig|955.1.peg.1111	CDS	culex126f06.p1k	358	1230	+	873	Mobile element protein	- none -
fig|955.1.peg.1112	CDS	culex126f06.p1k	1573	1409	-	165	Mobile element protein	- none -
fig|955.1.peg.1113	CDS	culex126f06.p1k	2033	1557	-	477	Mobile element protein	- none -
fig|955.1.peg.1116	CDS	culex126f06.p1k	4283	4152	-	132	Mobile element protein	- none -
fig|955.1.peg.1117	CDS	culex126f06.p1k	4420	4289	-	132	Mobile element protein	- none -
fig|955.1.peg.1135	CDS	culex79g04.p1k	13719	14195	+	477	Mobile element protein	- none -
fig|955.1.peg.1136	CDS	culex79g04.p1k	14179	14343	+	165	Mobile element protein	- none -
fig|955.1.peg.1137	CDS	culex79g04.p1k	14303	14659	+	357	Mobile element protein	- none -
fig|955.1.peg.1167	CDS	culex79g04.p1k	47332	46976	-	357	Mobile element protein	- none -
fig|955.1.peg.1168	CDS	culex79g04.p1k	47474	47292	-	183	Mobile element protein	- none -
fig|955.1.peg.1169	CDS	culex79g04.p1k	48362	47490	-	873	Mobile element protein	- none -

fig|955.1.peg.1170	CDS	culex79g04.p1k	48902	48432	-	471	Mobile element protein	- none -
fig|955.1.peg.1176	CDS	culex79g04.p1k	56389	56865	+	477	Mobile element protein	- none -
fig|955.1.peg.1177	CDS	culex79g04.p1k	56849	57013	+	165	Mobile element protein	- none -
fig|955.1.peg.1178	CDS	culex79g04.p1k	56973	57329	+	357	Mobile element protein	- none -
fig|955.1.peg.1225	CDS	culex176d05.q1k	399	1637	+	1239	Mobile element protein	- none -
fig|955.1.peg.1240	CDS	culex176d05.q1k	15732	14494	-	1239	Mobile element protein	- none -
fig|955.1.peg.1253	CDS	culex176d05.q1k	28740	27868	-	873	Mobile element protein	- none -
fig|955.1.peg.1261	CDS	culex176d05.q1k	35974	35102	-	873	Mobile element protein	- none -
fig|955.1.peg.1262	CDS	culex176d05.q1k	36149	36505	+	357	Mobile element protein	- none -
fig|955.1.peg.1269	CDS	culex70a11.p1k	314	904	+	591	Mobile element protein	- none -
fig|955.1.peg.1279	CDS	culex70a11.p1k	10273	9791	-	483	Mobile element protein	- none -
fig|955.1.peg.1285	CDS	culex70a11.p1k	15897	15532	-	366	Mobile element protein	- none -
fig|955.1.peg.1324	CDS	culex180d12.p1k	8580	8912	+	333	Mobile element protein	- none -
fig|955.1.peg.1329	CDS	culex180d12.p1k	15056	15928	+	873	Mobile element protein	- none -
fig|955.1.peg.1334	CDS	culex180d12.p1k	19616	20488	+	873	Mobile element protein	- none -
fig|955.1.peg.1358	CDS	culex143c03.p1k	883	5	-	879	Mobile element protein	- none -
fig|955.1.peg.1367	CDS	culex143c03.p1k	11951	12823	+	873	Mobile element protein	- none -
fig|955.1.peg.1368	CDS	culex143c03.p1k	13004	13672	+	669	Mobile element protein	- none -
fig|955.1.peg.1369	CDS	culex143c03.p1k	13644	14234	+	591	Mobile element protein	- none -
fig|955.1.peg.1402	CDS	culex143c03.p1k	42759	43241	+	483	Mobile element protein	- none -
fig|955.1.peg.1412	CDS	culex143c03.p1k	53318	52953	-	366	Mobile element protein	- none -
fig|955.1.peg.1414	CDS	culex125c07.q1k	866	3	-	864	Mobile element protein	- none -
fig|955.1.peg.1451	CDS	culex125c07.q1k	29492	28620	-	873	Mobile element protein	- none -

	fig|955.1.peg.1465	CDS	culex37f09.p1k	89	355	+	267	Mobile element protein	- none -
	fig|955.1.peg.1466	CDS	culex37f09.p1k	339	503	+	165	Mobile element protein	- none -
	fig|955.1.peg.1467	CDS	culex37f09.p1k	463	819	+	357	Mobile element protein	- none -
	fig|955.1.peg.1474	CDS	culex37f09.p1k	5160	4678	-	483	Mobile element protein	- none -
	fig|955.1.peg.1484	CDS	culex37f09.p1k	15988	15581	-	408	Mobile element protein	- none -
	fig|955.1.peg.1485	CDS	culex37f09.p1k	16516	16133	-	384	Mobile element protein	- none -
	fig|955.1.peg.1486	CDS	culex37f09.p1k	17413	16682	-	732	Mobile element protein	- none -
	fig|955.1.peg.1487	CDS	culex160e12.q1k	772	182	-	591	Mobile element protein	- none -
	fig|955.1.peg.1507	CDS	culex160e12.q1k	25453	26325	+	873	Mobile element protein	- none -
	fig|955.1.peg.1508	CDS	culex160e12.q1k	26848	26372	-	477	Mobile element protein	- none -
	fig|955.1.peg.1509	CDS	culex160e12.q1k	27939	27067	-	873	Mobile element protein	- none -
	fig|955.1.peg.1511	CDS	culex160e12.q1k	28406	28735	+	330	Mobile element protein	- none -
	fig|955.1.peg.1514	CDS	culex160e12.q1k	30140	29544	-	597	Mobile element protein	- none -
	fig|955.1.peg.1526	CDS	culex16c12.p1k	14148	15020	+	873	Mobile element protein	- none -
	fig|955.1.peg.1547	CDS	culex16c12.p1k	33201	33064	-	138	Mobile element protein	- none -
	fig|955.1.peg.1549	CDS	culex102c04.p1k	885	94	-	792	Mobile element protein	- none -
	fig|955.1.peg.1560	CDS	culex102c04.p1k	12390	13490	+	1101	Mobile element protein	- none -
	fig|955.1.peg.1561	CDS	culex102c04.p1k	13496	13627	+	132	Mobile element protein	- none -
	fig|955.1.peg.1570	CDS	culex102c04.p1k	21652	22524	+	873	Mobile element protein	- none -
	fig|955.1.peg.1584	CDS	wPip_culex15b06	29	313	+	285	Mobile element protein	- none -
wstri	fig|368602.8.peg.23	CDS	MUIX01000001.1	17370	18716	+	1347	Mobile element protein	- none -
	fig|368602.8.peg.96	CDS	MUIX01000001.1	88853	89002	+	150	Mobile element protein	- none -
	fig|368602.8.peg.103	CDS	MUIX01000001.1	95367	95218	-	150	Mobile element protein	- none -

fig|368602.8.peg.196	CDS	MUIX01000001.1	179971	181317	+	1347	Mobile element protein	- none -
fig|368602.8.peg.227	CDS	MUIX01000001.1	216498	216647	+	150	Mobile element protein	- none -
fig|368602.8.peg.235	CDS	MUIX01000001.1	223177	223326	+	150	Mobile element protein	- none -
fig|368602.8.peg.252	CDS	MUIX01000001.1	242563	241616	-	948	Mobile element protein	- none -
fig|368602.8.peg.262	CDS	MUIX01000001.1	247657	247508	-	150	Mobile element protein	- none -
fig|368602.8.peg.275	CDS	MUIX01000001.1	255418	255567	+	150	Mobile element protein	- none -
fig|368602.8.peg.287	CDS	MUIX01000001.1	264423	265121	+	699	Mobile element protein	- none -
fig|368602.8.peg.295	CDS	MUIX01000001.1	280878	279931	-	948	Mobile element protein	- none -
fig|368602.8.peg.317	CDS	MUIX01000001.1	296748	295402	-	1347	Mobile element protein	- none -
fig|368602.8.peg.319	CDS	MUIX01000001.1	298277	298498	+	222	Mobile element protein	- none -
fig|368602.8.peg.321	CDS	MUIX01000001.1	299137	299253	+	117	Mobile element protein	- none -
fig|368602.8.peg.344	CDS	MUIX01000001.1	319167	320039	+	873	Mobile element protein	- none -
fig|368602.8.peg.366	CDS	MUIX01000001.1	344772	344921	+	150	Mobile element protein	- none -
fig|368602.8.peg.380	CDS	MUIX01000001.1	362060	361650	-	411	Mobile element protein	- none -
fig|368602.8.peg.381	CDS	MUIX01000001.1	362483	362103	-	381	Mobile element protein	- none -
fig|368602.8.peg.391	CDS	MUIX01000001.1	370613	371110	+	498	Mobile element protein	- none -
fig|368602.8.peg.414	CDS	MUIX01000001.1	392882	393829	+	948	Mobile element protein	- none -
fig|368602.8.peg.440	CDS	MUIX01000001.1	417169	417318	+	150	Mobile element protein	- none -
fig|368602.8.peg.443	CDS	MUIX01000001.1	419148	418999	-	150	Mobile element protein	- none -
fig|368602.8.peg.457	CDS	MUIX01000001.1	433674	433264	-	411	Mobile element protein	- none -
fig|368602.8.peg.458	CDS	MUIX01000001.1	434097	433717	-	381	Mobile element protein	- none -
fig|368602.8.peg.554	CDS	MUIX01000001.1	533338	533033	-	306	Mobile element protein	- none -
fig|368602.8.peg.576	CDS	MUIX01000001.1	560179	560655	+	477	Mobile element protein	- none -

fig|368602.8.peg.577	CDS	MUIX01000001.1	560621	560740	+	120	Mobile element protein	- none -
fig|368602.8.peg.578	CDS	MUIX01000001.1	560755	561102	+	348	Mobile element protein	- none -
fig|368602.8.peg.628	CDS	MUIX01000001.1	626680	626829	+	150	Mobile element protein	- none -
fig|368602.8.peg.669	CDS	MUIX01000001.1	667114	666965	-	150	Mobile element protein	- none -
fig|368602.8.peg.689	CDS	MUIX01000001.1	683754	683617	-	138	Mobile element protein	- none -
fig|368602.8.peg.703	CDS	MUIX01000001.1	696903	697778	+	876	Mobile element protein	- none -
fig|368602.8.peg.706	CDS	MUIX01000001.1	707063	706191	-	873	Mobile element protein	- none -
fig|368602.8.peg.715	CDS	MUIX01000001.1	719300	718803	-	498	Mobile element protein	- none -
fig|368602.8.peg.786	CDS	MUIX01000001.1	798428	797919	-	510	Mobile element protein	- none -
fig|368602.8.peg.787	CDS	MUIX01000001.1	798913	798800	-	114	Mobile element protein	- none -
fig|368602.8.peg.811	CDS	MUIX01000001.1	823415	822069	-	1347	Mobile element protein	- none -
fig|368602.8.peg.840	CDS	MUIX01000001.1	851013	850840	-	174	Mobile element protein	- none -
fig|368602.8.peg.842	CDS	MUIX01000001.1	851928	852041	+	114	Mobile element protein	- none -
fig|368602.8.peg.845	CDS	MUIX01000001.1	852731	852886	+	156	Mobile element protein	- none -
fig|368602.8.peg.922	CDS	MUIX01000001.1	921951	921784	-	168	Mobile element protein	- none -
fig|368602.8.peg.923	CDS	MUIX01000001.1	922204	922079	-	126	Mobile element protein	- none -
fig|368602.8.peg.981	CDS	MUIX01000001.1	971913	972062	+	150	Mobile element protein	- none -
fig|368602.8.peg.992	CDS	MUIX01000001.1	982194	982343	+	150	Mobile element protein	- none -
fig|368602.8.peg.1005	CDS	MUIX01000001.1	992272	991400	-	873	Mobile element protein	- none -
fig|368602.8.peg.1008	CDS	MUIX01000001.1	995596	994250	-	1347	Mobile element protein	- none -
fig|368602.8.peg.1010	CDS	MUIX01000001.1	995884	996339	+	456	Mobile element protein	- none -
fig|368602.8.peg.1011	CDS	MUIX01000001.1	996348	996761	+	414	Mobile element protein	- none -
fig|368602.8.peg.1012	CDS	MUIX01000001.1	996866	996997	+	132	Mobile element protein	- none -

fig|368602.8.peg.1054	CDS	MUIX01000001.1	1035134	1034187	-	948	Mobile element protein	- none -
fig|368602.8.peg.1056	CDS	MUIX01000001.1	1037348	1036002	-	1347	Mobile element protein	- none -
fig|368602.8.peg.1060	CDS	MUIX01000001.1	1040617	1040468	-	150	Mobile element protein	- none -
fig|368602.8.peg.1082	CDS	MUIX01000001.1	1061014	1061886	+	873	Mobile element protein	- none -
fig|368602.8.peg.1161	CDS	MUIX01000001.1	1159348	1159473	+	126	Mobile element protein	- none -
fig|368602.8.peg.1212	CDS	MUIX01000001.1	1197114	1196965	-	150	Mobile element protein	- none -
fig|368602.8.peg.1245	CDS	MUIX01000001.1	1223093	1223242	+	150	Mobile element protein	- none -
fig|368602.8.peg.1247	CDS	MUIX01000001.1	1224153	1225499	+	1347	Mobile element protein	- none -
fig|368602.8.peg.1292	CDS	MUIX01000001.1	1273206	1273057	-	150	Mobile element protein	- none -
fig|368602.8.peg.1370	CDS	MUIX01000001.1	1337921	1337769	-	153	Mobile element protein	- none -
fig|368602.8.peg.1388	CDS	MUIX01000001.1	1367815	1368243	+	429	Mobile element protein	- none -
fig|368602.8.peg.1404	CDS	MUIX01000001.1	1378346	1378197	-	150	Mobile element protein	- none -
fig|368602.8.peg.1410	CDS	MUIX01000001.1	1382200	1382349	+	150	Mobile element protein	- none -
fig|368602.8.peg.1432	CDS	MUIX01000001.1	1402164	1402322	+	159	Mobile element protein	- none -
fig|368602.8.peg.1492	CDS	MUIX01000001.1	1461761	1461582	-	180	Mobile element protein	- none -
fig|368602.8.peg.1500	CDS	MUIX01000001.1	1465595	1466185	+	591	Mobile element protein	- none -
fig|368602.8.peg.1509	CDS	MUIX01000001.1	1477470	1476523	-	948	Mobile element protein	- none -
fig|368602.8.peg.1511	CDS	MUIX01000001.1	1478191	1478511	+	321	Mobile element protein	- none -
fig|368602.8.peg.1512	CDS	MUIX01000001.1	1478514	1478810	+	297	Mobile element protein	- none -
fig|368602.8.peg.1563	CDS	MUIX01000001.1	1532161	1532012	-	150	Mobile element protein	- none -
fig|368602.8.peg.1578	CDS	MUIX01000001.1	1548911	1548039	-	873	Mobile element protein	- none -
fig|368602.8.peg.1581	CDS	MUIX01000001.1	1551001	1550858	-	144	Mobile element protein	- none -
fig|368602.8.peg.1650	CDS	MUIX01000002.1	13813	13664	-	150	Mobile element protein	- none -

	fig|368602.8.peg.1662	CDS	MUIX01000002.1	24502	25089	+	588	Mobile element protein	- none -
	fig|368602.8.peg.1676	CDS	MUIX01000002.1	41983	41036	-	948	Mobile element protein	- none -
	fig|368602.8.peg.1684	CDS	MUIX01000002.1	46876	45929	-	948	Mobile element protein	- none -
wTpre	fig|125593.4.peg.19	CDS	NZ_CM003641.1	19000	19152	+	153	Mobile element protein	- none -
	fig|125593.4.peg.20	CDS	NZ_CM003641.1	19459	19599	+	141	Mobile element protein	- none -
	fig|125593.4.peg.83	CDS	NZ_CM003641.1	63818	63934	+	117	Mobile element protein	- none -
	fig|125593.4.peg.84	CDS	NZ_CM003641.1	64010	64162	+	153	Mobile element protein	- none -
	fig|125593.4.peg.103	CDS	NZ_CM003641.1	81741	81454	-	288	Mobile element protein	- none -
	fig|125593.4.peg.116	CDS	NZ_CM003641.1	92046	92351	+	306	Mobile element protein	- none -
	fig|125593.4.peg.133	CDS	NZ_CM003641.1	102732	102869	+	138	Mobile element protein	- none -
	fig|125593.4.peg.143	CDS	NZ_CM003641.1	110438	110322	-	117	Mobile element protein	- none -
	fig|125593.4.peg.305	CDS	NZ_CM003641.1	246336	246452	+	117	Mobile element protein	- none -
	fig|125593.4.peg.327	CDS	NZ_CM003641.1	258863	259054	+	192	Mobile element protein	- none -
	fig|125593.4.peg.353	CDS	NZ_CM003641.1	280927	281193	+	267	Mobile element protein	- none -
	fig|125593.4.peg.354	CDS	NZ_CM003641.1	281316	281621	+	306	Mobile element protein	- none -
	fig|125593.4.peg.404	CDS	NZ_CM003641.1	315522	315394	-	129	Mobile element protein	- none -
	fig|125593.4.peg.405	CDS	NZ_CM003641.1	315730	315599	-	132	Mobile element protein	- none -
	fig|125593.4.peg.456	CDS	NZ_CM003641.1	360148	359957	-	192	Mobile element protein	- none -
	fig|125593.4.peg.532	CDS	NZ_CM003641.1	425795	426100	+	306	Mobile element protein	- none -
	fig|125593.4.peg.543	CDS	NZ_CM003641.1	439570	440172	+	603	Mobile element protein	- none -
	fig|125593.4.peg.564	CDS	NZ_CM003641.1	455497	455679	+	183	Mobile element protein	- none -
	fig|125593.4.peg.565	CDS	NZ_CM003641.1	455683	455811	+	129	Mobile element protein	- none -
	fig|125593.4.peg.566	CDS	NZ_CM003641.1	455838	456071	+	234	Mobile element protein	- none -

fig|125593.4.peg.567	CDS	NZ_CM003641.1	456225	456827	+	603	Mobile element protein	- none -
fig|125593.4.peg.768	CDS	NZ_CM003641.1	625607	625491	-	117	Mobile element protein	- none -
fig|125593.4.peg.847	CDS	NZ_CM003641.1	688739	688137	-	603	Mobile element protein	- none -
fig|125593.4.peg.848	CDS	NZ_CM003641.1	689126	688893	-	234	Mobile element protein	- none -
fig|125593.4.peg.849	CDS	NZ_CM003641.1	689281	689153	-	129	Mobile element protein	- none -
fig|125593.4.peg.850	CDS	NZ_CM003641.1	689467	689285	-	183	Mobile element protein	- none -
fig|125593.4.peg.933	CDS	NZ_CM003641.1	754707	754105	-	603	Mobile element protein	- none -
fig|125593.4.peg.934	CDS	NZ_CM003641.1	755094	754861	-	234	Mobile element protein	- none -
fig|125593.4.peg.935	CDS	NZ_CM003641.1	755249	755121	-	129	Mobile element protein	- none -
fig|125593.4.peg.936	CDS	NZ_CM003641.1	755435	755253	-	183	Mobile element protein	- none -
fig|125593.4.peg.937	CDS	NZ_CM003641.1	755848	755654	-	195	Mobile element protein	- none -
fig|125593.4.peg.938	CDS	NZ_CM003641.1	755963	755838	-	126	Mobile element protein	- none -
fig|125593.4.peg.939	CDS	NZ_CM003641.1	756131	755988	-	144	Mobile element protein	- none -
fig|125593.4.peg.964	CDS	NZ_CM003641.1	772019	772135	+	117	Mobile element protein	- none -
fig|125593.4.peg.987	CDS	NZ_CM003641.1	789463	788861	-	603	Mobile element protein	- none -
fig|125593.4.peg.988	CDS	NZ_CM003641.1	789850	789617	-	234	Mobile element protein	- none -
fig|125593.4.peg.989	CDS	NZ_CM003641.1	790005	789877	-	129	Mobile element protein	- none -
fig|125593.4.peg.990	CDS	NZ_CM003641.1	790191	790009	-	183	Mobile element protein	- none -
fig|125593.4.peg.1095	CDS	NZ_CM003641.1	874360	874542	+	183	Mobile element protein	- none -
fig|125593.4.peg.1096	CDS	NZ_CM003641.1	874546	874674	+	129	Mobile element protein	- none -
fig|125593.4.peg.1097	CDS	NZ_CM003641.1	874701	874934	+	234	Mobile element protein	- none -
fig|125593.4.peg.1098	CDS	NZ_CM003641.1	875088	875690	+	603	Mobile element protein	- none -
fig|125593.4.peg.1104	CDS	NZ_CM003641.1	882093	882209	+	117	Mobile element protein	- none -

	fig|125593.4.peg.1176	CDS	NZ_CM003641.1	938835	938692	-	144	Mobile element protein	- none -
	fig|125593.4.peg.1192	CDS	NZ_CM003641.1	952419	952276	-	144	Mobile element protein	- none -
	fig|125593.4.peg.1193	CDS	NZ_CM003641.1	952656	952498	-	159	Mobile element protein	- none -
	fig|125593.4.peg.1194	CDS	NZ_CM003641.1	952864	952733	-	132	Mobile element protein	- none -
	fig|125593.4.peg.1298	CDS	NZ_CM003641.1	1057695	1057877	+	183	Mobile element protein	- none -
	fig|125593.4.peg.1299	CDS	NZ_CM003641.1	1057881	1058009	+	129	Mobile element protein	- none -
	fig|125593.4.peg.1300	CDS	NZ_CM003641.1	1058036	1058269	+	234	Mobile element protein	- none -
	fig|125593.4.peg.1301	CDS	NZ_CM003641.1	1058423	1059025	+	603	Mobile element protein	- none -
	fig|125593.4.peg.1360	CDS	NZ_CM003641.1	1110505	1110347	-	159	Mobile element protein	- none -
wVulC	fig|1220511.6.peg.1	CDS	ALWU01000001.1	486	229	-	258	Mobile element protein	- none -
	fig|1220511.6.peg.2	CDS	ALWU01000001.1	719	555	-	165	Mobile element protein	- none -
	fig|1220511.6.peg.9	CDS	ALWU01000001.1	6218	6790	+	573	Mobile element protein	- none -
	fig|1220511.6.peg.10	CDS	ALWU01000001.1	7059	7457	+	399	Mobile element protein	- none -
	fig|1220511.6.peg.11	CDS	ALWU01000001.1	7489	7695	+	207	Mobile element protein	- none -
	fig|1220511.6.peg.12	CDS	ALWU01000001.1	7703	8392	+	690	Mobile element protein	- none -
	fig|1220511.6.peg.17	CDS	ALWU01000001.1	11853	13052	+	1200	Mobile element protein	- none -
	fig|1220511.6.peg.20	CDS	ALWU01000001.1	15013	14717	-	297	Mobile element protein	- none -
	fig|1220511.6.peg.103	CDS	ALWU01000001.1	101350	101787	+	438	Mobile element protein	- none -
	fig|1220511.6.peg.104	CDS	ALWU01000001.1	101835	102008	+	174	Mobile element protein	- none -
	fig|1220511.6.peg.121	CDS	ALWU01000001.1	119941	119159	-	783	Mobile element protein	- none -
	fig|1220511.6.peg.122	CDS	ALWU01000001.1	120201	120010	-	192	Mobile element protein	- none -
	fig|1220511.6.peg.125	CDS	ALWU01000001.1	122714	122956	+	243	Mobile element protein	- none -
	fig|1220511.6.peg.126	CDS	ALWU01000001.1	123164	123298	+	135	Mobile element protein	- none -

fig|1220511.6.peg.127	CDS	ALWU01000001.1	123592	123723	+	132	Mobile element protein	- none -
fig|1220511.6.peg.130	CDS	ALWU01000001.1	125723	125331	-	393	Mobile element protein	- none -
fig|1220511.6.peg.131	CDS	ALWU01000001.1	125959	125828	-	132	Mobile element protein	- none -
fig|1220511.6.peg.132	CDS	ALWU01000001.1	126387	126253	-	135	Mobile element protein	- none -
fig|1220511.6.peg.133	CDS	ALWU01000001.1	126711	126595	-	117	Mobile element protein	- none -
fig|1220511.6.peg.143	CDS	ALWU01000001.1	131924	132277	+	354	Mobile element protein	- none -
fig|1220511.6.peg.144	CDS	ALWU01000001.1	132473	132592	+	120	Mobile element protein	- none -
fig|1220511.6.peg.148	CDS	ALWU01000001.1	133518	133796	+	279	Mobile element protein	- none -
fig|1220511.6.peg.155	CDS	ALWU01000001.1	140437	140751	+	315	Mobile element protein	- none -
fig|1220511.6.peg.156	CDS	ALWU01000001.1	140755	140922	+	168	Mobile element protein	- none -
fig|1220511.6.peg.209	CDS	ALWU01000001.1	192037	191348	-	690	Mobile element protein	- none -
fig|1220511.6.peg.211	CDS	ALWU01000001.1	192719	192588	-	132	Mobile element protein	- none -
fig|1220511.6.peg.212	CDS	ALWU01000001.1	193147	193013	-	135	Mobile element protein	- none -
fig|1220511.6.peg.213	CDS	ALWU01000001.1	193597	193355	-	243	Mobile element protein	- none -
fig|1220511.6.peg.217	CDS	ALWU01000001.1	195487	195747	+	261	Mobile element protein	- none -
fig|1220511.6.peg.218	CDS	ALWU01000001.1	196019	196246	+	228	Mobile element protein	- none -
fig|1220511.6.peg.219	CDS	ALWU01000001.1	196338	196508	+	171	Mobile element protein	- none -
fig|1220511.6.peg.233	CDS	ALWU01000001.1	207218	207084	-	135	Mobile element protein	- none -
fig|1220511.6.peg.234	CDS	ALWU01000001.1	207668	207426	-	243	Mobile element protein	- none -
fig|1220511.6.peg.238	CDS	ALWU01000001.1	210025	209894	-	132	Mobile element protein	- none -
fig|1220511.6.peg.239	CDS	ALWU01000001.1	210453	210319	-	135	Mobile element protein	- none -
fig|1220511.6.peg.240	CDS	ALWU01000001.1	210903	210661	-	243	Mobile element protein	- none -
fig|1220511.6.peg.266	CDS	ALWU01000001.1	233597	233767	+	171	Mobile element protein	- none -

fig|1220511.6.peg.267	CDS	ALWU01000001.1	234108	234236	+	129	Mobile element protein	- none -
fig|1220511.6.peg.285	CDS	ALWU01000001.1	254207	254013	-	195	Mobile element protein	- none -
fig|1220511.6.peg.293	CDS	ALWU01000001.1	256508	256822	+	315	Mobile element protein	- none -
fig|1220511.6.peg.294	CDS	ALWU01000001.1	256779	257138	+	360	Mobile element protein	- none -
fig|1220511.6.peg.305	CDS	ALWU01000001.1	266087	266440	+	354	Mobile element protein	- none -
fig|1220511.6.peg.319	CDS	ALWU01000001.1	279147	279266	+	120	Mobile element protein	- none -
fig|1220511.6.peg.320	CDS	ALWU01000001.1	279325	279447	+	123	Mobile element protein	- none -
fig|1220511.6.peg.321	CDS	ALWU01000001.1	280487	279855	-	633	Mobile element protein	- none -
fig|1220511.6.peg.322	CDS	ALWU01000001.1	281443	281036	-	408	Mobile element protein	- none -
fig|1220511.6.peg.323	CDS	ALWU01000001.1	282118	281828	-	291	Mobile element protein	- none -
fig|1220511.6.peg.325	CDS	ALWU01000001.1	283228	283019	-	210	Mobile element protein	- none -
fig|1220511.6.peg.330	CDS	ALWU01000001.1	287291	287196	-	96	Mobile element protein	- none -
fig|1220511.6.peg.350	CDS	ALWU01000001.1	303646	304131	+	486	Mobile element protein	- none -
fig|1220511.6.peg.351	CDS	ALWU01000001.1	304283	304438	+	156	Mobile element protein	- none -
fig|1220511.6.peg.356	CDS	ALWU01000001.1	307442	308194	+	753	Mobile element protein	- none -
fig|1220511.6.peg.358	CDS	ALWU01000001.1	309528	309839	+	312	Mobile element protein	- none -
fig|1220511.6.peg.359	CDS	ALWU01000001.1	310346	309861	-	486	Mobile element protein	- none -
fig|1220511.6.peg.374	CDS	ALWU01000001.1	320649	320807	+	159	Mobile element protein	- none -
fig|1220511.6.peg.375	CDS	ALWU01000001.1	320768	320932	+	165	Mobile element protein	- none -
fig|1220511.6.peg.376	CDS	ALWU01000001.1	321314	321550	+	237	Mobile element protein	- none -
fig|1220511.6.peg.378	CDS	ALWU01000001.1	321911	322090	+	180	Mobile element protein	- none -
fig|1220511.6.peg.381	CDS	ALWU01000001.1	324347	324514	+	168	Mobile element protein	- none -
fig|1220511.6.peg.400	CDS	ALWU01000001.1	347078	346962	-	117	Mobile element protein	- none -

fig|1220511.6.peg.401	CDS	ALWU01000001.1	347456	347331	-	126	Mobile element protein	- none -
fig|1220511.6.peg.402	CDS	ALWU01000001.1	347609	347490	-	120	Mobile element protein	- none -
fig|1220511.6.peg.489	CDS	ALWU01000001.1	418396	418241	-	156	Mobile element protein	- none -
fig|1220511.6.peg.490	CDS	ALWU01000001.1	418520	418362	-	159	Mobile element protein	- none -
fig|1220511.6.peg.491	CDS	ALWU01000001.1	418812	418621	-	192	Mobile element protein	- none -
fig|1220511.6.peg.492	CDS	ALWU01000001.1	419048	418893	-	156	Mobile element protein	- none -
fig|1220511.6.peg.493	CDS	ALWU01000001.1	419421	419302	-	120	Mobile element protein	- none -
fig|1220511.6.peg.513	CDS	ALWU01000001.1	433083	432952	-	132	Mobile element protein	- none -
fig|1220511.6.peg.514	CDS	ALWU01000001.1	433511	433377	-	135	Mobile element protein	- none -
fig|1220511.6.peg.515	CDS	ALWU01000001.1	433982	433719	-	264	Mobile element protein	- none -
fig|1220511.6.peg.523	CDS	ALWU01000001.1	441991	442206	+	216	Mobile element protein	- none -
fig|1220511.6.peg.524	CDS	ALWU01000001.1	442250	442564	+	315	Mobile element protein	- none -
fig|1220511.6.peg.525	CDS	ALWU01000001.1	442569	442751	+	183	Mobile element protein	- none -
fig|1220511.6.peg.528	CDS	ALWU01000001.1	445242	445114	-	129	Mobile element protein	- none -
fig|1220511.6.peg.530	CDS	ALWU01000001.1	445814	445677	-	138	Mobile element protein	- none -
fig|1220511.6.peg.542	CDS	ALWU01000001.1	455260	455123	-	138	Mobile element protein	- none -
fig|1220511.6.peg.543	CDS	ALWU01000001.1	455685	455530	-	156	Mobile element protein	- none -
fig|1220511.6.peg.544	CDS	ALWU01000001.1	455849	455673	-	177	Mobile element protein	- none -
fig|1220511.6.peg.555	CDS	ALWU01000001.1	467015	466848	-	168	Mobile element protein	- none -
fig|1220511.6.peg.556	CDS	ALWU01000001.1	467491	467357	-	135	Mobile element protein	- none -
fig|1220511.6.peg.557	CDS	ALWU01000001.1	468345	467593	-	753	Mobile element protein	- none -
fig|1220511.6.peg.564	CDS	ALWU01000001.1	476088	475399	-	690	Mobile element protein	- none -
fig|1220511.6.peg.565	CDS	ALWU01000001.1	476290	476096	-	195	Mobile element protein	- none -

fig|1220511.6.peg.570	CDS	ALWU01000001.1	482031	481900	-	132	Mobile element protein	- none -
fig|1220511.6.peg.571	CDS	ALWU01000001.1	482459	482325	-	135	Mobile element protein	- none -
fig|1220511.6.peg.572	CDS	ALWU01000001.1	482909	482667	-	243	Mobile element protein	- none -
fig|1220511.6.peg.600	CDS	ALWU01000001.1	513153	512971	-	183	Mobile element protein	- none -
fig|1220511.6.peg.601	CDS	ALWU01000001.1	513472	513158	-	315	Mobile element protein	- none -
fig|1220511.6.peg.602	CDS	ALWU01000001.1	513731	513516	-	216	Mobile element protein	- none -
fig|1220511.6.peg.608	CDS	ALWU01000001.1	518428	518201	-	228	Mobile element protein	- none -
fig|1220511.6.peg.609	CDS	ALWU01000001.1	518960	518700	-	261	Mobile element protein	- none -
fig|1220511.6.peg.610	CDS	ALWU01000001.1	519153	518968	-	186	Mobile element protein	- none -
fig|1220511.6.peg.625	CDS	ALWU01000001.1	536523	536729	+	207	Mobile element protein	- none -
fig|1220511.6.peg.626	CDS	ALWU01000001.1	536737	537426	+	690	Mobile element protein	- none -
fig|1220511.6.peg.628	CDS	ALWU01000001.1	539346	539149	-	198	Mobile element protein	- none -
fig|1220511.6.peg.629	CDS	ALWU01000001.1	539530	539417	-	114	Mobile element protein	- none -
fig|1220511.6.peg.640	CDS	ALWU01000001.1	551851	552099	+	249	Mobile element protein	- none -
fig|1220511.6.peg.641	CDS	ALWU01000001.1	552254	552457	+	204	Mobile element protein	- none -
fig|1220511.6.peg.652	CDS	ALWU01000001.1	562995	562864	-	132	Mobile element protein	- none -
fig|1220511.6.peg.653	CDS	ALWU01000001.1	563423	563289	-	135	Mobile element protein	- none -
fig|1220511.6.peg.654	CDS	ALWU01000001.1	563873	563631	-	243	Mobile element protein	- none -
fig|1220511.6.peg.666	CDS	ALWU01000001.1	572408	572268	-	141	Mobile element protein	- none -
fig|1220511.6.peg.671	CDS	ALWU01000001.1	576644	576159	-	486	Mobile element protein	- none -
fig|1220511.6.peg.675	CDS	ALWU01000001.1	578067	578180	+	114	Mobile element protein	- none -
fig|1220511.6.peg.676	CDS	ALWU01000001.1	578370	578242	-	129	Mobile element protein	- none -
fig|1220511.6.peg.687	CDS	ALWU01000002.1	3779	3384	-	396	Mobile element protein	- none -

fig|1220511.6.peg.733	CDS	ALWU01000003.1	899	117	-	783	Mobile element protein	- none -
fig|1220511.6.peg.734	CDS	ALWU01000003.1	1159	968	-	192	Mobile element protein	- none -
fig|1220511.6.peg.738	CDS	ALWU01000003.1	4170	3943	-	228	Mobile element protein	- none -
fig|1220511.6.peg.739	CDS	ALWU01000003.1	4597	4442	-	156	Mobile element protein	- none -
fig|1220511.6.peg.740	CDS	ALWU01000003.1	4761	4585	-	177	Mobile element protein	- none -
fig|1220511.6.peg.742	CDS	ALWU01000003.1	5252	5025	-	228	Mobile element protein	- none -
fig|1220511.6.peg.743	CDS	ALWU01000003.1	5784	5524	-	261	Mobile element protein	- none -
fig|1220511.6.peg.744	CDS	ALWU01000003.1	6031	5792	-	240	Mobile element protein	- none -
fig|1220511.6.peg.752	CDS	ALWU01000003.1	13094	13342	+	249	Mobile element protein	- none -
fig|1220511.6.peg.753	CDS	ALWU01000003.1	13497	13700	+	204	Mobile element protein	- none -
fig|1220511.6.peg.758	CDS	ALWU01000003.1	18497	17298	-	1200	Mobile element protein	- none -
fig|1220511.6.peg.765	CDS	ALWU01000003.1	25105	25221	+	117	Mobile element protein	- none -
fig|1220511.6.peg.766	CDS	ALWU01000003.1	25429	25563	+	135	Mobile element protein	- none -
fig|1220511.6.peg.767	CDS	ALWU01000003.1	25857	25988	+	132	Mobile element protein	- none -
fig|1220511.6.peg.768	CDS	ALWU01000003.1	26394	26059	-	336	Mobile element protein	- none -
fig|1220511.6.peg.773	CDS	ALWU01000003.1	28454	29158	+	705	Mobile element protein	- none -
fig|1220511.6.peg.817	CDS	ALWU01000005.1	1003	17	-	987	Mobile element protein	- none -
fig|1220511.6.peg.819	CDS	ALWU01000005.1	1437	1589	+	153	Mobile element protein	- none -
fig|1220511.6.peg.820	CDS	ALWU01000005.1	1543	1719	+	177	Mobile element protein	- none -
fig|1220511.6.peg.822	CDS	ALWU01000005.1	2709	2915	+	207	Mobile element protein	- none -
fig|1220511.6.peg.823	CDS	ALWU01000005.1	2923	3612	+	690	Mobile element protein	- none -
fig|1220511.6.peg.853	CDS	ALWU01000005.1	35459	34677	-	783	Mobile element protein	- none -
fig|1220511.6.peg.854	CDS	ALWU01000005.1	35719	35528	-	192	Mobile element protein	- none -

fig|1220511.6.peg.888	CDS	ALWU01000005.1	62018	61236	-	783	Mobile element protein	- none -
fig|1220511.6.peg.889	CDS	ALWU01000005.1	62278	62087	-	192	Mobile element protein	- none -
fig|1220511.6.peg.890	CDS	ALWU01000005.1	63679	62495	-	1185	Mobile element protein	- none -
fig|1220511.6.peg.899	CDS	ALWU01000005.1	74512	73760	-	753	Mobile element protein	- none -
fig|1220511.6.peg.901	CDS	ALWU01000005.1	75096	75230	+	135	Mobile element protein	- none -
fig|1220511.6.peg.902	CDS	ALWU01000005.1	75524	75655	+	132	Mobile element protein	- none -
fig|1220511.6.peg.903	CDS	ALWU01000005.1	76284	75850	-	435	Mobile element protein	- none -
fig|1220511.6.peg.944	CDS	ALWU01000005.1	111706	111897	+	192	Mobile element protein	- none -
fig|1220511.6.peg.945	CDS	ALWU01000005.1	111966	112748	+	783	Mobile element protein	- none -
fig|1220511.6.peg.948	CDS	ALWU01000005.1	114600	113848	-	753	Mobile element protein	- none -
fig|1220511.6.peg.955	CDS	ALWU01000005.1	120253	119471	-	783	Mobile element protein	- none -
fig|1220511.6.peg.956	CDS	ALWU01000005.1	120513	120322	-	192	Mobile element protein	- none -
fig|1220511.6.peg.967	CDS	ALWU01000005.1	132323	132192	-	132	Mobile element protein	- none -
fig|1220511.6.peg.968	CDS	ALWU01000005.1	132751	132617	-	135	Mobile element protein	- none -
fig|1220511.6.peg.969	CDS	ALWU01000005.1	133075	132959	-	117	Mobile element protein	- none -
fig|1220511.6.peg.971	CDS	ALWU01000005.1	133903	133739	-	165	Mobile element protein	- none -
fig|1220511.6.peg.972	CDS	ALWU01000005.1	134204	133908	-	297	Mobile element protein	- none -
fig|1220511.6.peg.983	CDS	ALWU01000005.1	146558	146677	+	120	Mobile element protein	- none -
fig|1220511.6.peg.990	CDS	ALWU01000005.1	152037	151783	-	255	Mobile element protein	- none -
fig|1220511.6.peg.997	CDS	ALWU01000005.1	157573	157836	+	264	Mobile element protein	- none -
fig|1220511.6.peg.998	CDS	ALWU01000005.1	158044	158178	+	135	Mobile element protein	- none -
fig|1220511.6.peg.999	CDS	ALWU01000005.1	158472	158603	+	132	Mobile element protein	- none -
fig|1220511.6.peg.1002	CDS	ALWU01000005.1	159420	160052	+	633	Mobile element protein	- none -

fig|1220511.6.peg.1015	CDS	ALWU01000005.1	175722	174523	-	1200	Mobile element protein	- none -
fig|1220511.6.peg.1016	CDS	ALWU01000005.1	176263	176072	-	192	Mobile element protein	- none -
fig|1220511.6.peg.1017	CDS	ALWU01000005.1	176970	176311	-	660	Mobile element protein	- none -
fig|1220511.6.peg.1033	CDS	ALWU01000005.1	186831	187583	+	753	Mobile element protein	- none -
fig|1220511.6.peg.1035	CDS	ALWU01000005.1	189109	188660	-	450	Mobile element protein	- none -
fig|1220511.6.peg.1037	CDS	ALWU01000005.1	189422	189574	+	153	Mobile element protein	- none -
fig|1220511.6.peg.1044	CDS	ALWU01000005.1	193794	193516	-	279	Mobile element protein	- none -
fig|1220511.6.peg.1045	CDS	ALWU01000005.1	194091	194297	+	207	Mobile element protein	- none -
fig|1220511.6.peg.1046	CDS	ALWU01000005.1	194305	194994	+	690	Mobile element protein	- none -
fig|1220511.6.peg.1090	CDS	ALWU01000006.1	13400	13876	+	477	Mobile element protein	- none -
fig|1220511.6.peg.1093	CDS	ALWU01000006.1	15257	15448	+	192	Mobile element protein	- none -
fig|1220511.6.peg.1094	CDS	ALWU01000006.1	15517	16299	+	783	Mobile element protein	- none -
fig|1220511.6.peg.1102	CDS	ALWU01000006.1	26817	26906	+	90	Mobile element protein	- none -
fig|1220511.6.peg.1103	CDS	ALWU01000006.1	26916	27140	+	225	Mobile element protein	- none -
fig|1220511.6.peg.1104	CDS	ALWU01000006.1	27172	27333	+	162	Mobile element protein	- none -
fig|1220511.6.peg.1105	CDS	ALWU01000006.1	27405	27632	+	228	Mobile element protein	- none -
fig|1220511.6.peg.1106	CDS	ALWU01000006.1	27625	27930	+	306	Mobile element protein	- none -
fig|1220511.6.peg.1109	CDS	ALWU01000006.1	30985	30290	-	696	Mobile element protein	- none -
fig|1220511.6.peg.1117	CDS	ALWU01000006.1	34414	35013	+	600	Mobile element protein	- none -
fig|1220511.6.peg.1143	CDS	ALWU01000008.1	8373	7591	-	783	Mobile element protein	- none -
fig|1220511.6.peg.1144	CDS	ALWU01000008.1	8633	8442	-	192	Mobile element protein	- none -
fig|1220511.6.peg.1177	CDS	ALWU01000008.1	38367	38095	-	273	Mobile element protein	- none -
fig|1220511.6.peg.1213	CDS	ALWU01000008.1	76751	76461	-	291	Mobile element protein	- none -

fig|1220511.6.peg.1215	CDS	ALWU01000008.1	77819	78367	+	549	Mobile element protein	- none -
fig|1220511.6.peg.1216	CDS	ALWU01000008.1	78507	78749	+	243	Mobile element protein	- none -
fig|1220511.6.peg.1217	CDS	ALWU01000008.1	78957	79091	+	135	Mobile element protein	- none -
fig|1220511.6.peg.1227	CDS	ALWU01000008.1	86923	87129	+	207	Mobile element protein	- none -
fig|1220511.6.peg.1228	CDS	ALWU01000008.1	87137	87826	+	690	Mobile element protein	- none -
fig|1220511.6.peg.1229	CDS	ALWU01000008.1	88296	88120	-	177	Mobile element protein	- none -
fig|1220511.6.peg.1258	CDS	ALWU01000008.1	121359	120919	-	441	Mobile element protein	- none -
fig|1220511.6.peg.1259	CDS	ALWU01000008.1	121573	121367	-	207	Mobile element protein	- none -
fig|1220511.6.peg.1278	CDS	ALWU01000008.1	137147	137338	+	192	Mobile element protein	- none -
fig|1220511.6.peg.1279	CDS	ALWU01000008.1	137407	137895	+	489	Mobile element protein	- none -
fig|1220511.6.peg.1280	CDS	ALWU01000008.1	137853	138188	+	336	Mobile element protein	- none -
fig|1220511.6.peg.1316	CDS	ALWU01000008.1	169494	169913	+	420	Mobile element protein	- none -
fig|1220511.6.peg.1318	CDS	ALWU01000008.1	170793	170659	-	135	Mobile element protein	- none -
fig|1220511.6.peg.1319	CDS	ALWU01000008.1	171117	171001	-	117	Mobile element protein	- none -
fig|1220511.6.peg.1329	CDS	ALWU01000008.1	176330	176683	+	354	Mobile element protein	- none -
fig|1220511.6.peg.1330	CDS	ALWU01000008.1	176879	176998	+	120	Mobile element protein	- none -
fig|1220511.6.peg.1334	CDS	ALWU01000008.1	177924	178202	+	279	Mobile element protein	- none -
fig|1220511.6.peg.1359	CDS	ALWU01000010.1	2644	3129	+	486	Mobile element protein	- none -
fig|1220511.6.peg.1364	CDS	ALWU01000010.1	7529	7329	-	201	Mobile element protein	- none -
fig|1220511.6.peg.1365	CDS	ALWU01000010.1	7795	8004	+	210	Mobile element protein	- none -
fig|1220511.6.peg.1367	CDS	ALWU01000010.1	8905	9195	+	291	Mobile element protein	- none -
fig|1220511.6.peg.1369	CDS	ALWU01000010.1	9558	9788	+	231	Mobile element protein	- none -
fig|1220511.6.peg.1370	CDS	ALWU01000010.1	9785	10522	+	738	Mobile element protein	- none -

fig|1220511.6.peg.1375	CDS	ALWU01000010.1	16761	16952	+	192	Mobile element protein	- none -
fig|1220511.6.peg.1376	CDS	ALWU01000010.1	17021	17803	+	783	Mobile element protein	- none -
fig|1220511.6.peg.1408	CDS	ALWU01000010.1	50302	50051	-	252	Mobile element protein	- none -
fig|1220511.6.peg.1409	CDS	ALWU01000010.1	51024	50620	-	405	Mobile element protein	- none -
fig|1220511.6.peg.1410	CDS	ALWU01000010.1	51239	51054	-	186	Mobile element protein	- none -
fig|1220511.6.peg.1422	CDS	ALWU01000010.1	59385	58696	-	690	Mobile element protein	- none -
fig|1220511.6.peg.1423	CDS	ALWU01000010.1	59599	59393	-	207	Mobile element protein	- none -
fig|1220511.6.peg.1424	CDS	ALWU01000010.1	60029	59631	-	399	Mobile element protein	- none -
fig|1220511.6.peg.1425	CDS	ALWU01000010.1	60870	60298	-	573	Mobile element protein	- none -
fig|1220511.6.peg.1427	CDS	ALWU01000010.1	61511	61675	+	165	Mobile element protein	- none -
fig|1220511.6.peg.1429	CDS	ALWU01000010.1	61960	62106	+	147	Mobile element protein	- none -
fig|1220511.6.peg.1432	CDS	ALWU01000010.1	64820	65329	+	510	Mobile element protein	- none -
fig|1220511.6.peg.1464	CDS	ALWU01000010.1	96463	96615	+	153	Mobile element protein	- none -
fig|1220511.6.peg.1496	CDS	ALWU01000010.1	124352	124543	+	192	Mobile element protein	- none -
fig|1220511.6.peg.1497	CDS	ALWU01000010.1	124612	125394	+	783	Mobile element protein	- none -
fig|1220511.6.peg.1498	CDS	ALWU01000010.1	125681	125517	-	165	Mobile element protein	- none -
fig|1220511.6.peg.1519	CDS	ALWU01000010.1	139281	139487	+	207	Mobile element protein	- none -
fig|1220511.6.peg.1520	CDS	ALWU01000010.1	139495	140184	+	690	Mobile element protein	- none -
fig|1220511.6.peg.1532	CDS	ALWU01000010.1	148970	149722	+	753	Mobile element protein	- none -
fig|1220511.6.peg.1533	CDS	ALWU01000010.1	149966	150085	+	120	Mobile element protein	- none -
fig|1220511.6.peg.1545	CDS	ALWU01000010.1	159626	159480	-	147	Mobile element protein	- none -
fig|1220511.6.peg.1547	CDS	ALWU01000010.1	160075	159911	-	165	Mobile element protein	- none -
fig|1220511.6.peg.1550	CDS	ALWU01000010.1	161910	162047	+	138	Mobile element protein	- none -

fig|1220511.6.peg.1576	CDS	ALWU01000010.1	188072	188263	+	192	Mobile element protein	- none -
fig|1220511.6.peg.1577	CDS	ALWU01000010.1	188332	189114	+	783	Mobile element protein	- none -
fig|1220511.6.peg.1591	CDS	ALWU01000010.1	198550	198663	+	114	Mobile element protein	- none -
fig|1220511.6.peg.1592	CDS	ALWU01000010.1	198734	198931	+	198	Mobile element protein	- none -
fig|1220511.6.peg.1597	CDS	ALWU01000010.1	204452	204234	-	219	Mobile element protein	- none -
fig|1220511.6.peg.1598	CDS	ALWU01000010.1	204786	204487	-	300	Mobile element protein	- none -
fig|1220511.6.peg.1606	CDS	ALWU01000010.1	210161	211360	+	1200	Mobile element protein	- none -
fig|1220511.6.peg.1621	CDS	ALWU01000010.1	222489	222013	-	477	Mobile element protein	- none -
fig|1220511.6.peg.1635	CDS	ALWU01000010.1	231331	230378	-	954	Mobile element protein	- none -
fig|1220511.6.peg.1636	CDS	ALWU01000010.1	231643	231461	-	183	Mobile element protein	- none -
fig|1220511.6.peg.1697	CDS	ALWU01000010.1	292023	290779	-	1245	Mobile element protein	- none -
fig|1220511.6.peg.1711	CDS	ALWU01000010.1	305934	305152	-	783	Mobile element protein	- none -
fig|1220511.6.peg.1712	CDS	ALWU01000010.1	306194	306003	-	192	Mobile element protein	- none -
fig|1220511.6.peg.1727	CDS	ALWU01000010.1	315542	316051	+	510	Mobile element protein	- none -
fig|1220511.6.peg.1737	CDS	ALWU01000010.1	323950	323738	-	213	Mobile element protein	- none -
fig|1220511.6.peg.1738	CDS	ALWU01000010.1	324441	325640	+	1200	Mobile element protein	- none -
fig|1220511.6.peg.1741	CDS	ALWU01000010.1	326276	326464	+	189	Mobile element protein	- none -
fig|1220511.6.peg.1761	CDS	ALWU01000010.1	350833	350714	-	120	Mobile element protein	- none -
fig|1220511.6.peg.1781	CDS	ALWU01000010.1	364925	364806	-	120	Mobile element protein	- none -
fig|1220511.6.peg.1782	CDS	ALWU01000010.1	365057	364935	-	123	Mobile element protein	- none -
fig|1220511.6.peg.1783	CDS	ALWU01000010.1	365483	365079	-	405	Mobile element protein	- none -
fig|1220511.6.peg.1784	CDS	ALWU01000010.1	365660	365755	+	96	Mobile element protein	- none -
fig|1220511.6.peg.1788	CDS	ALWU01000010.1	369184	369321	+	138	Mobile element protein	- none -

	fig|1220511.6.peg.1791	CDS	ALWU01000010.1	370529	370681	+	153	Mobile element protein	- none -
	fig|1220511.6.peg.1793	CDS	ALWU01000010.1	371696	371986	+	291	Mobile element protein	- none -
	fig|1220511.6.peg.1794	CDS	ALWU01000010.1	372932	372798	-	135	Mobile element protein	- none -
	fig|1220511.6.peg.1795	CDS	ALWU01000010.1	373382	373140	-	243	Mobile element protein	- none -
	fig|1220511.6.peg.1802	CDS	ALWU01000010.1	378247	378507	+	261	Mobile element protein	- none -
	fig|1220511.6.peg.1803	CDS	ALWU01000010.1	378779	379006	+	228	Mobile element protein	- none -
	fig|1220511.6.peg.1804	CDS	ALWU01000010.1	379098	379268	+	171	Mobile element protein	- none -
	fig|1220511.6.peg.1874	CDS	ALWU01000010.1	442603	442469	-	135	Mobile element protein	- none -
	fig|1220511.6.peg.1911	CDS	ALWU01000010.1	478879	478097	-	783	Mobile element protein	- none -
	fig|1220511.6.peg.1912	CDS	ALWU01000010.1	479139	478948	-	192	Mobile element protein	- none -
	fig|1220511.6.peg.1931	CDS	ALWU01000010.1	502449	502096	-	354	Mobile element protein	- none -
	fig|1220511.6.peg.1935	CDS	ALWU01000010.1	505265	505119	-	147	Mobile element protein	- none -
	fig|1220511.6.peg.1936	CDS	ALWU01000010.1	505539	505399	-	141	Mobile element protein	- none -
wWb	fig|96496.9.peg.245	CDS	NZ_NJBR02000019.1	3174	2374	-	801	Mobile element protein	- none -
	fig|96496.9.peg.655	CDS	NZ_NJBR02000049.1	78	320	+	243	Mobile element protein	- none -
	fig|96496.9.peg.656	CDS	NZ_NJBR02000049.1	997	1215	+	219	Mobile element protein	- none -
	fig|96496.9.peg.681	CDS	NZ_NJBR02000054.1	3735	4562	+	822	Mobile element protein	- none -
	fig|96496.9.peg.832	CDS	NZ_NJBR02000069.1	17486	17124	-	360	Mobile element protein	- none -
	fig|96496.9.peg.835	CDS	NZ_NJBR02000071.1	1091	693	-	399	Mobile element protein	- none -
	fig|96496.9.peg.895	CDS	NZ_NJBR02000073.1	20475	21638	+	1152	Mobile element protein	- none -
	fig|96496.9.peg.1056	CDS	NZ_NJBR02000090.1	2858	2995	+	138	Mobile element protein	- none -

Table S9. List of potential prophage region detected using PHASTER. The color indicated the completeness of the prophage region: red for incomplet, blue for questionable and green for intact. The draft genomes are indicated by a star.

genome	Region	Region Length	Completeness	Score	# Total Proteins	Region Position	Most Common Phage	GC %
wBm	-	-	-	-	-	-	-	-
wBp	-	-	-	-	-	-	-	-
wCtub	-	-	-	-	-	-	-	-
wCauA	1	61Kb	intact	150	51	245484-306575 info_outline	PHAGE_Escher_vB_EcoM_ep3_NC_025430(10)	33.85%
	2	44.5Kb	intact	150	50	305256-349803 info_outline	PHAGE_Escher_vB_EcoM_ECO1230_10_NC_027995(12)	35.62%
	3	10.9Kb	questionable	70	9	732730-743690 info_outline	PHAGE_Paenib_Tripp_NC_028930(2)	36.32%
	4	15Kb	intact	100	18	999801-1014803 info_outline	PHAGE_Escher_vB_EcoM_ep3_NC_025430(5)	37.24%
	5	17.1Kb	questionable	90	22	1077288-1094462 info_outline	PHAGE_Escher_vB_EcoM_ep3_NC_025430(5)	36.39%
wCfeJ	-	-	-	-	-	-	-	-
wCfeT	1	40.5Kb	intact	150	46	1388834-1429333 info_outline	PHAGE_Escher_vB_EcoM_ECO1230_10_NC_027995(9)	36.41%
wCle	1	7.6Kb	intact	140	14	487551-495178 info_outline	PHAGE_Paenib_Tripp_NC_028930(2)	37.01%
	2	6.7Kb	questionable	80	11	857941-864678 info_outline	PHAGE_Paenib_Tripp_NC_028930(2)	35.53%
wDimm	-	-	-	-	-	-	-	-
wDcau	-	-	-	-	-	-	-	-
wFol	1	36.7Kb	intact	140	31	368051-404817 info_outline	PHAGE_Escher_vB_EcoM_ECO1230_10_NC_027995(6)	34.83%
	2	54.5Kb	intact	150	54	648544-703067 info_outline	PHAGE_Escher_vB_EcoM_ep3_NC_025430(11)	35.28%
	3	21.4Kb	questionable	90	19	709069-730486 info_outline	PHAGE_Escher_vB_EcoM_ep3_NC_025430(5)	35.30%
	4	9.3Kb	questionable	70	7	805472-814846 info_outline	PHAGE_Caulob_swift_NC_019411(2)	35.64%
	5	31.7Kb	intact	120	25	1056324-1088090 info_outline	PHAGE_Escher_vB_EcoM_ECO1230_10_NC_027995(6)	34.66%
	6	44.1Kb	intact	150	49	1115459-1159579 info_outline	PHAGE_Escher_vB_EcoM_ECO1230_10_NC_027995(11)	35.73%
	7	59.7Kb	intact	150	56	1200316-1260099 info_outline	PHAGE_Escher_vB_EcoM_ECO1230_10_NC_027995(10)	34.55%
wLbra*	-	-	-	-	-	-	-	-
wLsig	-	-	-	-	-	-	-	-
wLug*	wLug scaffold1	28.3Kb	incomplete	40	14	40679-69013 info_outline	PHAGE_Vibrio_VP58.5_NC_027981(4)	35.46%
	wLug scaffold1	8.8Kb	incomplete	20	8	437781-446632 info_outline	PHAGE_Shigel_SfIV_NC_022749(2)	36.60%
	wLug scaffold1	26.6Kb	questionable	80	18	840736-867413 info_outline	PHAGE_Lactob_phiPYB5_NC_027982(1)	31.43%
	wLug scaffold1	15.9Kb	incomplete	40	12	1068306-1084226 info_outline	PHAGE_Escher_vB_EcoM_ep3_NC_025430(5)	35.03%
	wLug scaffold2	8.9Kb	incomplete	60	12	302917-311874 info_outline	PHAGE_Escher_RCS47_NC_042128(2)	36.81%
	wLug scaffold2	41.1Kb	intact	150	51	319077-360264 info_outline	PHAGE_Escher_vB_EcoM_ep3_NC_025430(9)	35.44%
wMel	1	37.2Kb	intact	110	23	236286-273546 info_outline	PHAGE_Escher_vB_EcoM_ep3_NC_025430(4)	36.34%
	2	9.9Kb	questionable	70	14	548107-558081 info_outline	PHAGE_Pseudo_PPpW_3_NC_023006(6)	34.10%
	3	10.2Kb	incomplete	50	10	560258-570525 info_outline	PHAGE_Paenib_Tripp_NC_028930(2)	36.38%
	4	16.1Kb	questionable	80	18	626320-642425 info_outline	PHAGE_Escher_vB_EcoM_ep3_NC_025430(5)	35.04%
wMhie*	-	-	-	-	-	-	-	-
wNfla*	1	22.2Kb	intact	140	30	25289-47574 info_outline	PHAGE_Escher_vB_EcoM_ep3_NC_025430(9)	37.02%

	2	36.4Kb	intact	100	23	134523-171016 info_outline	PHAGE_Escher_vB_EcoM_ep3_NC_025430(6)	35.76%
	3	15.7Kb	incomplete	30	17	192739-208472 info_outline	PHAGE_Vibrio_VP58.5_NC_027981(4)	35.34%
	4	12.3Kb	incomplete	50	17	301484-313844 info_outline	PHAGE_Pseudo_PPpW_3_NC_023006(3)	33.58%
	5	11.5Kb	incomplete	50	12	934607-946146 info_outline	PHAGE_Escher_vB_EcoM_ep3_NC_025430(4)	34.55%
	6	6.6Kb	incomplete	60	10	985436-992091 info_outline	PHAGE_Escher_vB_EcoM_ep3_NC_025430(3)	34.83%
wOo	-	-	-	-	-	-	-	-
wOv	-	-	-	-	-	-	-	-
wPpe*	1	11.9Kb	incomplete	50	11	12404-24395 info_outline	PHAGE_Agroba_Atu_ph07_NC_042013(2)	32.81%
wPip	1	21Kb	questionable	80	24	247653-268692 info_outline	PHAGE_Vibrio_vB_VpaM_MAR	35.29%
	2	23.9Kb	questionable	80	24	317593-341555 info_outline	PHAGE_Vibrio_vB_VpaM_MAR	35.33%
	3	38Kb	questionable	90	22	337503-375565 info_outline	PHAGE_Vibrio_vB_VpaM_MAR	34.77%
	4	40.1Kb	questionable	80	21	424334-464512 info_outline	PHAGE_Vibrio_vB_VpaM_MAR	34.17%
	5	22.3Kb	incomplete	60	24	465011-487365 info_outline	PHAGE_Yersin_413C	35.14%
	6	42.7Kb	intact	150	47	1373737-1416457 info_outline	PHAGE_Vibrio_vB_VpaM_MAR	35.06%
wstri*	wstri scaffold1	7.5Kb	incomplete	50	13	82896-90494 info_outline	PHAGE_Vibrio_vB_VpaM_MAR_NC_019722(2)	36.10%
	wstri scaffold1	14.8Kb	incomplete	50	23	94364-109251 info_outline	PHAGE_Escher_vB_EcoM_ECOO78_NC_041926(5)	35.73%
	wstri scaffold1	61.3Kb	intact	150	72	233933-295249 info_outline	PHAGE_Escher_vB_EcoM_ep3_NC_025430(8)	33.75%
	wstri scaffold1	17.6Kb	incomplete	60	15	1288808-1306494 info_outline	PHAGE_Escher_vB_EcoM_ep3_NC_025430(5)	29.58%
	wstri scaffold1	18.1Kb	questionable	70	29	1581451-1599644 info_outline	PHAGE_Escher_vB_EcoM_ECOO78_NC_041926(5)	36.45%
	wstri scaffold2	59.3Kb	intact	150	59	5544-64915 info_outline	PHAGE_Escher_vB_EcoM_ep3_NC_025430(10)	34.96%
	wstri scaffold2	10.7Kb	incomplete	20	16	97729-108512 info_outline	PHAGE_Escher_vB_EcoM_ECOO78_NC_041926(5)	34.46%
wTpre	1	8Kb	incomplete	30	9	660724-668752 info_outline	PHAGE_Cellul_phi17:1_NC_021795(1)	36.48%
wVulC*	wVulC_contig01	9.4Kb	questionable	90	18	119159-128605 info_outline	PHAGE_Bacill_PfEFR_5_NC_031055(1)	34.02%
	wVulC_contig02	17.3Kb	intact	110	22	8417-25727 info_outline	PHAGE_Escher_vB_EcoM_ep3_NC_025430(6)	34.58%
	wVulC_contig04	15.6Kb	incomplete	40	11	21780-37451 info_outline	PHAGE_Vibrio_vB_VpaM_MAR_NC_019722(3)	35.99%
	wVulC_contig05	23.2Kb	questionable	90	24	85659-108896 info_outline	PHAGE_Escher_vB_EcoM_ep3_NC_025430(4)	36.34%
	wVulC_contig05	6.3Kb	incomplete	40	12	188660-194994 info_outline	PHAGE_Prochl_P_HM2_NC_015284(1)	35.41%
	wVulC_contig06	6.6Kb	incomplete	50	9	2027-8648 info_outline	PHAGE_Entero_Arya_NC_031048(5)	35.46%
	wVulC_contig08	23.3Kb	incomplete	40	22	121973-145317 info_outline	PHAGE_Bacill_G_NC_023719(2)	34.44%
	wVulC_contig09	9.1Kb	incomplete	60	13	26-9201 info_outline	PHAGE_Entero_Arya_NC_031048(5)	37.02%
	wVulC_contig10	34.4Kb	intact	110	31	189796-224236 info_outline	PHAGE_Shigel_SfIV_NC_022749(2)	34.28%
	wVulC_contig10	45.1Kb	intact	150	58	227250-272417 info_outline	PHAGE_Escher_vB_EcoM_ep3_NC_025430(9)	35.37%
wWb*	-	-	-	-	-	-	-	-

Table S10. List of phage-like genes detected by RAST pipeline.

genome	Feature ID	Type	Contig	Start	Stop	Strand	Length (bp)	Function
wBm	fig|292805.13.peg.289	CDS	wBm_reseq_reversed	194277	194104	-	174	Phage uncharacterized protein
	fig|292805.13.peg.290	CDS	wBm_reseq_reversed	194440	194261	-	180	Phage uncharacterized protein
	fig|292805.13.peg.292	CDS	wBm_reseq_reversed	195156	194971	-	186	some similarities to phage related proteins
	fig|292805.13.peg.392	CDS	wBm_reseq_reversed	264389	264264	-	126	Phage major capsid protein
	fig|292805.13.peg.393	CDS	wBm_reseq_reversed	264678	264415	-	264	Phage major capsid protein
	fig|292805.13.peg.551	CDS	wBm_reseq_reversed	376109	376360	+	252	phage major capsid protein, HK97 family
	fig|292805.13.peg.829	CDS	wBm_reseq_reversed	557961	557776	-	186	some similarities to phage related proteins
	fig|292805.13.peg.996	CDS	wBm_reseq_reversed	673208	673393	+	186	some similarities to phage related proteins
	fig|292805.13.peg.998	CDS	wBm_reseq_reversed	673924	674103	+	180	Phage uncharacterized protein
	fig|292805.13.peg.999	CDS	wBm_reseq_reversed	674087	674260	+	174	Phage uncharacterized protein
	fig|292805.13.peg.1217	CDS	wBm_reseq_reversed	813513	813370	-	144	Phage Host Specificity Protein
	fig|292805.13.peg.1276	CDS	wBm_reseq_reversed	854793	854620	-	174	Phage uncharacterized protein
	fig|292805.13.peg.1277	CDS	wBm_reseq_reversed	854956	854777	-	180	Phage uncharacterized protein
	fig|292805.13.peg.1279	CDS	wBm_reseq_reversed	855672	855487	-	186	some similarities to phage related proteins
wBp	fig|96495.5.peg.513	CDS	NZ_CP050521.1	419977	420096	+	120	Phage uncharacterized protein
	fig|96495.5.peg.514	CDS	NZ_CP050521.1	420080	420247	+	168	Phage uncharacterized protein
	fig|96495.5.peg.648	CDS	NZ_CP050521.1	532215	532048	-	168	Phage uncharacterized protein
	fig|96495.5.peg.649	CDS	NZ_CP050521.1	532318	532199	-	120	Phage uncharacterized protein
	fig|96495.5.peg.944	CDS	NZ_CP050521.1	776704	776537	-	168	Phage uncharacterized protein
	fig|96495.5.peg.945	CDS	NZ_CP050521.1	776807	776688	-	120	Phage uncharacterized protein
	fig|96495.5.peg.1033	CDS	NZ_CP050521.1	846814	846689	-	126	Phage major capsid protein
	fig|96495.5.peg.1277	CDS	NZ_CP050521.1	1048705	1048911	+	207	phage major capsid protein, HK97 family
wCauA	fig|2591635.5.peg.10	CDS	CP041215.1	8020	8319	+	300	some similarities to phage related proteins
	fig|2591635.5.peg.11	CDS	CP041215.1	8386	9483	+	1098	some similarities to phage related proteins
	fig|2591635.5.peg.15	CDS	CP041215.1	12227	13420	+	1194	Phage portal protein
	fig|2591635.5.peg.106	CDS	CP041215.1	97850	96399	-	1452	some similarities to phage related proteins
	fig|2591635.5.peg.278	CDS	CP041215.1	254553	255971	+	1419	Phage head, portal protein B
	fig|2591635.5.peg.279	CDS	CP041215.1	255968	256999	+	1032	Phage head, head-tail preconnector protease C
	fig|2591635.5.peg.280	CDS	CP041215.1	257077	257445	+	369	Phage head, head-DNA stabilization protein D
	fig|2591635.5.peg.281	CDS	CP041215.1	257471	258475	+	1005	Phage head, major capsid protein E
	fig|2591635.5.peg.283	CDS	CP041215.1	258779	259300	+	522	Phage minor tail protein Z
	fig|2591635.5.peg.284	CDS	CP041215.1	259297	259770	+	474	Phage protein
	fig|2591635.5.peg.285	CDS	CP041215.1	259757	260221	+	465	Phage baseplate assembly protein V
	fig|2591635.5.peg.287	CDS	CP041215.1	260503	260838	+	336	Phage baseplate assembly protein GpW
	fig|2591635.5.peg.288	CDS	CP041215.1	260841	261641	+	801	Phage baseplate assembly protein J
	fig|2591635.5.peg.289	CDS	CP041215.1	261920	263086	+	1167	Phage tail formation protein I
	fig|2591635.5.peg.292	CDS	CP041215.1	264470	264910	+	441	Phage protein

fig|2591635.5.peg.293	CDS	CP041215.1	264903	266393	+	1491	prophage LambdaW1, site-specific recombinase, resolvase family
fig|2591635.5.peg.321	CDS	CP041215.1	295196	294261	-	936	Phage tail formation protein D
fig|2591635.5.peg.322	CDS	CP041215.1	295406	295197	-	210	Phage protein
fig|2591635.5.peg.323	CDS	CP041215.1	295756	295409	-	348	prophage P2W3, tail protein U, putative
fig|2591635.5.peg.324	CDS	CP041215.1	298044	295756	-	2289	Phage tail length tape-measure protein T
fig|2591635.5.peg.325	CDS	CP041215.1	298414	298160	-	255	Phage tail protein E
fig|2591635.5.peg.326	CDS	CP041215.1	298832	298449	-	384	Phage tail tube protein FII
fig|2591635.5.peg.327	CDS	CP041215.1	299881	299375	-	507	Phage tail tube protein FII
fig|2591635.5.peg.338	CDS	CP041215.1	310107	309907	-	201	prophage P2W3, tail protein X, putative
fig|2591635.5.peg.339	CDS	CP041215.1	310507	310091	-	417	Phage tail protein
fig|2591635.5.peg.340	CDS	CP041215.1	312691	310511	-	2181	Phage tail length tape-measure protein T
fig|2591635.5.peg.343	CDS	CP041215.1	313572	313072	-	501	Phage tail tube protein FII
fig|2591635.5.peg.344	CDS	CP041215.1	314745	313594	-	1152	Phage tail sheath protein FI
fig|2591635.5.peg.358	CDS	CP041215.1	327588	327929	+	342	Phage protein
fig|2591635.5.peg.361	CDS	CP041215.1	331475	331957	+	483	Phage head, terminase subunit Nu1
fig|2591635.5.peg.363	CDS	CP041215.1	333458	335284	+	1827	Phage head, terminase DNA packaging protein A
fig|2591635.5.peg.365	CDS	CP041215.1	335513	336919	+	1407	Phage head, portal protein B
fig|2591635.5.peg.366	CDS	CP041215.1	336919	337953	+	1035	Phage head, head-tail preconnector protease C
fig|2591635.5.peg.367	CDS	CP041215.1	338021	338389	+	369	Phage head, head-DNA stabilization protein D
fig|2591635.5.peg.368	CDS	CP041215.1	338422	339426	+	1005	Phage head, major capsid protein E
fig|2591635.5.peg.371	CDS	CP041215.1	341227	341445	+	219	putative phage related protein
fig|2591635.5.peg.372	CDS	CP041215.1	341446	341943	+	498	Phage tail, component Z
fig|2591635.5.peg.373	CDS	CP041215.1	341940	342413	+	474	Phage protein
fig|2591635.5.peg.374	CDS	CP041215.1	342400	342864	+	465	Phage baseplate assembly protein V
fig|2591635.5.peg.377	CDS	CP041215.1	344222	344548	+	327	Phage baseplate assembly protein GpW
fig|2591635.5.peg.378	CDS	CP041215.1	344551	345336	+	786	Phage baseplate assembly protein J
fig|2591635.5.peg.379	CDS	CP041215.1	345336	346496	+	1161	Phage tail formation protein I
fig|2591635.5.peg.382	CDS	CP041215.1	347885	348322	+	438	Phage protein
fig|2591635.5.peg.383	CDS	CP041215.1	348273	348758	+	486	prophage LambdaW1, site-specific recombinase, resolvase family
fig|2591635.5.peg.944	CDS	CP041215.1	879470	882796	+	3327	Phage Host Specificity Protein
fig|2591635.5.peg.1058	CDS	CP041215.1	997233	997703	+	471	Phage protein
fig|2591635.5.peg.1059	CDS	CP041215.1	997707	998318	+	612	Phage head, terminase subunit Nu1
fig|2591635.5.peg.1061	CDS	CP041215.1	999801	1001633	+	1833	Phage head, terminase DNA packaging protein A
fig|2591635.5.peg.1064	CDS	CP041215.1	1002917	1004338	+	1422	Phage head, portal protein B
fig|2591635.5.peg.1065	CDS	CP041215.1	1004335	1005372	+	1038	Phage head, head-tail preconnector protease C
fig|2591635.5.peg.1066	CDS	CP041215.1	1005440	1005811	+	372	Phage head, head-DNA stabilization protein D
fig|2591635.5.peg.1067	CDS	CP041215.1	1005837	1006529	+	693	Phage head, major capsid protein E
fig|2591635.5.peg.1069	CDS	CP041215.1	1006926	1007423	+	498	Phage minor tail protein Z
fig|2591635.5.peg.1070	CDS	CP041215.1	1007404	1007877	+	474	Phage protein
fig|2591635.5.peg.1071	CDS	CP041215.1	1007864	1008319	+	456	Phage baseplate assembly protein V
fig|2591635.5.peg.1073	CDS	CP041215.1	1008588	1008923	+	336	Phage baseplate assembly protein

	fig|2591635.5.peg.1074	CDS	CP041215.1	1008933	1009730	+	798	Phage baseplate assembly protein GpJ
	fig|2591635.5.peg.1075	CDS	CP041215.1	1009737	1010954	+	1218	Phage tail formation protein I
	fig|2591635.5.peg.1082	CDS	CP041215.1	1017033	1018187	+	1155	Phage tail sheath protein FI
	fig|2591635.5.peg.1084	CDS	CP041215.1	1019792	1020298	+	507	Phage tail tube protein FII
	fig|2591635.5.peg.1118	CDS	CP041215.1	1057192	1058643	+	1452	some similarities to phage related proteins
	fig|2591635.5.peg.1137	CDS	CP041215.1	1073915	1074391	+	477	Phage protein
	fig|2591635.5.peg.1139	CDS	CP041215.1	1075755	1076237	+	483	Phage head, terminase subunit Nu1
	fig|2591635.5.peg.1141	CDS	CP041215.1	1077738	1079564	+	1827	Phage head, terminase DNA packaging protein A
	fig|2591635.5.peg.1145	CDS	CP041215.1	1080548	1081957	+	1410	Phage head, portal protein B
	fig|2591635.5.peg.1146	CDS	CP041215.1	1081957	1082988	+	1032	Phage head, head-tail preconnector protease C
	fig|2591635.5.peg.1147	CDS	CP041215.1	1083047	1083502	+	456	Phage head, head-DNA stabilization protein D
	fig|2591635.5.peg.1148	CDS	CP041215.1	1083507	1084511	+	1005	Phage head, major capsid protein E
	fig|2591635.5.peg.1151	CDS	CP041215.1	1087067	1087369	+	303	Phage minor tail protein Z
	fig|2591635.5.peg.1152	CDS	CP041215.1	1087366	1087839	+	474	Phage protein
	fig|2591635.5.peg.1153	CDS	CP041215.1	1087826	1088290	+	465	Phage baseplate assembly protein V
	fig|2591635.5.peg.1155	CDS	CP041215.1	1088572	1088907	+	336	Phage baseplate assembly protein GpW
	fig|2591635.5.peg.1156	CDS	CP041215.1	1088910	1089710	+	801	Phage baseplate assembly protein J
	fig|2591635.5.peg.1157	CDS	CP041215.1	1089989	1091155	+	1167	Phage tail formation protein I
	fig|2591635.5.peg.1160	CDS	CP041215.1	1092539	1092979	+	441	Phage protein
	fig|2591635.5.peg.1161	CDS	CP041215.1	1092972	1094462	+	1491	prophage LambdaW1, site-specific recombinase, resolvase family
	fig|2591635.5.peg.1259	CDS	CP041215.1	1199236	1198037	-	1200	Phage major capsid protein
	fig|2591635.5.peg.1342	CDS	CP041215.1	1274393	1274629	+	237	some similarities to phage related proteins
	fig|2591635.5.peg.1343	CDS	CP041215.1	1274670	1275803	+	1134	some similarities to phage related proteins
wCtub	-	-	-	-	-	-	-	-
wCfeJ	fig|212123.3.peg.171	CDS	CP051157.1	159434	159297	-	138	Phage portal protein
	fig|212123.3.peg.172	CDS	CP051157.1	160027	159575	-	453	Phage portal protein
	fig|212123.3.peg.293	CDS	CP051157.1	301523	300540	-	984	Phage integrase
	fig|212123.3.peg.498	CDS	CP051157.1	511005	512183	+	1179	Phage portal protein
	fig|212123.3.peg.780	CDS	CP051157.1	788877	790409	+	1533	some similarities to phage related proteins
	fig|212123.3.peg.792	CDS	CP051157.1	801831	800350	-	1482	some similarities to phage related proteins
	fig|212123.3.peg.825	CDS	CP051157.1	837780	837514	-	267	prophage LambdaW1, site-specific recombinase, resolvase family
	fig|212123.3.peg.914	CDS	CP051157.1	942443	942817	+	375	Phage Host Specificity Protein
	fig|212123.3.peg.916	CDS	CP051157.1	945050	947941	+	2892	Phage Host Specificity Protein
	fig|212123.3.peg.986	CDS	CP051157.1	1015314	1014112	-	1203	Phage major capsid protein
	fig|212123.3.peg.1022	CDS	CP051157.1	1057430	1058869	+	1440	Phage tail formation protein I
	fig|212123.3.peg.1146	CDS	CP051157.1	1171611	1171465	-	147	prophage LambdaW1, site-specific recombinase, resolvase family
wCfeT	fig|212123.4.peg.375	CDS	CP051156.1	371618	372637	+	1020	Phage portal protein
	fig|212123.4.peg.386	CDS	CP051156.1	385298	387463	+	2166	Phage Host Specificity Protein
	fig|212123.4.peg.387	CDS	CP051156.1	387529	388323	+	795	Phage Host Specificity Protein
	fig|212123.4.peg.399	CDS	CP051156.1	398722	399882	+	1161	Phage major capsid protein
	fig|212123.4.peg.705	CDS	CP051156.1	707133	708503	+	1371	Phage tail fiber protein

	fig|212123.4.peg.1240	CDS	CP051156.1	1243041	1244219	+	1179	some similarities to phage related proteins
	fig|212123.4.peg.1241	CDS	CP051156.1	1244232	1244375	+	144	Phage uncharacterized protein
	fig|212123.4.peg.1298	CDS	CP051156.1	1300553	1301638	+	1086	Phage tail fiber protein
	fig|212123.4.peg.1407	CDS	CP051156.1	1391897	1391094	-	804	Phage baseplate assembly protein GpJ
	fig|212123.4.peg.1408	CDS	CP051156.1	1392229	1391894	-	336	Phage baseplate assembly protein GpW
	fig|212123.4.peg.1410	CDS	CP051156.1	1393058	1392597	-	462	Phage baseplate assembly protein V
	fig|212123.4.peg.1411	CDS	CP051156.1	1393521	1393048	-	474	Phage protein
	fig|212123.4.peg.1421	CDS	CP051156.1	1405308	1405820	+	513	Phage protein
	fig|212123.4.peg.1423	CDS	CP051156.1	1407318	1407797	+	480	Phage head, terminase subunit Nu1
	fig|212123.4.peg.1425	CDS	CP051156.1	1409394	1411223	+	1830	Phage head, terminase DNA packaging protein A
	fig|212123.4.peg.1427	CDS	CP051156.1	1411608	1413023	+	1416	Phage head, portal protein B
	fig|212123.4.peg.1428	CDS	CP051156.1	1413020	1414135	+	1116	Phage head, head-tail preconnector protease C
	fig|212123.4.peg.1429	CDS	CP051156.1	1414218	1414592	+	375	Phage head, head-DNA stabilization protein D
	fig|212123.4.peg.1430	CDS	CP051156.1	1414595	1415599	+	1005	Phage head, major capsid protein E
	fig|212123.4.peg.1432	CDS	CP051156.1	1416003	1416515	+	513	prophage LambdaW5, minor tail protein Z, putative
	fig|212123.4.peg.1440	CDS	CP051156.1	1423315	1424514	+	1200	Phage tail sheath protein FI
	fig|212123.4.peg.1441	CDS	CP051156.1	1424564	1425070	+	507	Phage tail tube protein FII
	fig|212123.4.peg.1442	CDS	CP051156.1	1425111	1425368	+	258	Phage tail protein E
	fig|212123.4.peg.1443	CDS	CP051156.1	1425478	1427823	+	2346	Phage tail length tape-measure protein T
	fig|212123.4.peg.1444	CDS	CP051156.1	1427823	1428179	+	357	phage-related tail formation protein U
	fig|212123.4.peg.1445	CDS	CP051156.1	1428176	1428391	+	216	Phage tail protein GpX
	fig|212123.4.peg.1446	CDS	CP051156.1	1428392	1429333	+	942	Phage tail formation protein D
wCle	fig|246273.9.peg.151	CDS	wCle_NZ_AP013028	144250	145662	+	1413	ankyrin repeat domain protein
	fig|246273.9.peg.169	CDS	wCle_NZ_AP013028	158182	157943	-	240	ankyrin repeat domain protein
	fig|246273.9.peg.299	CDS	wCle_NZ_AP013028	262640	264079	+	1440	ankyrin repeat domain protein
	fig|246273.9.peg.306	CDS	wCle_NZ_AP013028	268534	269130	+	597	Ankyrin
	fig|246273.9.peg.307	CDS	wCle_NZ_AP013028	269365	269934	+	570	prophage LambdaW1, ankyrin repeat domain protein
	fig|246273.9.peg.313	CDS	wCle_NZ_AP013028	274012	275277	+	1266	ankyrin repeat domain protein
	fig|246273.9.peg.532	CDS	wCle_NZ_AP013028	481043	480465	-	579	Ankyrin
	fig|246273.9.peg.698	CDS	wCle_NZ_AP013028	620688	621938	+	1251	ankyrin repeat domain protein
	fig|246273.9.peg.703	CDS	wCle_NZ_AP013028	625421	625684	+	264	ankyrin repeat domain protein
	fig|246273.9.peg.759	CDS	wCle_NZ_AP013028	676109	674745	-	1365	ankyrin repeat domain protein
	fig|246273.9.peg.920	CDS	wCle_NZ_AP013028	816276	815854	-	423	prophage LambdaW1, ankyrin repeat domain protein
	fig|246273.9.peg.921	CDS	wCle_NZ_AP013028	816422	816273	-	150	prophage LambdaW1, ankyrin repeat domain protein
	fig|246273.9.peg.922	CDS	wCle_NZ_AP013028	817421	816657	-	765	Ankyrin
	fig|246273.9.peg.948	CDS	wCle_NZ_AP013028	836503	837048	+	546	ankyrin repeat domain protein
	fig|246273.9.peg.1081	CDS	wCle_NZ_AP013028	948026	948271	+	246	ankyrin repeat domain protein
	fig|246273.9.peg.1231	CDS	wCle_NZ_AP013028	1089572	1088325	-	1248	Ankyrin repeat domain protein
	fig|246273.9.peg.1245	CDS	wCle_NZ_AP013028	1099171	1099581	+	411	ankyrin domain protein ank2
	fig|246273.9.peg.1247	CDS	wCle_NZ_AP013028	1101100	1101756	+	657	ankyrin repeat protein, putative
	fig|246273.9.peg.1372	CDS	wCle_NZ_AP013028	1214854	1214645	-	210	ankyrin repeat domain protein

	fig|246273.9.peg.1373	CDS	wCle_NZ_AP013028	1215789	1214950	-		840	ankyrin repeat domain protein
wDimm	-	-	-	-	-	-	-		-
wDcau	-	-	-	-	-	-	-		-
wFol	fig|169402.10.peg.230	CDS	wFol_NZ_CP015510	240820	242073	+		1254	Phage major capsid protein
	fig|169402.10.peg.300	CDS	wFol_NZ_CP015510	318830	318399	-		432	prophage LambdaW1, site-specific recombinase, resolvase family
	fig|169402.10.peg.365	CDS	wFol_NZ_CP015510	385331	384984	-		348	prophage P2W3, tail protein U, putative
	fig|169402.10.peg.372	CDS	wFol_NZ_CP015510	390734	390228	-		507	prophage P2W3, contractile tail tube protein
	fig|169402.10.peg.373	CDS	wFol_NZ_CP015510	391928	390753	-		1176	Phage tail sheath monomer
	fig|169402.10.peg.403	CDS	wFol_NZ_CP015510	419737	418085	-		1653	Phage integrase, site-specific serine recombinase
	fig|169402.10.peg.404	CDS	wFol_NZ_CP015510	420173	419721	-		453	Putative bacteriophage-related protein
	fig|169402.10.peg.604	CDS	wFol_NZ_CP015510	659568	659362	-		207	prophage P2W3, tail protein X, putative
	fig|169402.10.peg.605	CDS	wFol_NZ_CP015510	659921	659565	-		357	prophage P2W3, tail protein U, putative
	fig|169402.10.peg.606	CDS	wFol_NZ_CP015510	662251	659921	-		2331	Phage tail length tape-measure protein
	fig|169402.10.peg.610	CDS	wFol_NZ_CP015510	663740	663234	-		507	prophage P2W3, contractile tail tube protein
	fig|169402.10.peg.611	CDS	wFol_NZ_CP015510	664934	663759	-		1176	Phage tail sheath monomer
	fig|169402.10.peg.632	CDS	wFol_NZ_CP015510	682428	682916	+		489	Phage protein
	fig|169402.10.peg.636	CDS	wFol_NZ_CP015510	686563	688389	+		1827	Phage terminase, large subunit
	fig|169402.10.peg.640	CDS	wFol_NZ_CP015510	689334	690746	+		1413	Phage portal protein
	fig|169402.10.peg.643	CDS	wFol_NZ_CP015510	692331	693332	+		1002	Phage protein
	fig|169402.10.peg.645	CDS	wFol_NZ_CP015510	693723	694235	+		513	prophage LambdaW5, minor tail protein Z, putative
	fig|169402.10.peg.649	CDS	wFol_NZ_CP015510	695417	695752	+		336	prophage LambdaW5, baseplate assembly protein W, putative
	fig|169402.10.peg.650	CDS	wFol_NZ_CP015510	695755	696564	+		810	prophage LambdaW1, baseplate assembly protein J, putative
	fig|169402.10.peg.668	CDS	wFol_NZ_CP015510	712200	712688	+		489	Phage protein
	fig|169402.10.peg.672	CDS	wFol_NZ_CP015510	716312	718141	+		1830	Phage terminase, large subunit
	fig|169402.10.peg.674	CDS	wFol_NZ_CP015510	718546	719958	+		1413	Phage portal protein
	fig|169402.10.peg.677	CDS	wFol_NZ_CP015510	721560	722561	+		1002	Phage protein
	fig|169402.10.peg.679	CDS	wFol_NZ_CP015510	722992	723507	+		516	prophage LambdaW5, minor tail protein Z, putative
	fig|169402.10.peg.683	CDS	wFol_NZ_CP015510	724685	725020	+		336	prophage LambdaW5, baseplate assembly protein W, putative
	fig|169402.10.peg.689	CDS	wFol_NZ_CP015510	728834	730486	+		1653	Phage integrase, site-specific serine recombinase
	fig|169402.10.peg.730	CDS	wFol_NZ_CP015510	786766	787959	+		1194	Phage portal protein
	fig|169402.10.peg.757	CDS	wFol_NZ_CP015510	807757	811089	+		3333	Phage Host Specificity Protein
	fig|169402.10.peg.1007	CDS	wFol_NZ_CP015510	1068113	1067901	-		213	prophage P2W3, tail protein X, putative
	fig|169402.10.peg.1008	CDS	wFol_NZ_CP015510	1068466	1068110	-		357	prophage P2W3, tail protein U, putative
	fig|169402.10.peg.1009	CDS	wFol_NZ_CP015510	1070796	1068466	-		2331	Phage tail length tape-measure protein
	fig|169402.10.peg.1012	CDS	wFol_NZ_CP015510	1074008	1073502	-		507	prophage P2W3, contractile tail tube protein
	fig|169402.10.peg.1013	CDS	wFol_NZ_CP015510	1075221	1074022	-		1200	Phage tail sheath monomer
	fig|169402.10.peg.1045	CDS	wFol_NZ_CP015510	1119089	1118280	-		810	prophage LambdaW1, baseplate assembly protein J, putative
	fig|169402.10.peg.1046	CDS	wFol_NZ_CP015510	1119427	1119092	-		336	prophage LambdaW5, baseplate assembly protein W, putative
	fig|169402.10.peg.1050	CDS	wFol_NZ_CP015510	1121124	1120609	-		516	prophage LambdaW5, minor tail protein Z, putative
	fig|169402.10.peg.1052	CDS	wFol_NZ_CP015510	1122530	1121526	-		1005	Phage protein
	fig|169402.10.peg.1055	CDS	wFol_NZ_CP015510	1125436	1124030	-		1407	Phage portal protein

	fig|169402.10.peg.1058	CDS	wFol_NZ_CP015510	1128534	1126714	-	1821	Phage terminase, large subunit
	fig|169402.10.peg.1062	CDS	wFol_NZ_CP015510	1132659	1132171	-	489	Phage protein
	fig|169402.10.peg.1084	CDS	wFol_NZ_CP015510	1153660	1154841	+	1182	Phage tail sheath monomer
	fig|169402.10.peg.1085	CDS	wFol_NZ_CP015510	1154888	1155394	+	507	prophage P2W3, contractile tail tube protein
	fig|169402.10.peg.1088	CDS	wFol_NZ_CP015510	1155802	1158075	+	2274	Phage tail length tape-measure protein
	fig|169402.10.peg.1089	CDS	wFol_NZ_CP015510	1158075	1158431	+	357	prophage P2W3, tail protein U, putative
	fig|169402.10.peg.1090	CDS	wFol_NZ_CP015510	1158428	1158637	+	210	prophage P2W3, tail protein X, putative
	fig|169402.10.peg.1132	CDS	wFol_NZ_CP015510	1201478	1201272	-	207	prophage P2W3, tail protein X, putative
	fig|169402.10.peg.1133	CDS	wFol_NZ_CP015510	1201822	1201475	-	348	prophage P2W3, tail protein U, putative
	fig|169402.10.peg.1136	CDS	wFol_NZ_CP015510	1205158	1204652	-	507	prophage P2W3, contractile tail tube protein
	fig|169402.10.peg.1137	CDS	wFol_NZ_CP015510	1206384	1205188	-	1197	Phage tail sheath monomer
	fig|169402.10.peg.1144	CDS	wFol_NZ_CP015510	1222334	1221816	-	519	Phage protein
	fig|169402.10.peg.1150	CDS	wFol_NZ_CP015510	1227862	1227674	-	189	putative phage related protein
	fig|169402.10.peg.1173	CDS	wFol_NZ_CP015510	1247975	1248310	+	336	prophage LambdaW5, baseplate assembly protein W, putative
	fig|169402.10.peg.1179	CDS	wFol_NZ_CP015510	1252124	1253776	+	1653	Phage integrase, site-specific serine recombinase
	fig|169402.10.peg.1198	CDS	wFol_NZ_CP015510	1291238	1289676	-	1563	some similarities to phage related proteins
	fig|169402.10.peg.1417	CDS	wFol_NZ_CP015510	1523771	1520121	-	3651	Phage tail fiber protein
wMel	fig|163164.1.peg.234	CDS	wMel_NC_002978	248608	249837	+	1230	prophage LambdaW1, DNA methylase
	fig|163164.1.peg.236	CDS	wMel_NC_002978	250515	252341	+	1827	Phage terminase, large subunit
	fig|163164.1.peg.242	CDS	wMel_NC_002978	258344	259345	+	1002	elements of external origin; phage-related functions and prophages
	fig|163164.1.peg.244	CDS	wMel_NC_002978	260904	259882	-	1023	prophage LambdaW1, transposase, IS110 family
	fig|163164.1.peg.246	CDS	wMel_NC_002978	261229	261726	+	498	prophage LambdaW1, minor tail protein Z, putative
	fig|163164.1.peg.249	CDS	wMel_NC_002978	262919	263245	+	327	prophage LambdaW1, baseplate assembly protein W, putative
	fig|163164.1.peg.250	CDS	wMel_NC_002978	263248	264057	+	810	prophage LambdaW1, baseplate assembly protein J, putative
	fig|163164.1.peg.252	CDS	wMel_NC_002978	265391	265993	+	603	prophage LambdaW1, ankyrin repeat domain protein
	fig|163164.1.peg.253	CDS	wMel_NC_002978	266255	266845	+	591	prophage LambdaW1, ankyrin repeat domain protein
	fig|163164.1.peg.254	CDS	wMel_NC_002978	266878	267306	+	429	Putative bacteriophage-related protein
	fig|163164.1.peg.255	CDS	wMel_NC_002978	267309	268811	+	1503	prophage LambdaW1, site-specific recombinase, resolvase family
	fig|163164.1.peg.258	CDS	wMel_NC_002978	271048	270374	-	675	prophage LambdaW1, ankyrin repeat domain protein
	fig|163164.1.peg.259	CDS	wMel_NC_002978	273255	271150	-	2106	prophage LambdaW1, ankyrin repeat domain protein
	fig|163164.1.peg.339	CDS	wMel_NC_002978	361426	364761	+	3336	Phage Host Specificity Protein
	fig|163164.1.peg.401	CDS	wMel_NC_002978	431961	431428	-	534	phage prohead protease
	fig|163164.1.peg.409	CDS	wMel_NC_002978	440219	439020	-	1200	Phage major capsid protein #Fam0006
	fig|163164.1.peg.504	CDS	wMel_NC_002978	552598	551654	-	945	prophage P2W3, tail protein D, putative
	fig|163164.1.peg.505	CDS	wMel_NC_002978	552808	552599	-	210	prophage P2W3, tail protein X, putative
	fig|163164.1.peg.506	CDS	wMel_NC_002978	553155	552805	-	351	prophage P2W3, tail protein U, putative
	fig|163164.1.peg.507	CDS	wMel_NC_002978	555194	553152	-	2043	Phage tail length tape-measure protein
	fig|163164.1.peg.511	CDS	wMel_NC_002978	556883	556386	-	498	prophage P2W3, contractile tail tube protein
	fig|163164.1.peg.528	CDS	wMel_NC_002978	572259	573485	+	1227	prophage LambdaW4, DNA methylase
	fig|163164.1.peg.530	CDS	wMel_NC_002978	573982	575463	+	1482	prophage LambdaW4, ankyrin repeat domain protein
	fig|163164.1.peg.531	CDS	wMel_NC_002978	575460	577283	+	1824	Phage terminase, large subunit

	fig|163164.1.peg.535	CDS	wMel_NC_002978	579610	580671	+		1062	prophage LambdaW4, minor capsid protein C, putative
	fig|163164.1.peg.537	CDS	wMel_NC_002978	581154	582158	+		1005	elements of external origin; phage-related functions and prophages
	fig|163164.1.peg.563	CDS	wMel_NC_002978	623343	626243	+		2901	prophage LambdaW5, ankyrin repeat domain protein
	fig|163164.1.peg.564	CDS	wMel_NC_002978	628119	626650	-		1470	prophage LambdaW5, site-specific recombinase, resolvase family
	fig|163164.1.peg.565	CDS	wMel_NC_002978	628553	628113	-		441	Putative bacteriophage-related protein
	fig|163164.1.peg.566	CDS	wMel_NC_002978	629024	628566	-		459	prophage LambdaW5, ankyrin repeat domain protein
	fig|163164.1.peg.567	CDS	wMel_NC_002978	629676	629050	-		627	prophage LambdaW5, ankyrin repeat domain protein
	fig|163164.1.peg.569	CDS	wMel_NC_002978	631903	631112	-		792	prophage LambdaW5, baseplate assembly protein J, putative
	fig|163164.1.peg.570	CDS	wMel_NC_002978	632232	631906	-		327	prophage LambdaW5, baseplate assembly protein W, putative
	fig|163164.1.peg.572	CDS	wMel_NC_002978	632970	632506	-		465	prophage LambdaW5, baseplate assembly protein V
	fig|163164.1.peg.574	CDS	wMel_NC_002978	633879	633430	-		450	prophage LambdaW5, minor tail protein Z, putative
	fig|163164.1.peg.673	CDS	wMel_NC_002978	726462	727391	+		930	site-specific recombinase, phage integrase family
	fig|163164.1.peg.903	CDS	wMel_NC_002978	976186	974993	-		1194	Phage portal protein
	fig|163164.1.peg.907	CDS	wMel_NC_002978	980557	979094	-		1464	some similarities to phage related proteins
	fig|163164.1.peg.1014	CDS	wMel_NC_002978	1082174	1083061	+		888	Phage-related protein
	fig|163164.1.peg.1029	CDS	wMel_NC_002978	1098113	1099099	+		987	site-specific recombinase, phage integrase family
	fig|163164.1.peg.1219	CDS	wMel_NC_002978	253852	254226	+		375	Phage portal protein # N-terminal fragment
	fig|163164.1.peg.1220	CDS	wMel_NC_002978	254208	255269	+		1062	Phage portal protein
	fig|163164.1.peg.1258	CDS	wMel_NC_002978	578196	579431	+		1236	Phage portal protein
	fig|163164.1.peg.1259	CDS	wMel_NC_002978	579419	579613	+		195	Phage portal protein # C-terminal fragment
wMhie	fig|1241303.4.peg.37	CDS	NODE_11105	168	305	+		138	Phage uncharacterized protein
	fig|1241303.4.peg.102	CDS	NODE_13107	72	830	+		759	Phage major capsid protein
	fig|1241303.4.peg.143	CDS	NODE_15112	229	498	+		270	Phage protein
	fig|1241303.4.peg.729	CDS	NODE_3460	1958	2785	+		828	Phage Host Specificity Protein
	fig|1241303.4.peg.730	CDS	NODE_3460	2751	3314	+		564	Phage Host Specificity Protein
	fig|1241303.4.peg.731	CDS	NODE_3460	3302	3865	+		564	Phage Host Specificity Protein
	fig|1241303.4.peg.732	CDS	NODE_3460	3867	5183	+		1317	Phage Host Specificity Protein
	fig|1241303.4.peg.1031	CDS	NODE_5322	3288	2809	-		480	Phage protein
	fig|1241303.4.peg.1079	CDS	NODE_5846	2739	2473	-		267	Phage protein
wLbra	-	-	-	-	-	-	-		-
wLsig	-	-	-	-	-	-	-		-
wLug	fig|1335053.6.peg.2	CDS	MUIY01000001.1	960	1520	+		561	Phage tail length tape-measure protein
	fig|1335053.6.peg.3	CDS	MUIY01000001.1	1520	1858	+		339	prophage P2W3, tail protein U, putative
	fig|1335053.6.peg.22	CDS	MUIY01000001.1	26889	26458	-		432	Phage protein
	fig|1335053.6.peg.25	CDS	MUIY01000001.1	29282	28263	-		1020	Phage tail formation protein I
	fig|1335053.6.peg.43	CDS	MUIY01000001.1	43093	42662	-		432	Phage protein
	fig|1335053.6.peg.46	CDS	MUIY01000001.1	45621	44467	-		1155	Phage tail formation protein I
	fig|1335053.6.peg.48	CDS	MUIY01000001.1	47238	47077	-		162	Phage head, portal protein B
	fig|1335053.6.peg.50	CDS	MUIY01000001.1	48805	47456	-		1350	Phage head, terminase DNA packaging protein A
	fig|1335053.6.peg.51	CDS	MUIY01000001.1	49452	48847	-		606	Phage head, major capsid protein E
	fig|1335053.6.peg.52	CDS	MUIY01000001.1	49859	49485	-		375	Phage head, head-DNA stabilization protein D

fig|1335053.6.peg.53	CDS	MUIY01000001.1	50979	49927	-	1053	Phage head, head-tail preconnector protease C
fig|1335053.6.peg.54	CDS	MUIY01000001.1	52235	50976	-	1260	Phage head, portal protein B
fig|1335053.6.peg.56	CDS	MUIY01000001.1	54263	54108	-	156	Phage head, portal protein B
fig|1335053.6.peg.59	CDS	MUIY01000001.1	56065	55529	-	537	Phage head, terminase DNA packaging protein A
fig|1335053.6.peg.61	CDS	MUIY01000001.1	58972	57611	-	1362	Phage head, terminase DNA packaging protein A
fig|1335053.6.peg.64	CDS	MUIY01000001.1	60923	60450	-	474	Phage head, terminase subunit Nu1
fig|1335053.6.peg.66	CDS	MUIY01000001.1	62731	62276	-	456	Phage protein
fig|1335053.6.peg.83	CDS	MUIY01000001.1	76016	76273	+	258	Phage tail protein E
fig|1335053.6.peg.85	CDS	MUIY01000001.1	76383	78647	+	2265	Phage tail length tape-measure protein T
fig|1335053.6.peg.86	CDS	MUIY01000001.1	78647	78994	+	348	phage-related tail formation protein U
fig|1335053.6.peg.87	CDS	MUIY01000001.1	78991	79200	+	210	Phage tail protein GpX
fig|1335053.6.peg.88	CDS	MUIY01000001.1	79201	80145	+	945	Phage tail formation protein D
fig|1335053.6.peg.127	CDS	MUIY01000001.1	127284	123955	-	3330	Phage Host Specificity Protein
fig|1335053.6.peg.395	CDS	MUIY01000001.1	377706	379028	+	1323	Phage tail fiber protein
fig|1335053.6.peg.397	CDS	MUIY01000001.1	380184	382616	+	2433	Phage tail fiber protein
fig|1335053.6.peg.400	CDS	MUIY01000001.1	385605	387056	+	1452	some similarities to phage related proteins
fig|1335053.6.peg.460	CDS	MUIY01000001.1	439214	437781	-	1434	some similarities to phage related proteins
fig|1335053.6.peg.655	CDS	MUIY01000001.1	608051	609064	+	1014	Phage tail fiber protein
fig|1335053.6.peg.656	CDS	MUIY01000001.1	609049	609207	+	159	Phage tail fiber protein
fig|1335053.6.peg.677	CDS	MUIY01000001.1	626036	624876	-	1161	Phage tail formation protein I
fig|1335053.6.peg.678	CDS	MUIY01000001.1	627493	626036	-	1458	Phage baseplate assembly protein J
fig|1335053.6.peg.922	CDS	MUIY01000001.1	852099	852242	+	144	prophage LambdaW1, baseplate assembly protein J, putative
fig|1335053.6.peg.1001	CDS	MUIY01000001.1	939456	938293	-	1164	Phage major capsid protein
fig|1335053.6.peg.1022	CDS	MUIY01000001.1	961963	961646	-	318	Phage protein
fig|1335053.6.peg.1137	CDS	MUIY01000001.1	1077559	1076399	-	1161	Phage tail formation protein I
fig|1335053.6.peg.1138	CDS	MUIY01000001.1	1078344	1077559	-	786	Phage baseplate assembly protein J
fig|1335053.6.peg.1139	CDS	MUIY01000001.1	1078682	1078347	-	336	Phage baseplate assembly protein GpW
fig|1335053.6.peg.1141	CDS	MUIY01000001.1	1079411	1078947	-	465	Phage baseplate assembly protein V
fig|1335053.6.peg.1142	CDS	MUIY01000001.1	1079871	1079398	-	474	Phage protein
fig|1335053.6.peg.1143	CDS	MUIY01000001.1	1080371	1079868	-	504	Phage minor tail protein Z
fig|1335053.6.peg.1145	CDS	MUIY01000001.1	1081237	1080773	-	465	Phage head, major capsid protein E
fig|1335053.6.peg.1146	CDS	MUIY01000001.1	1081658	1081227	-	432	Phage protein
fig|1335053.6.peg.1149	CDS	MUIY01000001.1	1084198	1083032	-	1167	Phage tail formation protein I
fig|1335053.6.peg.1150	CDS	MUIY01000001.1	1085259	1084477	-	783	Phage baseplate assembly protein J
fig|1335053.6.peg.1151	CDS	MUIY01000001.1	1085597	1085262	-	336	Phage baseplate assembly protein GpW
fig|1335053.6.peg.1153	CDS	MUIY01000001.1	1086314	1085817	-	498	Phage baseplate assembly protein V
fig|1335053.6.peg.1154	CDS	MUIY01000001.1	1086774	1086301	-	474	Phage protein
fig|1335053.6.peg.1155	CDS	MUIY01000001.1	1087274	1086771	-	504	Phage minor tail protein Z
fig|1335053.6.peg.1157	CDS	MUIY01000001.1	1088582	1087578	-	1005	Phage head, major capsid protein E
fig|1335053.6.peg.1158	CDS	MUIY01000001.1	1089029	1088619	-	411	Phage head, head-DNA stabilization protein D
fig|1335053.6.peg.1159	CDS	MUIY01000001.1	1090079	1089066	-	1014	Phage head, head-tail preconnector protease C

	fig|1335053.6.peg.1160	CDS	MUIY01000001.1	1091326	1090079	-	1248	Phage head, portal protein B
	fig|1335053.6.peg.1162	CDS	MUIY01000001.1	1095487	1093199	-	2289	Phage head, terminase DNA packaging protein A
	fig|1335053.6.peg.1164	CDS	MUIY01000001.1	1097451	1096987	-	465	Phage head, terminase subunit Nu1
	fig|1335053.6.peg.1166	CDS	MUIY01000001.1	1098055	1097585	-	471	Phage protein
	fig|1335053.6.peg.1196	CDS	MUIY01000001.1	1124794	1124651	-	144	prophage LambdaW1, baseplate assembly protein J, putative
	fig|1335053.6.peg.1200	CDS	MUIY01000001.1	1127909	1127487	-	423	Phage tail fiber protein
	fig|1335053.6.peg.1342	CDS	MUIY01000002.1	123586	122411	-	1176	Phage portal protein
	fig|1335053.6.peg.1551	CDS	MUIY01000002.1	321363	320932	-	432	Phage protein
	fig|1335053.6.peg.1554	CDS	MUIY01000002.1	323903	322737	-	1167	Phage tail formation protein I
	fig|1335053.6.peg.1555	CDS	MUIY01000002.1	324964	324182	-	783	Phage baseplate assembly protein J
	fig|1335053.6.peg.1556	CDS	MUIY01000002.1	325302	324967	-	336	Phage baseplate assembly protein GpW
	fig|1335053.6.peg.1559	CDS	MUIY01000002.1	327092	326628	-	465	Phage baseplate assembly protein V
	fig|1335053.6.peg.1560	CDS	MUIY01000002.1	327552	327079	-	474	Phage protein
	fig|1335053.6.peg.1561	CDS	MUIY01000002.1	328052	327549	-	504	Phage minor tail protein Z
	fig|1335053.6.peg.1563	CDS	MUIY01000002.1	329458	328454	-	1005	Phage head, major capsid protein E
	fig|1335053.6.peg.1564	CDS	MUIY01000002.1	329905	329495	-	411	Phage head, head-DNA stabilization protein D
	fig|1335053.6.peg.1565	CDS	MUIY01000002.1	330955	329942	-	1014	Phage head, head-tail preconnector protease C
	fig|1335053.6.peg.1566	CDS	MUIY01000002.1	332193	330955	-	1239	Phage head, portal protein B
	fig|1335053.6.peg.1567	CDS	MUIY01000002.1	333904	333434	-	471	Phage head, terminase DNA packaging protein A
	fig|1335053.6.peg.1568	CDS	MUIY01000002.1	335186	333990	-	1197	Phage head, terminase DNA packaging protein A
	fig|1335053.6.peg.1571	CDS	MUIY01000002.1	337137	336667	-	471	Phage head, terminase subunit Nu1
	fig|1335053.6.peg.1573	CDS	MUIY01000002.1	338945	338490	-	456	Phage protein
	fig|1335053.6.peg.1593	CDS	MUIY01000002.1	354335	355480	+	1146	Phage tail sheath protein FI
	fig|1335053.6.peg.1594	CDS	MUIY01000002.1	355496	356002	+	507	Phage tail tube protein FII
	fig|1335053.6.peg.1595	CDS	MUIY01000002.1	356085	356342	+	258	Phage tail protein E
	fig|1335053.6.peg.1597	CDS	MUIY01000002.1	356452	358758	+	2307	Phage tail length tape-measure protein T
	fig|1335053.6.peg.1598	CDS	MUIY01000002.1	358760	359107	+	348	phage-related tail formation protein U
	fig|1335053.6.peg.1599	CDS	MUIY01000002.1	359104	359313	+	210	Phage tail protein GpX
	fig|1335053.6.peg.1600	CDS	MUIY01000002.1	359314	360264	+	951	Phage tail formation protein D
wNfla	fig|1854759.6.peg.24	CDS	NZ_LYUW01000001.1	26459	26259	-	201	prophage P2W3, tail protein X, putative
	fig|1854759.6.peg.25	CDS	NZ_LYUW01000001.1	26850	26443	-	408	Phage tail protein
	fig|1854759.6.peg.26	CDS	NZ_LYUW01000001.1	29025	26854	-	2172	Phage tail length tape-measure protein T
	fig|1854759.6.peg.28	CDS	NZ_LYUW01000001.1	29907	29407	-	501	Phage tail tube protein FII
	fig|1854759.6.peg.29	CDS	NZ_LYUW01000001.1	31109	29922	-	1188	Phage tail sheath protein FI
	fig|1854759.6.peg.37	CDS	NZ_LYUW01000001.1	38416	37199	-	1218	Phage tail formation protein I
	fig|1854759.6.peg.38	CDS	NZ_LYUW01000001.1	39220	38423	-	798	Phage tail tip, host specificity protein J
	fig|1854759.6.peg.39	CDS	NZ_LYUW01000001.1	39565	39230	-	336	Phage baseplate assembly protein GpW
	fig|1854759.6.peg.41	CDS	NZ_LYUW01000001.1	40289	39834	-	456	Phage baseplate assembly protein V
	fig|1854759.6.peg.42	CDS	NZ_LYUW01000001.1	40749	40276	-	474	Phage protein
	fig|1854759.6.peg.43	CDS	NZ_LYUW01000001.1	41227	40730	-	498	Phage minor tail protein Z
	fig|1854759.6.peg.45	CDS	NZ_LYUW01000001.1	42628	41624	-	1005	Phage head, major capsid protein E

fig|1854759.6.peg.46	CDS	NZ_LYUW01000001.1	43026	42655	-	372	Phage head, head-DNA stabilization protein D
fig|1854759.6.peg.47	CDS	NZ_LYUW01000001.1	44124	43054	-	1071	Phage head, head-tail preconnector protease C
fig|1854759.6.peg.48	CDS	NZ_LYUW01000001.1	45539	44121	-	1419	Phage head, portal protein B
fig|1854759.6.peg.50	CDS	NZ_LYUW01000001.1	47574	45757	-	1818	Phage head, terminase DNA packaging protein A
fig|1854759.6.peg.52	CDS	NZ_LYUW01000001.1	49544	49074	-	471	Phage head, terminase subunit Nu1
fig|1854759.6.peg.54	CDS	NZ_LYUW01000001.1	50171	49677	-	495	Phage protein
fig|1854759.6.peg.411	CDS	NZ_LYUW01000011.1	22014	23018	+	1005	some similarities to phage related proteins
fig|1854759.6.peg.419	CDS	NZ_LYUW01000012.1	9233	7737	-	1497	prophage LambdaW1, site-specific recombinase, resolvase family
fig|1854759.6.peg.420	CDS	NZ_LYUW01000012.1	9661	9227	-	435	Phage protein
fig|1854759.6.peg.421	CDS	NZ_LYUW01000012.1	10105	9704	-	402	prophage LambdaW1, ankyrin repeat domain protein
fig|1854759.6.peg.422	CDS	NZ_LYUW01000012.1	10236	10102	-	135	prophage LambdaW1, ankyrin repeat domain protein
fig|1854759.6.peg.425	CDS	NZ_LYUW01000012.1	13144	11984	-	1161	Phage tail formation protein I
fig|1854759.6.peg.426	CDS	NZ_LYUW01000012.1	13944	13144	-	801	Phage tail tip, host specificity protein J
fig|1854759.6.peg.427	CDS	NZ_LYUW01000012.1	14180	13947	-	234	Phage baseplate assembly protein GpW
fig|1854759.6.peg.430	CDS	NZ_LYUW01000012.1	16183	17331	+	1149	Phage tail sheath protein FI
fig|1854759.6.peg.431	CDS	NZ_LYUW01000012.1	17347	17853	+	507	Phage tail tube protein FII
fig|1854759.6.peg.436	CDS	NZ_LYUW01000012.1	18831	21407	+	2577	Phage tail length tape-measure protein T
fig|1854759.6.peg.437	CDS	NZ_LYUW01000012.1	21407	21661	+	255	prophage P2W3, tail protein U, putative
fig|1854759.6.peg.473	CDS	NZ_LYUW01000014.1	64	957	+	894	Phage head, portal protein B
fig|1854759.6.peg.474	CDS	NZ_LYUW01000014.1	970	2001	+	1032	Phage head, head-tail preconnector protease C
fig|1854759.6.peg.475	CDS	NZ_LYUW01000014.1	2079	2450	+	372	Phage head, head-DNA stabilization protein D
fig|1854759.6.peg.476	CDS	NZ_LYUW01000014.1	2488	3492	+	1005	Phage head, major capsid protein E
fig|1854759.6.peg.478	CDS	NZ_LYUW01000014.1	3800	4285	+	486	Phage minor tail protein Z
fig|1854759.6.peg.494	CDS	NZ_LYUW01000015.1	3321	3455	+	135	Phage head, portal protein B
fig|1854759.6.peg.556	CDS	NZ_LYUW01000018.1	18709	17765	-	945	Phage tail formation protein D
fig|1854759.6.peg.557	CDS	NZ_LYUW01000018.1	18919	18710	-	210	Phage tail protein GpX
fig|1854759.6.peg.558	CDS	NZ_LYUW01000018.1	19263	18916	-	348	phage-related tail formation protein U
fig|1854759.6.peg.729	CDS	NZ_LYUW01000028.1	2729	3445	+	717	Phage portal protein
fig|1854759.6.peg.730	CDS	NZ_LYUW01000028.1	3506	3922	+	417	Phage portal protein
fig|1854759.6.peg.788	CDS	NZ_LYUW01000032.1	2447	3646	+	1200	Phage major capsid protein
fig|1854759.6.peg.818	CDS	NZ_LYUW01000033.1	13262	12318	-	945	Phage tail formation protein D
fig|1854759.6.peg.819	CDS	NZ_LYUW01000033.1	13473	13264	-	210	Phage tail protein GpX
fig|1854759.6.peg.820	CDS	NZ_LYUW01000033.1	13616	13470	-	147	prophage P2W3, tail protein U, putative
fig|1854759.6.peg.1051	CDS	NZ_LYUW01000050.1	5312	4881	-	432	Phage protein
fig|1854759.6.peg.1054	CDS	NZ_LYUW01000050.1	7870	6704	-	1167	Phage tail formation protein I
fig|1854759.6.peg.1055	CDS	NZ_LYUW01000050.1	8960	8160	-	801	Phage baseplate assembly protein J
fig|1854759.6.peg.1056	CDS	NZ_LYUW01000050.1	9298	8963	-	336	Phage baseplate assembly protein GpW
fig|1854759.6.peg.1058	CDS	NZ_LYUW01000050.1	10020	9556	-	465	Phage baseplate assembly protein V
fig|1854759.6.peg.1059	CDS	NZ_LYUW01000050.1	10534	10007	-	528	Phage protein
fig|1854759.6.peg.1106	CDS	NZ_LYUW01000055.1	3379	4593	+	1215	Phage tail sheath protein FI
fig|1854759.6.peg.1107	CDS	NZ_LYUW01000055.1	4605	5111	+	507	Phage tail tube protein FII

	fig|1854759.6.peg.1110	CDS	NZ_LYUW01000055.1	5881	6138	+	258	Phage tail protein E
	fig|1854759.6.peg.1112	CDS	NZ_LYUW01000055.1	6239	8539	+	2301	Phage tail length tape-measure protein T
	fig|1854759.6.peg.1113	CDS	NZ_LYUW01000055.1	8539	8796	+	258	phage-related tail formation protein U
	fig|1854759.6.peg.1128	CDS	NZ_LYUW01000057.1	6823	3497	-	3327	Phage Host Specificity Protein
	fig|1854759.6.peg.1164	CDS	NZ_LYUW01000061.1	3348	4052	+	705	Phage tail sheath protein FI
	fig|1854759.6.peg.1165	CDS	NZ_LYUW01000061.1	4155	4550	+	396	Phage tail sheath protein FI
	fig|1854759.6.peg.1166	CDS	NZ_LYUW01000061.1	4571	5077	+	507	Phage tail tube protein FII
	fig|1854759.6.peg.1167	CDS	NZ_LYUW01000061.1	5166	5423	+	258	Phage tail protein E
	fig|1854759.6.peg.1169	CDS	NZ_LYUW01000061.1	5533	7668	+	2136	Phage tail length tape-measure protein T
	fig|1854759.6.peg.1257	CDS	NZ_LYUW01000073.1	22	168	+	147	phage-related tail formation protein U
	fig|1854759.6.peg.1258	CDS	NZ_LYUW01000073.1	165	374	+	210	Phage tail protein GpX
	fig|1854759.6.peg.1259	CDS	NZ_LYUW01000073.1	375	1319	+	945	Phage tail formation protein D
	fig|1854759.6.peg.1446	CDS	NZ_LYUW01000114.1	39	410	+	372	phage-related tail formation protein U
	fig|1854759.6.peg.1456	CDS	NZ_LYUW01000117.1	1101	472	-	630	some similarities to phage related proteins
	fig|1854759.6.peg.1474	CDS	NZ_LYUW01000126.1	1082	579	-	504	Phage protein
wOo	-	-	-	-	-	-	-	-
wOv	-	-	-	-	-	-	-	-
wPip	fig|955.1.peg.49	CDS	culex94e01.p1k	11967	11536	-	432	Putative bacteriophage-related protein
	fig|955.1.peg.359	CDS	culex12a01.q1k	2928	1495	-	1434	Phage-related protein
	fig|955.1.peg.449	CDS	culex17f04.q1k	4008	3799	-	210	prophage P2W3, tail protein X, putative
	fig|955.1.peg.450	CDS	culex17f04.q1k	4361	4005	-	357	prophage P2W3, tail protein U, putative
	fig|955.1.peg.451	CDS	culex17f04.q1k	6625	4361	-	2265	Phage tail length tape-measure protein
	fig|955.1.peg.456	CDS	culex147f12.p1k	1227	730	-	498	prophage LambdaW5, minor tail protein Z, putative
	fig|955.1.peg.459	CDS	culex147f12.p1k	2938	2567	-	372	PROBABLE BACTERIOPHAGE-RELATED PROTEIN
	fig|955.1.peg.463	CDS	culex69g03.p1k	1544	1224	-	321	PROBABLE BACTERIOPHAGE-RELATED PROTEIN
	fig|955.1.peg.475	CDS	culex140f11.p1k	691	2	-	690	Phage-related baseplate assembly protein
	fig|955.1.peg.476	CDS	culex140f11.p1k	927	694	-	234	Phage baseplate assembly protein W
	fig|955.1.peg.480	CDS	culex141a08.q1k	1867	1508	-	360	prophage P2W3, tail protein D, putative
	fig|955.1.peg.511	CDS	culex102f01.p1k	185	3	-	183	prophage LambdaW4, DNA methylase
	fig|955.1.peg.512	CDS	culex102f01.p1k	763	299	-	465	Putative bacteriophage-related protein
	fig|955.1.peg.527	CDS	culex102f01.p1k	17434	18612	+	1179	Phage tail sheath monomer
	fig|955.1.peg.528	CDS	culex102f01.p1k	18628	19134	+	507	Phage major tail tube protein
	fig|955.1.peg.533	CDS	culex102f01.p1k	20112	22403	+	2292	Phage tail length tape-measure protein
	fig|955.1.peg.534	CDS	culex102f01.p1k	22403	22750	+	348	prophage P2W3, tail protein U, putative
	fig|955.1.peg.535	CDS	culex102f01.p1k	22957	23901	+	945	Phage protein D
	fig|955.1.peg.547	CDS	culex102f01.p1k	33590	32799	-	792	Phage-related baseplate assembly protein
	fig|955.1.peg.548	CDS	culex102f01.p1k	33928	33593	-	336	Phage baseplate assembly protein
	fig|955.1.peg.552	CDS	culex102f01.p1k	35606	35109	-	498	prophage LambdaW5, minor tail protein Z, putative
	fig|955.1.peg.555	CDS	culex102f01.p1k	37412	37041	-	372	PROBABLE BACTERIOPHAGE-RELATED PROTEIN
	fig|955.1.peg.557	CDS	culex102f01.p1k	40018	38600	-	1419	Phage portal protein
	fig|955.1.peg.559	CDS	culex102f01.p1k	42077	40233	-	1845	Phage terminase, large subunit

	fig|955.1.peg.563	CDS	culex102f01.p1k	45831	45367	-	465	Putative bacteriophage-related protein
	fig|955.1.peg.567	CDS	culex102f01.p1k	50379	52436	+	2058	PROBABLE BACTERIOPHAGE-RELATED PROTEIN
	fig|955.1.peg.842	CDS	culex131h09.q1k	1367	750	-	618	Phage-related baseplate assembly protein
	fig|955.1.peg.843	CDS	culex50h05.p1k	6	1808	+	1803	PROBABLE BACTERIOPHAGE-RELATED PROTEIN
	fig|955.1.peg.853	CDS	culex40h12.p1k	4714	5175	+	462	prophage LambdaW4, terminase large subunit, putative
	fig|955.1.peg.870	CDS	culex40h12.p1k	33536	33132	-	405	Putative bacteriophage-related protein
	fig|955.1.peg.909	CDS	culex18h04.p1k	28	570	+	543	Phage-related tail protein
	fig|955.1.peg.910	CDS	culex18h04.p1k	570	926	+	357	prophage P2W3, tail protein U, putative
	fig|955.1.peg.911	CDS	culex18h04.p1k	923	1132	+	210	prophage P2W3, tail protein X, putative
	fig|955.1.peg.1182	CDS	culex79g04.p1k	60509	61684	+	1176	Phage portal protein
	fig|955.1.peg.1317	CDS	culex180d12.p1k	4625	4996	+	372	PROBABLE BACTERIOPHAGE-RELATED PROTEIN
	fig|955.1.peg.1320	CDS	culex180d12.p1k	6408	6899	+	492	prophage LambdaW5, minor tail protein Z, putative
	fig|955.1.peg.1338	CDS	culex180d12.p1k	26226	24031	-	2196	Phage Host Specificity Protein
	fig|955.1.peg.1372	CDS	culex143c03.p1k	16074	15643	-	432	Putative bacteriophage-related protein
	fig|955.1.peg.1377	CDS	culex143c03.p1k	21327	20542	-	786	Phage-related baseplate assembly protein
	fig|955.1.peg.1378	CDS	culex143c03.p1k	21665	21330	-	336	Phage baseplate assembly protein
	fig|955.1.peg.1382	CDS	culex143c03.p1k	23331	22840	-	492	prophage LambdaW5, minor tail protein Z, putative
	fig|955.1.peg.1386	CDS	culex143c03.p1k	25488	25117	-	372	PROBABLE BACTERIOPHAGE-RELATED PROTEIN
	fig|955.1.peg.1391	CDS	culex143c03.p1k	31843	31379	-	465	Putative bacteriophage-related protein
	fig|955.1.peg.1395	CDS	culex143c03.p1k	38991	37201	-	1791	PROBABLE BACTERIOPHAGE-RELATED PROTEIN
	fig|955.1.peg.1529	CDS	culex16c12.p1k	16431	17828	+	1398	Phage-related protein
wPpe	-	-	-	-	-	-	-	-
wstri	fig|368602.8.peg.1	CDS	MUIX01000001.1	2	211	+	210	Phage tail fiber protein
	fig|368602.8.peg.5	CDS	MUIX01000001.1	6352	5921	-	432	Phage protein
	fig|368602.8.peg.8	CDS	MUIX01000001.1	8932	7766	-	1167	Phage tail formation protein I
	fig|368602.8.peg.9	CDS	MUIX01000001.1	10010	9210	-	801	Phage baseplate assembly protein J
	fig|368602.8.peg.10	CDS	MUIX01000001.1	10348	10013	-	336	Phage baseplate assembly protein GpW
	fig|368602.8.peg.12	CDS	MUIX01000001.1	10794	10627	-	168	Phage baseplate assembly protein V
	fig|368602.8.peg.13	CDS	MUIX01000001.1	11507	11034	-	474	Phage protein
	fig|368602.8.peg.14	CDS	MUIX01000001.1	12016	11504	-	513	Phage minor tail protein Z
	fig|368602.8.peg.19	CDS	MUIX01000001.1	15546	15767	+	222	Phage major capsid protein
	fig|368602.8.peg.83	CDS	MUIX01000001.1	76352	76696	+	345	Phage protein
	fig|368602.8.peg.85	CDS	MUIX01000001.1	81034	81411	+	378	Phage head, terminase subunit Nu1
	fig|368602.8.peg.87	CDS	MUIX01000001.1	82896	84722	+	1827	Phage head, terminase DNA packaging protein A
	fig|368602.8.peg.90	CDS	MUIX01000001.1	85646	87052	+	1407	Phage head, portal protein B
	fig|368602.8.peg.91	CDS	MUIX01000001.1	87052	87480	+	429	Phage head, head-tail preconnector protease C
	fig|368602.8.peg.92	CDS	MUIX01000001.1	87607	88095	+	489	Phage head, head-tail preconnector protease C
	fig|368602.8.peg.93	CDS	MUIX01000001.1	88170	88541	+	372	Phage head, head-DNA stabilization protein D
	fig|368602.8.peg.94	CDS	MUIX01000001.1	88504	88653	+	150	Phage head, major capsid protein E
	fig|368602.8.peg.99	CDS	MUIX01000001.1	91161	90685	-	477	Phage protein
	fig|368602.8.peg.110	CDS	MUIX01000001.1	99602	98892	-	711	Phage tail formation protein D

fig|368602.8.peg.111	CDS	MUIX01000001.1	99812	99603	-	210	Phage tail protein GpX
fig|368602.8.peg.112	CDS	MUIX01000001.1	100156	99809	-	348	phage-related tail formation protein U
fig|368602.8.peg.113	CDS	MUIX01000001.1	102464	100158	-	2307	Phage tail length tape-measure protein T
fig|368602.8.peg.114	CDS	MUIX01000001.1	102831	102574	-	258	Phage tail protein E
fig|368602.8.peg.115	CDS	MUIX01000001.1	103420	102914	-	507	Phage tail tube protein FII
fig|368602.8.peg.116	CDS	MUIX01000001.1	103750	103436	-	315	Phage tail sheath protein FI
fig|368602.8.peg.117	CDS	MUIX01000001.1	104012	103704	-	309	Phage head, portal protein B
fig|368602.8.peg.119	CDS	MUIX01000001.1	106062	104227	-	1836	Phage head, terminase DNA packaging protein A
fig|368602.8.peg.121	CDS	MUIX01000001.1	108001	107531	-	471	Phage head, terminase subunit Nu1
fig|368602.8.peg.123	CDS	MUIX01000001.1	109830	109408	-	423	Phage protein
fig|368602.8.peg.149	CDS	MUIX01000001.1	133841	135016	+	1176	Phage tail sheath protein FI
fig|368602.8.peg.150	CDS	MUIX01000001.1	135032	135538	+	507	Phage tail tube protein FII
fig|368602.8.peg.151	CDS	MUIX01000001.1	135621	135878	+	258	Phage tail protein E
fig|368602.8.peg.153	CDS	MUIX01000001.1	135988	138408	+	2421	Phage tail length tape-measure protein T
fig|368602.8.peg.154	CDS	MUIX01000001.1	138408	138755	+	348	phage-related tail formation protein U
fig|368602.8.peg.155	CDS	MUIX01000001.1	138752	138961	+	210	Phage tail protein GpX
fig|368602.8.peg.156	CDS	MUIX01000001.1	138963	139913	+	951	Phage tail formation protein D
fig|368602.8.peg.178	CDS	MUIX01000001.1	168987	168556	-	432	Phage protein
fig|368602.8.peg.181	CDS	MUIX01000001.1	171533	170367	-	1167	Phage tail formation protein I
fig|368602.8.peg.182	CDS	MUIX01000001.1	172611	171811	-	801	Phage baseplate assembly protein J
fig|368602.8.peg.183	CDS	MUIX01000001.1	172949	172614	-	336	Phage baseplate assembly protein GpW
fig|368602.8.peg.185	CDS	MUIX01000001.1	173395	173228	-	168	Phage baseplate assembly protein V
fig|368602.8.peg.186	CDS	MUIX01000001.1	174108	173635	-	474	Phage protein
fig|368602.8.peg.187	CDS	MUIX01000001.1	174617	174105	-	513	Phage minor tail protein Z
fig|368602.8.peg.192	CDS	MUIX01000001.1	178147	178368	+	222	Phage major capsid protein
fig|368602.8.peg.197	CDS	MUIX01000001.1	182296	181301	-	996	Phage head, portal protein B
fig|368602.8.peg.201	CDS	MUIX01000001.1	185034	183211	-	1824	Phage head, terminase DNA packaging protein A
fig|368602.8.peg.203	CDS	MUIX01000001.1	186998	186519	-	480	Phage head, terminase subunit Nu1
fig|368602.8.peg.205	CDS	MUIX01000001.1	188785	188330	-	456	Phage protein
fig|368602.8.peg.206	CDS	MUIX01000001.1	190153	189422	-	732	Prophage antirepressor
fig|368602.8.peg.209	CDS	MUIX01000001.1	194485	195177	+	693	Phage tail formation protein I
fig|368602.8.peg.212	CDS	MUIX01000001.1	196557	196988	+	432	Phage protein
fig|368602.8.peg.223	CDS	MUIX01000001.1	214568	214062	-	507	Phage tail tube protein FII
fig|368602.8.peg.224	CDS	MUIX01000001.1	215761	214580	-	1182	Phage tail sheath protein FI
fig|368602.8.peg.246	CDS	MUIX01000001.1	237828	237397	-	432	Phage protein
fig|368602.8.peg.249	CDS	MUIX01000001.1	240360	239200	-	1161	Phage tail formation protein I
fig|368602.8.peg.250	CDS	MUIX01000001.1	241151	240360	-	792	Phage baseplate assembly protein J
fig|368602.8.peg.251	CDS	MUIX01000001.1	241387	241154	-	234	Phage baseplate assembly protein GpW
fig|368602.8.peg.253	CDS	MUIX01000001.1	242890	242777	-	114	Phage baseplate assembly protein GpW
fig|368602.8.peg.256	CDS	MUIX01000001.1	244691	244227	-	465	Phage baseplate assembly protein V
fig|368602.8.peg.257	CDS	MUIX01000001.1	245154	244678	-	477	Phage protein

fig|368602.8.peg.258	CDS	MUIX01000001.1	245672	245151	-	522	Phage minor tail protein Z
fig|368602.8.peg.260	CDS	MUIX01000001.1	246578	245973	-	606	Phage head, major capsid protein E
fig|368602.8.peg.265	CDS	MUIX01000001.1	250380	248065	-	2316	Phage tail length tape-measure protein T
fig|368602.8.peg.266	CDS	MUIX01000001.1	250747	250490	-	258	Phage tail protein E
fig|368602.8.peg.269	CDS	MUIX01000001.1	251870	251364	-	507	Phage tail tube protein FII
fig|368602.8.peg.270	CDS	MUIX01000001.1	253072	251888	-	1185	Phage tail sheath protein FI
fig|368602.8.peg.291	CDS	MUIX01000001.1	275913	275482	-	432	Phage protein
fig|368602.8.peg.294	CDS	MUIX01000001.1	278019	277327	-	693	Phage tail formation protein I
fig|368602.8.peg.296	CDS	MUIX01000001.1	281205	281107	-	99	Phage baseplate assembly protein GpW
fig|368602.8.peg.298	CDS	MUIX01000001.1	281923	281459	-	465	Phage baseplate assembly protein V
fig|368602.8.peg.299	CDS	MUIX01000001.1	282383	281910	-	474	Phage protein
fig|368602.8.peg.300	CDS	MUIX01000001.1	282871	282380	-	492	Phage minor tail protein Z
fig|368602.8.peg.301	CDS	MUIX01000001.1	283091	282873	-	219	putative phage related protein
fig|368602.8.peg.302	CDS	MUIX01000001.1	284268	283264	-	1005	Phage head, major capsid protein E
fig|368602.8.peg.303	CDS	MUIX01000001.1	284662	284294	-	369	Phage head, head-DNA stabilization protein D
fig|368602.8.peg.304	CDS	MUIX01000001.1	285783	284740	-	1044	Phage head, head-tail preconnector protease C
fig|368602.8.peg.305	CDS	MUIX01000001.1	286582	285770	-	813	Phage head, portal protein B
fig|368602.8.peg.306	CDS	MUIX01000001.1	287441	286659	-	783	Phage head, major capsid protein E
fig|368602.8.peg.307	CDS	MUIX01000001.1	287835	287467	-	369	Phage head, head-DNA stabilization protein D
fig|368602.8.peg.308	CDS	MUIX01000001.1	288956	287913	-	1044	Phage head, head-tail preconnector protease C
fig|368602.8.peg.309	CDS	MUIX01000001.1	290799	288943	-	1857	Phage head, major capsid protein E
fig|368602.8.peg.310	CDS	MUIX01000001.1	291193	290825	-	369	Phage head, head-DNA stabilization protein D
fig|368602.8.peg.311	CDS	MUIX01000001.1	292314	291271	-	1044	Phage head, head-tail preconnector protease C
fig|368602.8.peg.312	CDS	MUIX01000001.1	293719	292301	-	1419	Phage head, portal protein B
fig|368602.8.peg.316	CDS	MUIX01000001.1	295249	294701	-	549	Phage head, terminase DNA packaging protein A
fig|368602.8.peg.643	CDS	MUIX01000001.1	641854	640940	-	915	Phage portal protein
fig|368602.8.peg.645	CDS	MUIX01000001.1	644368	644141	-	228	Phage portal protein
fig|368602.8.peg.990	CDS	MUIX01000001.1	981690	982022	+	333	some similarities to phage related proteins
fig|368602.8.peg.1055	CDS	MUIX01000001.1	1035843	1035340	-	504	some similarities to phage related proteins
fig|368602.8.peg.1057	CDS	MUIX01000001.1	1038207	1037386	-	822	some similarities to phage related proteins
fig|368602.8.peg.1063	CDS	MUIX01000001.1	1046164	1042691	-	3474	Phage tail fiber protein
fig|368602.8.peg.1317	CDS	MUIX01000001.1	1293083	1291923	-	1161	Phage tail formation protein I
fig|368602.8.peg.1318	CDS	MUIX01000001.1	1293226	1293083	-	144	prophage LambdaW1, baseplate assembly protein J, putative
fig|368602.8.peg.1319	CDS	MUIX01000001.1	1293869	1293249	-	621	Phage baseplate assembly protein J
fig|368602.8.peg.1320	CDS	MUIX01000001.1	1294207	1293872	-	336	Phage baseplate assembly protein GpW
fig|368602.8.peg.1322	CDS	MUIX01000001.1	1294936	1294472	-	465	Phage baseplate assembly protein V
fig|368602.8.peg.1323	CDS	MUIX01000001.1	1295396	1294923	-	474	Phage protein
fig|368602.8.peg.1324	CDS	MUIX01000001.1	1295905	1295393	-	513	prophage LambdaW5, minor tail protein Z, putative
fig|368602.8.peg.1326	CDS	MUIX01000001.1	1297213	1296209	-	1005	Phage head, major capsid protein E
fig|368602.8.peg.1327	CDS	MUIX01000001.1	1297660	1297250	-	411	Phage head, head-DNA stabilization protein D
fig|368602.8.peg.1328	CDS	MUIX01000001.1	1298113	1297697	-	417	Phage head, head-tail preconnector protease C

fig|368602.8.peg.1331	CDS	MUIX01000001.1	1302009	1302863	+	855	Phage head, terminase DNA packaging protein A
fig|368602.8.peg.1332	CDS	MUIX01000001.1	1304089	1303619	-	471	Phage head, terminase subunit Nu1
fig|368602.8.peg.1334	CDS	MUIX01000001.1	1305918	1305496	-	423	Phage protein
fig|368602.8.peg.1355	CDS	MUIX01000001.1	1325332	1326513	+	1182	Phage tail sheath protein FI
fig|368602.8.peg.1356	CDS	MUIX01000001.1	1326529	1327035	+	507	Phage tail tube protein FII
fig|368602.8.peg.1357	CDS	MUIX01000001.1	1327118	1327375	+	258	Phage tail protein E
fig|368602.8.peg.1359	CDS	MUIX01000001.1	1327485	1329791	+	2307	Phage tail length tape-measure protein T
fig|368602.8.peg.1360	CDS	MUIX01000001.1	1329793	1330140	+	348	phage-related tail formation protein U
fig|368602.8.peg.1361	CDS	MUIX01000001.1	1330137	1330346	+	210	Phage tail protein GpX
fig|368602.8.peg.1362	CDS	MUIX01000001.1	1330347	1331297	+	951	Phage tail formation protein D
fig|368602.8.peg.1386	CDS	MUIX01000001.1	1367344	1367231	-	114	prophage LambdaW1, site-specific recombinase
fig|368602.8.peg.1390	CDS	MUIX01000001.1	1370517	1370086	-	432	Phage protein
fig|368602.8.peg.1393	CDS	MUIX01000001.1	1373097	1371931	-	1167	Phage tail formation protein I
fig|368602.8.peg.1394	CDS	MUIX01000001.1	1374175	1373375	-	801	Phage baseplate assembly protein J
fig|368602.8.peg.1395	CDS	MUIX01000001.1	1374513	1374178	-	336	Phage baseplate assembly protein GpW
fig|368602.8.peg.1397	CDS	MUIX01000001.1	1374959	1374792	-	168	Phage baseplate assembly protein V
fig|368602.8.peg.1398	CDS	MUIX01000001.1	1375672	1375199	-	474	Phage protein
fig|368602.8.peg.1399	CDS	MUIX01000001.1	1376181	1375669	-	513	Phage minor tail protein Z
fig|368602.8.peg.1525	CDS	MUIX01000001.1	1490714	1489785	-	930	Phage major capsid protein
fig|368602.8.peg.1612	CDS	MUIX01000001.1	1587343	1586666	-	678	Phage tail formation protein D
fig|368602.8.peg.1613	CDS	MUIX01000001.1	1587553	1587344	-	210	Phage tail protein GpX
fig|368602.8.peg.1614	CDS	MUIX01000001.1	1587897	1587550	-	348	phage-related tail formation protein U
fig|368602.8.peg.1615	CDS	MUIX01000001.1	1590205	1587899	-	2307	Phage tail length tape-measure protein T
fig|368602.8.peg.1616	CDS	MUIX01000001.1	1590572	1590315	-	258	Phage tail protein E
fig|368602.8.peg.1617	CDS	MUIX01000001.1	1591161	1590655	-	507	Phage tail tube protein FII
fig|368602.8.peg.1626	CDS	MUIX01000001.1	1597154	1596477	-	678	Phage tail formation protein D
fig|368602.8.peg.1627	CDS	MUIX01000001.1	1597364	1597155	-	210	Phage tail protein GpX
fig|368602.8.peg.1628	CDS	MUIX01000001.1	1597708	1597361	-	348	phage-related tail formation protein U
fig|368602.8.peg.1629	CDS	MUIX01000001.1	1599644	1597710	-	1935	Phage tail length tape-measure protein T
fig|368602.8.peg.1630	CDS	MUIX01000001.1	1600254	1599619	-	636	Phage tail length tape-measure protein T
fig|368602.8.peg.1632	CDS	MUIX01000002.1	326	117	-	210	Phage tail protein GpX
fig|368602.8.peg.1633	CDS	MUIX01000002.1	661	323	-	339	prophage P2W3, tail protein U, putative
fig|368602.8.peg.1634	CDS	MUIX01000002.1	3099	661	-	2439	Phage tail length tape-measure protein T
fig|368602.8.peg.1635	CDS	MUIX01000002.1	3334	3209	-	126	Phage tail protein E
fig|368602.8.peg.1639	CDS	MUIX01000002.1	6494	5544	-	951	Phage tail formation protein D
fig|368602.8.peg.1640	CDS	MUIX01000002.1	6705	6496	-	210	Phage tail protein GpX
fig|368602.8.peg.1641	CDS	MUIX01000002.1	7040	6702	-	339	prophage P2W3, tail protein U, putative
fig|368602.8.peg.1642	CDS	MUIX01000002.1	9478	7040	-	2439	Phage tail length tape-measure protein T
fig|368602.8.peg.1643	CDS	MUIX01000002.1	9845	9588	-	258	Phage tail protein E
fig|368602.8.peg.1644	CDS	MUIX01000002.1	10398	9892	-	507	Phage tail tube protein FII
fig|368602.8.peg.1645	CDS	MUIX01000002.1	10630	10427	-	204	Phage tail sheath protein FI

fig|368602.8.peg.1669	CDS	MUIX01000002.1	36941	36810	-	132	Phage protein
fig|368602.8.peg.1670	CDS	MUIX01000002.1	37240	37028	-	213	Phage protein
fig|368602.8.peg.1673	CDS	MUIX01000002.1	39766	38606	-	1161	Phage tail formation protein I
fig|368602.8.peg.1674	CDS	MUIX01000002.1	40566	39766	-	801	Phage baseplate assembly protein J
fig|368602.8.peg.1675	CDS	MUIX01000002.1	40664	40569	-	96	Phage baseplate assembly protein
fig|368602.8.peg.1677	CDS	MUIX01000002.1	42310	42197	-	114	Phage baseplate assembly protein GpW
fig|368602.8.peg.1680	CDS	MUIX01000002.1	44111	43647	-	465	Phage baseplate assembly protein V
fig|368602.8.peg.1681	CDS	MUIX01000002.1	44574	44098	-	477	Phage protein
fig|368602.8.peg.1682	CDS	MUIX01000002.1	45092	44571	-	522	Phage minor tail protein Z
fig|368602.8.peg.1685	CDS	MUIX01000002.1	47203	47090	-	114	Phage baseplate assembly protein GpW
fig|368602.8.peg.1687	CDS	MUIX01000002.1	47903	47460	-	444	Phage baseplate assembly protein V
fig|368602.8.peg.1688	CDS	MUIX01000002.1	48366	47890	-	477	Phage protein
fig|368602.8.peg.1689	CDS	MUIX01000002.1	48539	48363	-	177	prophage LambdaW5, minor tail protein Z, putative
fig|368602.8.peg.1690	CDS	MUIX01000002.1	48823	48575	-	249	Phage minor tail protein Z
fig|368602.8.peg.1692	CDS	MUIX01000002.1	49795	49127	-	669	Phage head, major capsid protein E
fig|368602.8.peg.1693	CDS	MUIX01000002.1	50153	49785	-	369	Phage head, head-tail preconnector protease C
fig|368602.8.peg.1694	CDS	MUIX01000002.1	51579	50167	-	1413	Phage head, portal protein B
fig|368602.8.peg.1696	CDS	MUIX01000002.1	53629	51794	-	1836	Phage head, terminase DNA packaging protein A
fig|368602.8.peg.1699	CDS	MUIX01000002.1	55614	55132	-	483	Phage head, terminase subunit Nu1
fig|368602.8.peg.1701	CDS	MUIX01000002.1	58285	57815	-	471	Phage head, terminase subunit Nu1
fig|368602.8.peg.1703	CDS	MUIX01000002.1	60114	59638	-	477	Phage protein
fig|368602.8.peg.1730	CDS	MUIX01000002.1	83815	84990	+	1176	Phage tail sheath protein FI
fig|368602.8.peg.1731	CDS	MUIX01000002.1	85006	85512	+	507	Phage tail tube protein FII
fig|368602.8.peg.1733	CDS	MUIX01000002.1	86639	86896	+	258	Phage tail protein E
fig|368602.8.peg.1735	CDS	MUIX01000002.1	87006	88790	+	1785	Phage tail length tape-measure protein T
fig|368602.8.peg.1738	CDS	MUIX01000002.1	90748	91923	+	1176	Phage tail sheath protein FI
fig|368602.8.peg.1739	CDS	MUIX01000002.1	91939	92445	+	507	Phage tail tube protein FII
fig|368602.8.peg.1741	CDS	MUIX01000002.1	93572	93829	+	258	Phage tail protein E
fig|368602.8.peg.1743	CDS	MUIX01000002.1	93939	95681	+	1743	Phage tail length tape-measure protein T
fig|368602.8.peg.1747	CDS	MUIX01000002.1	97729	98904	+	1176	Phage tail sheath protein FI
fig|368602.8.peg.1748	CDS	MUIX01000002.1	98920	99426	+	507	Phage tail tube protein FII
fig|368602.8.peg.1750	CDS	MUIX01000002.1	100553	100810	+	258	Phage tail protein E
fig|368602.8.peg.1752	CDS	MUIX01000002.1	100920	102311	+	1392	Phage tail length tape-measure protein T
fig|368602.8.peg.1753	CDS	MUIX01000002.1	102367	103179	+	813	Phage tail length tape-measure protein T
fig|368602.8.peg.1754	CDS	MUIX01000002.1	103193	103540	+	348	phage-related tail formation protein U
fig|368602.8.peg.1755	CDS	MUIX01000002.1	103537	103746	+	210	Phage tail protein GpX
fig|368602.8.peg.1756	CDS	MUIX01000002.1	103747	104691	+	945	Phage tail formation protein D
fig|368602.8.peg.1792	CDS	MUIX01000002.1	147856	149484	+	1629	Phage head, terminase DNA packaging protein A
fig|368602.8.peg.1795	CDS	MUIX01000002.1	150759	152165	+	1407	Phage head, portal protein B
fig|368602.8.peg.1813	CDS	MUIX01000002.1	171756	172931	+	1176	Phage tail sheath protein FI
fig|368602.8.peg.1814	CDS	MUIX01000002.1	172947	173453	+	507	Phage tail tube protein FII

	fig|368602.8.peg.1816	CDS	MUIX01000002.1	174580	174837	+	258	Phage tail protein E
	fig|368602.8.peg.1818	CDS	MUIX01000002.1	174947	177385	+	2439	Phage tail length tape-measure protein T
	fig|368602.8.peg.1819	CDS	MUIX01000002.1	177385	177723	+	339	prophage P2W3, tail protein U, putative
	fig|368602.8.peg.1820	CDS	MUIX01000002.1	177720	177929	+	210	Phage tail protein GpX
	fig|368602.8.peg.1821	CDS	MUIX01000002.1	177931	178881	+	951	Phage tail formation protein D
wTpre	fig|125593.4.peg.73	CDS	NZ_CM003641.1	55803	56231	+	429	Phage tail sheath protein FI
	fig|125593.4.peg.74	CDS	NZ_CM003641.1	56306	56494	+	189	Phage tail sheath protein FI
	fig|125593.4.peg.177	CDS	NZ_CM003641.1	136658	136957	+	300	prophage LambdaW5, ankyrin repeat domain protein
	fig|125593.4.peg.178	CDS	NZ_CM003641.1	137078	137233	+	156	prophage LambdaW5, ankyrin repeat domain protein
	fig|125593.4.peg.206	CDS	NZ_CM003641.1	158015	158200	+	186	uncharacterized phage protein, putative
	fig|125593.4.peg.217	CDS	NZ_CM003641.1	164222	164067	-	156	Phage major capsid protein
	fig|125593.4.peg.218	CDS	NZ_CM003641.1	164840	164577	-	264	Phage major capsid protein
	fig|125593.4.peg.734	CDS	NZ_CM003641.1	602681	603088	+	408	Phage tail fiber protein
	fig|125593.4.peg.735	CDS	NZ_CM003641.1	603110	603352	+	243	Phage tail fiber protein
	fig|125593.4.peg.736	CDS	NZ_CM003641.1	603382	603510	+	129	Phage tail fiber protein
	fig|125593.4.peg.737	CDS	NZ_CM003641.1	604108	604263	+	156	Phage tail fiber protein
	fig|125593.4.peg.739	CDS	NZ_CM003641.1	604411	604569	+	159	Phage tail fiber protein
	fig|125593.4.peg.740	CDS	NZ_CM003641.1	604538	604849	+	312	Phage tail fiber protein
	fig|125593.4.peg.814	CDS	NZ_CM003641.1	662026	661595	-	432	some similarities to phage related proteins
	fig|125593.4.peg.815	CDS	NZ_CM003641.1	662762	662151	-	612	some similarities to phage related proteins
	fig|125593.4.peg.953	CDS	NZ_CM003641.1	765303	764710	-	594	Phage tail fiber protein
	fig|125593.4.peg.954	CDS	NZ_CM003641.1	765641	765300	-	342	Phage tail fiber protein
	fig|125593.4.peg.1339	CDS	NZ_CM003641.1	1096320	1096535	+	216	Phage tail formation protein D
	fig|125593.4.peg.1363	CDS	NZ_CM003641.1	1115627	1115439	-	189	prophage LambdaW1, site-specific recombinase, resolvase family
	fig|125593.4.peg.1364	CDS	NZ_CM003641.1	1115961	1115839	-	123	prophage LambdaW1, site-specific recombinase, resolvase family
	fig|125593.4.peg.1365	CDS	NZ_CM003641.1	1116525	1115971	-	555	prophage LambdaW1, site-specific recombinase, resolvase family
wVulC	fig|1220511.6.peg.28	CDS	ALWU01000001.1	24120	27323	+	3204	Phage Host Specificity Protein
	fig|1220511.6.peg.118	CDS	ALWU01000001.1	117882	116743	-	1140	Phage major capsid protein
	fig|1220511.6.peg.137	CDS	ALWU01000001.1	129054	128713	-	342	prophage LambdaW1, site-specific recombinase, resolvase family
	fig|1220511.6.peg.367	CDS	ALWU01000001.1	313351	313692	+	342	prophage LambdaW1, site-specific recombinase, resolvase family
	fig|1220511.6.peg.703	CDS	ALWU01000002.1	17665	16730	-	936	Phage tail formation protein D
	fig|1220511.6.peg.704	CDS	ALWU01000002.1	17875	17666	-	210	Phage protein
	fig|1220511.6.peg.705	CDS	ALWU01000002.1	18225	17878	-	348	prophage P2W3, tail protein U, putative
	fig|1220511.6.peg.706	CDS	ALWU01000002.1	20495	18225	-	2271	Phage tail length tape-measure protein T
	fig|1220511.6.peg.707	CDS	ALWU01000002.1	20865	20611	-	255	Phage tail protein E
	fig|1220511.6.peg.708	CDS	ALWU01000002.1	21418	20912	-	507	Phage tail tube protein FII
	fig|1220511.6.peg.709	CDS	ALWU01000002.1	21827	21432	-	396	Phage tail sheath protein FI
	fig|1220511.6.peg.710	CDS	ALWU01000002.1	22614	21865	-	750	Phage tail sheath protein FI
	fig|1220511.6.peg.727	CDS	ALWU01000002.1	38746	39207	+	462	Phage protein
	fig|1220511.6.peg.730	CDS	ALWU01000002.1	42735	43217	+	483	Phage head, terminase subunit Nu1
	fig|1220511.6.peg.774	CDS	ALWU01000004.1	1192	8	-	1185	Phage tail length tape-measure protein T

fig|1220511.6.peg.775	CDS	ALWU01000004.1	1559	1302	-	258	Phage tail protein E
fig|1220511.6.peg.776	CDS	ALWU01000004.1	2140	1634	-	507	Phage tail tube protein FII
fig|1220511.6.peg.777	CDS	ALWU01000004.1	3333	2152	-	1182	Phage tail sheath protein FI
fig|1220511.6.peg.796	CDS	ALWU01000004.1	21208	21672	+	465	Phage protein
fig|1220511.6.peg.799	CDS	ALWU01000004.1	25150	25632	+	483	Phage head, terminase subunit Nu1
fig|1220511.6.peg.802	CDS	ALWU01000004.1	27108	28622	+	1515	Phage head, terminase DNA packaging protein A
fig|1220511.6.peg.806	CDS	ALWU01000004.1	30196	30306	+	111	Phage head, portal protein B
fig|1220511.6.peg.807	CDS	ALWU01000004.1	30319	31593	+	1275	Phage head, portal protein B
fig|1220511.6.peg.808	CDS	ALWU01000004.1	31593	32645	+	1053	Phage head, head-tail preconnector protease C
fig|1220511.6.peg.809	CDS	ALWU01000004.1	32692	33063	+	372	Phage head, head-DNA stabilization protein D
fig|1220511.6.peg.810	CDS	ALWU01000004.1	33088	34089	+	1002	Phage head, major capsid protein E
fig|1220511.6.peg.815	CDS	ALWU01000004.1	36957	37451	+	495	Phage minor tail protein Z
fig|1220511.6.peg.864	CDS	ALWU01000005.1	42324	42716	+	393	Phage tail fiber protein
fig|1220511.6.peg.911	CDS	ALWU01000005.1	87955	87524	-	432	Phage protein
fig|1220511.6.peg.914	CDS	ALWU01000005.1	90517	89351	-	1167	Phage tail formation protein I
fig|1220511.6.peg.916	CDS	ALWU01000005.1	91586	90804	-	783	Phage tail tip, host specificity protein J
fig|1220511.6.peg.917	CDS	ALWU01000005.1	91924	91589	-	336	Phage baseplate assembly protein GpW
fig|1220511.6.peg.919	CDS	ALWU01000005.1	92669	92205	-	465	Phage baseplate assembly protein V
fig|1220511.6.peg.920	CDS	ALWU01000005.1	93132	92656	-	477	Phage protein
fig|1220511.6.peg.921	CDS	ALWU01000005.1	93646	93152	-	495	Phage minor tail protein Z
fig|1220511.6.peg.926	CDS	ALWU01000005.1	97512	96511	-	1002	Phage head, major capsid protein E
fig|1220511.6.peg.927	CDS	ALWU01000005.1	97908	97537	-	372	Phage head, head-DNA stabilization protein D
fig|1220511.6.peg.928	CDS	ALWU01000005.1	98411	98106	-	306	Phage head, head-tail preconnector protease C
fig|1220511.6.peg.930	CDS	ALWU01000005.1	100660	99923	-	738	Phage head, head-tail preconnector protease C
fig|1220511.6.peg.931	CDS	ALWU01000005.1	102090	100657	-	1434	Phage head, portal protein B
fig|1220511.6.peg.934	CDS	ALWU01000005.1	104621	102801	-	1821	Phage head, terminase DNA packaging protein A
fig|1220511.6.peg.937	CDS	ALWU01000005.1	106584	106105	-	480	Phage head, terminase subunit Nu1
fig|1220511.6.peg.939	CDS	ALWU01000005.1	108371	107916	-	456	Phage protein
fig|1220511.6.peg.1038	CDS	ALWU01000005.1	189620	190084	+	465	Phage baseplate assembly protein V
fig|1220511.6.peg.1074	CDS	ALWU01000006.1	1821	1991	+	171	putative phage related protein
fig|1220511.6.peg.1075	CDS	ALWU01000006.1	2027	2518	+	492	Phage minor tail protein Z
fig|1220511.6.peg.1076	CDS	ALWU01000006.1	2538	3014	+	477	Phage protein
fig|1220511.6.peg.1077	CDS	ALWU01000006.1	3001	3465	+	465	Phage baseplate assembly protein V
fig|1220511.6.peg.1082	CDS	ALWU01000006.1	7492	7863	+	372	Phage baseplate assembly protein GpW
fig|1220511.6.peg.1083	CDS	ALWU01000006.1	7866	8648	+	783	Phage baseplate assembly protein J
fig|1220511.6.peg.1084	CDS	ALWU01000006.1	8648	9808	+	1161	Phage tail formation protein I
fig|1220511.6.peg.1087	CDS	ALWU01000006.1	11219	11650	+	432	Phage protein
fig|1220511.6.peg.1088	CDS	ALWU01000006.1	11653	13128	+	1476	prophage LambdaW1, site-specific recombinase, resolvase family
fig|1220511.6.peg.1111	CDS	ALWU01000006.1	31788	31444	-	345	prophage LambdaW1, site-specific recombinase, resolvase family
fig|1220511.6.peg.1192	CDS	ALWU01000008.1	54805	53426	-	1380	some similarities to phage related proteins
fig|1220511.6.peg.1300	CDS	ALWU01000008.1	156193	157626	+	1434	some similarities to phage related proteins

fig|1220511.6.peg.1323	CDS	ALWU01000008.1	173460	173119	-	342	prophage LambdaW1, site-specific recombinase, resolvase family
fig|1220511.6.peg.1336	CDS	ALWU01000009.1	805	26	-	780	Phage baseplate assembly protein J
fig|1220511.6.peg.1337	CDS	ALWU01000009.1	1143	808	-	336	Phage baseplate assembly protein GpW
fig|1220511.6.peg.1339	CDS	ALWU01000009.1	1861	1397	-	465	Phage baseplate assembly protein V
fig|1220511.6.peg.1340	CDS	ALWU01000009.1	2321	1848	-	474	Phage protein
fig|1220511.6.peg.1341	CDS	ALWU01000009.1	2817	2338	-	480	Phage minor tail protein Z
fig|1220511.6.peg.1342	CDS	ALWU01000009.1	3037	2819	-	219	putative phage related protein
fig|1220511.6.peg.1343	CDS	ALWU01000009.1	4214	3210	-	1005	Phage head, major capsid protein E
fig|1220511.6.peg.1344	CDS	ALWU01000009.1	4626	4252	-	375	Phage head, head-DNA stabilization protein D
fig|1220511.6.peg.1345	CDS	ALWU01000009.1	5748	4744	-	1005	Phage head, head-tail preconnector protease C
fig|1220511.6.peg.1346	CDS	ALWU01000009.1	7151	5745	-	1407	Phage head, portal protein B
fig|1220511.6.peg.1348	CDS	ALWU01000009.1	9201	7366	-	1836	Phage head, terminase DNA packaging protein A
fig|1220511.6.peg.1350	CDS	ALWU01000009.1	11187	10702	-	486	Phage head, terminase subunit Nu1
fig|1220511.6.peg.1352	CDS	ALWU01000009.1	12998	12534	-	465	Phage protein
fig|1220511.6.peg.1440	CDS	ALWU01000010.1	72470	73645	+	1176	Phage portal protein
fig|1220511.6.peg.1442	CDS	ALWU01000010.1	74923	75066	+	144	Phage portal protein
fig|1220511.6.peg.1528	CDS	ALWU01000010.1	147132	146815	-	318	prophage LambdaW1, site-specific recombinase, resolvase family
fig|1220511.6.peg.1529	CDS	ALWU01000010.1	148295	147099	-	1197	prophage LambdaW1, site-specific recombinase, resolvase family
fig|1220511.6.peg.1607	CDS	ALWU01000010.1	211437	211583	+	147	prophage LambdaW1, site-specific recombinase, resolvase family
fig|1220511.6.peg.1609	CDS	ALWU01000010.1	212090	212446	+	357	prophage LambdaW1, site-specific recombinase, resolvase family
fig|1220511.6.peg.1623	CDS	ALWU01000010.1	224236	222761	-	1476	prophage LambdaW1, site-specific recombinase, resolvase family
fig|1220511.6.peg.1624	CDS	ALWU01000010.1	224670	224239	-	432	Phage protein
fig|1220511.6.peg.1627	CDS	ALWU01000010.1	227250	226090	-	1161	Phage tail formation protein I
fig|1220511.6.peg.1628	CDS	ALWU01000010.1	228050	227250	-	801	Phage baseplate assembly protein J
fig|1220511.6.peg.1629	CDS	ALWU01000010.1	228388	228053	-	336	Phage baseplate assembly protein GpW
fig|1220511.6.peg.1631	CDS	ALWU01000010.1	229106	228642	-	465	Phage baseplate assembly protein V
fig|1220511.6.peg.1632	CDS	ALWU01000010.1	229575	229093	-	483	Phage protein
fig|1220511.6.peg.1633	CDS	ALWU01000010.1	230066	229590	-	477	Phage tail, component Z
fig|1220511.6.peg.1634	CDS	ALWU01000010.1	230285	230067	-	219	putative phage related protein
fig|1220511.6.peg.1639	CDS	ALWU01000010.1	235511	234510	-	1002	Phage head, major capsid protein E
fig|1220511.6.peg.1640	CDS	ALWU01000010.1	235912	235544	-	369	Phage head, head-DNA stabilization protein D
fig|1220511.6.peg.1641	CDS	ALWU01000010.1	237014	235980	-	1035	Phage head, head-tail preconnector protease C
fig|1220511.6.peg.1642	CDS	ALWU01000010.1	238411	237014	-	1398	Phage head, portal protein B
fig|1220511.6.peg.1644	CDS	ALWU01000010.1	240458	238944	-	1515	Phage head, terminase DNA packaging protein A
fig|1220511.6.peg.1647	CDS	ALWU01000010.1	242416	241934	-	483	Phage head, terminase subunit Nu1
fig|1220511.6.peg.1650	CDS	ALWU01000010.1	246405	245944	-	462	Phage protein
fig|1220511.6.peg.1672	CDS	ALWU01000010.1	262463	263665	+	1203	Phage tail sheath protein FI
fig|1220511.6.peg.1673	CDS	ALWU01000010.1	263677	264183	+	507	Phage tail tube protein FII
fig|1220511.6.peg.1675	CDS	ALWU01000010.1	265372	265629	+	258	Phage tail protein E
fig|1220511.6.peg.1677	CDS	ALWU01000010.1	265739	268039	+	2301	Phage tail length tape-measure protein T
fig|1220511.6.peg.1678	CDS	ALWU01000010.1	268039	268386	+	348	phage-related tail formation protein U

	fig|1220511.6.peg.1679	CDS	ALWU01000010.1	268383	268589	+	207	Phage tail protein GpX
	fig|1220511.6.peg.1680	CDS	ALWU01000010.1	268590	269537	+	948	Phage tail formation protein D
	fig|1220511.6.peg.1713	CDS	ALWU01000010.1	306984	306562	-	423	Phage head, terminase DNA packaging protein A
	fig|1220511.6.peg.1714	CDS	ALWU01000010.1	307174	306944	-	231	Phage head, terminase DNA packaging protein A
	fig|1220511.6.peg.1716	CDS	ALWU01000010.1	309114	308641	-	474	Phage head, terminase subunit Nu1
	fig|1220511.6.peg.1720	CDS	ALWU01000010.1	310801	310421	-	381	Phage protein
	fig|1220511.6.peg.1721	CDS	ALWU01000010.1	310889	310761	-	129	Phage protein
	fig|1220511.6.peg.1945	CDS	ALWU01000010.1	509327	509668	+	342	prophage LambdaW1, site-specific recombinase, resolvase family
wWb	fig|96496.9.peg.476	CDS	NZ_NJBR02000028.1	38534	38184	-	351	Phage major capsid protein
	fig|96496.9.peg.698	CDS	NZ_NJBR02000055.1	97	315	+	219	Phage uncharacterized protein
	fig|96496.9.peg.707	CDS	NZ_NJBR02000055.1	5944	6150	+	204	uncharacterized phage protein, putative
	fig|96496.9.peg.952	CDS	NZ_NJBR02000078.1	729	547	-	183	Phage major capsid protein

Table S11. List of ankyrin repeat domain protein sequences detected by RAST pipeline.

genome	Feature ID	Type	Contig	Start	Stop	Strand	Length (bp)	Function
wBm	fig|292805.13.peg.114	CDS	wBm_reseq_reversed	73102	72863	-	240	Ankyrin repeat domain protein
	fig|292805.13.peg.117	CDS	wBm_reseq_reversed	73863	73522	-	342	Ankyrin repeat domain protein
	fig|292805.13.peg.148	CDS	wBm_reseq_reversed	92642	91302	-	1341	Ankyrin repeat domain protein
	fig|292805.13.peg.359	CDS	wBm_reseq_reversed	244000	243626	-	375	ankyrin repeat domain protein
	fig|292805.13.peg.366	CDS	wBm_reseq_reversed	246465	246289	-	177	ankyrin repeat domain protein
	fig|292805.13.peg.451	CDS	wBm_reseq_reversed	305656	303566	-	2091	Ankyrin 3
	fig|292805.13.peg.768	CDS	wBm_reseq_reversed	527797	527916	+	120	ankyrin repeat domain protein
	fig|292805.13.peg.770	CDS	wBm_reseq_reversed	528230	528469	+	240	Ankyrin repeat domain protein
	fig|292805.13.peg.876	CDS	wBm_reseq_reversed	591256	590702	-	555	Ankyrin 1
	fig|292805.13.peg.1178	CDS	wBm_reseq_reversed	789538	789783	+	246	Ankyrin repeat-containing protein
	fig|292805.13.peg.1195	CDS	wBm_reseq_reversed	801082	802404	+	1323	Ankyrin repeat domain protein
	fig|292805.13.peg.1351	CDS	wBm_reseq_reversed	914605	914147	-	459	Ankyrin repeat-containing protein
	fig|292805.13.peg.1370	CDS	wBm_reseq_reversed	931551	931234	-	318	Ankyrin 1
	fig|292805.13.peg.1466	CDS	wBm_reseq_reversed	994127	997453	+	3327	Ankyrin repeat domain protein
wBp	fig|96495.5.peg.83	CDS	NZ_CP050521.1	68548	68694	+	147	ankyrin repeat domain protein
	fig|96495.5.peg.84	CDS	NZ_CP050521.1	68851	68994	+	144	ankyrin repeat domain protein
	fig|96495.5.peg.134	CDS	NZ_CP050521.1	106398	107738	+	1341	Ankyrin repeat domain protein
	fig|96495.5.peg.162	CDS	NZ_CP050521.1	125187	125528	+	342	Ankyrin repeat domain protein
	fig|96495.5.peg.164	CDS	NZ_CP050521.1	126044	126187	+	144	Ankyrin repeat domain protein
	fig|96495.5.peg.360	CDS	NZ_CP050521.1	284373	281047	-	3327	Ankyrin repeat domain protein
	fig|96495.5.peg.449	CDS	NZ_CP050521.1	360334	360915	+	582	Ankyrin repeat-containing protein
	fig|96495.5.peg.635	CDS	NZ_CP050521.1	519936	521258	+	1323	Ankyrin repeat domain protein
	fig|96495.5.peg.687	CDS	NZ_CP050521.1	564919	564788	-	132	Ankyrin repeat-containing protein
	fig|96495.5.peg.688	CDS	NZ_CP050521.1	565291	564962	-	330	Ankyrin repeat-containing protein
	fig|96495.5.peg.830	CDS	NZ_CP050521.1	681971	682525	+	555	Ankyrin 1
	fig|96495.5.peg.1006	CDS	NZ_CP050521.1	826430	826194	-	237	ankyrin repeat domain protein
	fig|96495.5.peg.1012	CDS	NZ_CP050521.1	828893	828717	-	177	ankyrin repeat domain protein
	fig|96495.5.peg.1079	CDS	NZ_CP050521.1	888052	885962	-	2091	Ankyrin 3
	fig|96495.5.peg.1173	CDS	NZ_CP050521.1	964250	964104	-	147	Ankyrin repeat domain protein
	fig|96495.5.peg.1174	CDS	NZ_CP050521.1	964912	964658	-	255	ankyrin repeat domain protein
wCauA	fig|2591635.5.peg.201	CDS	CP041215.1	184749	183538	-	1212	ankyrin repeat domain protein, putative
	fig|2591635.5.peg.290	CDS	CP041215.1	263255	263965	+	711	FOG: Ankyrin repeat
	fig|2591635.5.peg.320	CDS	CP041215.1	294202	293663	-	540	ankyrin repeat domain protein
	fig|2591635.5.peg.336	CDS	CP041215.1	308423	308959	+	537	ankyrin repeat domain protein
	fig|2591635.5.peg.428	CDS	CP041215.1	402304	405075	+	2772	ankyrin repeat domain protein
	fig|2591635.5.peg.477	CDS	CP041215.1	442575	441520	-	1056	ankyrin repeat domain protein
	fig|2591635.5.peg.484	CDS	CP041215.1	446226	445711	-	516	ankyrin repeat domain protein

	fig|2591635.5.peg.526	CDS	CP041215.1	481560	480694	-	867	ankyrin repeat domain protein
	fig|2591635.5.peg.588	CDS	CP041215.1	532168	531350	-	819	ankyrin repeat domain protein
	fig|2591635.5.peg.643	CDS	CP041215.1	595336	596529	+	1194	ankyrin repeat domain protein
	fig|2591635.5.peg.828	CDS	CP041215.1	775309	774353	-	957	ankyrin repeat domain protein
	fig|2591635.5.peg.834	CDS	CP041215.1	782160	782645	+	486	Ankyrin 2,3/unc44
	fig|2591635.5.peg.947	CDS	CP041215.1	885203	886486	+	1284	ankyrin repeat domain protein
	fig|2591635.5.peg.1002	CDS	CP041215.1	943133	940311	-	2823	ankyrin repeat domain protein
	fig|2591635.5.peg.1158	CDS	CP041215.1	1091324	1092034	+	711	FOG: Ankyrin repeat
	fig|2591635.5.peg.1187	CDS	CP041215.1	1120192	1120923	+	732	ankyrin repeat domain protein
	fig|2591635.5.peg.1238	CDS	CP041215.1	1180532	1177956	-	2577	ankyrin repeat domain protein
	fig|2591635.5.peg.1241	CDS	CP041215.1	1184408	1183206	-	1203	Ankyrin repeat domain protein
	fig|2591635.5.peg.1309	CDS	CP041215.1	1242840	1244204	+	1365	ankyrin repeat domain protein
wCtub	fig|6666666.333112.peg.64	CDS	wCtub_1_pilon_pilon	65291	66523	+	1233	ankyrin repeat domain protein
	fig|6666666.333112.peg.712	CDS	wCtub_1_pilon_pilon	773336	772614	-	723	Ankyrin repeat domain protein
wCfeJ	fig|212123.3.peg.23	CDS	CP051157.1	19644	19054	-	591	ankyrin repeat domain protein
	fig|212123.3.peg.26	CDS	CP051157.1	24735	21877	-	2859	ankyrin repeat domain protein
	fig|212123.3.peg.170	CDS	CP051157.1	159267	157360	-	1908	ankyrin repeat domain protein
	fig|212123.3.peg.255	CDS	CP051157.1	252846	254228	+	1383	ankyrin repeat domain protein
	fig|212123.3.peg.339	CDS	CP051157.1	347939	351184	+	3246	Ankyrin 3
	fig|212123.3.peg.500	CDS	CP051157.1	512440	513063	+	624	ankyrin repeat domain protein
	fig|212123.3.peg.523	CDS	CP051157.1	532548	531325	-	1224	Ankyrin repeat domain protein
	fig|212123.3.peg.824	CDS	CP051157.1	836134	837399	+	1266	similar to ankyrin 2,3/unc44
	fig|212123.3.peg.956	CDS	CP051157.1	980620	982188	+	1569	ankyrin repeat protein, putative
	fig|212123.3.peg.1023	CDS	CP051157.1	1059287	1060123	+	837	Ankyrin
	fig|212123.3.peg.1030	CDS	CP051157.1	1068174	1065370	-	2805	ankyrin repeat domain protein
	fig|212123.3.peg.1157	CDS	CP051157.1	1180619	1179558	-	1062	ankyrin repeat domain protein
wCfeT	fig|212123.4.peg.232	CDS	CP051156.1	223517	221640	-	1878	Ankyrin
	fig|212123.4.peg.263	CDS	CP051156.1	263288	262080	-	1209	ankyrin repeat domain protein
	fig|212123.4.peg.325	CDS	CP051156.1	324510	325271	+	762	ankyrin repeat domain protein
	fig|212123.4.peg.459	CDS	CP051156.1	460975	459788	-	1188	Ankyrin repeat domain protein
	fig|212123.4.peg.576	CDS	CP051156.1	576510	577058	+	549	ankyrin repeat domain protein
	fig|212123.4.peg.683	CDS	CP051156.1	684534	684055	-	480	ankyrin repeat domain protein
	fig|212123.4.peg.753	CDS	CP051156.1	763460	764476	+	1017	ankyrin repeat domain protein
	fig|212123.4.peg.796	CDS	CP051156.1	804877	805116	+	240	ankyrin repeat domain protein
	fig|212123.4.peg.1447	CDS	CP051156.1	1429394	1429915	+	522	ankyrin repeat domain protein
	fig|212123.4.peg.1453	CDS	CP051156.1	1432543	1433331	+	789	ankyrin repeat domain protein
wCle	fig|246273.9.peg.151	CDS	wCle_NZ_AP013028	144250	145662	+	1413	ankyrin repeat domain protein
	fig|246273.9.peg.169	CDS	wCle_NZ_AP013028	158182	157943	-	240	ankyrin repeat domain protein
	fig|246273.9.peg.299	CDS	wCle_NZ_AP013028	262640	264079	+	1440	ankyrin repeat domain protein
	fig|246273.9.peg.306	CDS	wCle_NZ_AP013028	268534	269130	+	597	Ankyrin
	fig|246273.9.peg.307	CDS	wCle_NZ_AP013028	269365	269934	+	570	prophage LambdaW1, ankyrin repeat domain protein

	fig|246273.9.peg.313	CDS	wCle_NZ_AP013028	274012	275277	+	1266	ankyrin repeat domain protein
	fig|246273.9.peg.532	CDS	wCle_NZ_AP013028	481043	480465	-	579	Ankyrin
	fig|246273.9.peg.698	CDS	wCle_NZ_AP013028	620688	621938	+	1251	ankyrin repeat domain protein
	fig|246273.9.peg.703	CDS	wCle_NZ_AP013028	625421	625684	+	264	ankyrin repeat domain protein
	fig|246273.9.peg.759	CDS	wCle_NZ_AP013028	676109	674745	-	1365	ankyrin repeat domain protein
	fig|246273.9.peg.920	CDS	wCle_NZ_AP013028	816276	815854	-	423	prophage LambdaW1, ankyrin repeat domain protein
	fig|246273.9.peg.921	CDS	wCle_NZ_AP013028	816422	816273	-	150	prophage LambdaW1, ankyrin repeat domain protein
	fig|246273.9.peg.922	CDS	wCle_NZ_AP013028	817421	816657	-	765	Ankyrin
	fig|246273.9.peg.948	CDS	wCle_NZ_AP013028	836503	837048	+	546	ankyrin repeat domain protein
	fig|246273.9.peg.1081	CDS	wCle_NZ_AP013028	948026	948271	+	246	ankyrin repeat domain protein
	fig|246273.9.peg.1231	CDS	wCle_NZ_AP013028	1089572	1088325	-	1248	Ankyrin repeat domain protein
	fig|246273.9.peg.1245	CDS	wCle_NZ_AP013028	1099171	1099581	+	411	ankyrin domain protein ank2
	fig|246273.9.peg.1247	CDS	wCle_NZ_AP013028	1101100	1101756	+	657	ankyrin repeat protein, putative
	fig|246273.9.peg.1372	CDS	wCle_NZ_AP013028	1214854	1214645	-	210	ankyrin repeat domain protein
	fig|246273.9.peg.1373	CDS	wCle_NZ_AP013028	1215789	1214950	-	840	ankyrin repeat domain protein
wDimm	fig|1812115.6.peg.87	CDS	canuwDimmCt1p0_circ_corr_ori_Copy_(reversed)	66360	63784	-	2577	Ankyrin repeat domain protein
	fig|1812115.6.peg.284	CDS	canuwDimmCt1p0_circ_corr_ori_Copy_(reversed)	236311	237453	+	1143	Ankyrin repeat domain protein
wDcau	fig|6666666.428248.peg.164	CDS	wDcau	188959	187757	-	1203	Ankyrin repeat domain protein
wFol	fig|169402.10.peg.217	CDS	wFol_NZ_CP015510	225797	227014	+	1218	ankyrin repeat protein, putative
	fig|169402.10.peg.234	CDS	wFol_NZ_CP015510	249370	247841	-	1530	ankyrin repeat domain protein
	fig|169402.10.peg.284	CDS	wFol_NZ_CP015510	302682	301432	-	1251	Ankyrin repeat domain protein
	fig|169402.10.peg.304	CDS	wFol_NZ_CP015510	321595	320990	-	606	Ankyrin
	fig|169402.10.peg.305	CDS	wFol_NZ_CP015510	322471	321800	-	672	Ankyrin
	fig|169402.10.peg.335	CDS	wFol_NZ_CP015510	360679	359891	-	789	Putative ankyrin repeat protein RF_0381
	fig|169402.10.peg.402	CDS	wFol_NZ_CP015510	416676	417902	+	1227	ankyrin repeat domain protein
	fig|169402.10.peg.515	CDS	wFol_NZ_CP015510	570503	571501	+	999	ankyrin repeat domain protein
	fig|169402.10.peg.554	CDS	wFol_NZ_CP015510	615056	618346	+	3291	ankyrin domain protein ank2
	fig|169402.10.peg.602	CDS	wFol_NZ_CP015510	658368	657838	-	531	ankyrin repeat domain protein
	fig|169402.10.peg.686	CDS	wFol_NZ_CP015510	727231	727890	+	660	Ankyrin
	fig|169402.10.peg.747	CDS	wFol_NZ_CP015510	802088	803296	+	1209	ankyrin repeat domain protein
	fig|169402.10.peg.1005	CDS	wFol_NZ_CP015510	1066904	1066380	-	525	ankyrin repeat domain protein
	fig|169402.10.peg.1041	CDS	wFol_NZ_CP015510	1115293	1114622	-	672	Ankyrin
	fig|169402.10.peg.1092	CDS	wFol_NZ_CP015510	1159631	1160209	+	579	ankyrin repeat domain protein
	fig|169402.10.peg.1093	CDS	wFol_NZ_CP015510	1160371	1160940	+	570	ankyrin repeat domain protein
	fig|169402.10.peg.1176	CDS	wFol_NZ_CP015510	1250521	1251180	+	660	Ankyrin
	fig|169402.10.peg.1195	CDS	wFol_NZ_CP015510	1286321	1283064	-	3258	Ankyrin 3
wMel	fig|163164.1.peg.32	CDS	wMel_NC_002978	37671	38537	+	867	ankyrin repeat domain protein
	fig|163164.1.peg.65	CDS	wMel_NC_002978	68396	70798	+	2403	ankyrin repeat domain protein
	fig|163164.1.peg.130	CDS	wMel_NC_002978	133174	136038	+	2865	ankyrin repeat domain protein
	fig|163164.1.peg.168	CDS	wMel_NC_002978	175126	174320	-	807	ankyrin repeat domain protein
	fig|163164.1.peg.252	CDS	wMel_NC_002978	265391	265993	+	603	prophage LambdaW1, ankyrin repeat domain protein

	fig|163164.1.peg.253	CDS	wMel_NC_002978	266255	266845	+	591	prophage LambdaW1, ankyrin repeat domain protein
	fig|163164.1.peg.258	CDS	wMel_NC_002978	271048	270374	-	675	prophage LambdaW1, ankyrin repeat domain protein
	fig|163164.1.peg.259	CDS	wMel_NC_002978	273255	271150	-	2106	prophage LambdaW1, ankyrin repeat domain protein
	fig|163164.1.peg.261	CDS	wMel_NC_002978	275225	273600	-	1626	ankyrin repeat domain protein
	fig|163164.1.peg.342	CDS	wMel_NC_002978	367326	368954	+	1629	ankyrin repeat domain protein
	fig|163164.1.peg.392	CDS	wMel_NC_002978	422022	420124	-	1899	ankyrin repeat domain protein
	fig|163164.1.peg.395	CDS	wMel_NC_002978	425912	424704	-	1209	ankyrin repeat domain protein
	fig|163164.1.peg.446	CDS	wMel_NC_002978	479223	480230	+	1008	ankyrin repeat domain protein
	fig|163164.1.peg.459	CDS	wMel_NC_002978	507200	505791	-	1410	ankyrin repeat domain protein
	fig|163164.1.peg.489	CDS	wMel_NC_002978	538083	537094	-	990	ankyrin repeat domain protein
	fig|163164.1.peg.503	CDS	wMel_NC_002978	551597	551076	-	522	ankyrin repeat domain protein
	fig|163164.1.peg.530	CDS	wMel_NC_002978	573982	575463	+	1482	prophage LambdaW4, ankyrin repeat domain protein
	fig|163164.1.peg.563	CDS	wMel_NC_002978	623343	626243	+	2901	prophage LambdaW5, ankyrin repeat domain protein
	fig|163164.1.peg.566	CDS	wMel_NC_002978	629024	628566	-	459	prophage LambdaW5, ankyrin repeat domain protein
	fig|163164.1.peg.567	CDS	wMel_NC_002978	629676	629050	-	627	prophage LambdaW5, ankyrin repeat domain protein
	fig|163164.1.peg.675	CDS	wMel_NC_002978	729022	727721	-	1302	ankyrin repeat domain protein
	fig|163164.1.peg.687	CDS	wMel_NC_002978	738997	740421	+	1425	ankyrin repeat domain protein
	fig|163164.1.peg.1090	CDS	wMel_NC_002978	1160993	1159749	-	1245	ankyrin repeat domain protein, putative
wMhie	fig|1241303.4.peg.485	CDS	NODE_2425_length_8960_cov_76.725110_wb_F	4985	6232	+	1248	Ankyrin repeat domain protein
	fig|1241303.4.peg.533	CDS	NODE_2622_length_8194_cov_99.073183_wb_F	8114	6366	-	1749	ankyrin repeat domain protein
	fig|1241303.4.peg.833	CDS	NODE_4004_length_4861_cov_83.281971_wb_F	4187	4699	+	513	ankyrin repeat domain protein
	fig|1241303.4.peg.889	CDS	NODE_4371_length_4323_cov_72.559074_wb_F	333	2372	+	2040	Ankyrin repeat domain protein
	fig|1241303.4.peg.1182	CDS	NODE_7789_length_2150_cov_103.059738	1568	204	-	1365	ankyrin repeat domain protein
wLbra	fig|1812117.6.peg.155	CDS	NODE_153_length_129046_cov_595.533057_wb	37638	40688	+	3051	Ankyrin repeat domain protein
	fig|1812117.6.peg.1035	CDS	NODE_444_length_40856_cov_557.980812_wb	224	358	+	135	Ankyrin repeat domain protein
	fig|1812117.6.peg.1038	CDS	NODE_444_length_40856_cov_557.980812_wb	1246	1518	+	273	Ankyrin repeat domain protein
wLsig	fig|80850.8.peg.1170	CDS	canuwLsigCt1p4_wLsig_tig1_corr_circularized_ori	833976	830653	-	3324	Ankyrin repeat domain protein
wLug	fig|1335053.6.peg.6	CDS	MUIY01000001.1	3064	3606	+	543	ankyrin repeat domain protein
	fig|1335053.6.peg.89	CDS	MUIY01000001.1	80198	80731	+	534	ankyrin repeat domain protein
	fig|1335053.6.peg.323	CDS	MUIY01000001.1	304990	304124	-	867	ankyrin repeat domain protein
	fig|1335053.6.peg.434	CDS	MUIY01000001.1	417440	417153	-	288	ankyrin repeat protein, putative
	fig|1335053.6.peg.435	CDS	MUIY01000001.1	418000	417443	-	558	ankyrin repeat protein, putative
	fig|1335053.6.peg.447	CDS	MUIY01000001.1	428259	428426	+	168	ankyrin repeat protein, putative
	fig|1335053.6.peg.627	CDS	MUIY01000001.1	584055	582916	-	1140	ankyrin repeat domain protein, putative
	fig|1335053.6.peg.743	CDS	MUIY01000001.1	684266	682170	-	2097	ankyrin repeat domain protein
	fig|1335053.6.peg.746	CDS	MUIY01000001.1	687966	686911	-	1056	Ankyrin repeat domain protein
	fig|1335053.6.peg.768	CDS	MUIY01000001.1	709727	711067	+	1341	ankyrin repeat domain protein
	fig|1335053.6.peg.774	CDS	MUIY01000001.1	717607	716420	-	1188	ankyrin repeat domain protein
	fig|1335053.6.peg.784	CDS	MUIY01000001.1	726030	725113	-	918	ankyrin domain protein ank2
	fig|1335053.6.peg.996	CDS	MUIY01000001.1	934070	933273	-	798	ankyrin repeat domain protein
	fig|1335053.6.peg.1065	CDS	MUIY01000001.1	1002512	1000857	-	1656	ankyrin repeat domain protein

	fig|1335053.6.peg.1194	CDS	MUIY01000001.1	1121977	1121066	-	912	ankyrin repeat domain protein
	fig|1335053.6.peg.1341	CDS	MUIY01000002.1	122358	121471	-	888	ankyrin repeat protein, putative
	fig|1335053.6.peg.1348	CDS	MUIY01000002.1	129065	127929	-	1137	ankyrin domain protein ank2
	fig|1335053.6.peg.1457	CDS	MUIY01000002.1	230560	234033	+	3474	Ankyrin 3
	fig|1335053.6.peg.1490	CDS	MUIY01000002.1	266538	265498	-	1041	ankyrin repeat domain protein
	fig|1335053.6.peg.1491	CDS	MUIY01000002.1	267549	266959	-	591	ankyrin repeat domain protein
	fig|1335053.6.peg.1492	CDS	MUIY01000002.1	267878	267594	-	285	ankyrin repeat domain protein
	fig|1335053.6.peg.1601	CDS	MUIY01000002.1	360277	360813	+	537	ankyrin repeat domain protein
wNfla	fig|1854759.6.peg.321	CDS	NZ_LYUW01000008.1	18514	20700	+	2187	ankyrin repeat domain protein
	fig|1854759.6.peg.371	CDS	NZ_LYUW01000010.1	15180	13234	-	1947	ankyrin repeat domain protein
	fig|1854759.6.peg.421	CDS	NZ_LYUW01000012.1	10105	9704	-	402	prophage LambdaW1, ankyrin repeat domain protein
	fig|1854759.6.peg.422	CDS	NZ_LYUW01000012.1	10236	10102	-	135	prophage LambdaW1, ankyrin repeat domain protein
	fig|1854759.6.peg.555	CDS	NZ_LYUW01000018.1	17712	17179	-	534	ankyrin repeat domain protein
	fig|1854759.6.peg.580	CDS	NZ_LYUW01000019.1	19030	17774	-	1257	ankyrin repeat domain protein
	fig|1854759.6.peg.647	CDS	NZ_LYUW01000023.1	47	775	+	729	Ankyrin repeat domain protein
	fig|1854759.6.peg.651	CDS	NZ_LYUW01000023.1	3869	5746	+	1878	ankyrin repeat domain protein
	fig|1854759.6.peg.817	CDS	NZ_LYUW01000033.1	12267	11776	-	492	ankyrin repeat domain protein
	fig|1854759.6.peg.1038	CDS	NZ_LYUW01000049.1	1789	2655	+	867	ankyrin repeat domain protein
	fig|1854759.6.peg.1098	CDS	NZ_LYUW01000054.1	7183	6872	-	312	ankyrin repeat domain protein
	fig|1854759.6.peg.1125	CDS	NZ_LYUW01000057.1	925	29	-	897	ankyrin repeat domain protein
	fig|1854759.6.peg.1156	CDS	NZ_LYUW01000060.1	5845	6642	+	798	ankyrin repeat domain protein, putative
	fig|1854759.6.peg.1157	CDS	NZ_LYUW01000060.1	6693	7043	+	351	ankyrin repeat domain protein, putative
	fig|1854759.6.peg.1195	CDS	NZ_LYUW01000064.1	5621	5034	-	588	ankyrin repeat domain protein
	fig|1854759.6.peg.1196	CDS	NZ_LYUW01000064.1	6992	5763	-	1230	ankyrin repeat domain protein
	fig|1854759.6.peg.1223	CDS	NZ_LYUW01000068.1	4831	3896	-	936	ankyrin repeat domain protein
	fig|1854759.6.peg.1260	CDS	NZ_LYUW01000073.1	1591	1866	+	276	ankyrin repeat domain protein
	fig|1854759.6.peg.1319	CDS	NZ_LYUW01000081.1	3885	4409	+	525	ankyrin repeat domain protein
	fig|1854759.6.peg.1356	CDS	NZ_LYUW01000089.1	47	397	+	351	ankyrin repeat domain protein
	fig|1854759.6.peg.1442	CDS	NZ_LYUW01000111.1	335	1699	+	1365	ankyrin repeat domain protein
wOo	fig|6666666.201426.peg.17	CDS	wOo	14070	13042	-	1029	ankyrin repeat domain protein
	fig|6666666.201426.peg.249	CDS	wOo	275178	274990	-	189	Ankyrin 3
wOv	fig|1410384.7.peg.552	CDS	wOo_NZ_HG810405	609948	608794	-	1155	ankyrin repeat domain protein
	fig|1410384.7.peg.784	CDS	wOo_NZ_HG810405	871356	871168	-	189	Ankyrin 3
wPip	fig|955.1.peg.8	CDS	wPip_culex15b06	8463	9614	+	1152	UNC-44 ankyrins
	fig|955.1.peg.9	CDS	wPip_culex15b06	10040	11734	+	1695	ankyrin repeat family protein
	fig|955.1.peg.112	CDS	culex8d01.q1ka	22400	23569	+	1170	Ankyrin 2
	fig|955.1.peg.144	CDS	culex8d01.q1ka	58928	55518	-	3411	Ankyrin 3
	fig|955.1.peg.157	CDS	culex8d01.q1ka	75760	76620	+	861	Ankyrin 1
	fig|955.1.peg.325	CDS	culex61a11.q1k	3975	3112	-	864	ankyrin repeat family protein
	fig|955.1.peg.447	CDS	culex17f04.q1k	2806	2282	-	525	Chain A, Sank E3_5: An Artificial Ankyrin Repeat Protein
	fig|955.1.peg.479	CDS	culex141a08.q1k	1463	921	-	543	Ankyrin

	fig|955.1.peg.570	CDS	culex107d08.p1k	5240	3	-	5238	UNC-44 ankyrins
	fig|955.1.peg.742	CDS	culex111d03.p1k	20757	18430	-	2328	Ankyrin 1
	fig|955.1.peg.749	CDS	culex111d03.p1k	32311	29954	-	2358	Ankyrin 2
	fig|955.1.peg.863	CDS	culex40h12.p1k	18377	10131	-	8247	Ankyrin 2
	fig|955.1.peg.913	CDS	culex18h04.p1k	2125	2649	+	525	Chain A, Sank E3_5: An Artificial Ankyrin Repeat Protein
	fig|955.1.peg.1011	CDS	culex180a01.p1k	7163	9139	+	1977	Ankyrin 3
	fig|955.1.peg.1179	CDS	culex79g04.p1k	57593	58432	+	840	Ankyrin
	fig|955.1.peg.1183	CDS	culex79g04.p1k	61738	62283	+	546	Ankyrin 2
	fig|955.1.peg.1192	CDS	culex79g04.p1k	73793	74866	+	1074	Ankyrin 1
	fig|955.1.peg.1504	CDS	culex160e12.q1k	22114	22710	+	597	Ankyrin 1
wPpe	fig|6666666.425803.peg.122	CDS	wPpe_NZ_MJMG01000001	110793	109780	-	1014	Ankyrin repeat domain protein
	fig|6666666.425803.peg.393	CDS	NZ_MJMG01000002.1	18926	17715	-	1212	ankyrin repeat domain protein
	fig|6666666.425803.peg.443	CDS	NZ_MJMG01000004.1	6283	5336	-	948	FOG: Ankyrin repeat
	fig|6666666.425803.peg.605	CDS	NZ_MJMG01000006.1	71915	70542	-	1374	ankyrin repeat domain protein
	fig|6666666.425803.peg.984	CDS	NZ_MJMG01000011.1	32291	33304	+	1014	ankyrin repeat domain protein
wstri	fig|368602.8.peg.7	CDS	MUIX01000001.1	7598	6870	-	729	FOG: Ankyrin repeat
	fig|368602.8.peg.49	CDS	MUIX01000001.1	43212	44108	+	897	ankyrin repeat domain protein
	fig|368602.8.peg.157	CDS	MUIX01000001.1	139951	140436	+	486	ankyrin repeat domain protein
	fig|368602.8.peg.244	CDS	MUIX01000001.1	234409	235461	+	1053	Putative ankyrin repeat protein RF_0381
	fig|368602.8.peg.248	CDS	MUIX01000001.1	239031	238327	-	705	FOG: Ankyrin repeat
	fig|368602.8.peg.286	CDS	MUIX01000001.1	263993	264355	+	363	Ankyrin repeat domain protein
	fig|368602.8.peg.293	CDS	MUIX01000001.1	277159	276431	-	729	FOG: Ankyrin repeat
	fig|368602.8.peg.482	CDS	MUIX01000001.1	456434	457345	+	912	ankyrin domain protein ank2
	fig|368602.8.peg.492	CDS	MUIX01000001.1	464826	466013	+	1188	ankyrin repeat domain protein
	fig|368602.8.peg.496	CDS	MUIX01000001.1	472000	470660	-	1341	ankyrin repeat domain protein
	fig|368602.8.peg.517	CDS	MUIX01000001.1	492284	493264	+	981	Ankyrin repeat domain protein
	fig|368602.8.peg.642	CDS	MUIX01000001.1	640888	639896	-	993	ankyrin repeat protein, putative
	fig|368602.8.peg.733	CDS	MUIX01000001.1	743574	747008	+	3435	Ankyrin 3
	fig|368602.8.peg.768	CDS	MUIX01000001.1	782603	782064	-	540	ankyrin repeat domain protein
	fig|368602.8.peg.937	CDS	MUIX01000001.1	935303	934164	-	1140	ankyrin repeat domain protein, putative
	fig|368602.8.peg.994	CDS	MUIX01000001.1	983420	984178	+	759	ankyrin repeat protein, putative
	fig|368602.8.peg.1003	CDS	MUIX01000001.1	990385	990140	-	246	ankyrin repeat protein, putative
	fig|368602.8.peg.1131	CDS	MUIX01000001.1	1121774	1122640	+	867	ankyrin repeat domain protein
	fig|368602.8.peg.1228	CDS	MUIX01000001.1	1209426	1209022	-	405	ankyrin repeat domain protein
	fig|368602.8.peg.1290	CDS	MUIX01000001.1	1270986	1272044	+	1059	similar to ankyrin 2,3/unc44
	fig|368602.8.peg.1363	CDS	MUIX01000001.1	1331331	1331846	+	516	ankyrin repeat domain protein
	fig|368602.8.peg.1392	CDS	MUIX01000001.1	1371763	1371035	-	729	FOG: Ankyrin repeat
	fig|368602.8.peg.1473	CDS	MUIX01000001.1	1442122	1444113	+	1992	ankyrin repeat domain protein
	fig|368602.8.peg.1520	CDS	MUIX01000001.1	1486802	1486284	-	519	ankyrin repeat domain protein
	fig|368602.8.peg.1608	CDS	MUIX01000001.1	1584441	1583926	-	516	ankyrin repeat domain protein
	fig|368602.8.peg.1622	CDS	MUIX01000001.1	1594252	1593737	-	516	ankyrin repeat domain protein

	fig|368602.8.peg.1638	CDS	MUIX01000002.1	5506	5015	-	492	ankyrin repeat domain protein
	fig|368602.8.peg.1672	CDS	MUIX01000002.1	38437	37748	-	690	FOG: Ankyrin repeat
	fig|368602.8.peg.1757	CDS	MUIX01000002.1	104744	105277	+	534	ankyrin repeat domain protein
	fig|368602.8.peg.1822	CDS	MUIX01000002.1	178919	179410	+	492	ankyrin repeat domain protein
wTpre	fig|125593.4.peg.1	CDS	NZ_CM003641.1	493	2	-	492	ankyrin repeat domain protein
	fig|125593.4.peg.40	CDS	NZ_CM003641.1	33476	33024	-	453	conserved hypothetical protein (Ankyrin repeat domain)
	fig|125593.4.peg.42	CDS	NZ_CM003641.1	34357	33956	-	402	ankyrin repeat domain protein
	fig|125593.4.peg.43	CDS	NZ_CM003641.1	34519	34406	-	114	ankyrin repeat domain protein
	fig|125593.4.peg.44	CDS	NZ_CM003641.1	34699	34565	-	135	ankyrin repeat domain protein
	fig|125593.4.peg.175	CDS	NZ_CM003641.1	135581	136018	+	438	ankyrin repeat domain protein
	fig|125593.4.peg.177	CDS	NZ_CM003641.1	136658	136957	+	300	prophage LambdaW5, ankyrin repeat domain protein
	fig|125593.4.peg.178	CDS	NZ_CM003641.1	137078	137233	+	156	prophage LambdaW5, ankyrin repeat domain protein
	fig|125593.4.peg.224	CDS	NZ_CM003641.1	169242	169376	+	135	ankyrin repeat domain protein
	fig|125593.4.peg.225	CDS	NZ_CM003641.1	169393	169656	+	264	ankyrin repeat domain protein
	fig|125593.4.peg.226	CDS	NZ_CM003641.1	169778	169927	+	150	ankyrin repeat domain protein
	fig|125593.4.peg.370	CDS	NZ_CM003641.1	289388	289798	+	411	ankyrin repeat domain protein
	fig|125593.4.peg.371	CDS	NZ_CM003641.1	289864	290004	+	141	ankyrin repeat domain protein
	fig|125593.4.peg.415	CDS	NZ_CM003641.1	325025	325177	+	153	ankyrin repeat domain protein
	fig|125593.4.peg.459	CDS	NZ_CM003641.1	362087	361986	-	102	similar to ankyrin 2,3/unc44
	fig|125593.4.peg.460	CDS	NZ_CM003641.1	362339	362091	-	249	similar to ankyrin 2,3/unc44
	fig|125593.4.peg.461	CDS	NZ_CM003641.1	362815	362393	-	423	similar to ankyrin 2,3/unc44
	fig|125593.4.peg.539	CDS	NZ_CM003641.1	437432	437217	-	216	ankyrin repeat domain protein
	fig|125593.4.peg.549	CDS	NZ_CM003641.1	445846	446034	+	189	ankyrin repeat domain protein
	fig|125593.4.peg.664	CDS	NZ_CM003641.1	531803	531420	-	384	ankyrin repeat domain protein
	fig|125593.4.peg.666	CDS	NZ_CM003641.1	532311	532144	-	168	ankyrin repeat domain protein
	fig|125593.4.peg.762	CDS	NZ_CM003641.1	624401	624144	-	258	ankyrin repeat domain protein
	fig|125593.4.peg.763	CDS	NZ_CM003641.1	624531	624418	-	114	ankyrin repeat domain protein
	fig|125593.4.peg.764	CDS	NZ_CM003641.1	624695	624552	-	144	ankyrin repeat domain protein
	fig|125593.4.peg.765	CDS	NZ_CM003641.1	624874	624701	-	174	ankyrin repeat domain protein
	fig|125593.4.peg.766	CDS	NZ_CM003641.1	625072	624878	-	195	ankyrin repeat domain protein
	fig|125593.4.peg.767	CDS	NZ_CM003641.1	625416	625093	-	324	ankyrin repeat domain protein
	fig|125593.4.peg.776	CDS	NZ_CM003641.1	630150	630272	+	123	ankyrin repeat protein, putative
	fig|125593.4.peg.777	CDS	NZ_CM003641.1	630329	630628	+	300	ankyrin repeat protein, putative
	fig|125593.4.peg.778	CDS	NZ_CM003641.1	630777	630914	+	138	ankyrin repeat protein, putative
	fig|125593.4.peg.883	CDS	NZ_CM003641.1	711136	714609	+	3474	Ankyrin 3
	fig|125593.4.peg.946	CDS	NZ_CM003641.1	762030	761896	-	135	ankyrin repeat protein, putative
	fig|125593.4.peg.1077	CDS	NZ_CM003641.1	858481	858344	-	138	ankyrin repeat domain protein, putative
	fig|125593.4.peg.1078	CDS	NZ_CM003641.1	858894	858694	-	201	ankyrin repeat domain protein, putative
	fig|125593.4.peg.1103	CDS	NZ_CM003641.1	880600	881127	+	528	ankyrin repeat domain protein
	fig|125593.4.peg.1137	CDS	NZ_CM003641.1	907323	907613	+	291	ankyrin repeat domain protein
	fig|125593.4.peg.1138	CDS	NZ_CM003641.1	907803	907973	+	171	ankyrin repeat domain protein

	fig|125593.4.peg.1139	CDS	NZ_CM003641.1	907998	908279	+	282	Ankyrin 1
	fig|125593.4.peg.1140	CDS	NZ_CM003641.1	908599	909780	+	1182	ankyrin repeat domain protein
	fig|125593.4.peg.1189	CDS	NZ_CM003641.1	951499	951756	+	258	ankyrin domain protein ank2
	fig|125593.4.peg.1190	CDS	NZ_CM003641.1	951812	951994	+	183	ankyrin domain protein ank2
	fig|125593.4.peg.1251	CDS	NZ_CM003641.1	1009740	1009895	+	156	ankyrin repeat domain protein
	fig|125593.4.peg.1335	CDS	NZ_CM003641.1	1094662	1094036	-	627	FOG: Ankyrin repeat
	fig|125593.4.peg.1340	CDS	NZ_CM003641.1	1096663	1096818	+	156	ankyrin repeat domain protein
	fig|125593.4.peg.1341	CDS	NZ_CM003641.1	1096860	1097021	+	162	ankyrin repeat domain protein
	fig|125593.4.peg.1342	CDS	NZ_CM003641.1	1097018	1097194	+	177	ankyrin repeat domain protein
	fig|125593.4.peg.1371	CDS	NZ_CM003641.1	1120976	1122316	+	1341	ankyrin repeat domain protein
	fig|125593.4.peg.1379	CDS	NZ_CM003641.1	1130329	1128287	-	2043	ankyrin repeat domain protein
	fig|125593.4.peg.1382	CDS	NZ_CM003641.1	1133807	1132989	-	819	Ankyrin repeat domain protein
wVulC	fig|1220511.6.peg.182	CDS	ALWU01000001.1	169776	168436	-	1341	ankyrin repeat domain protein
	fig|1220511.6.peg.256	CDS	ALWU01000001.1	226104	225553	-	552	Ankyrin repeat domain protein
	fig|1220511.6.peg.488	CDS	ALWU01000001.1	418030	418224	+	195	ankyrin repeat domain protein
	fig|1220511.6.peg.516	CDS	ALWU01000001.1	435504	434161	-	1344	ankyrin repeat domain protein
	fig|1220511.6.peg.562	CDS	ALWU01000001.1	470805	470467	-	339	Ankyrin repeat domain protein
	fig|1220511.6.peg.569	CDS	ALWU01000001.1	481106	480210	-	897	ankyrin repeat domain protein
	fig|1220511.6.peg.636	CDS	ALWU01000001.1	548972	548259	-	714	ankyrin repeat domain protein
	fig|1220511.6.peg.683	CDS	ALWU01000002.1	1104	1871	+	768	ankyrin repeat domain protein
	fig|1220511.6.peg.702	CDS	ALWU01000002.1	16677	16141	-	537	ankyrin repeat domain protein
	fig|1220511.6.peg.835	CDS	ALWU01000005.1	18276	19259	+	984	Ankyrin repeat domain protein
	fig|1220511.6.peg.838	CDS	ALWU01000005.1	21959	24043	+	2085	ankyrin repeat domain protein
	fig|1220511.6.peg.1085	CDS	ALWU01000006.1	9977	10678	+	702	FOG: Ankyrin repeat
	fig|1220511.6.peg.1118	CDS	ALWU01000006.1	35307	36791	+	1485	ankyrin repeat domain protein
	fig|1220511.6.peg.1145	CDS	ALWU01000008.1	9582	8854	-	729	ankyrin repeat domain protein
	fig|1220511.6.peg.1160	CDS	ALWU01000008.1	25019	24630	-	390	conserved hypothetical protein (Ankyrin repeat domain)
	fig|1220511.6.peg.1303	CDS	ALWU01000008.1	159709	160134	+	426	ankyrin repeat domain protein
	fig|1220511.6.peg.1441	CDS	ALWU01000010.1	73725	74813	+	1089	ankyrin repeat domain protein
	fig|1220511.6.peg.1507	CDS	ALWU01000010.1	130853	130251	-	603	ankyrin domain protein ank2
	fig|1220511.6.peg.1615	CDS	ALWU01000010.1	214812	215984	+	1173	ankyrin repeat domain protein
	fig|1220511.6.peg.1827	CDS	ALWU01000010.1	401103	404606	+	3504	Ankyrin 3
	fig|1220511.6.peg.1861	CDS	ALWU01000010.1	436267	436094	-	174	ankyrin repeat domain protein
	fig|1220511.6.peg.1862	CDS	ALWU01000010.1	437153	436341	-	813	Ankyrin 1
	fig|1220511.6.peg.1863	CDS	ALWU01000010.1	438568	437408	-	1161	ankyrin repeat domain protein
	fig|1220511.6.peg.1881	CDS	ALWU01000010.1	452153	451014	-	1140	ankyrin repeat domain protein
wWb	fig|96496.9.peg.22	CDS	wWb_NZ_NJBR02000002	23837	22472	-	1347	ankyrin repeat domain protein
	fig|96496.9.peg.46	CDS	wWb_NZ_NJBR02000002	42646	41306	-	1341	ankyrin repeat domain protein
	fig|96496.9.peg.514	CDS	NZ_NJBR02000035.1	859	981	+	123	ankyrin repeat domain protein
	fig|96496.9.peg.517	CDS	NZ_NJBR02000035.1	1422	1568	+	147	ankyrin repeat domain protein
	fig|96496.9.peg.620	CDS	NZ_NJBR02000044.1	5792	5676	-	117	Ankyrin repeat-containing protein

fig|96496.9.peg.621	CDS	NZ_NJBR02000044.1	5997	5758	-	240	Ankyrin repeat-containing protein
fig|96496.9.peg.741	CDS	NZ_NJBR02000058.1	2128	908	-	1221	ankyrin repeat domain protein
fig|96496.9.peg.904	CDS	NZ_NJBR02000073.1	25943	26191	+	249	ankyrin repeat domain protein
fig|96496.9.peg.906	CDS	NZ_NJBR02000073.1	26461	26613	+	153	Ankyrin repeat domain protein
fig|96496.9.peg.914	CDS	NZ_NJBR02000073.1	31586	32056	+	471	Ankyrin 1
fig|96496.9.peg.1012	CDS	NZ_NJBR02000084.1	497	249	-	249	ankyrin repeat domain protein
